# Supplementary figures and images for: Taxonomic, Genomic, and Functional Variation in the Gut Microbiomes of Wild Spotted Hyenas Across 2 Decades of Study
Source: mSystems. 2022 Dec 19;8(1):e00965-22. doi: 10.1128/msystems.00965-22 (PMC9948708; doi:10.1128/msystems.00965-22)

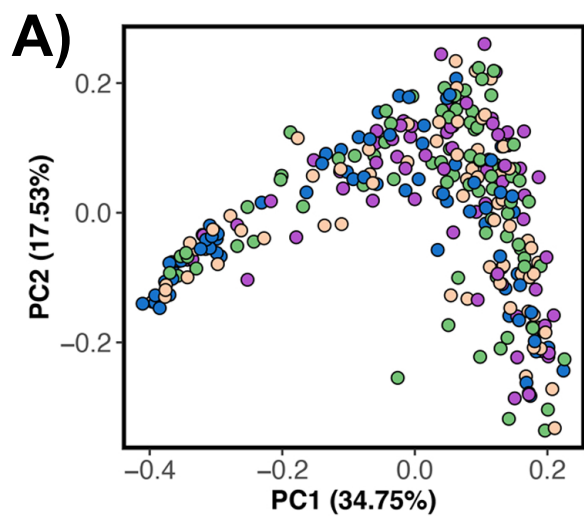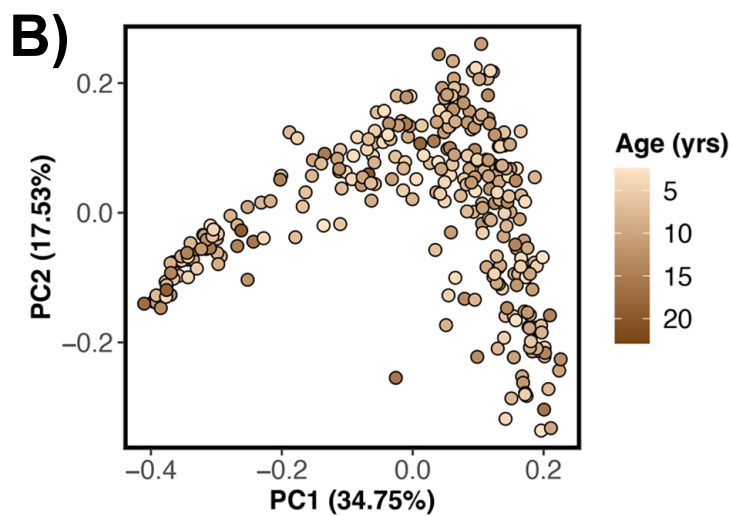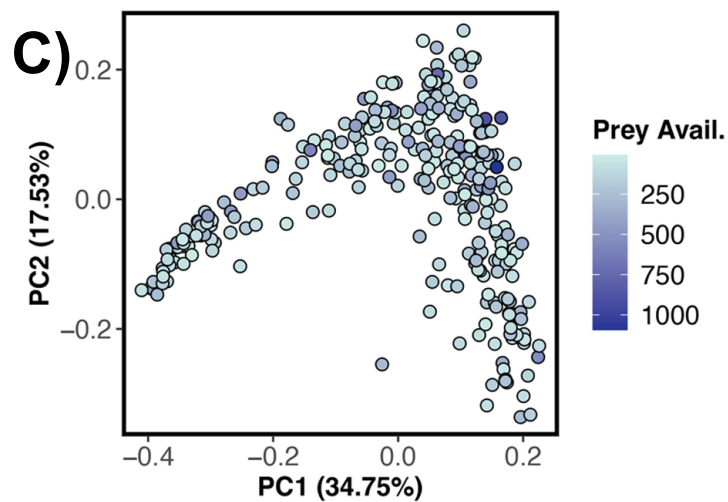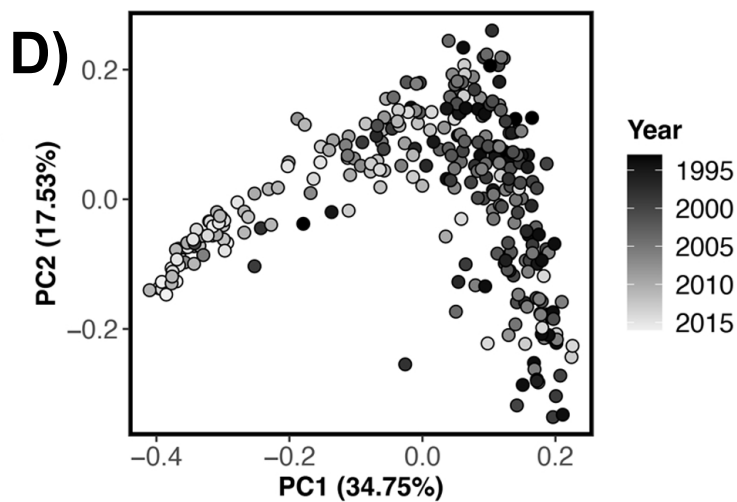

Supplement: FIG S1 [file msystems.00965-22-s0005.pdf]

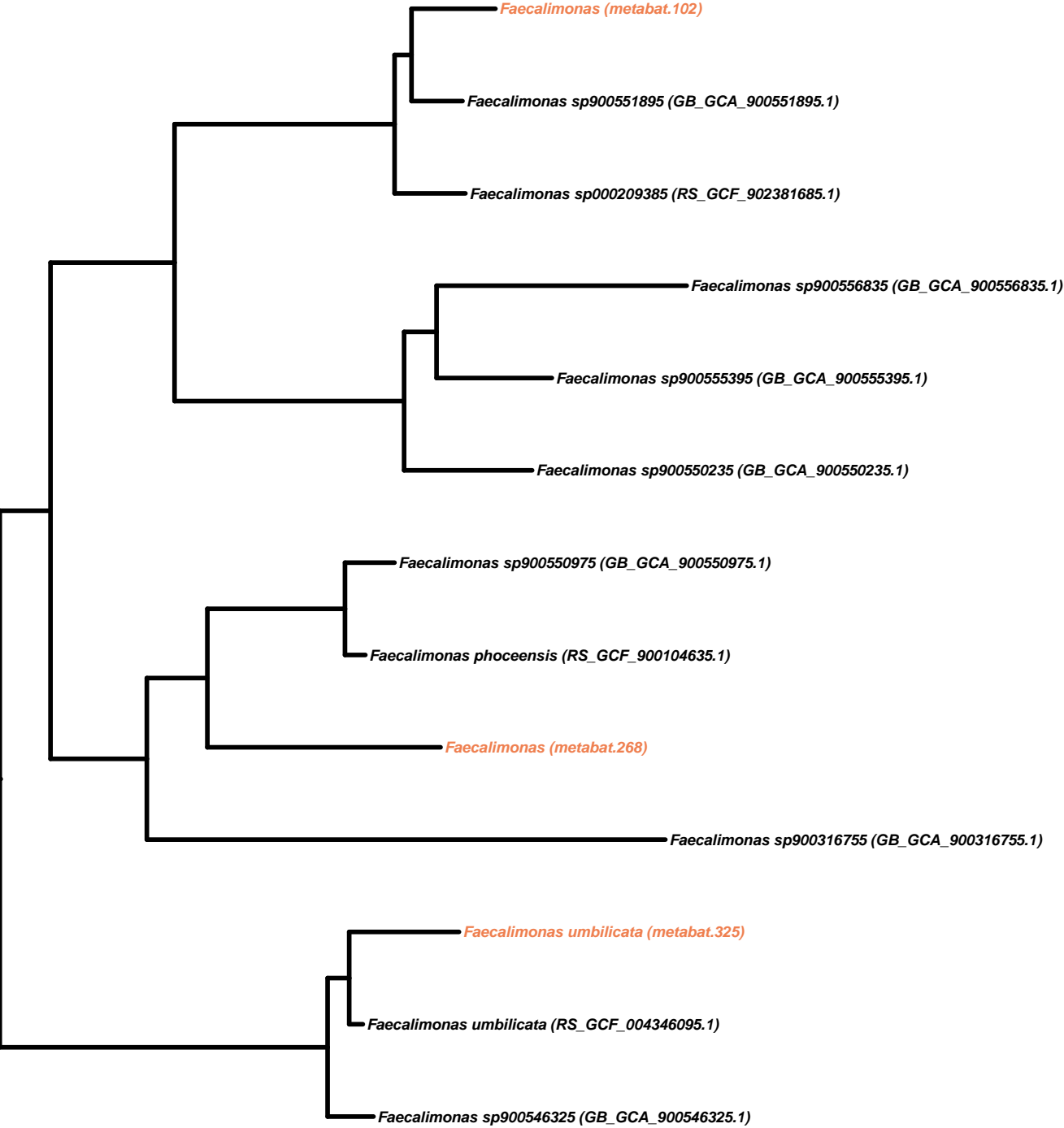

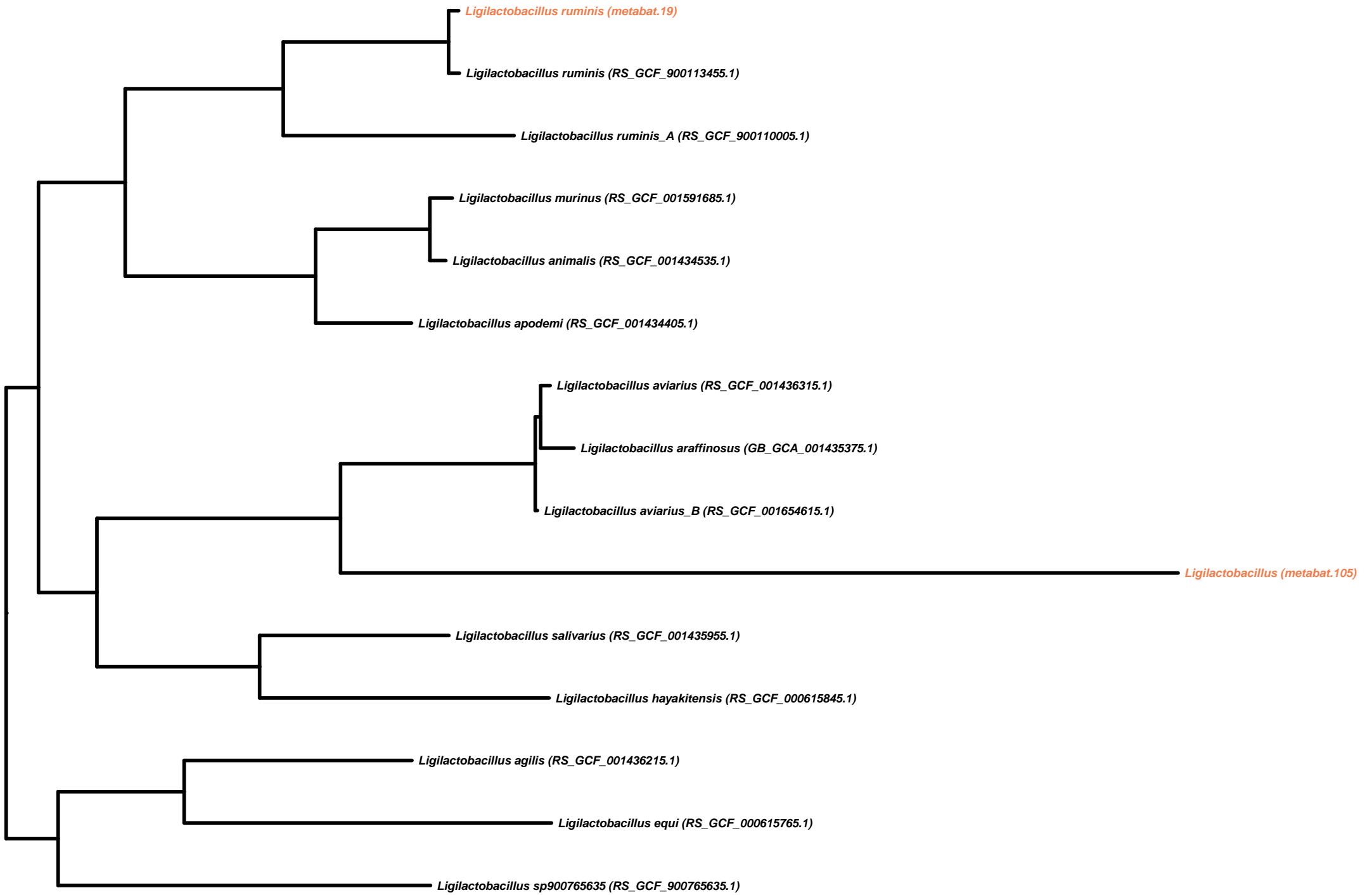

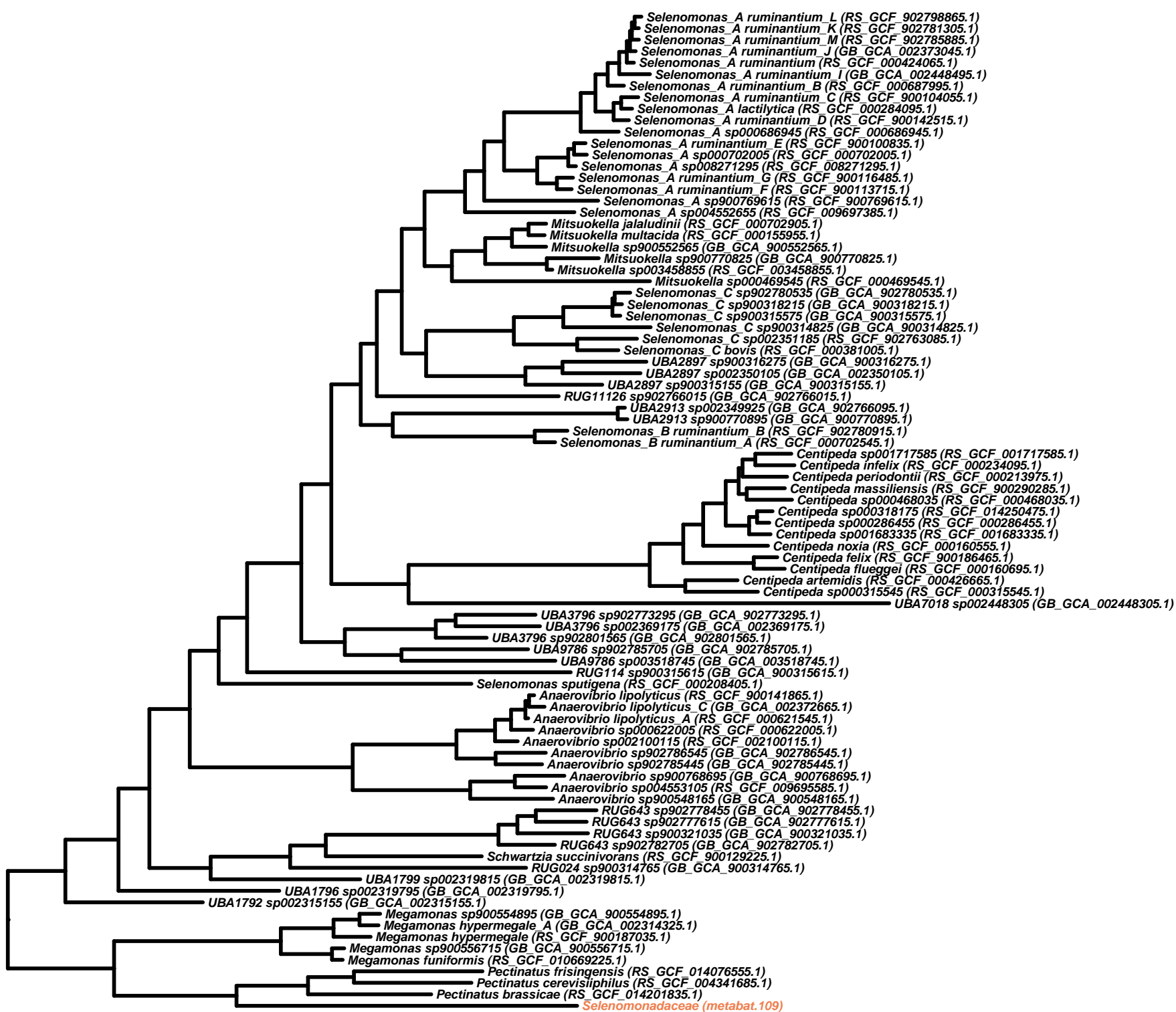

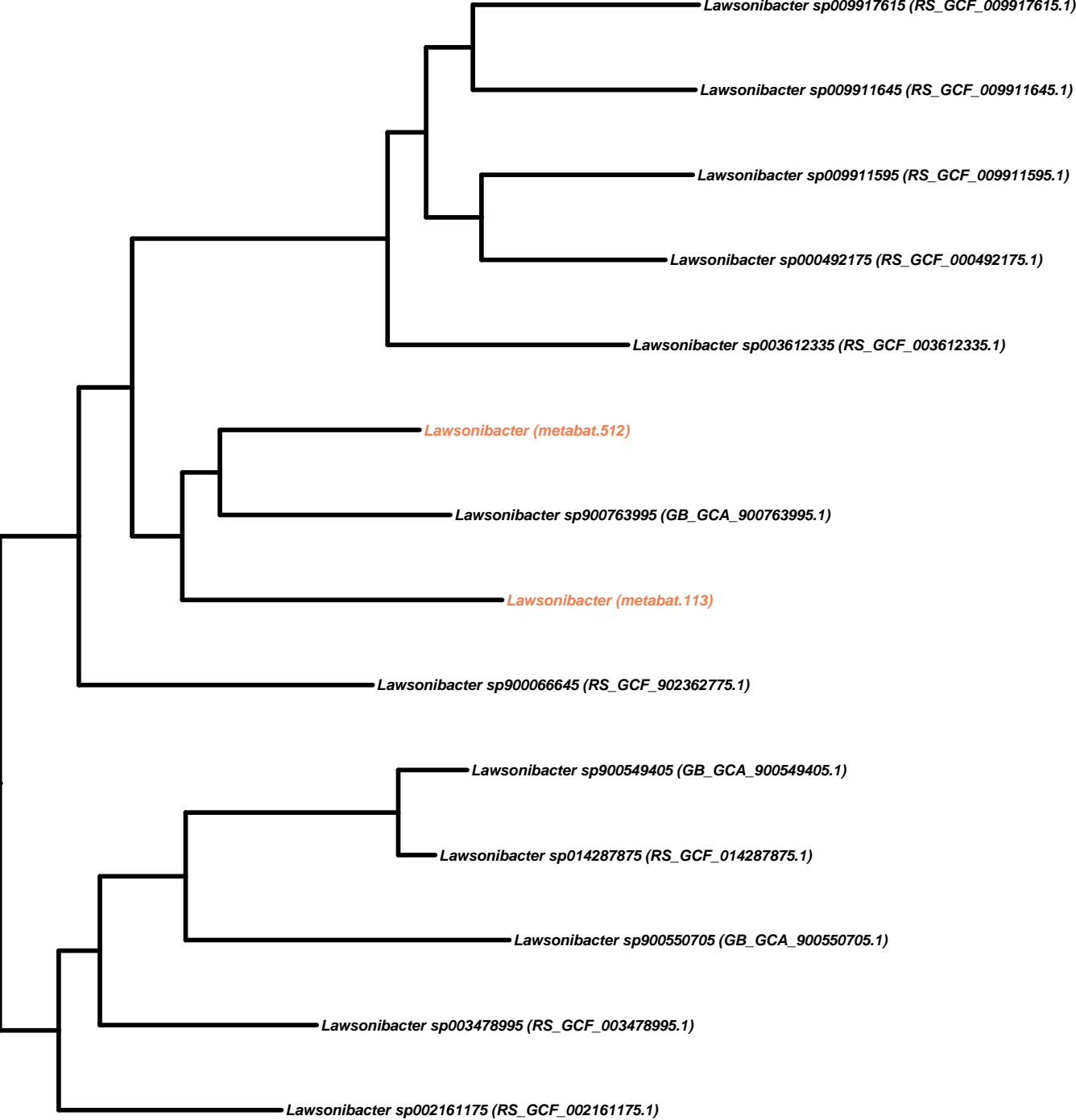

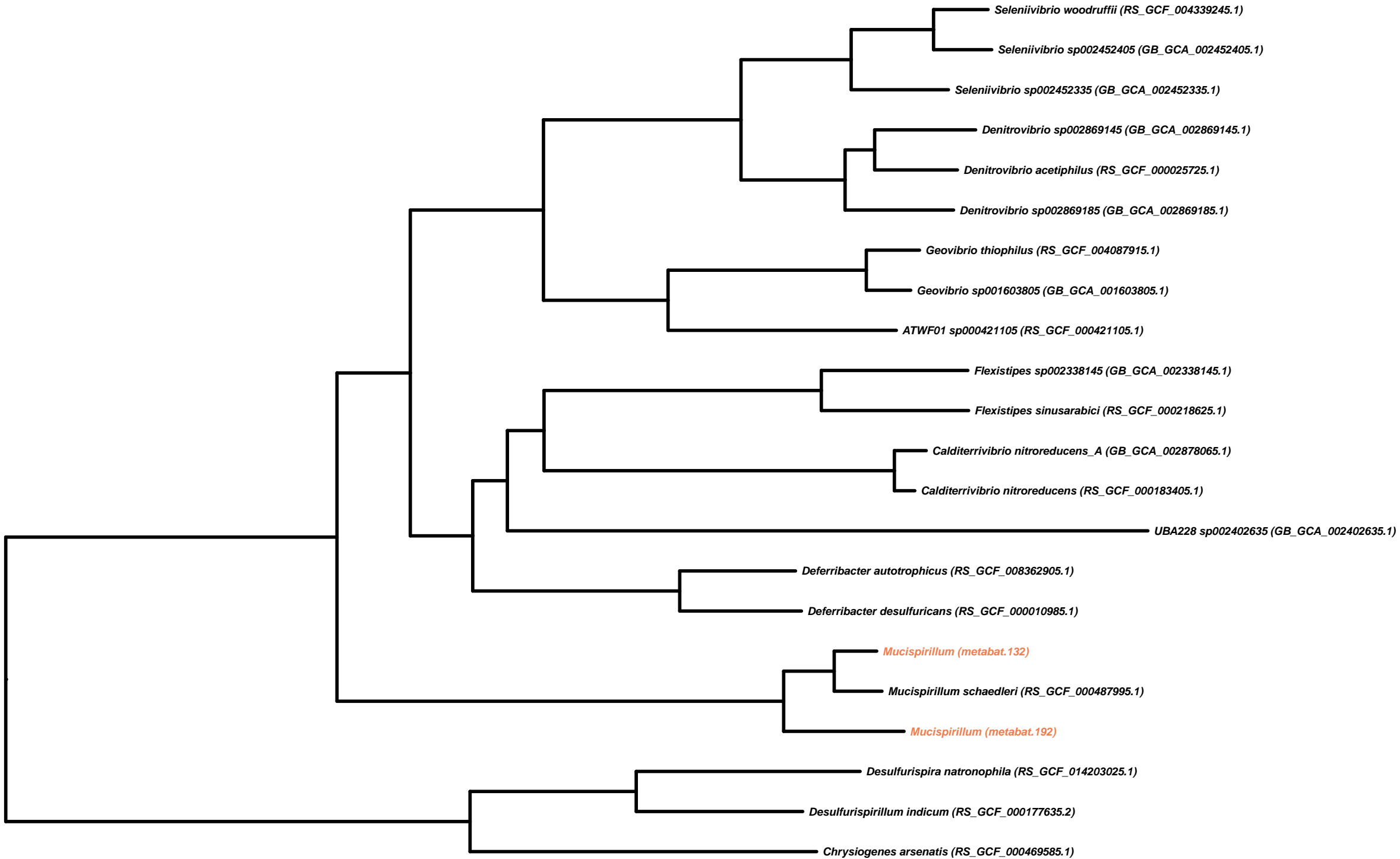

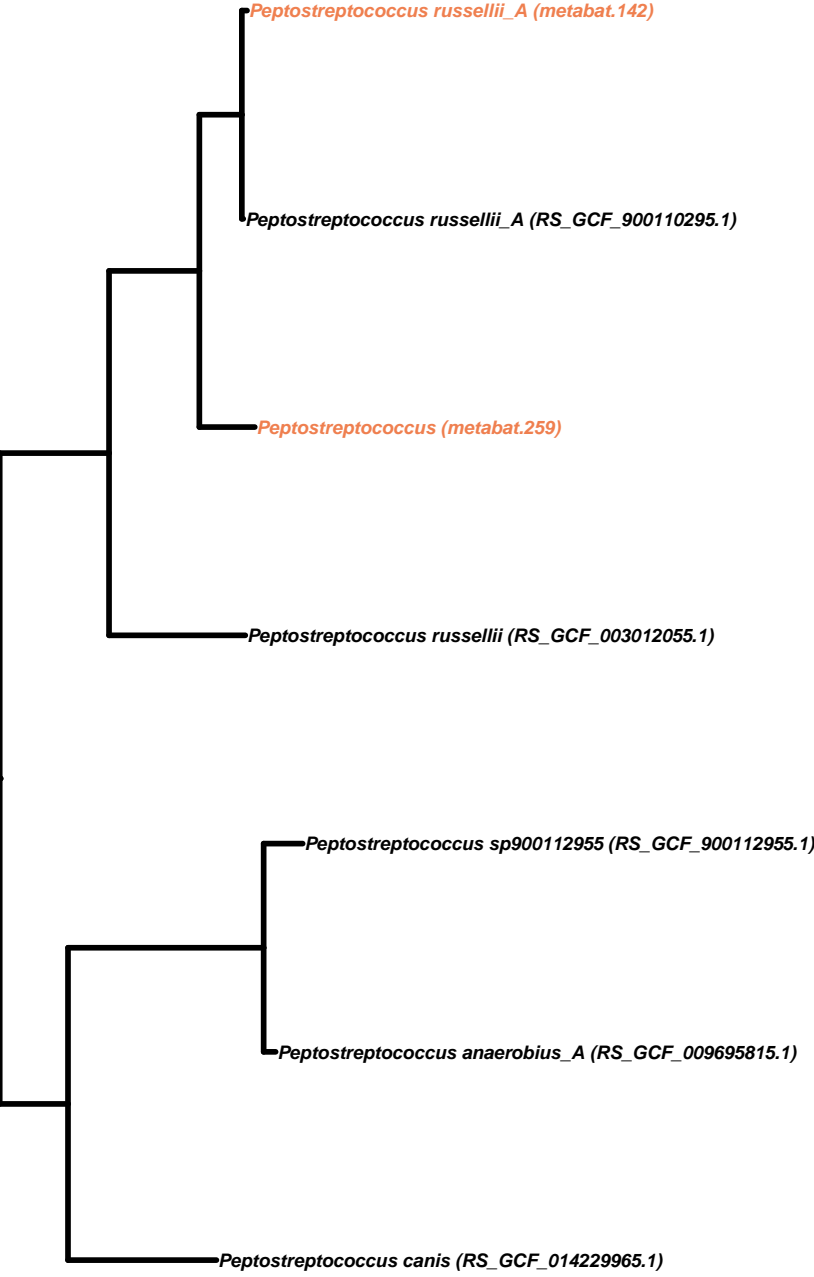

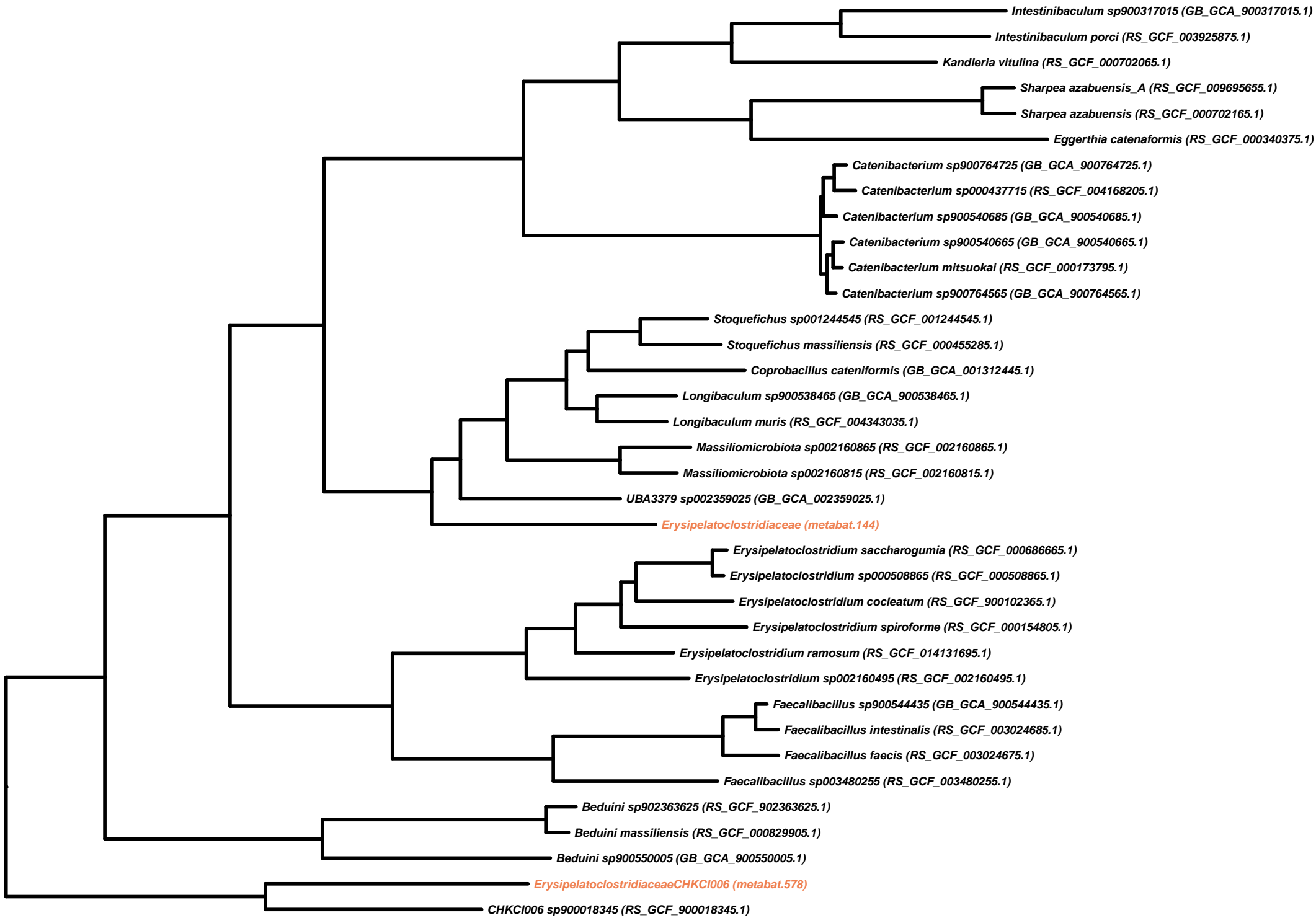

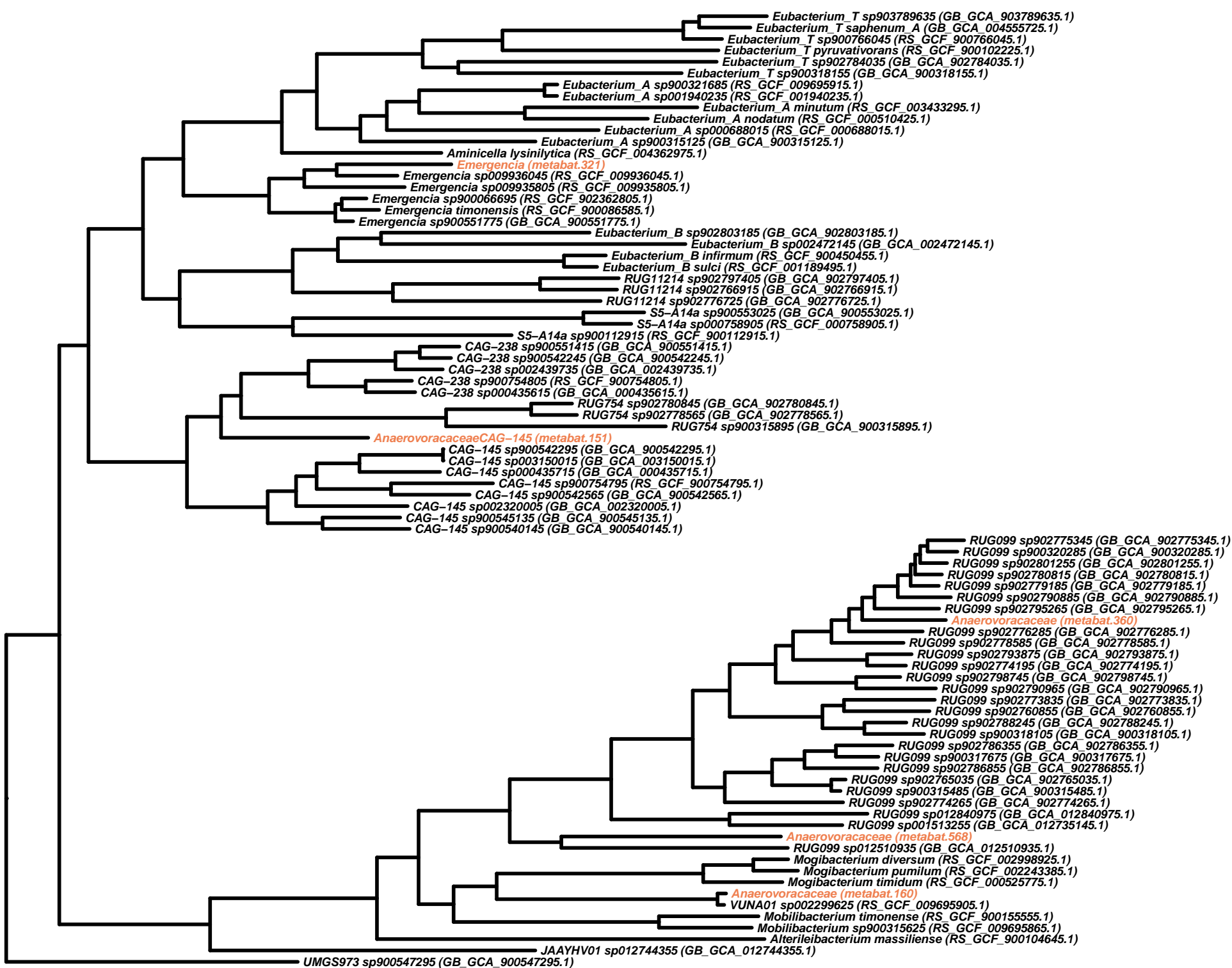

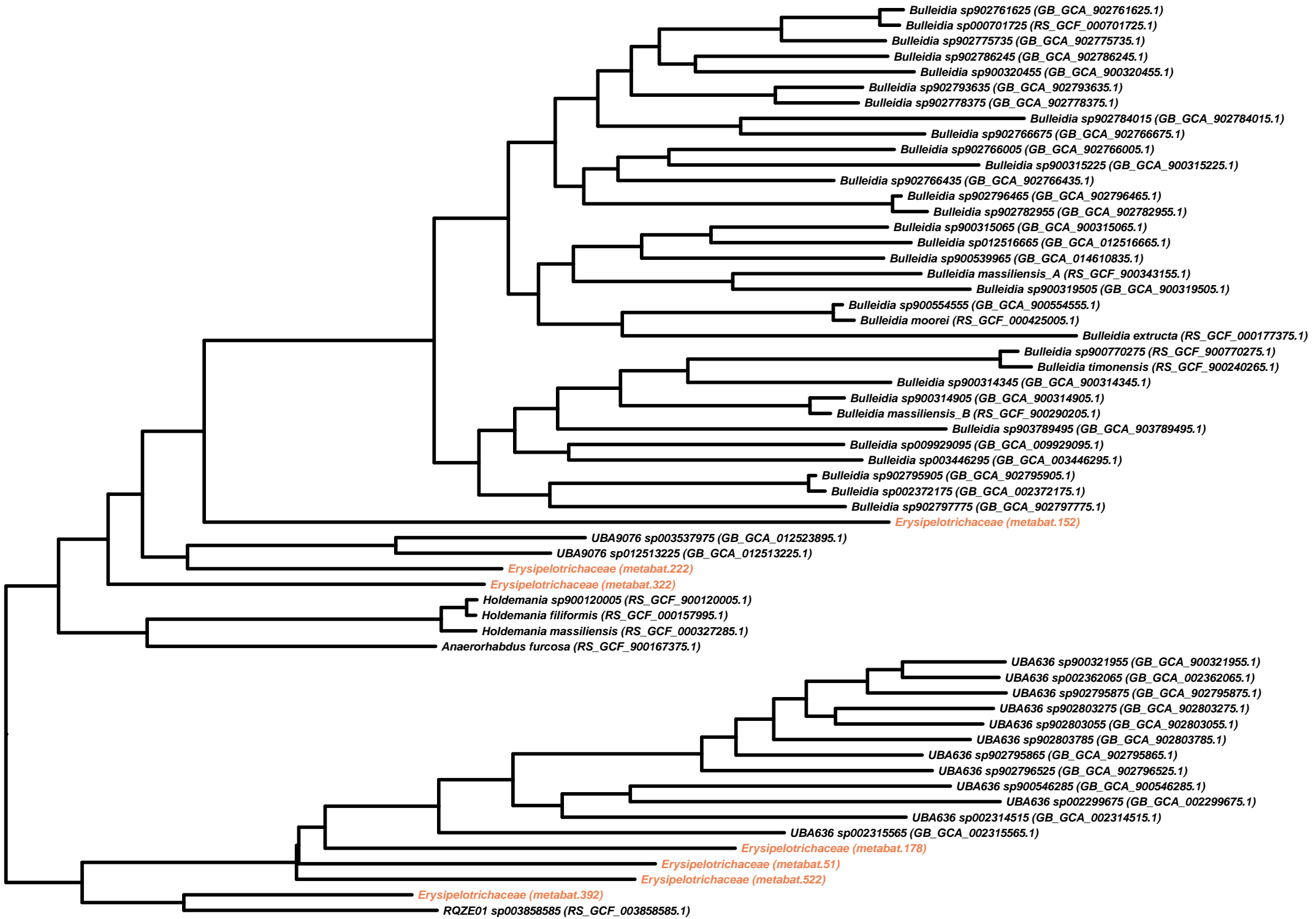

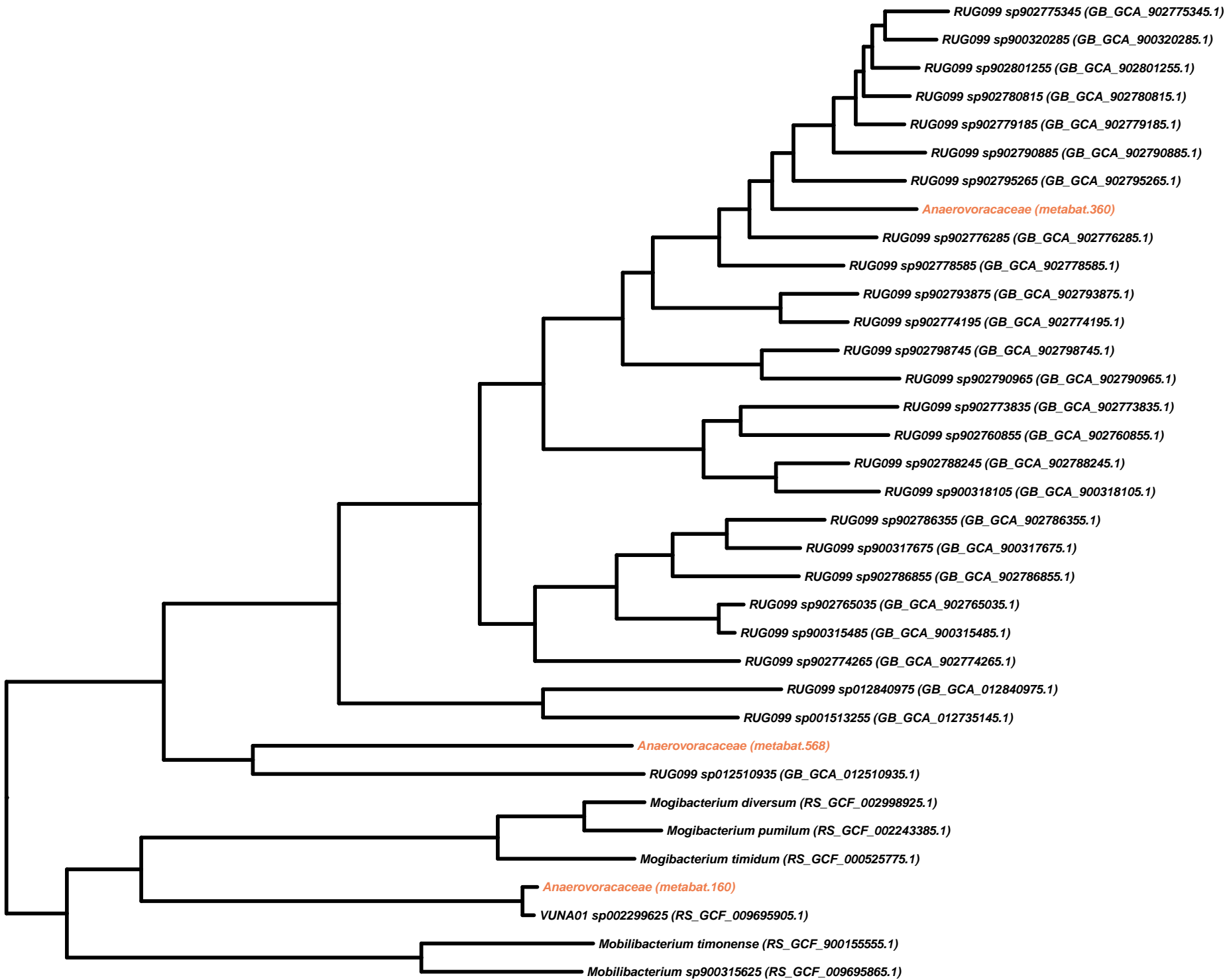

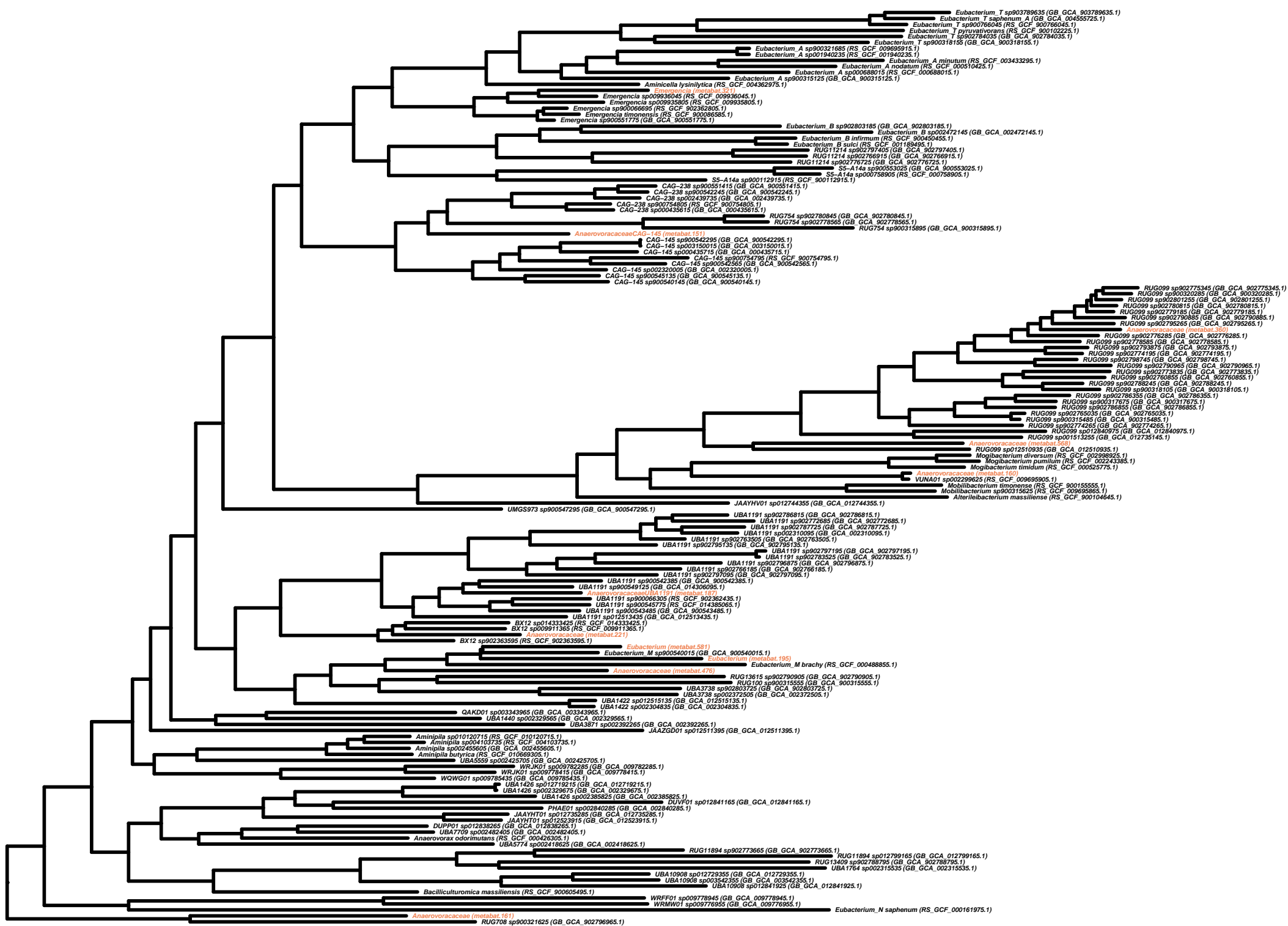

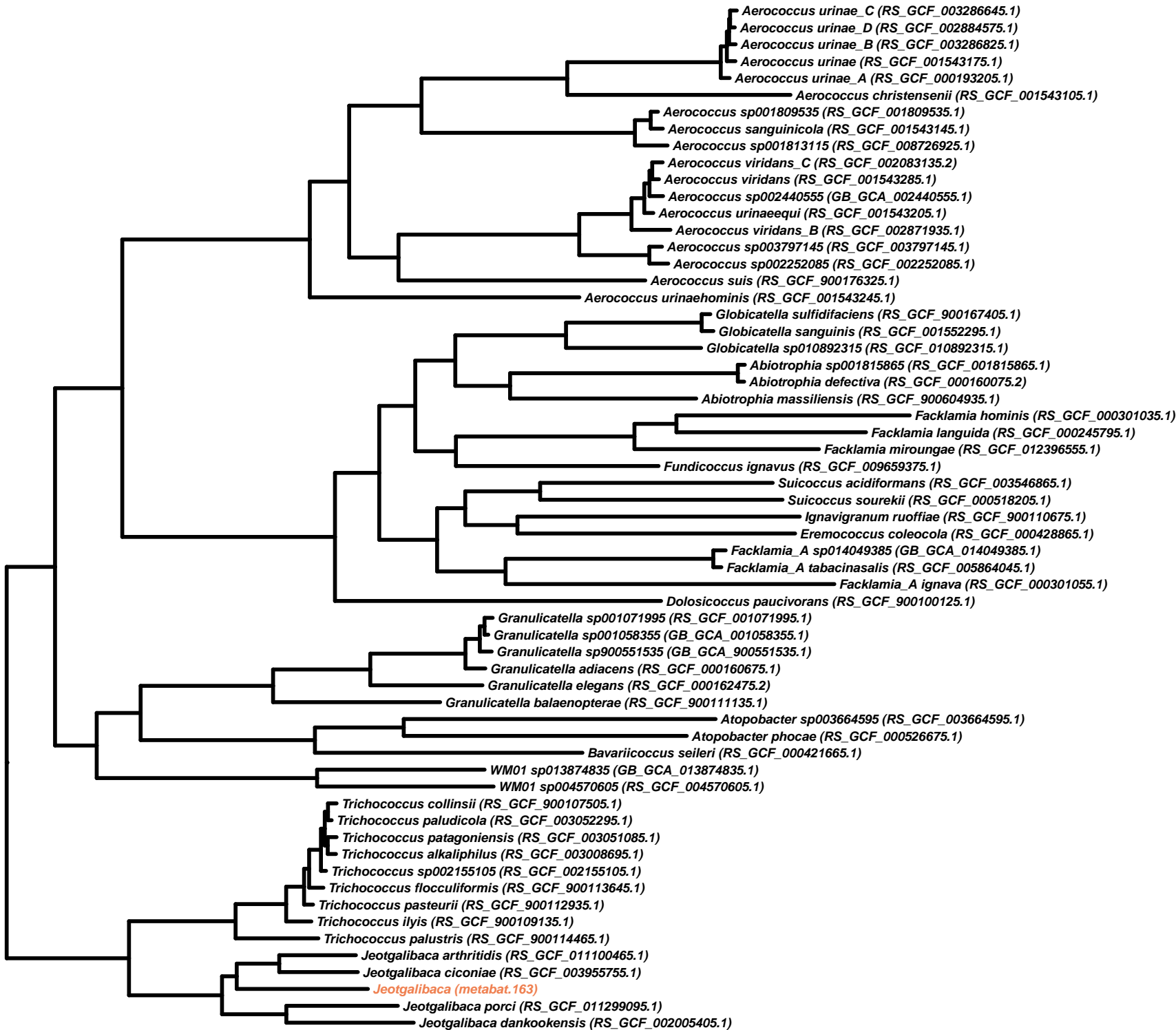

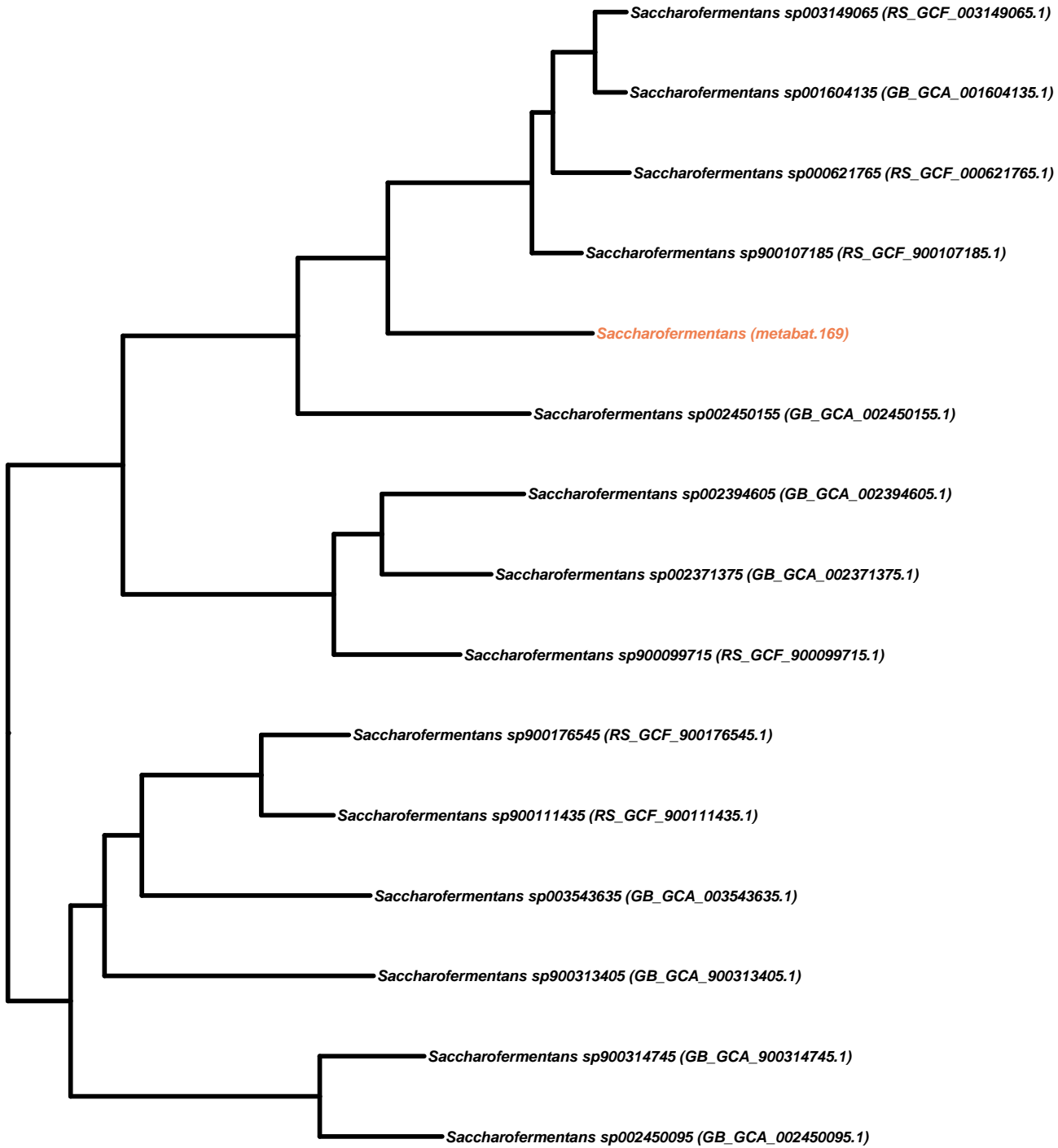

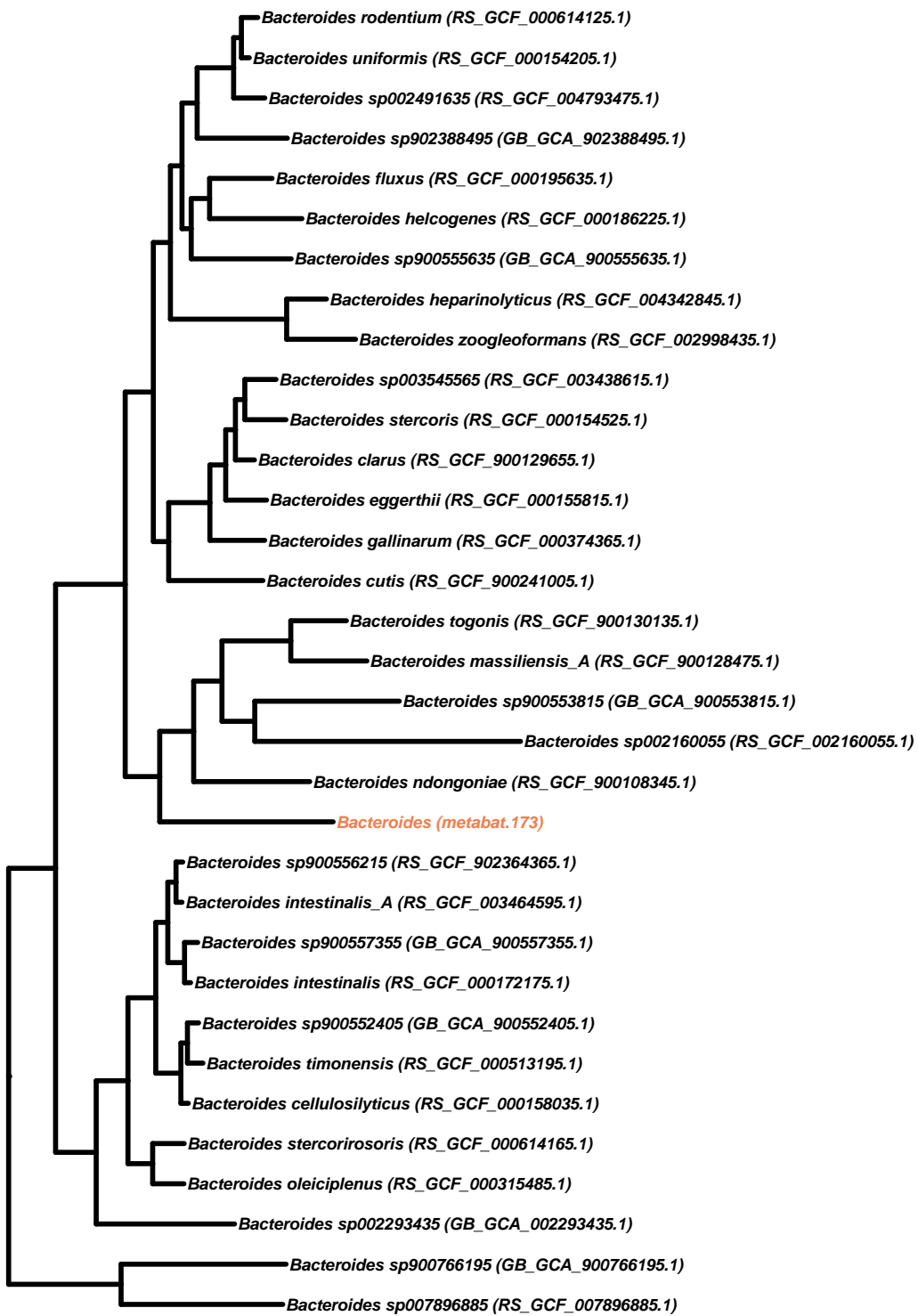

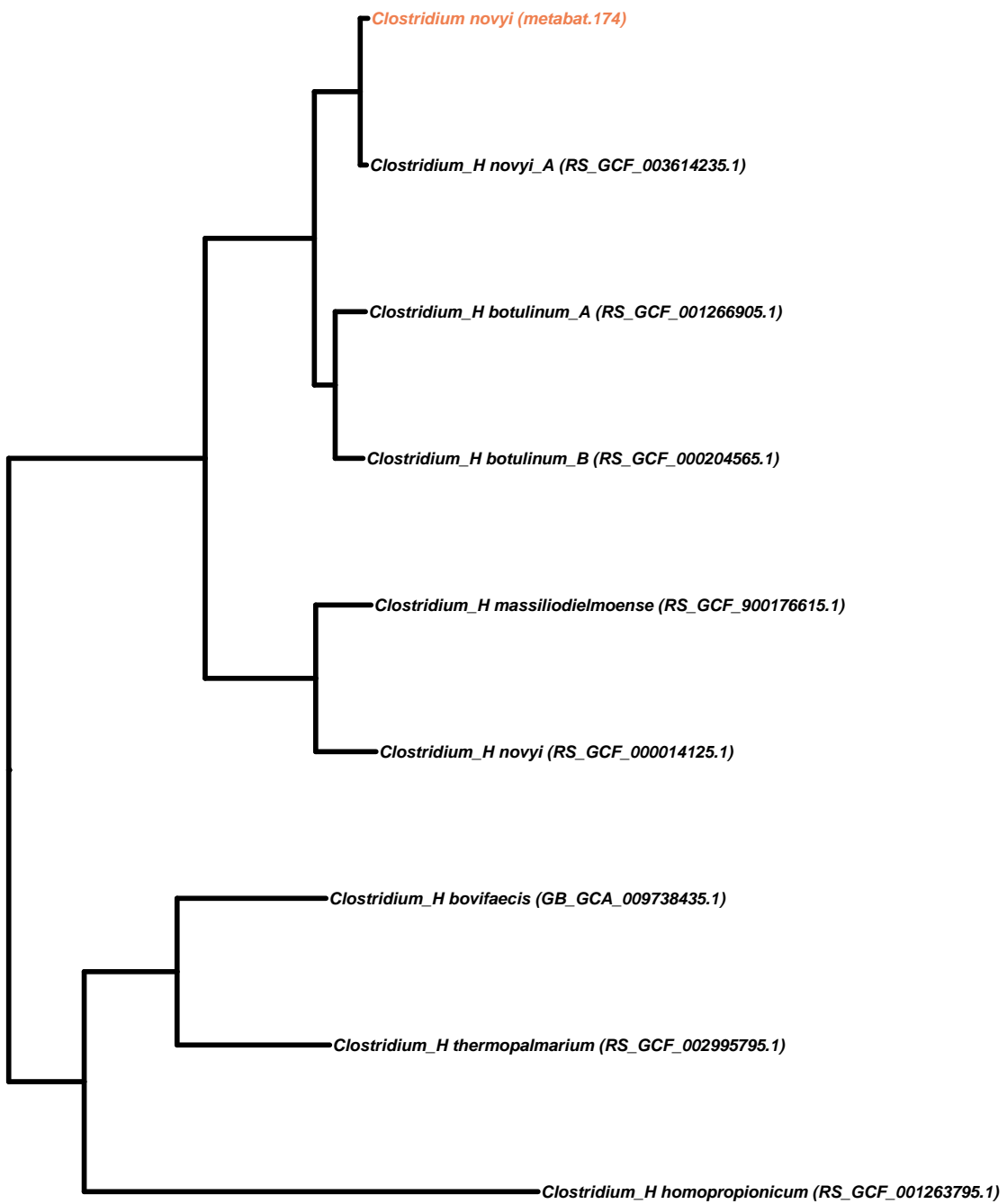

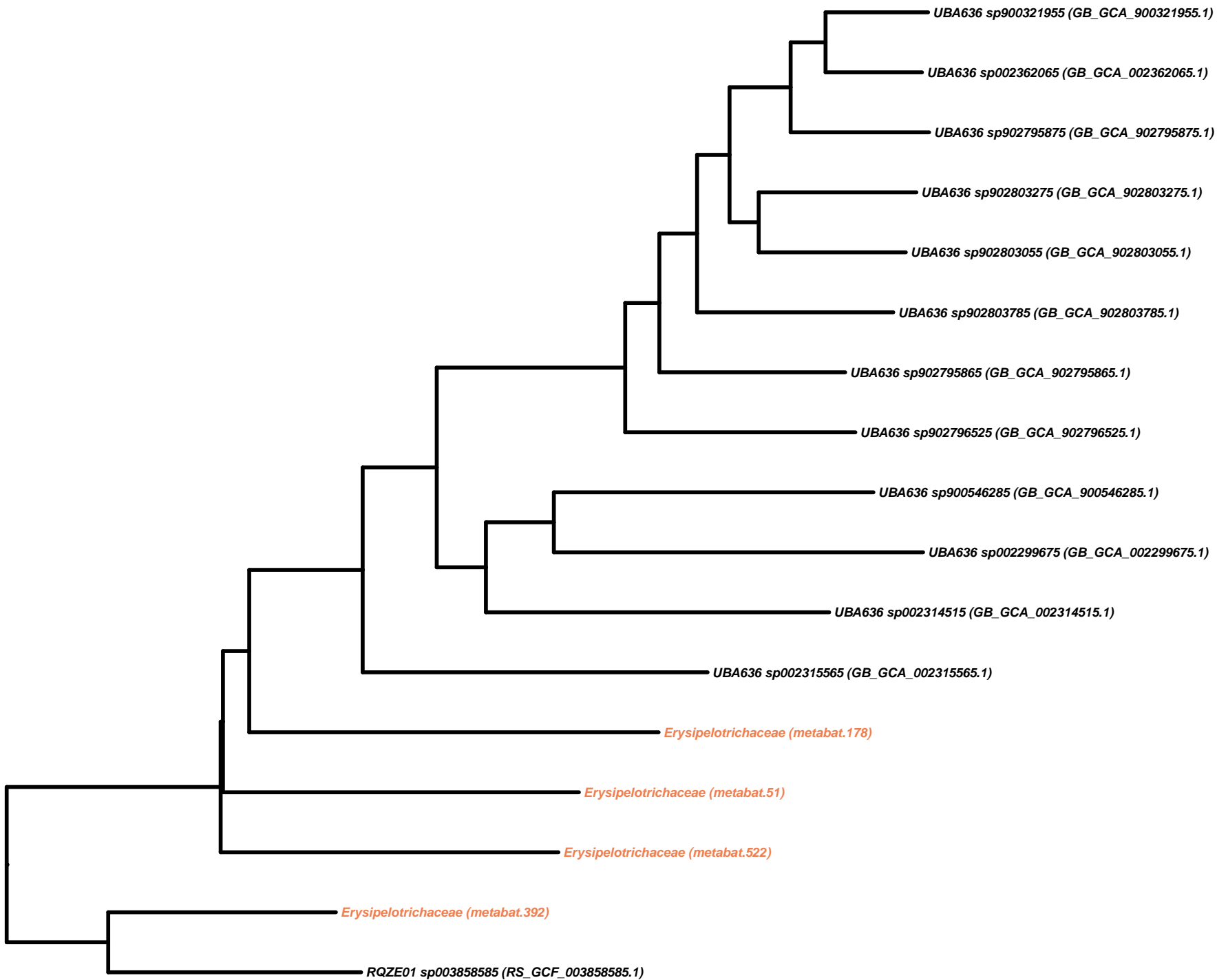

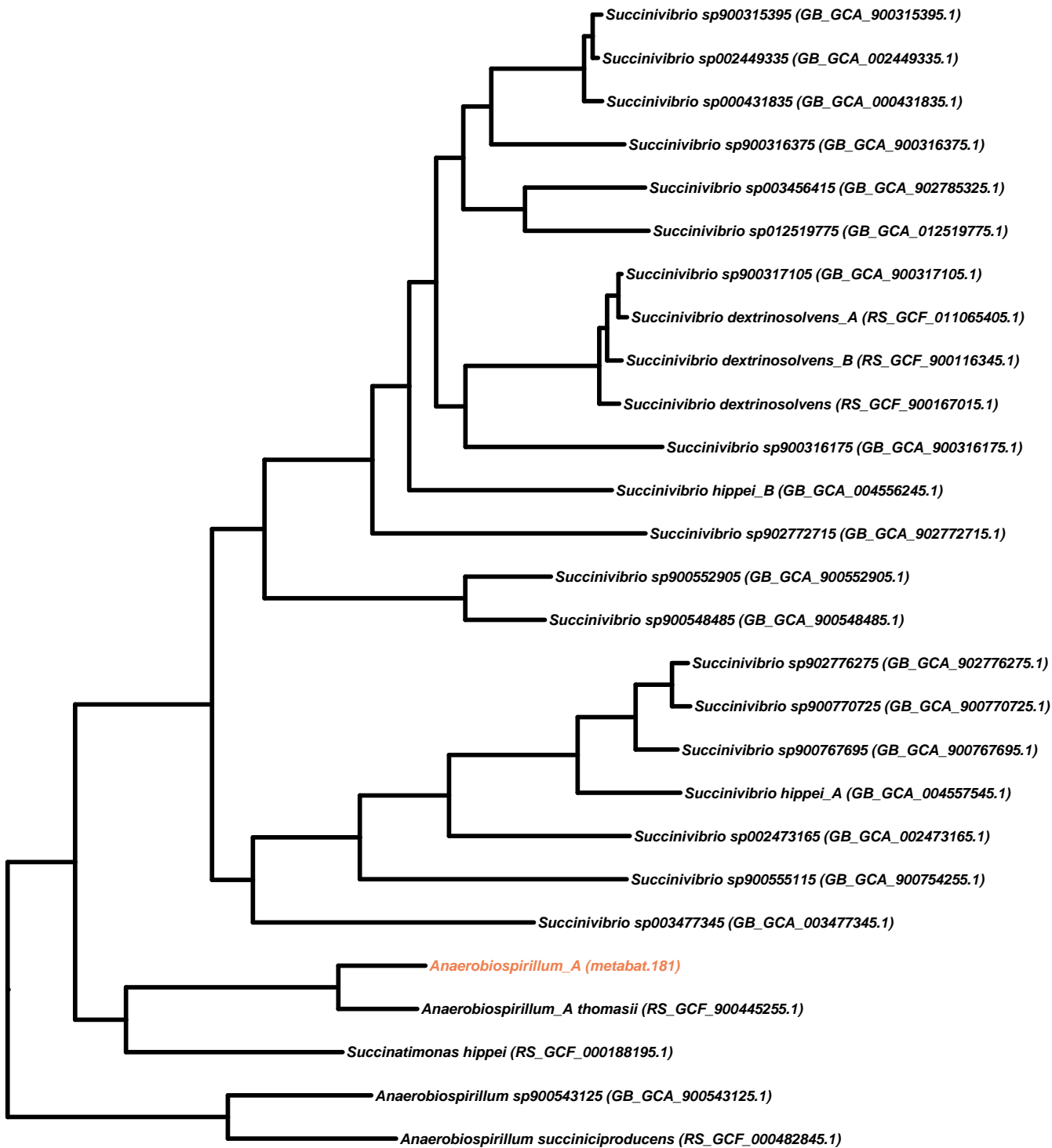

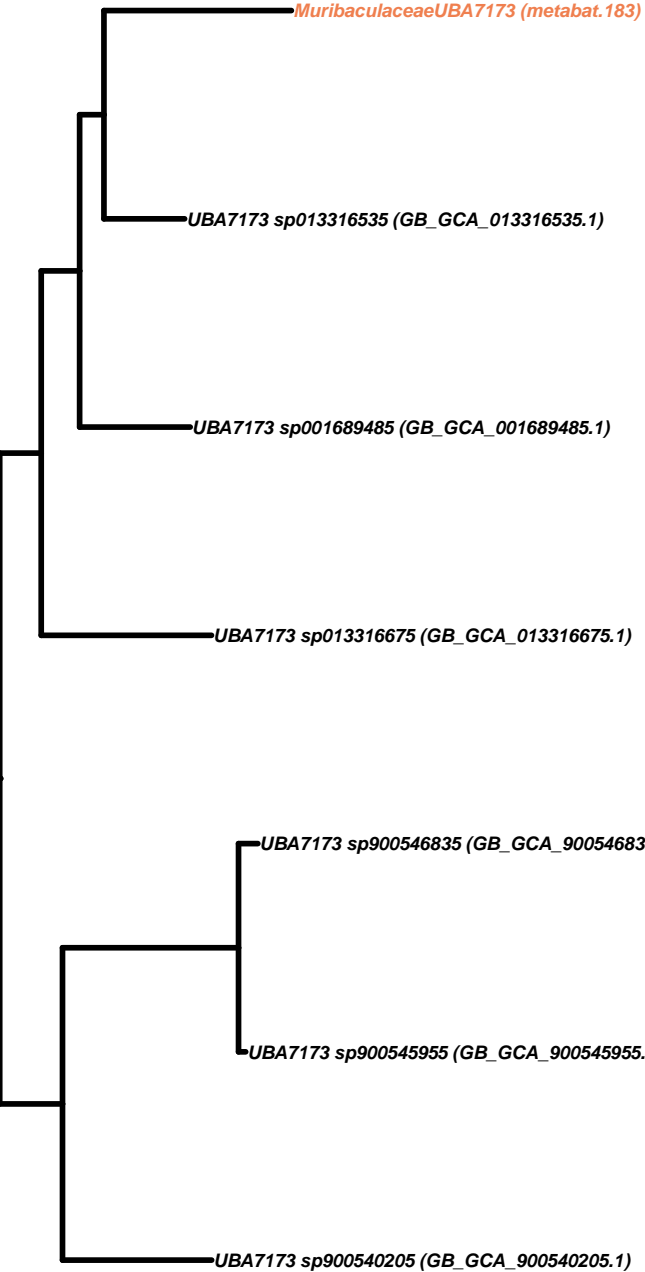

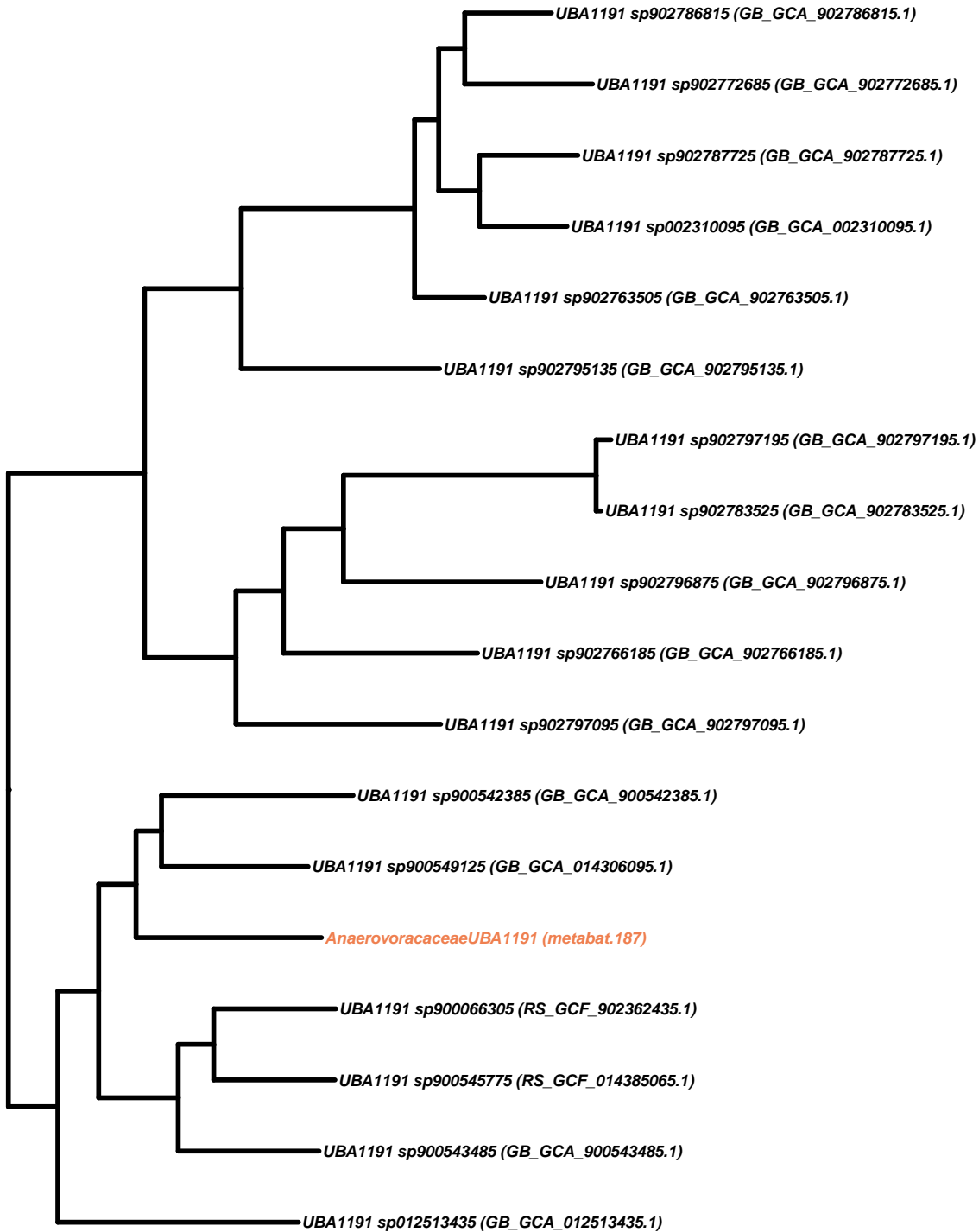

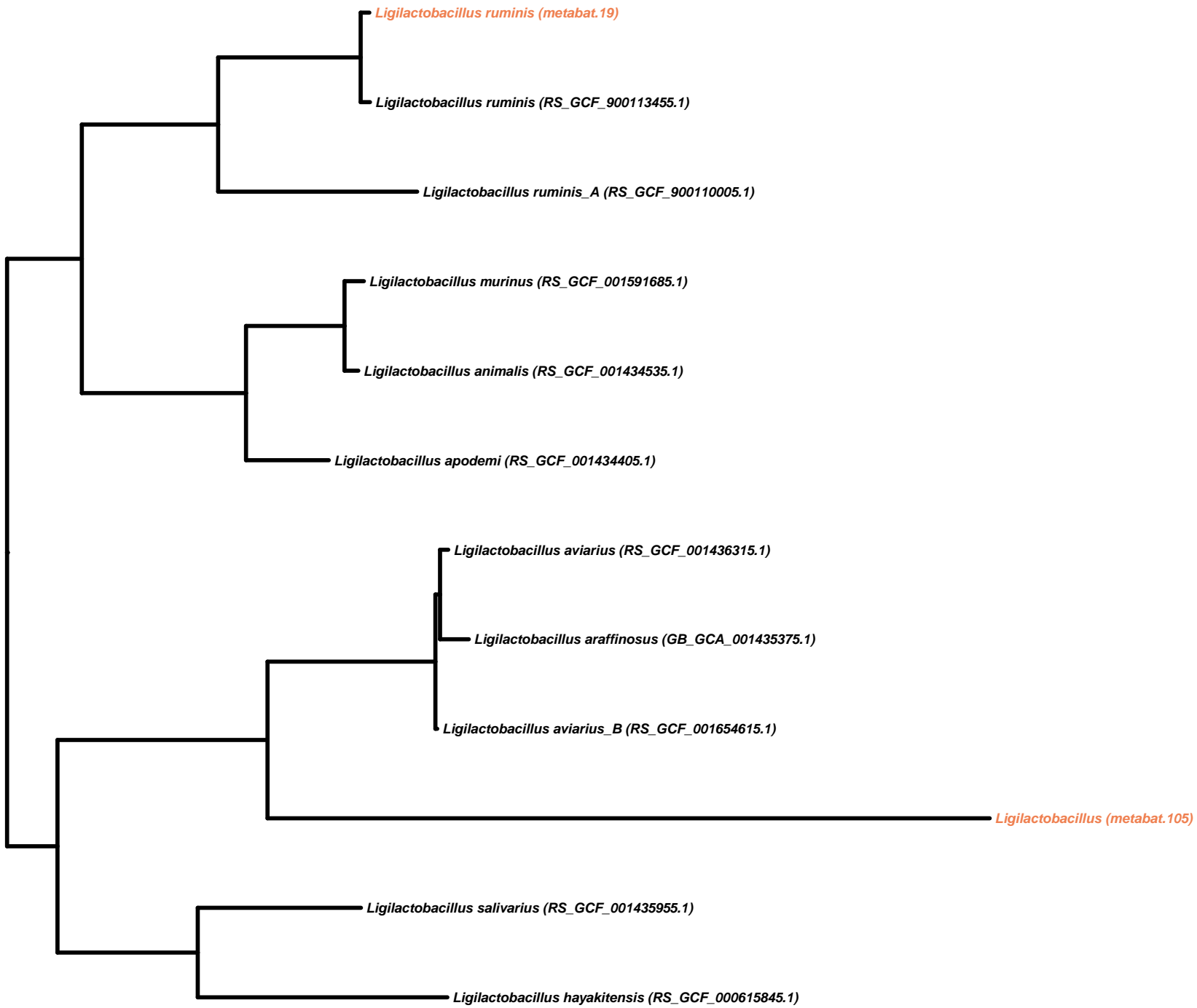

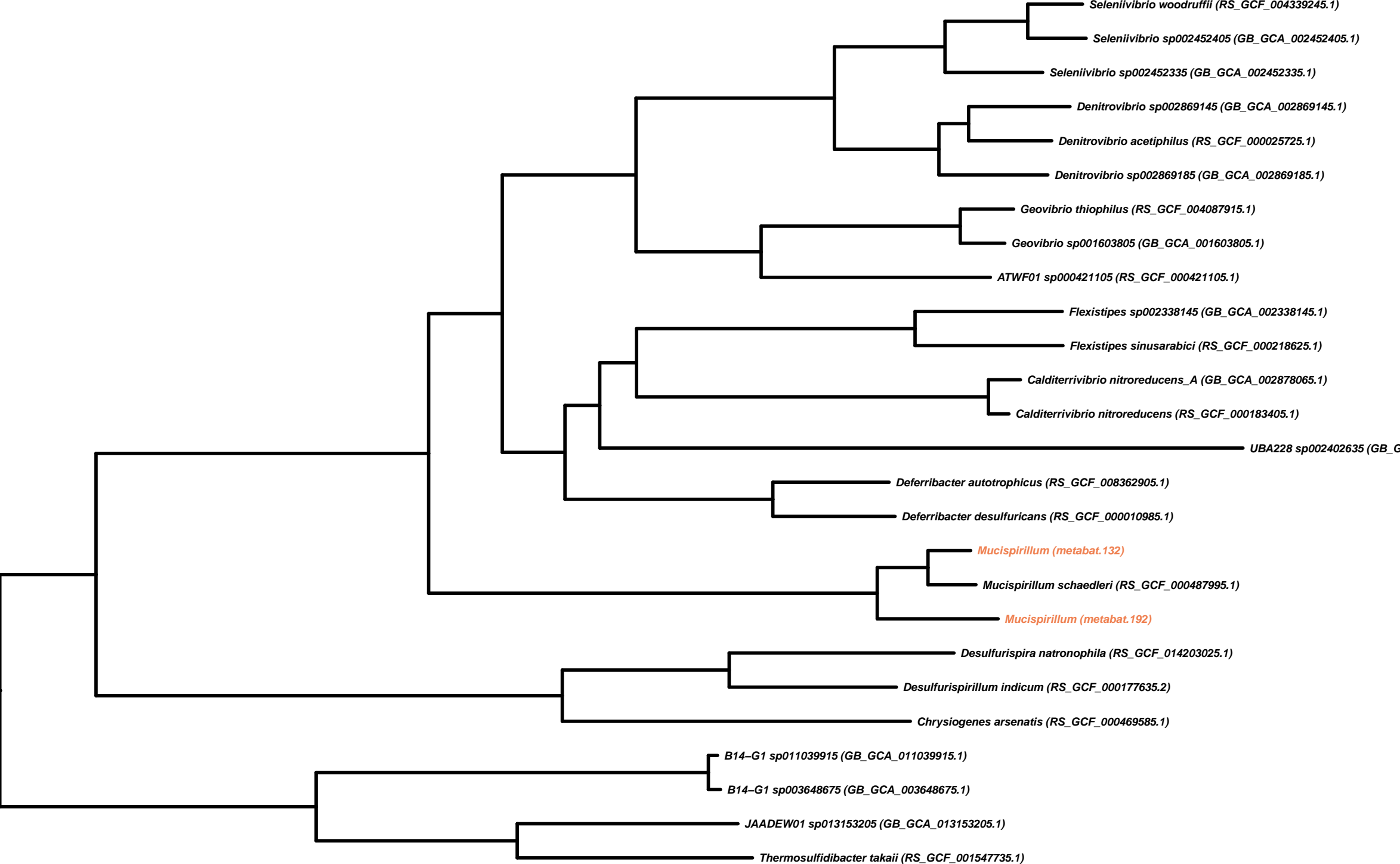

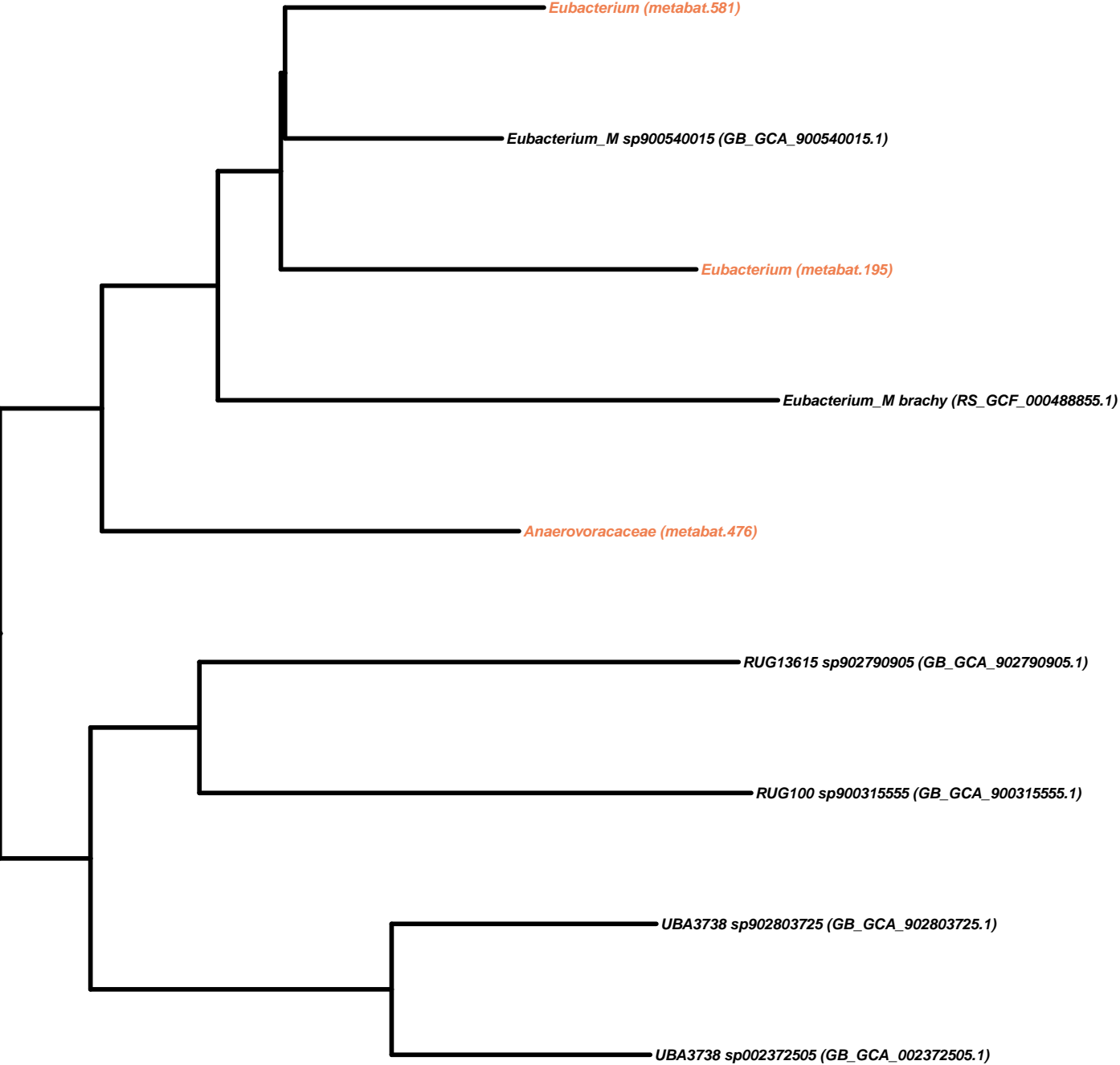

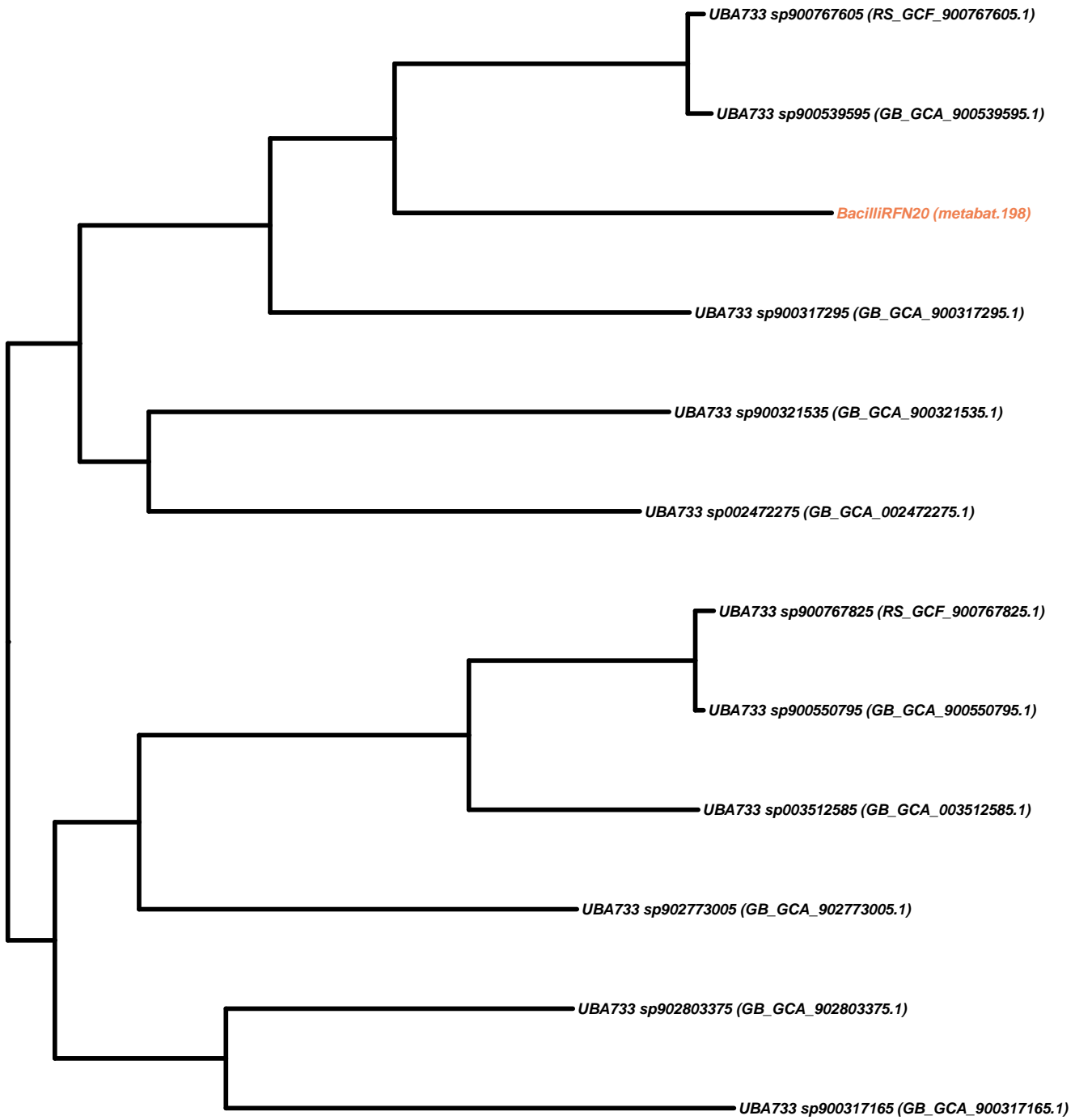

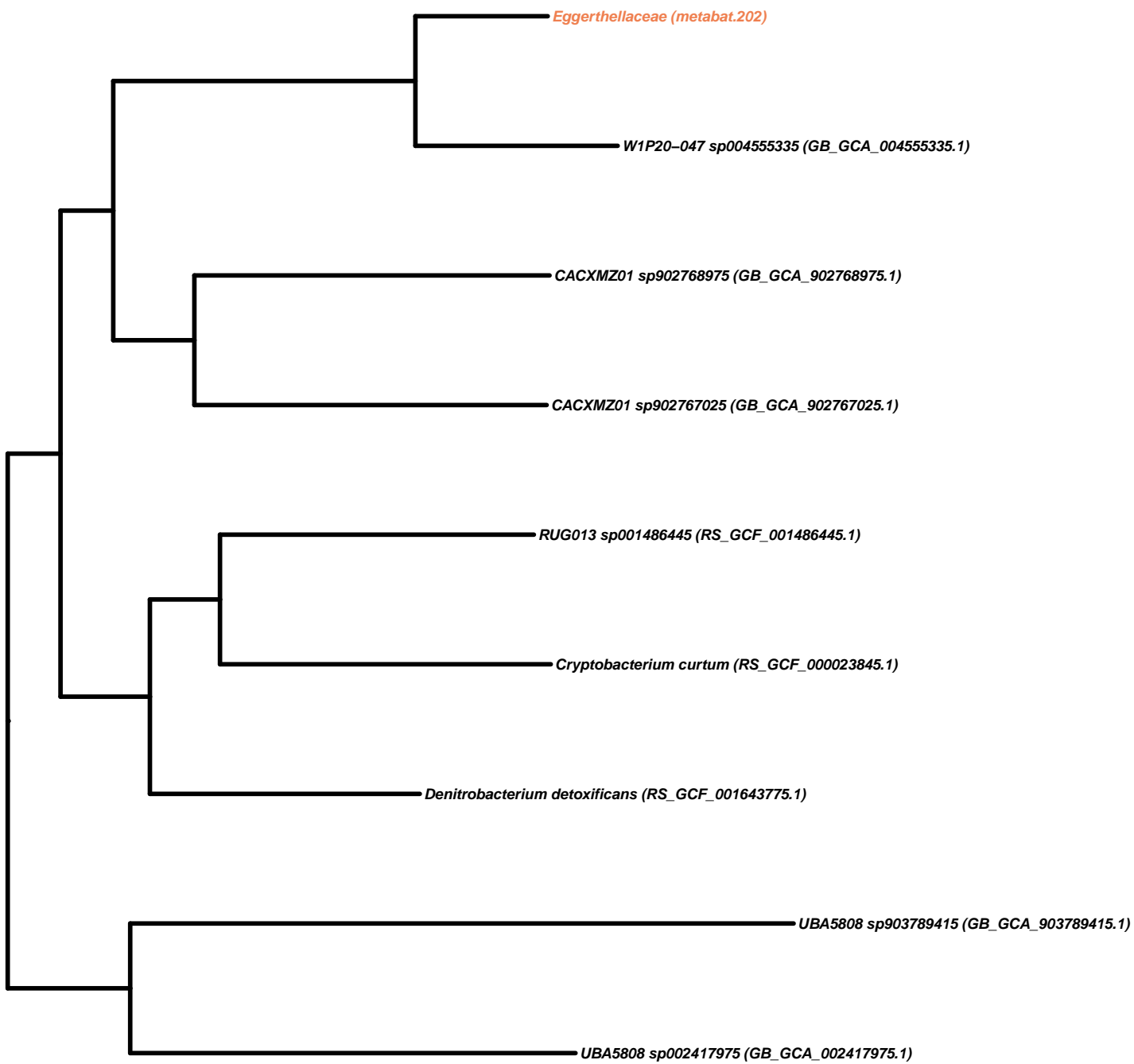

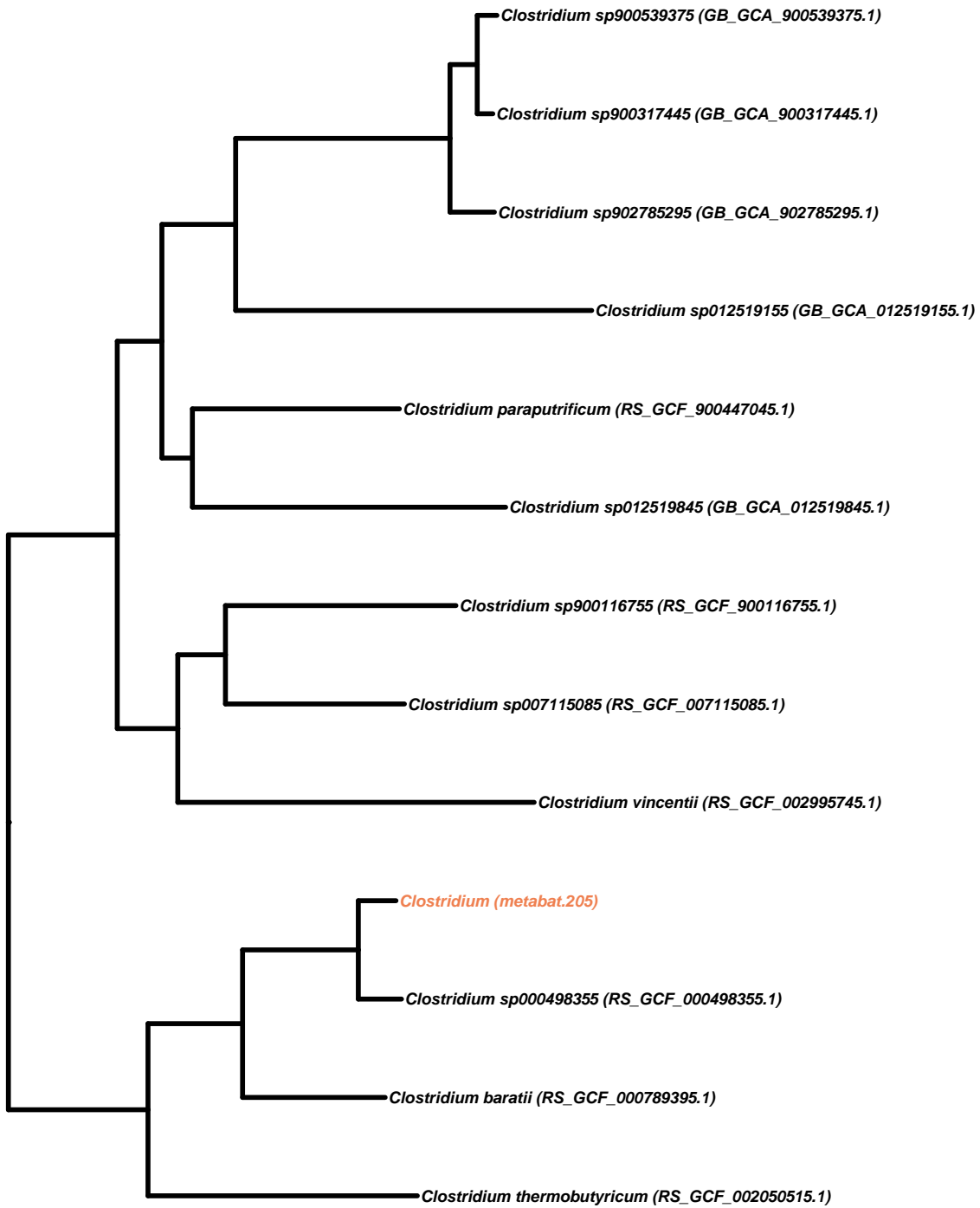

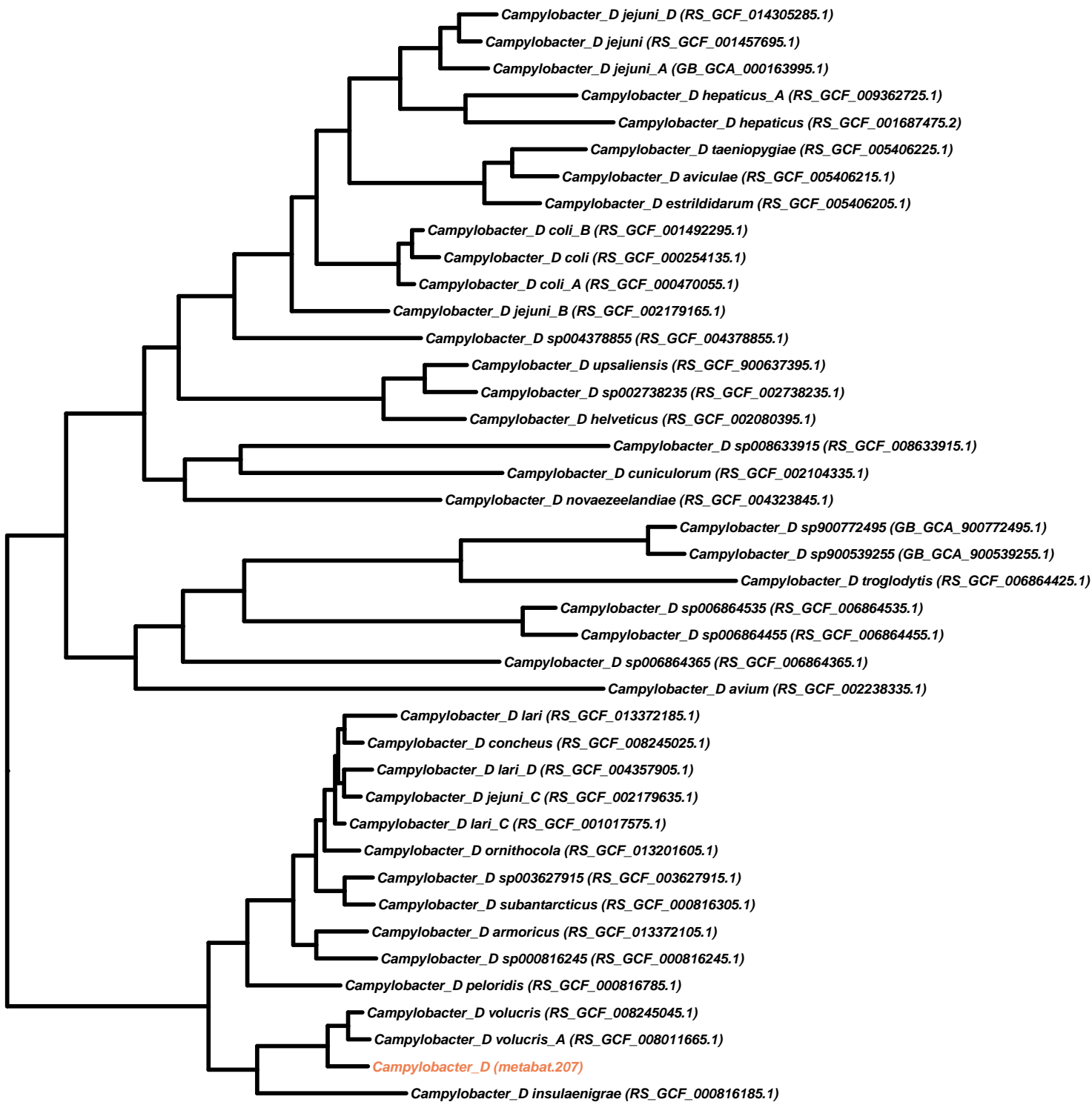

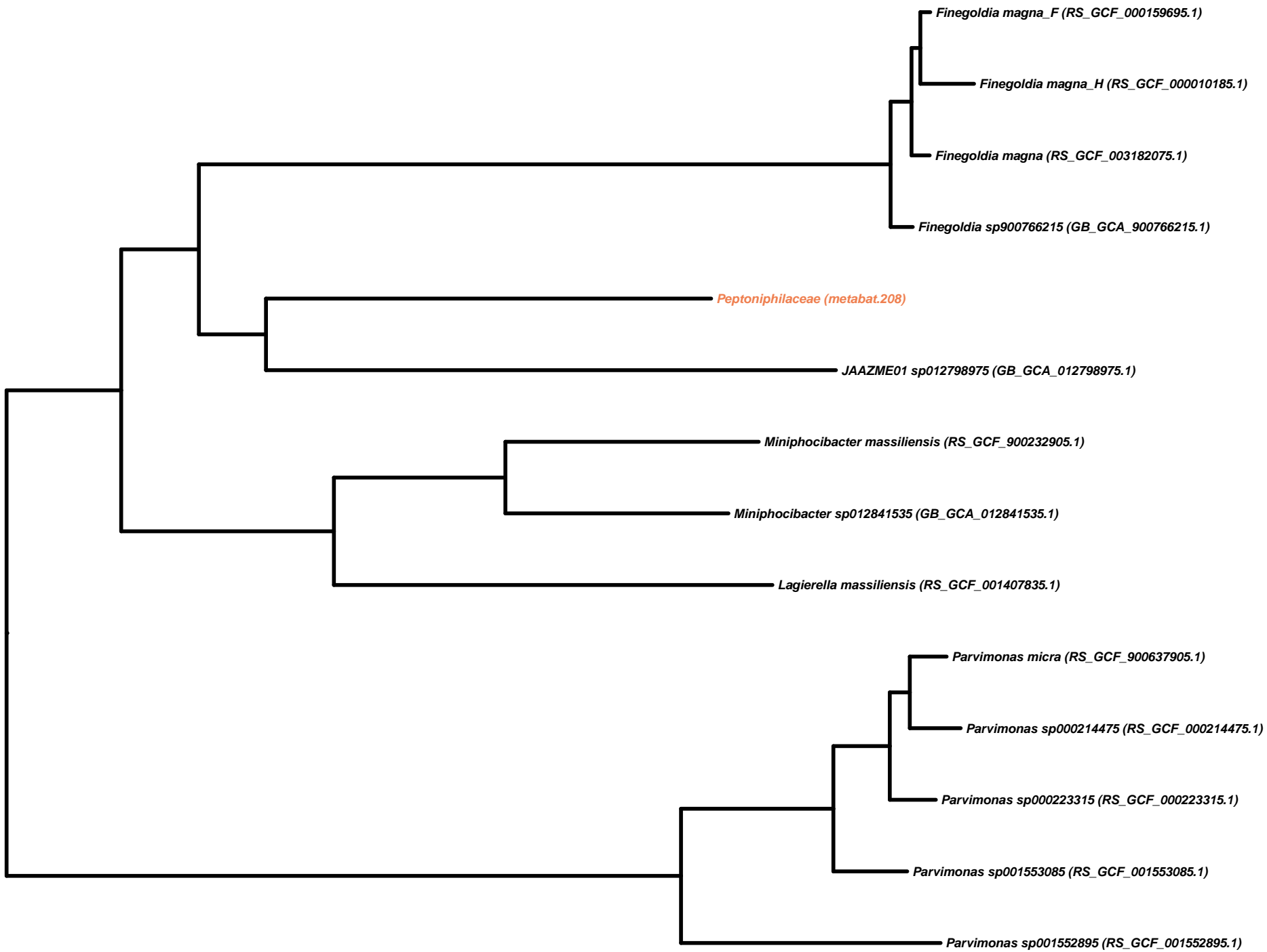

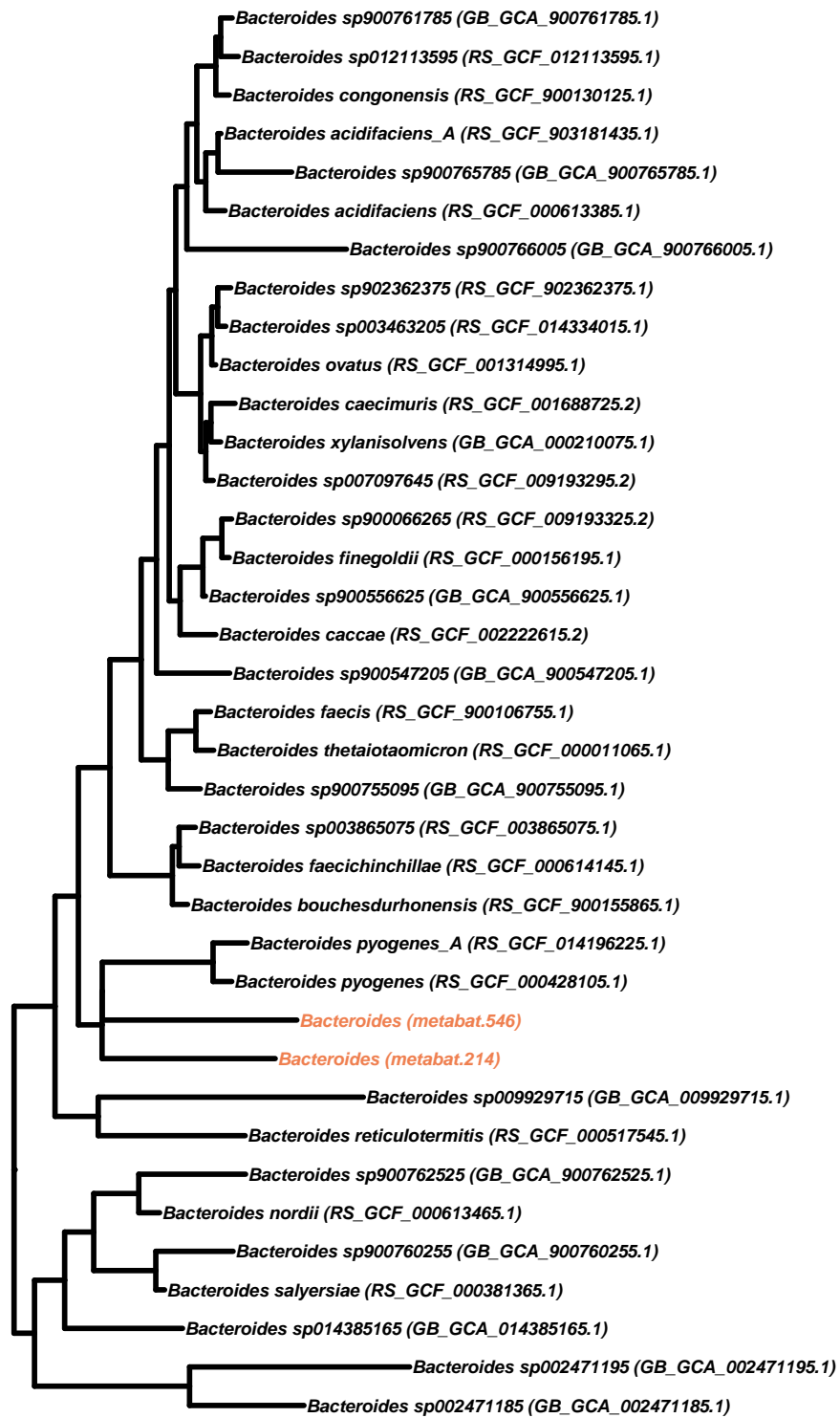

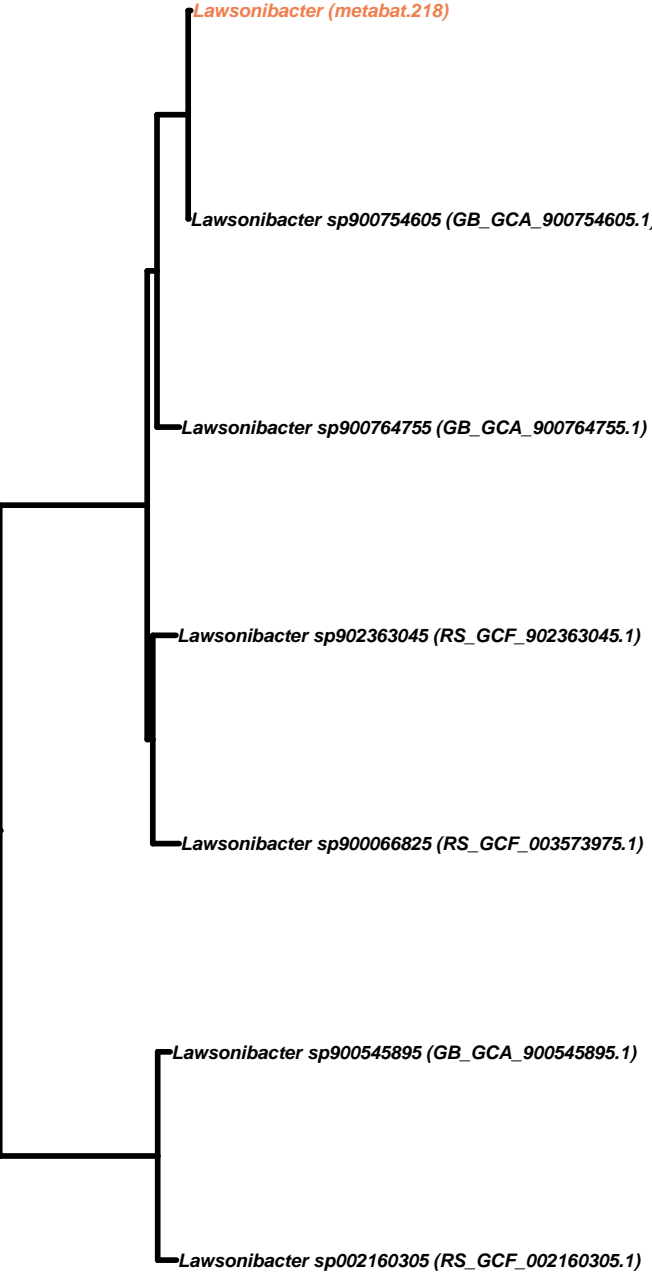

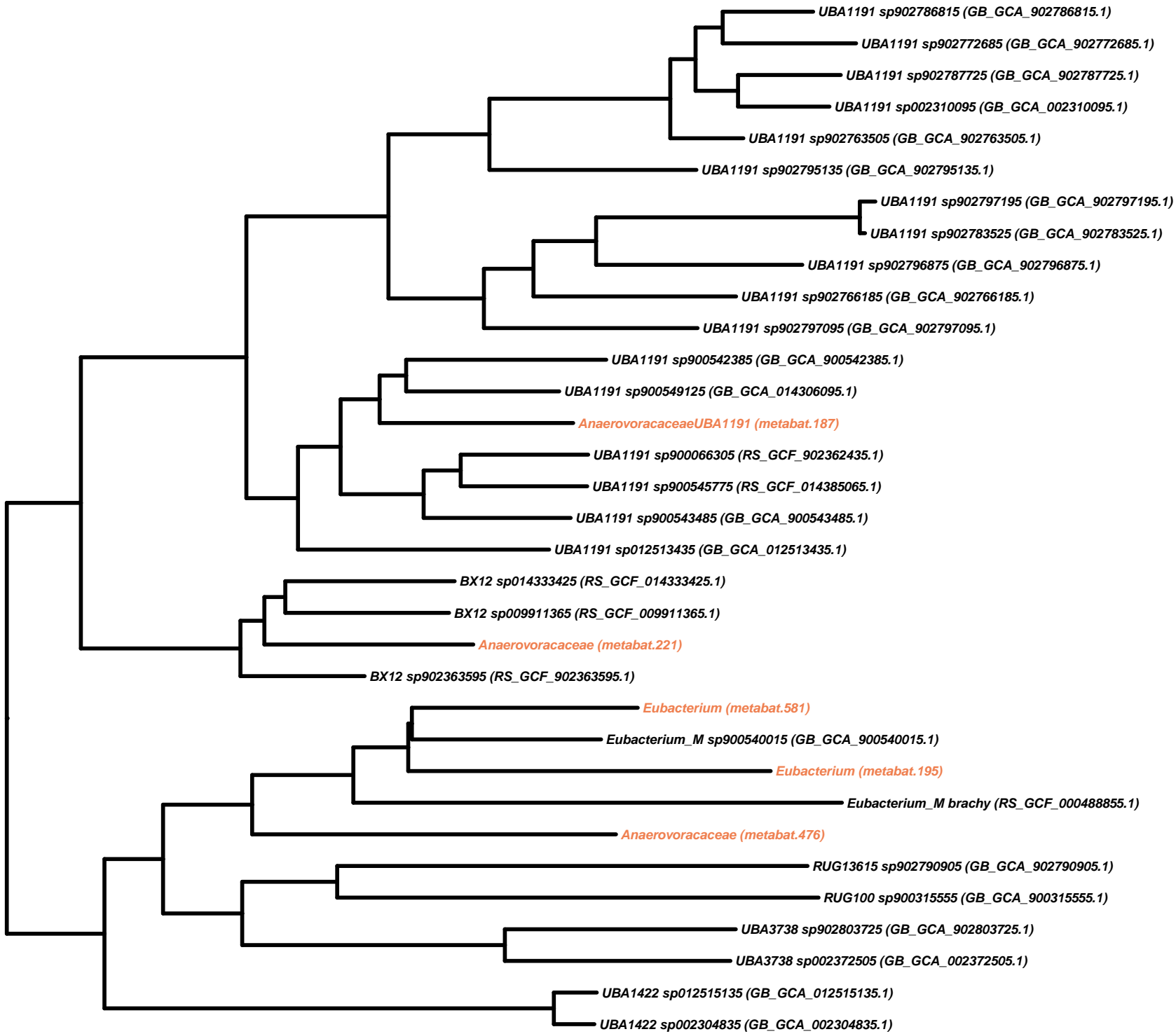

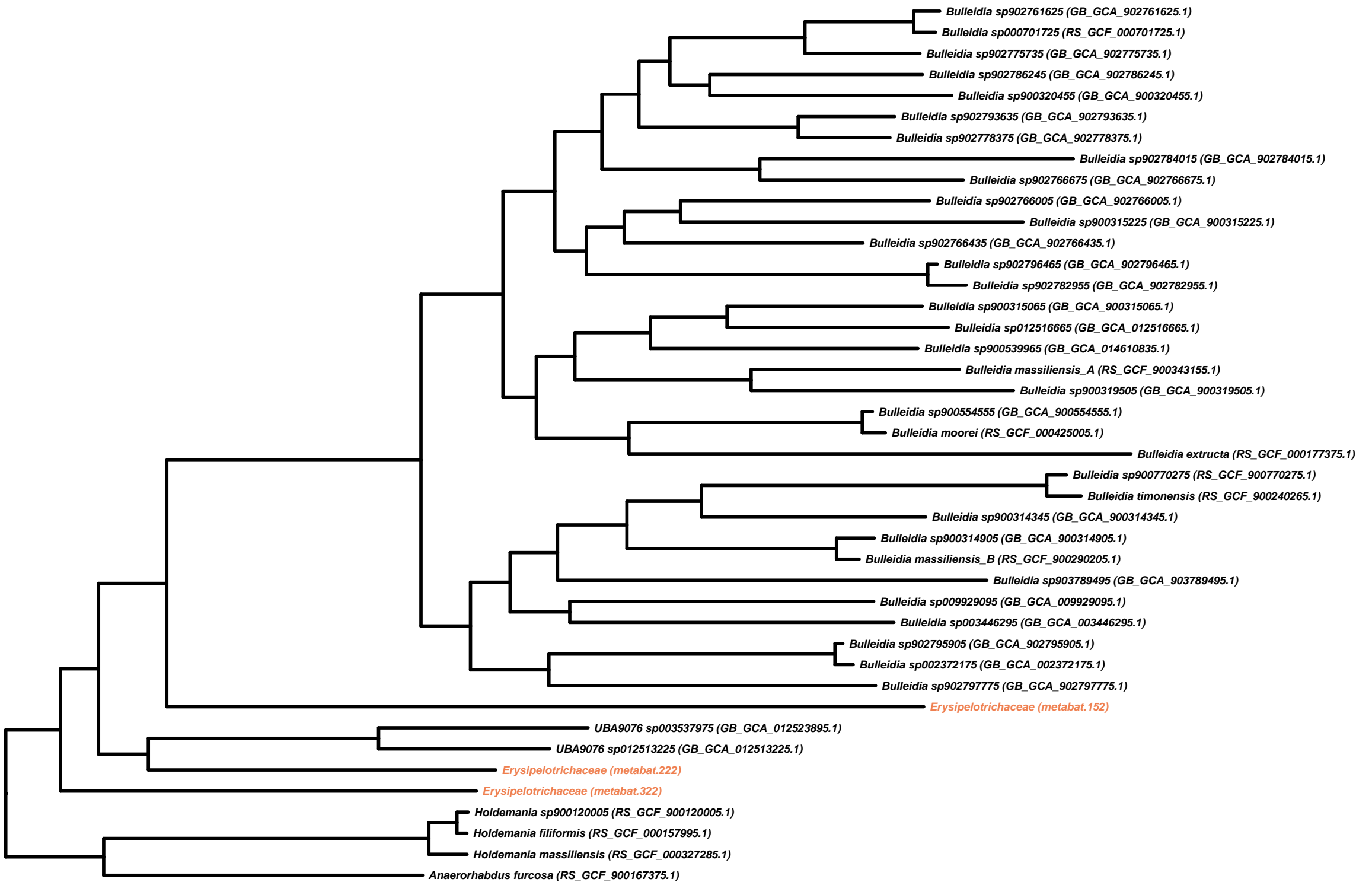

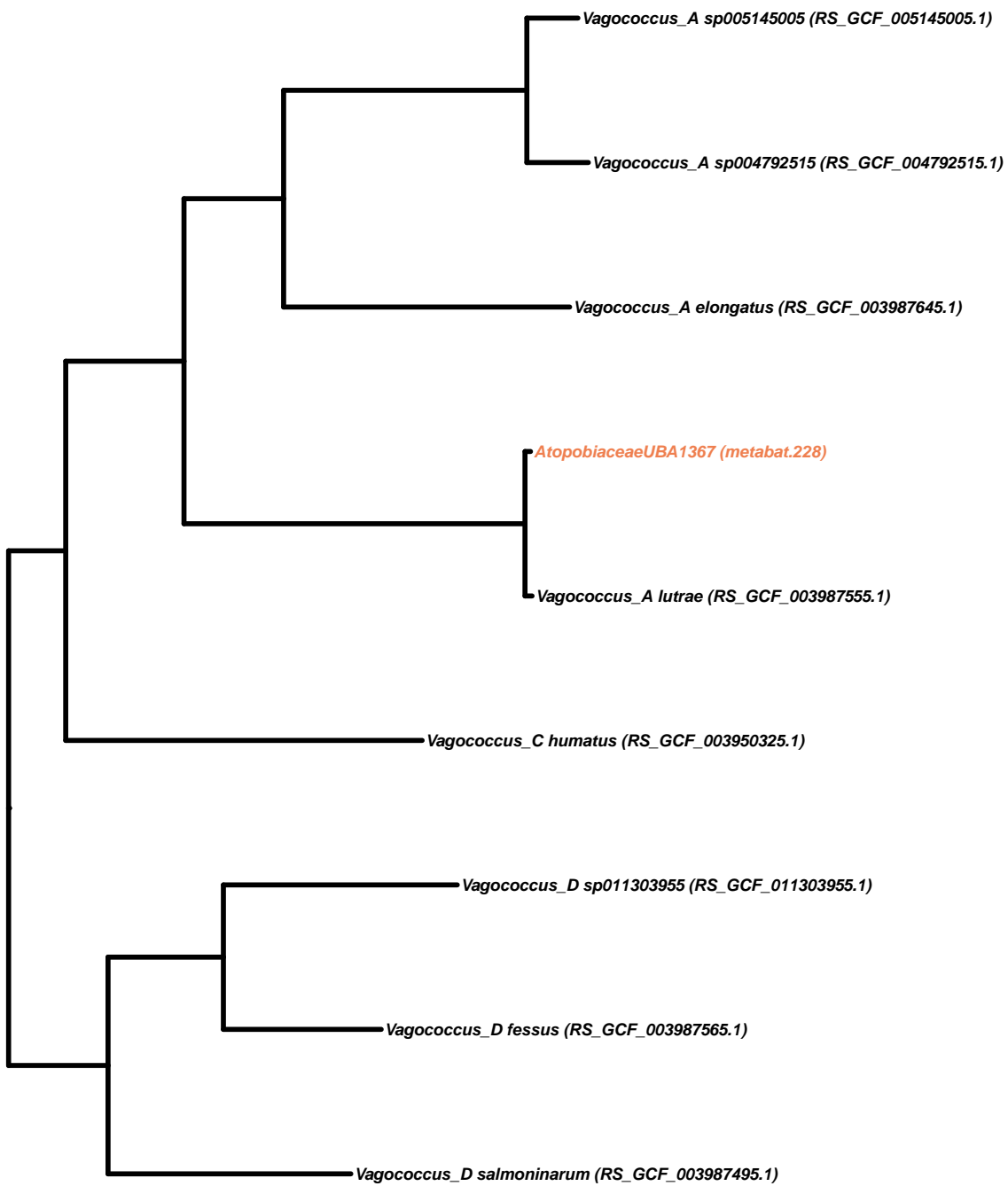

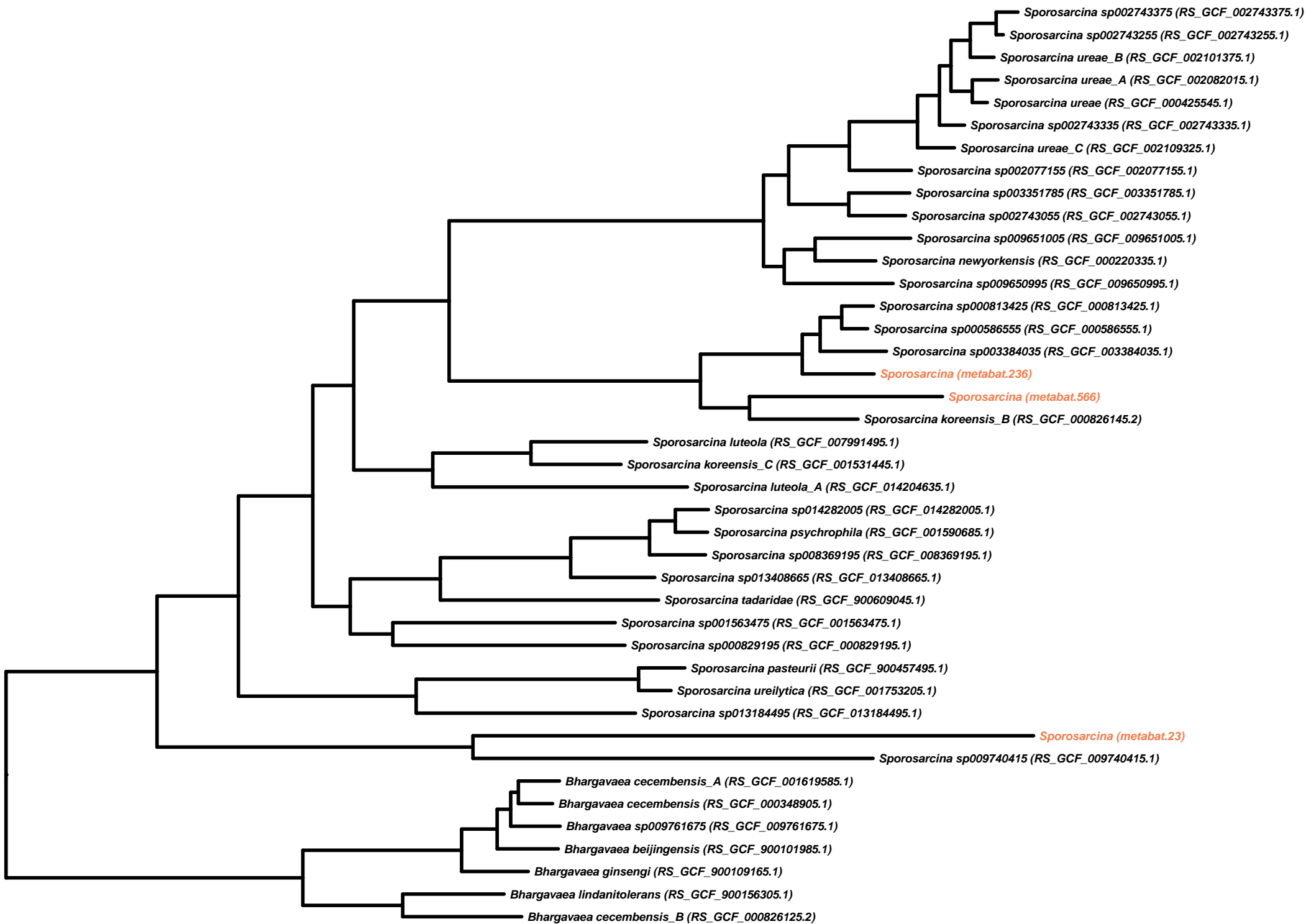

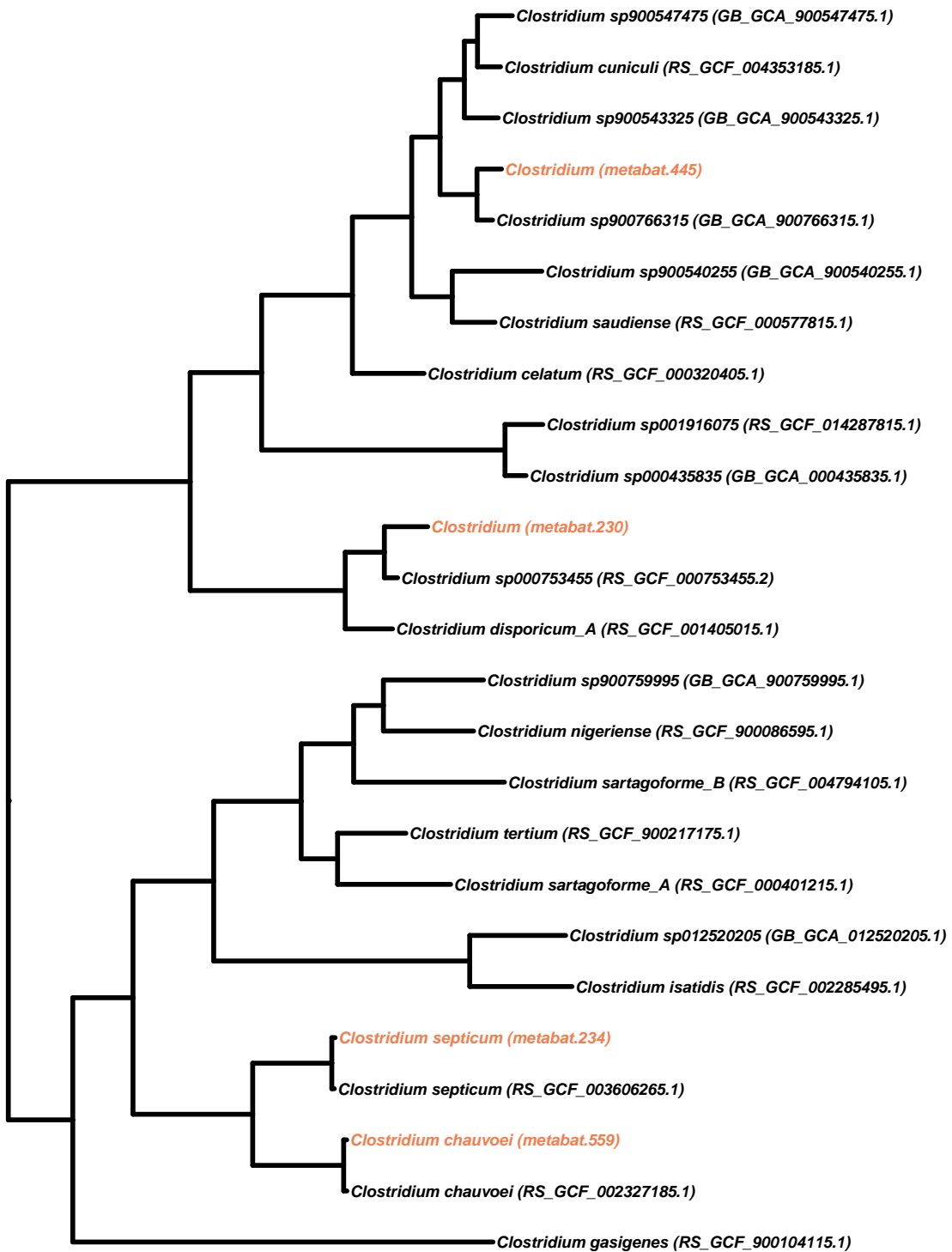

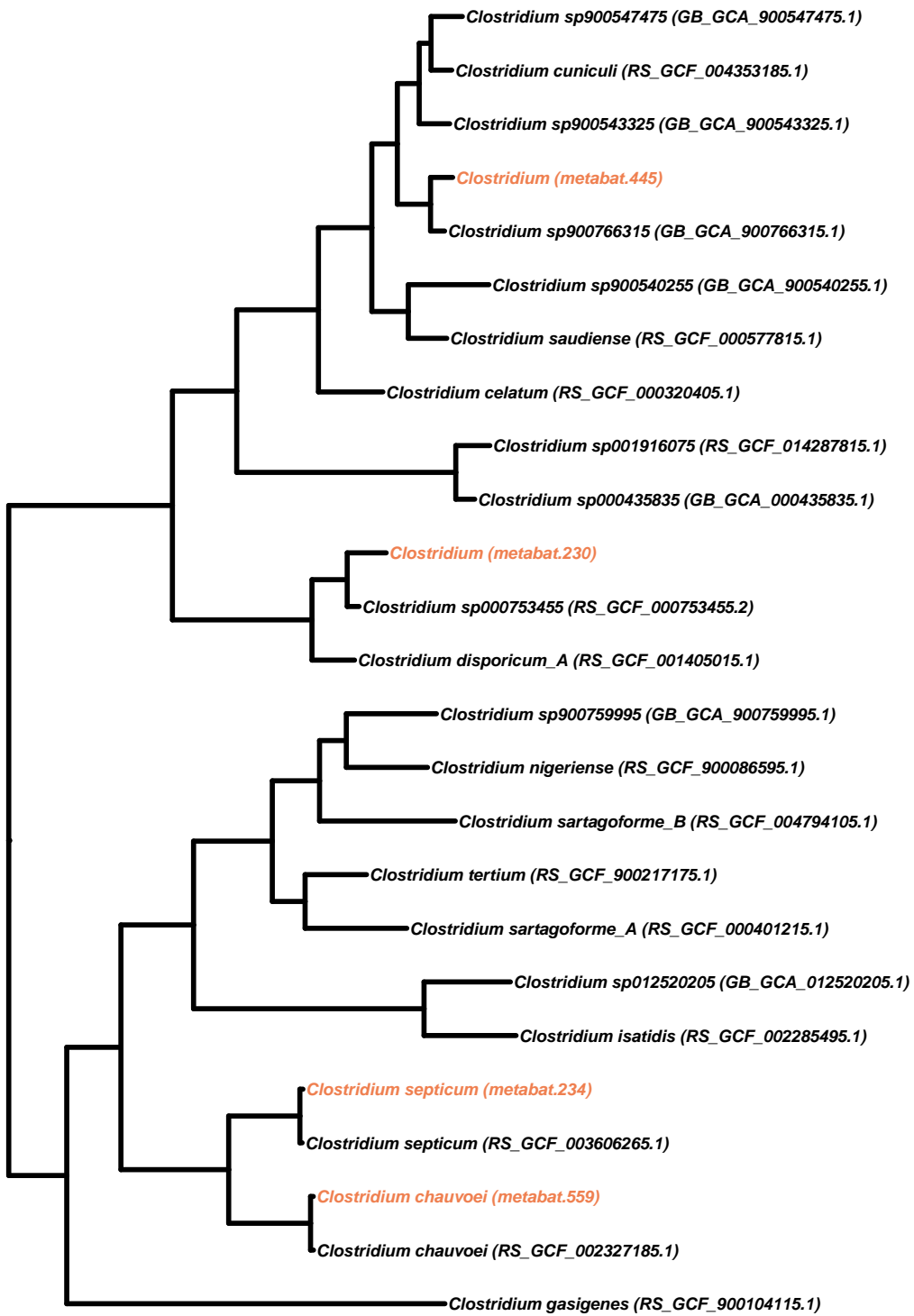

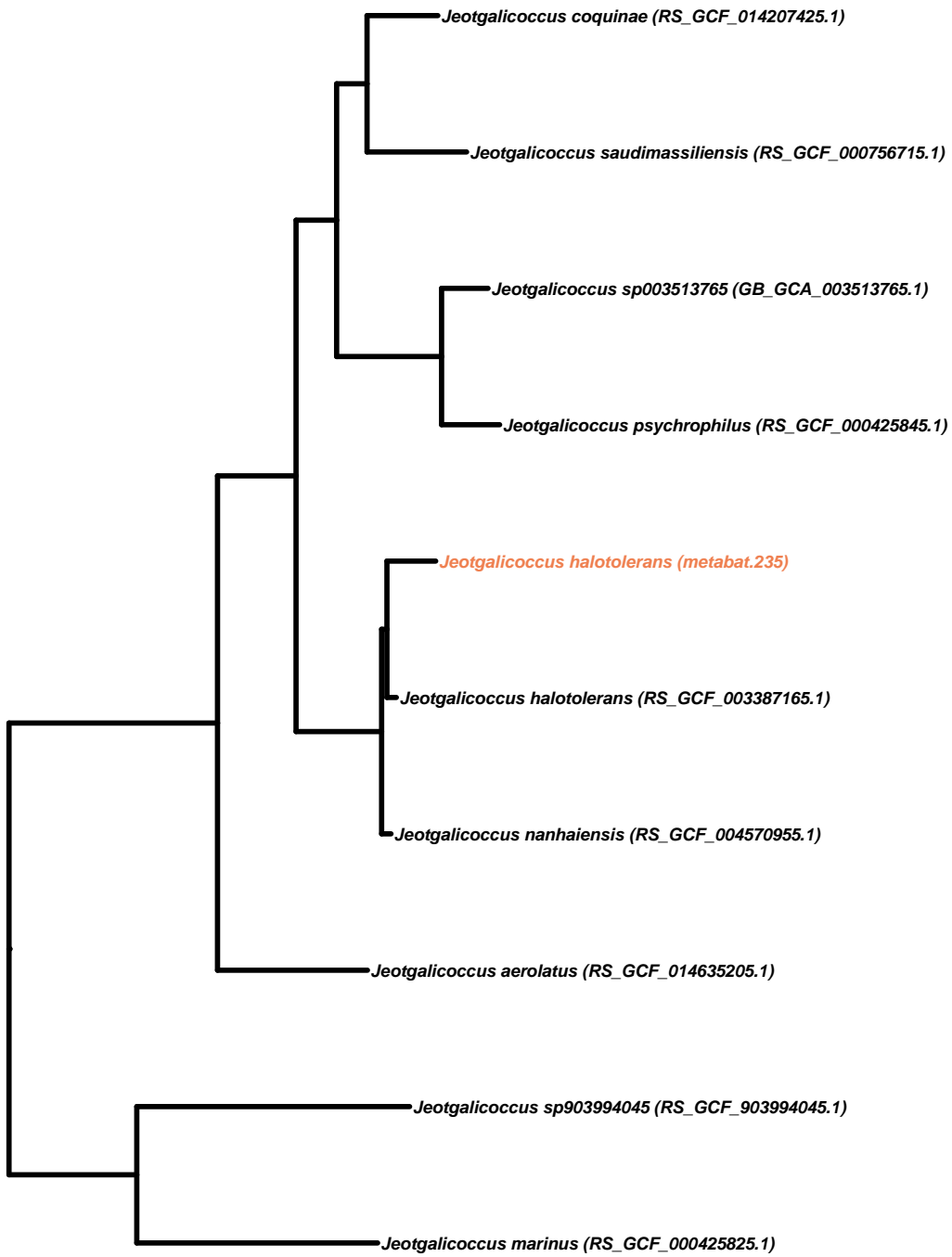

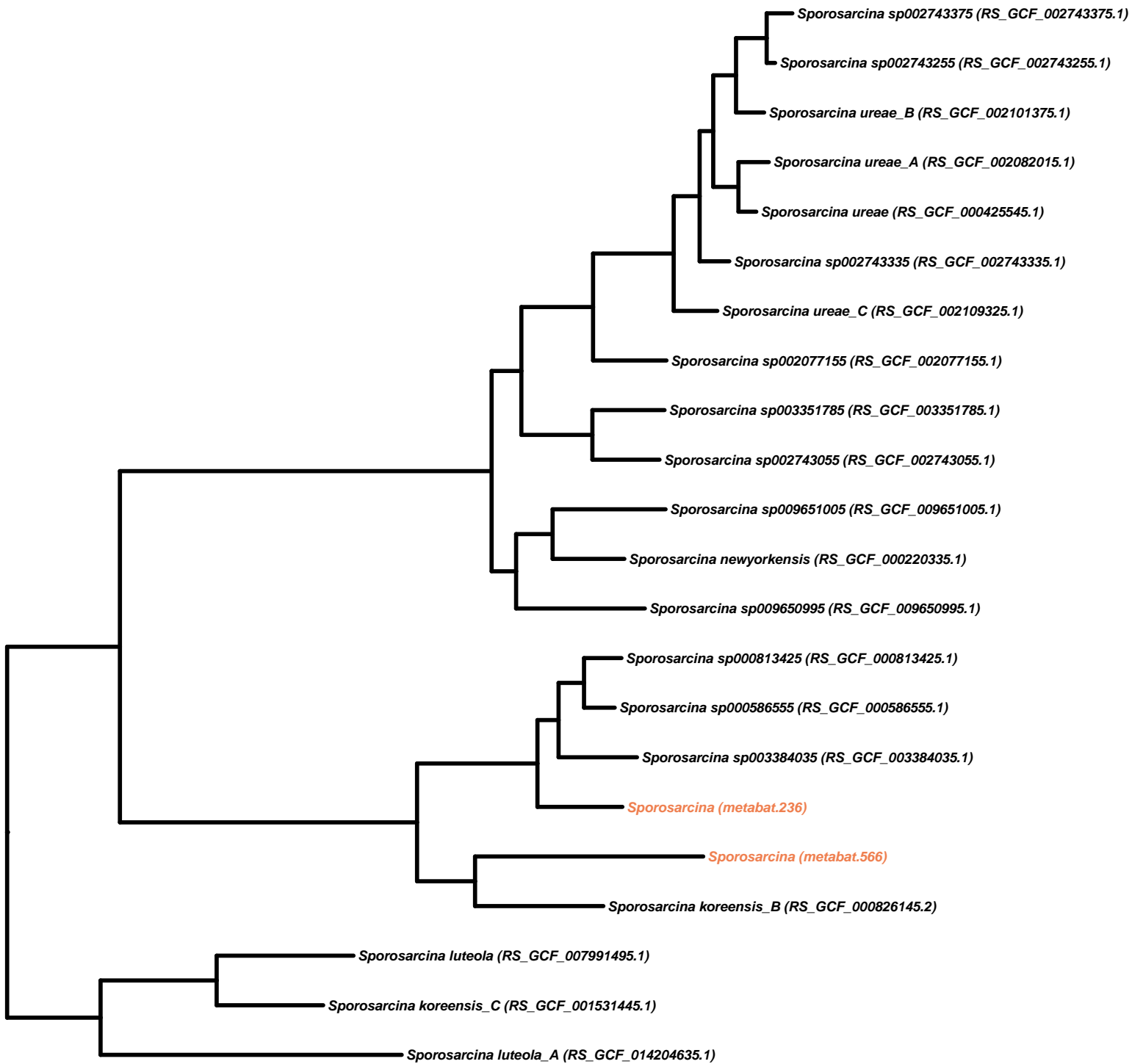

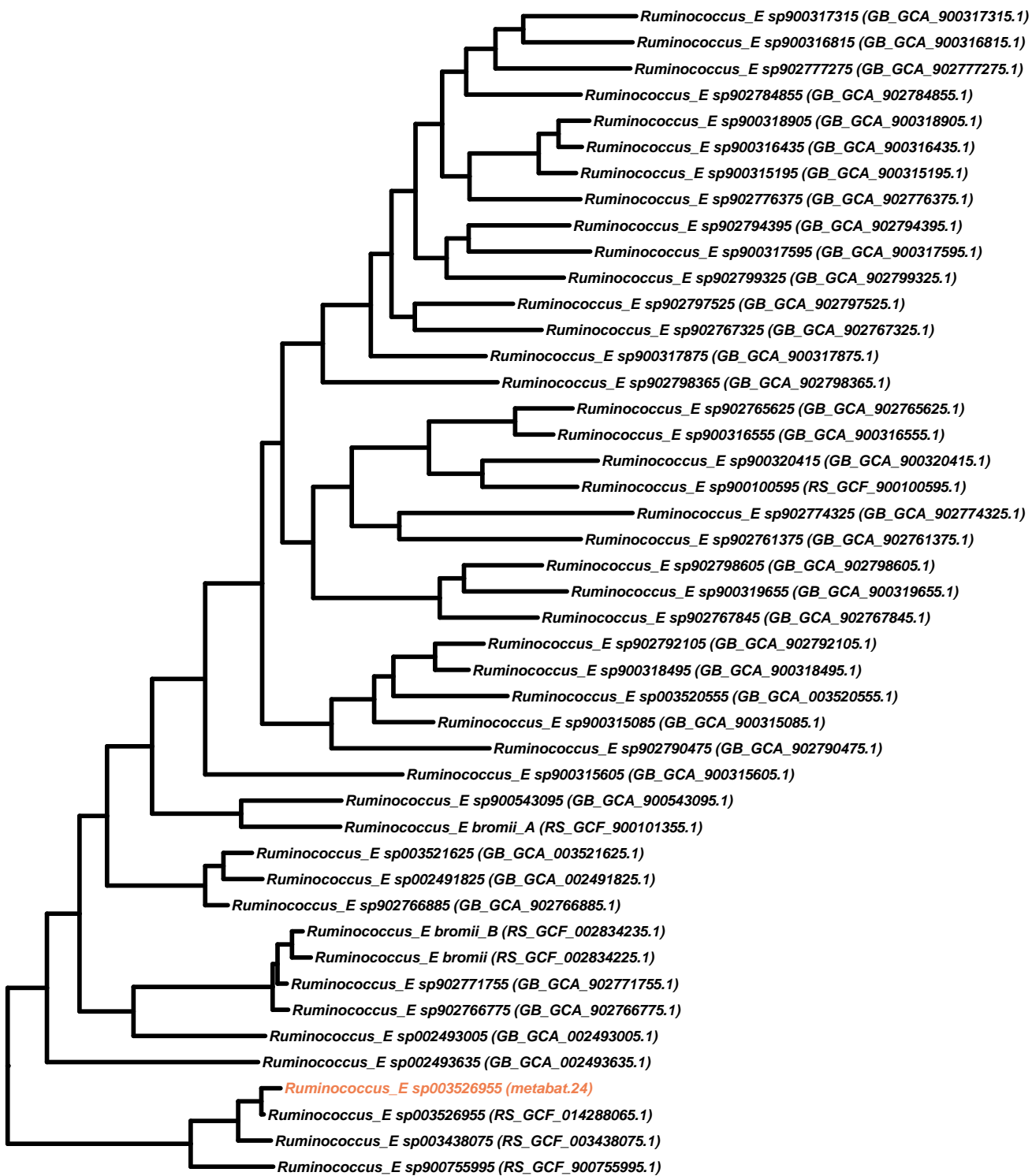

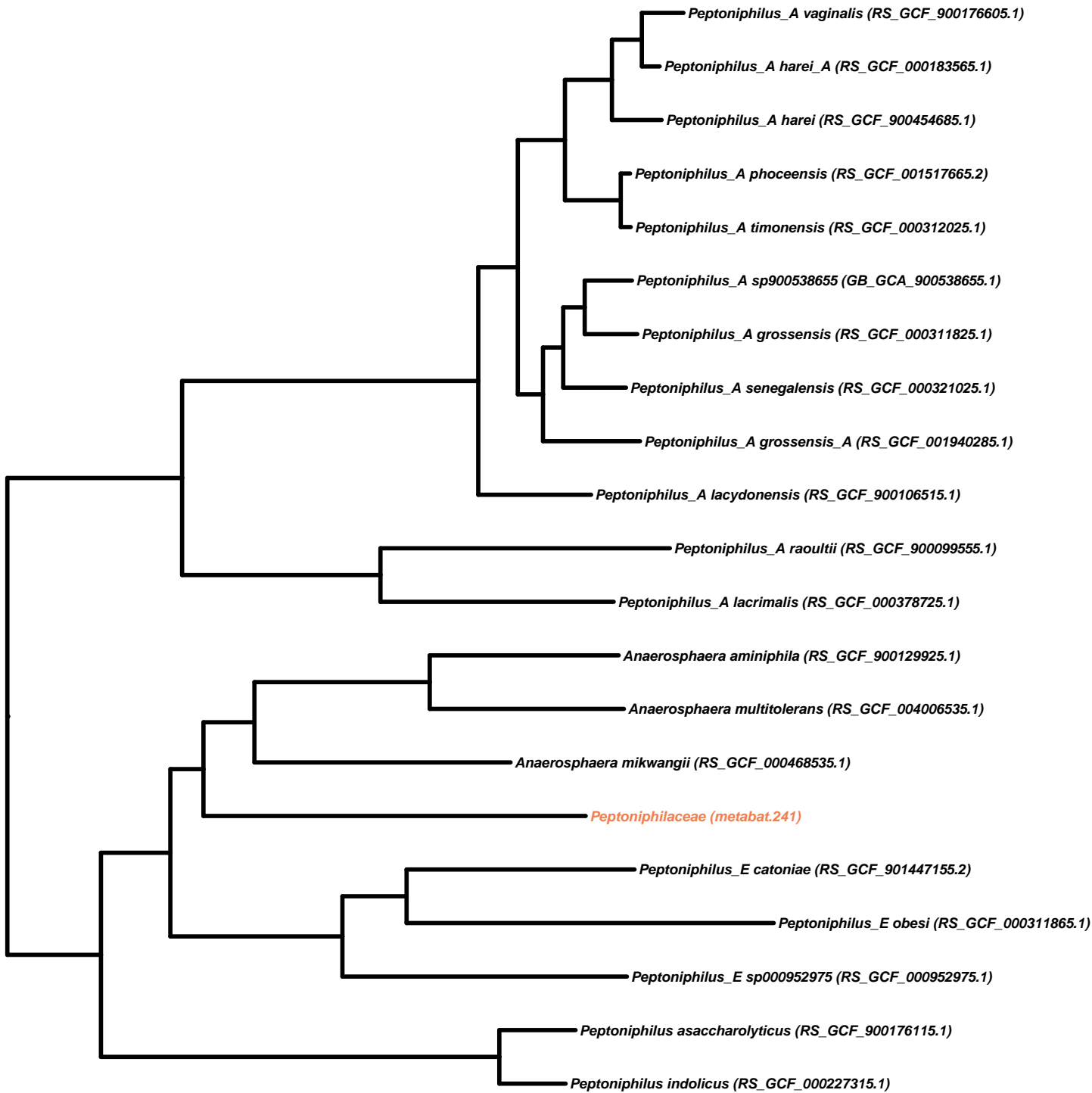

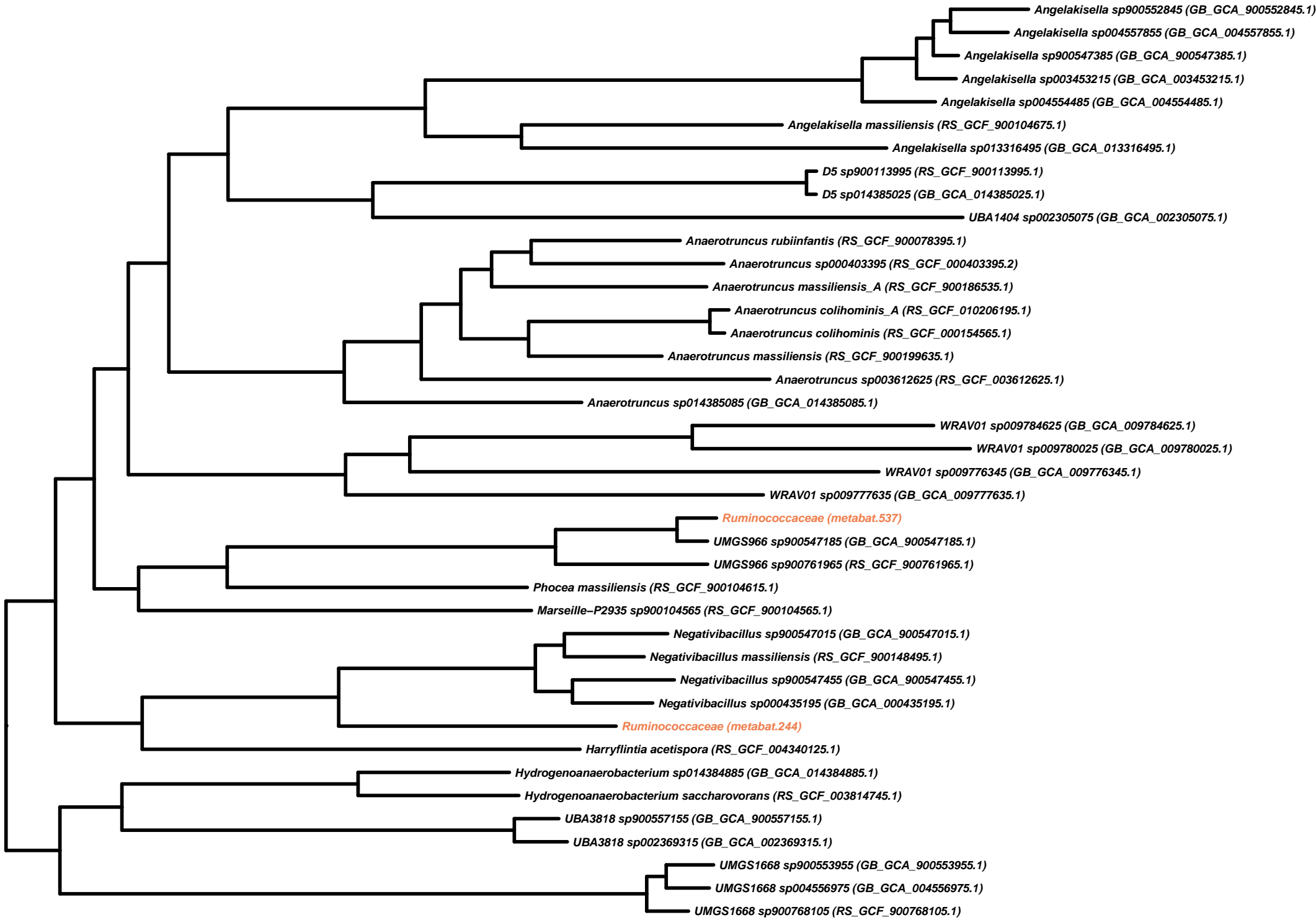

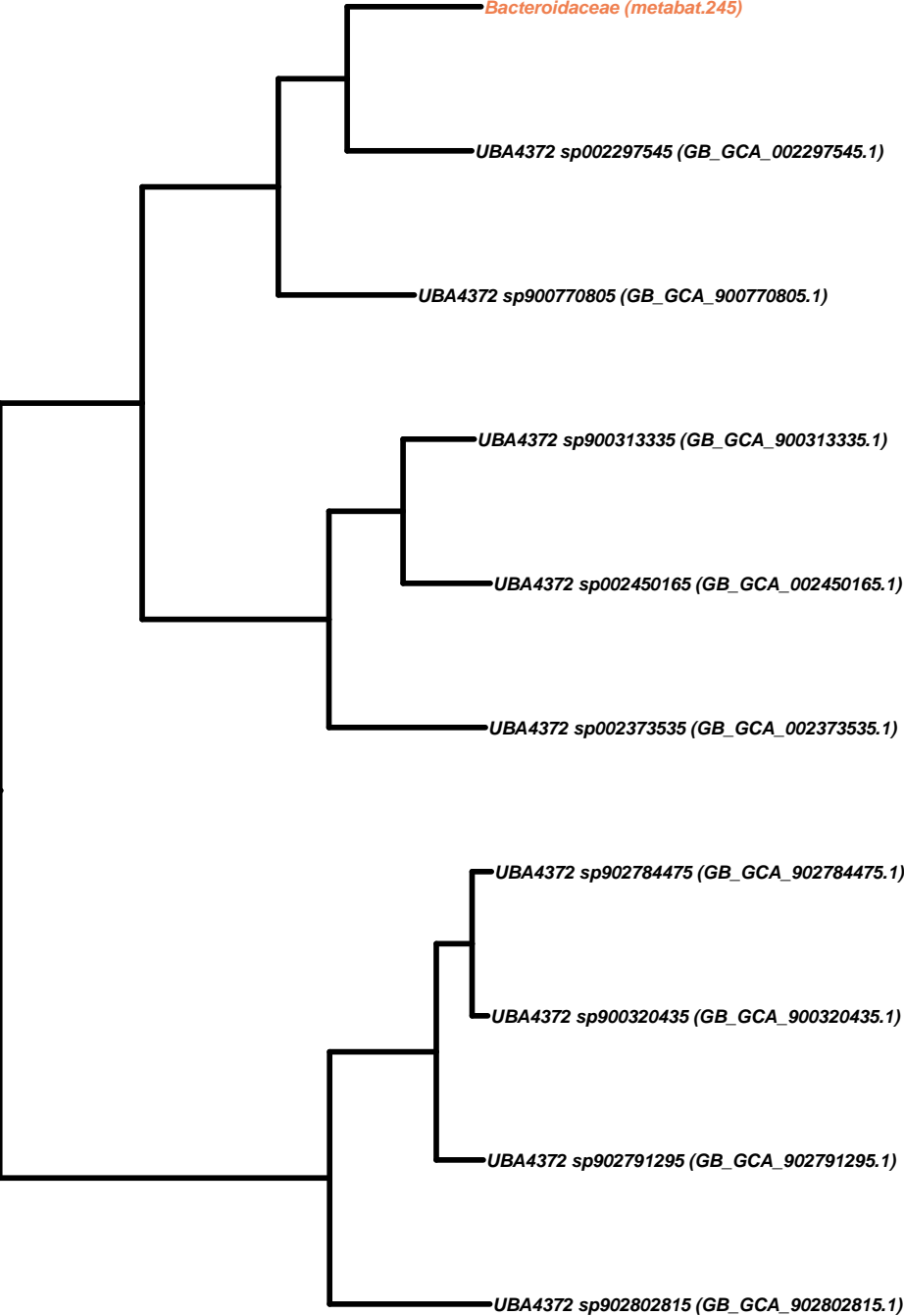

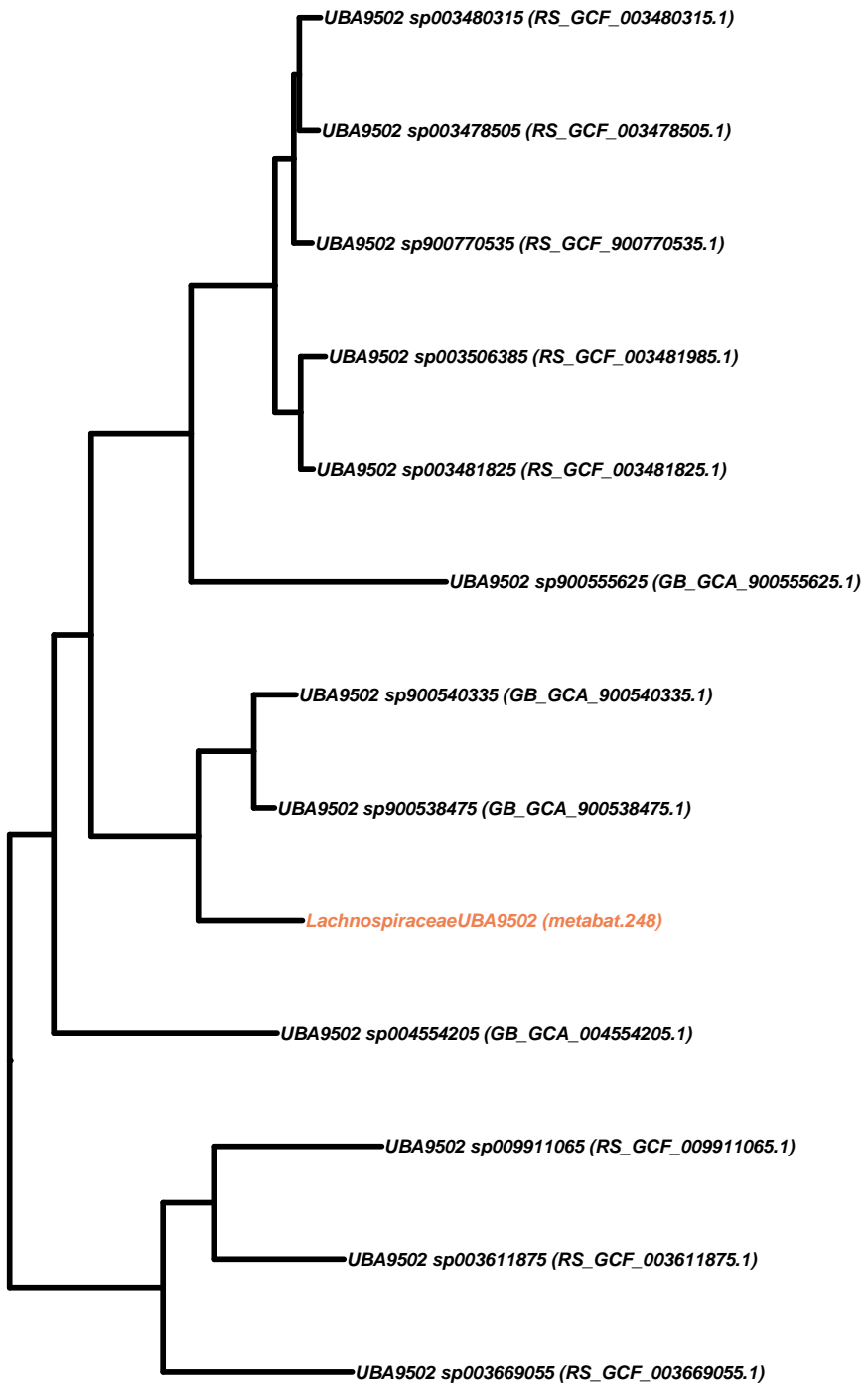

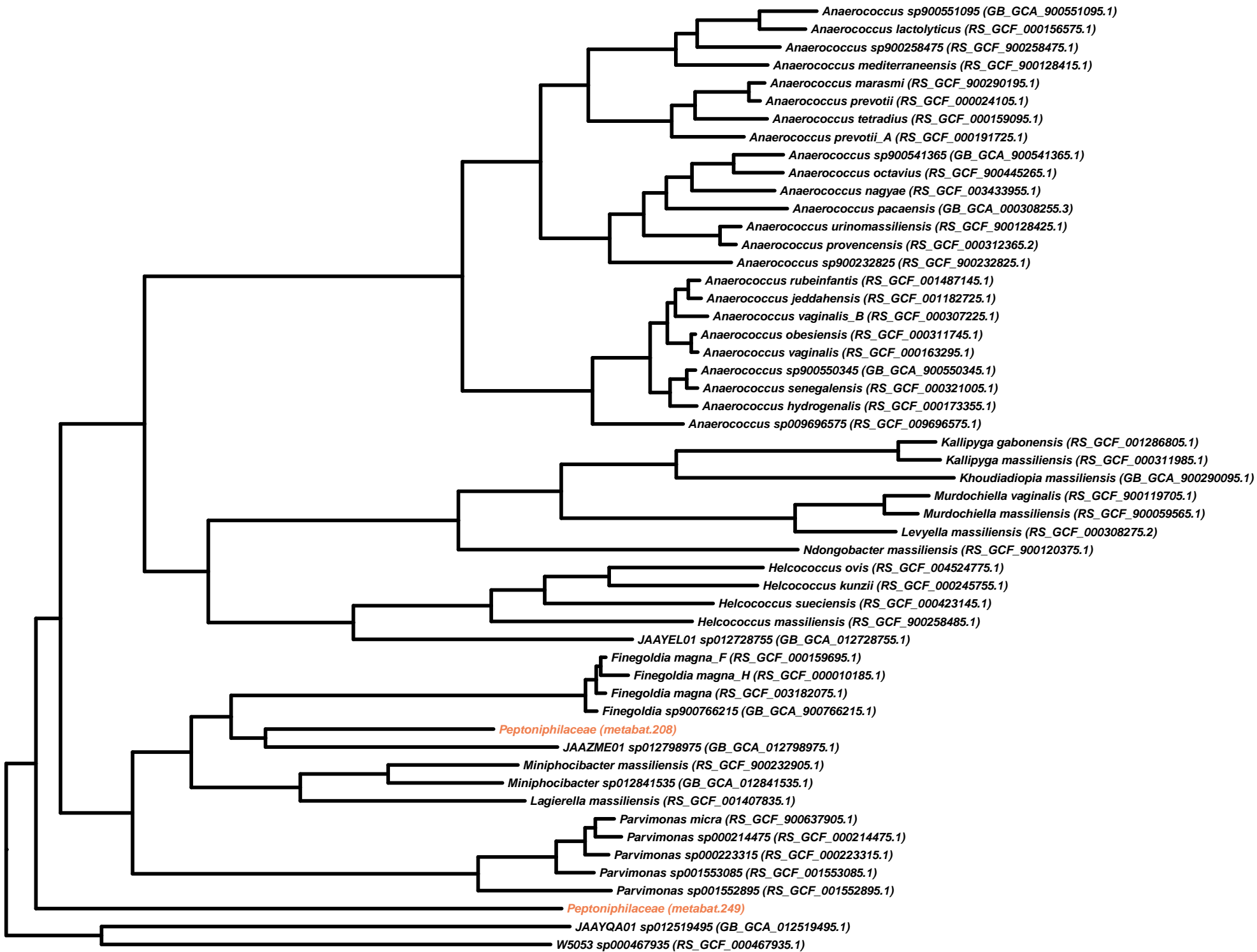

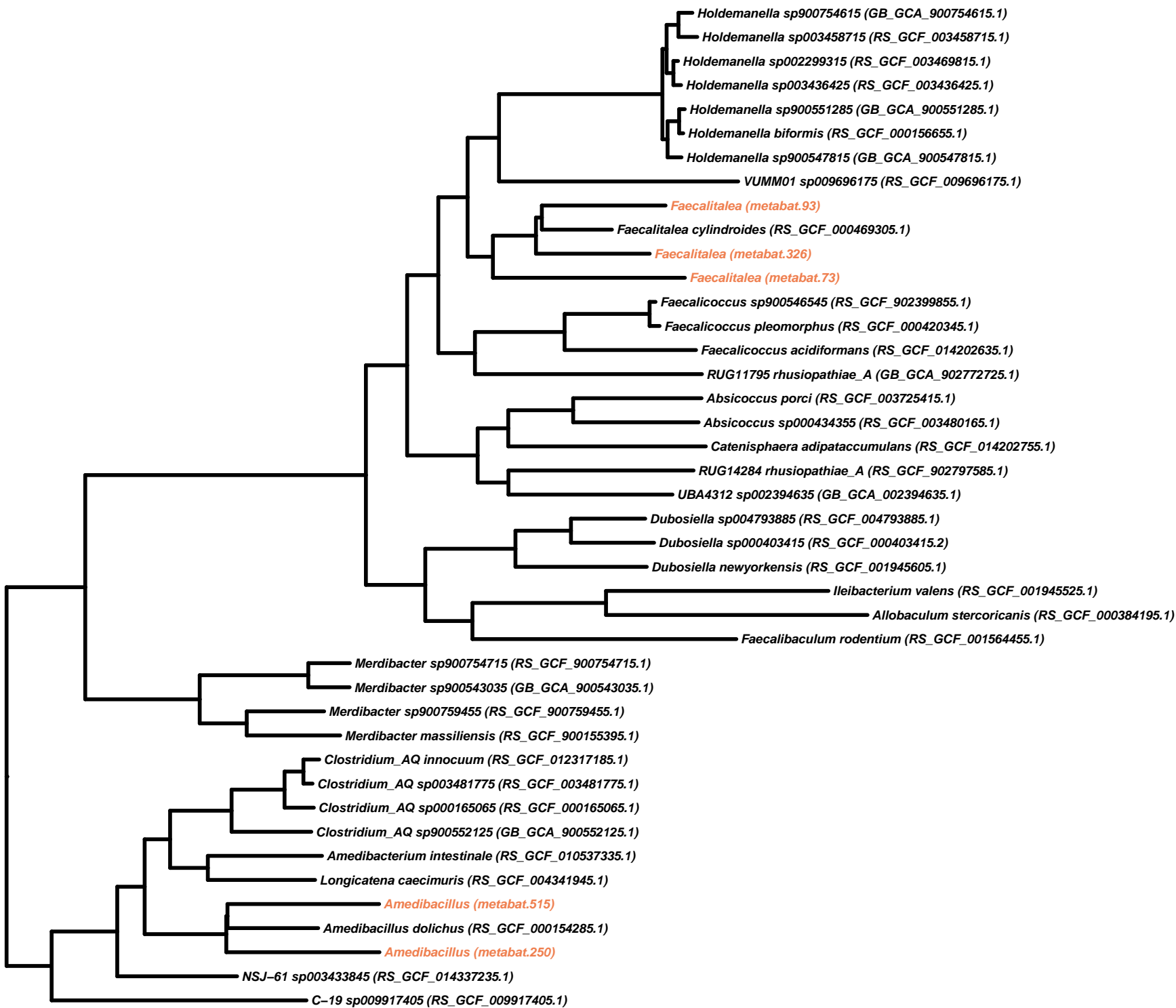

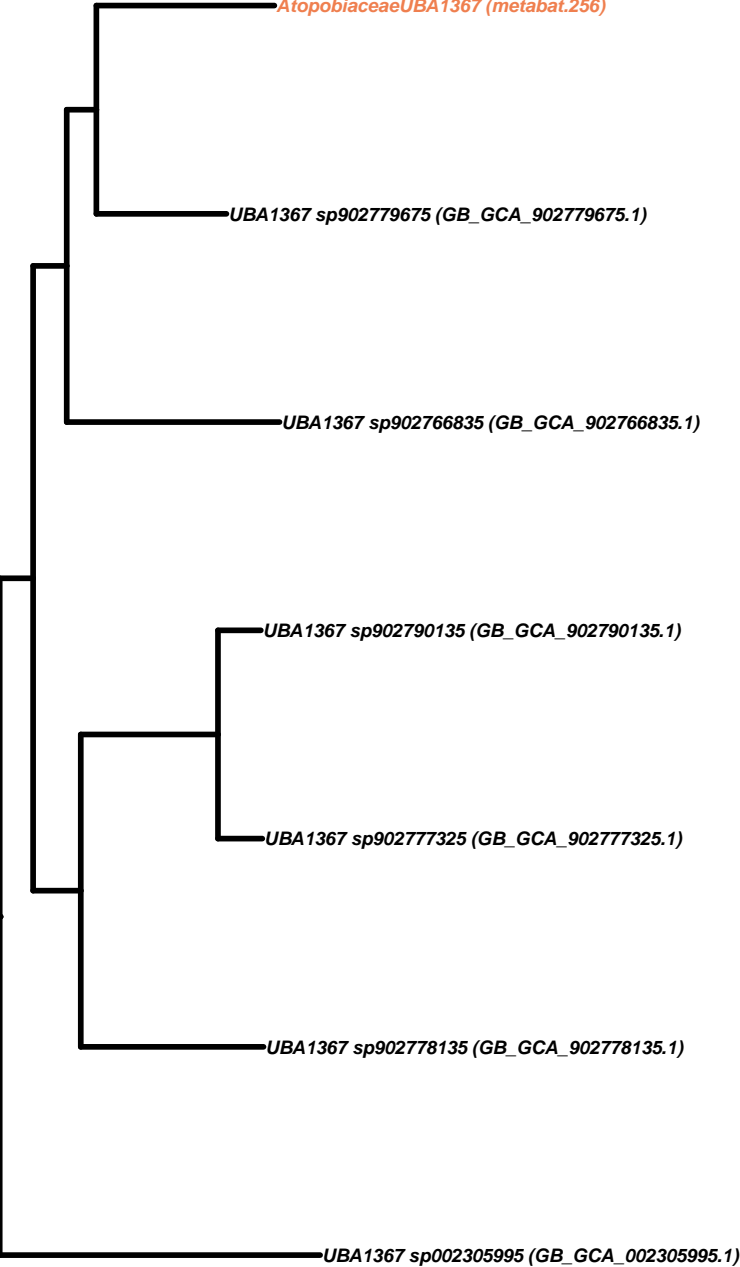

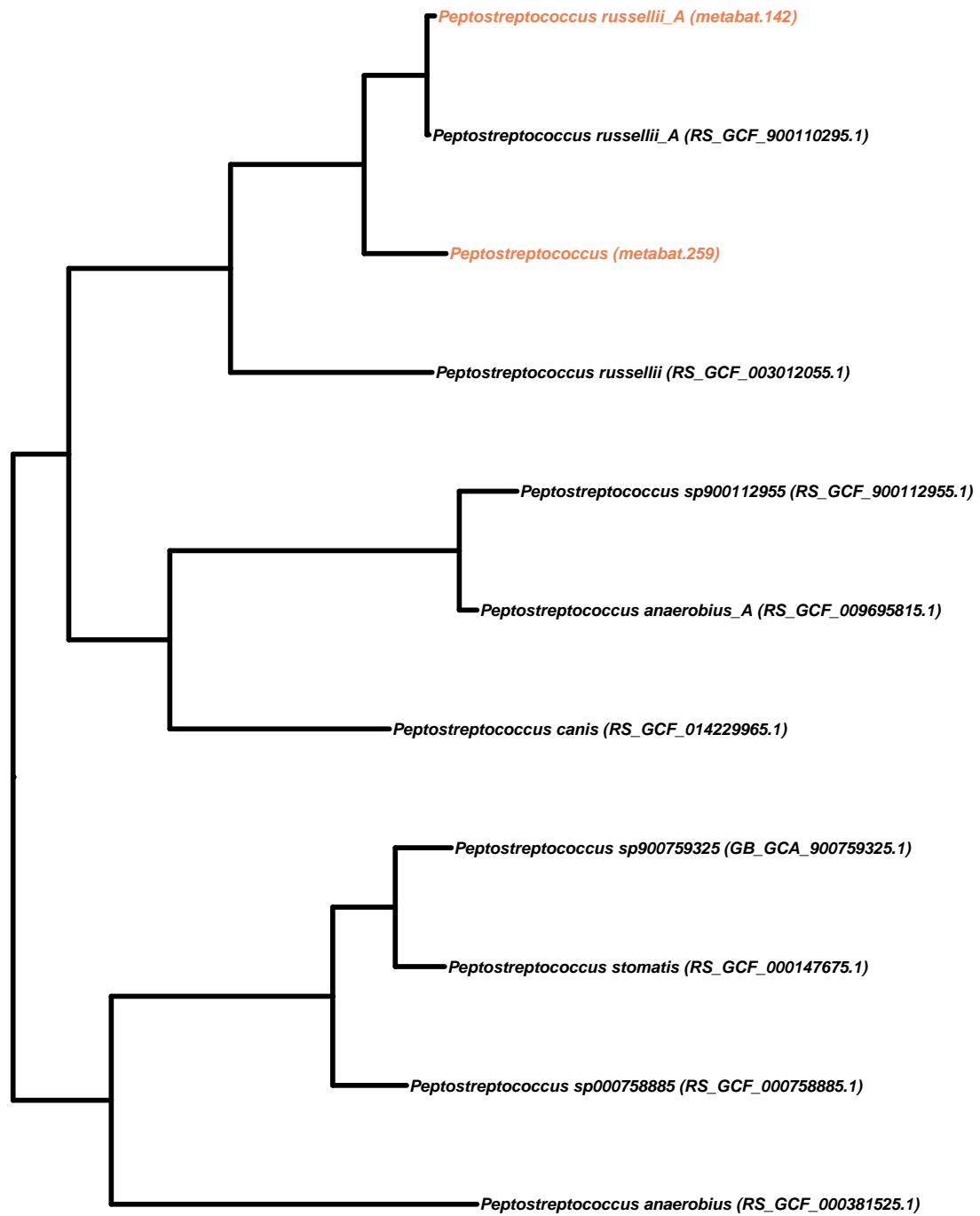

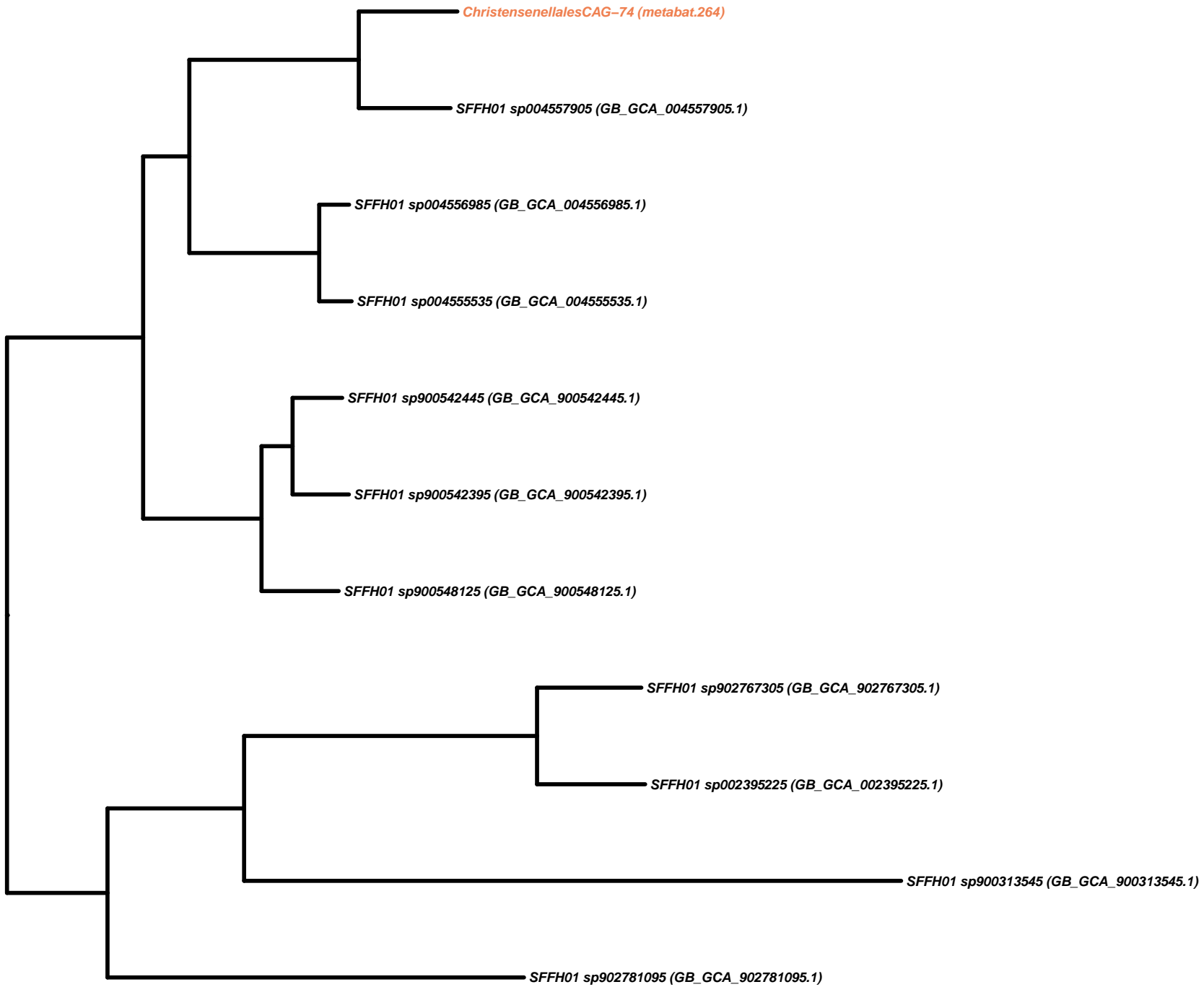

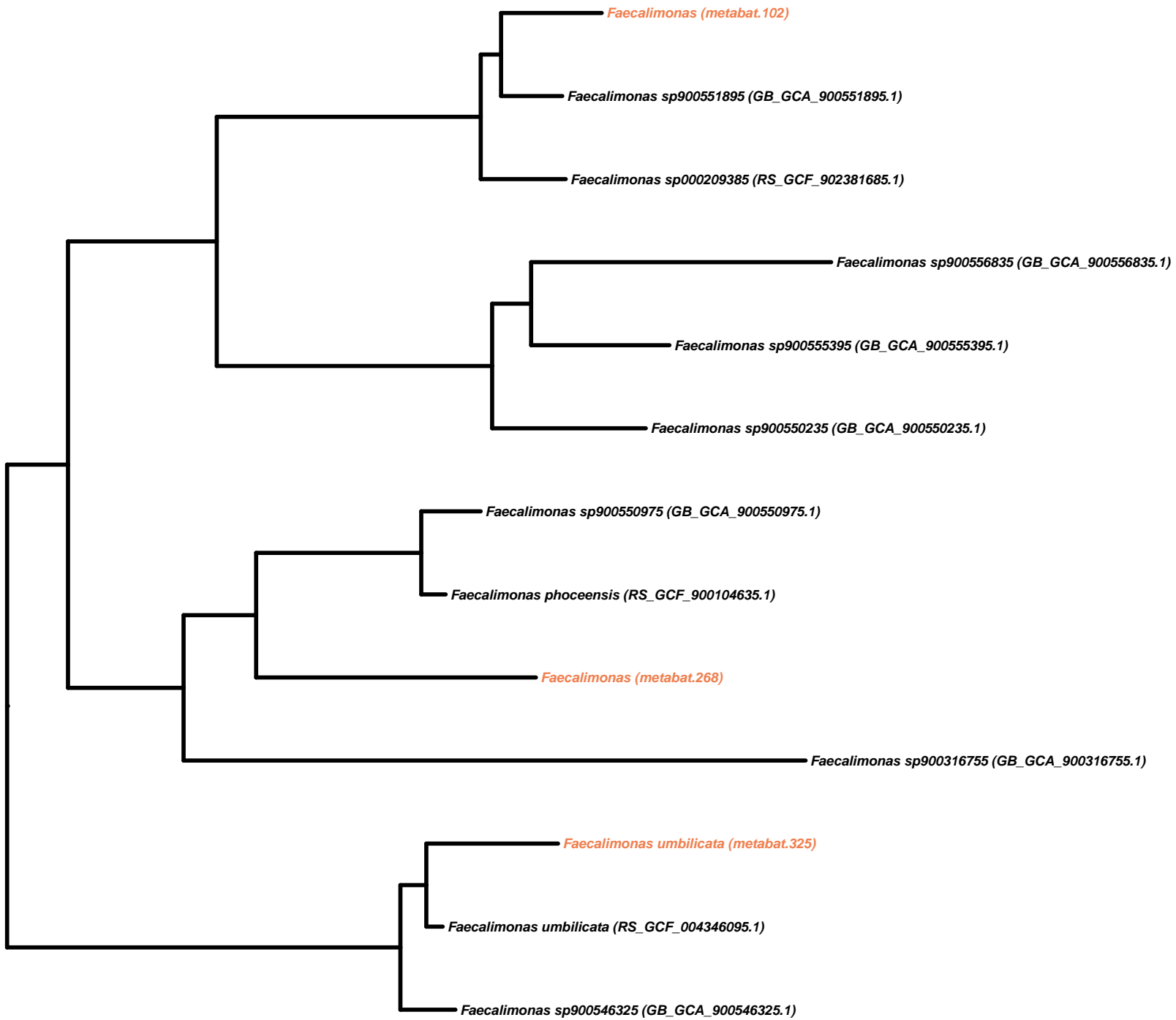

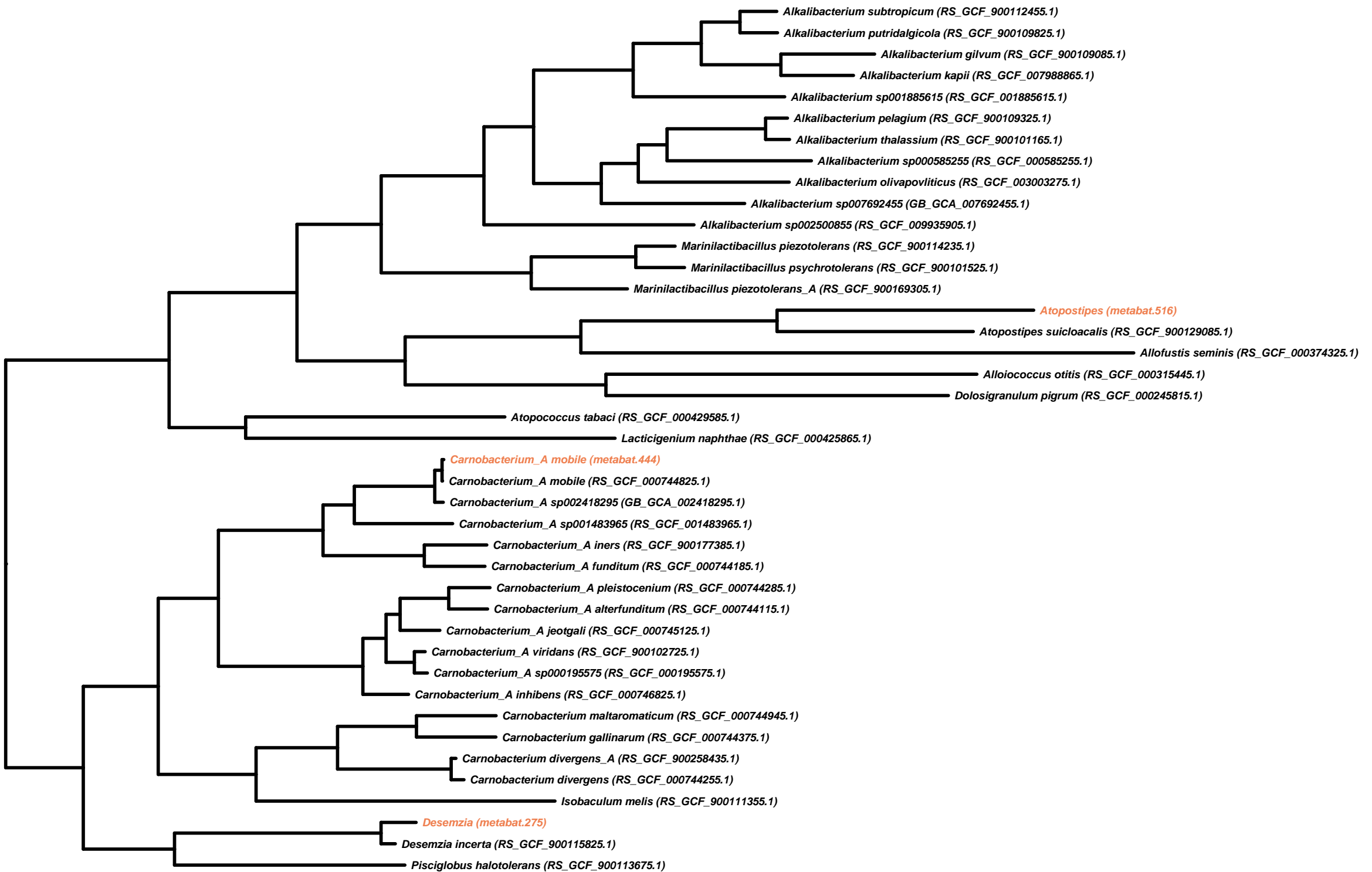

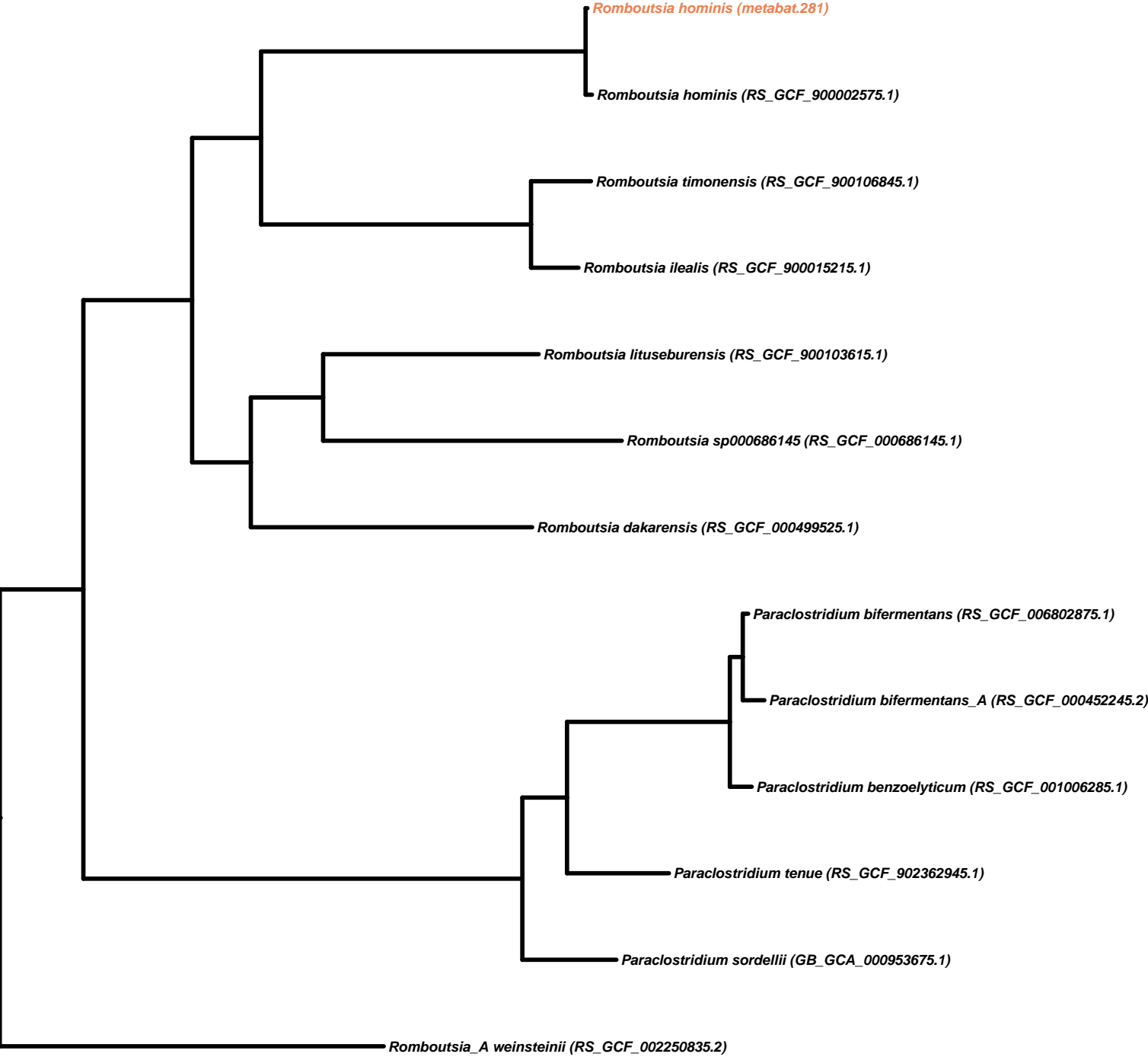

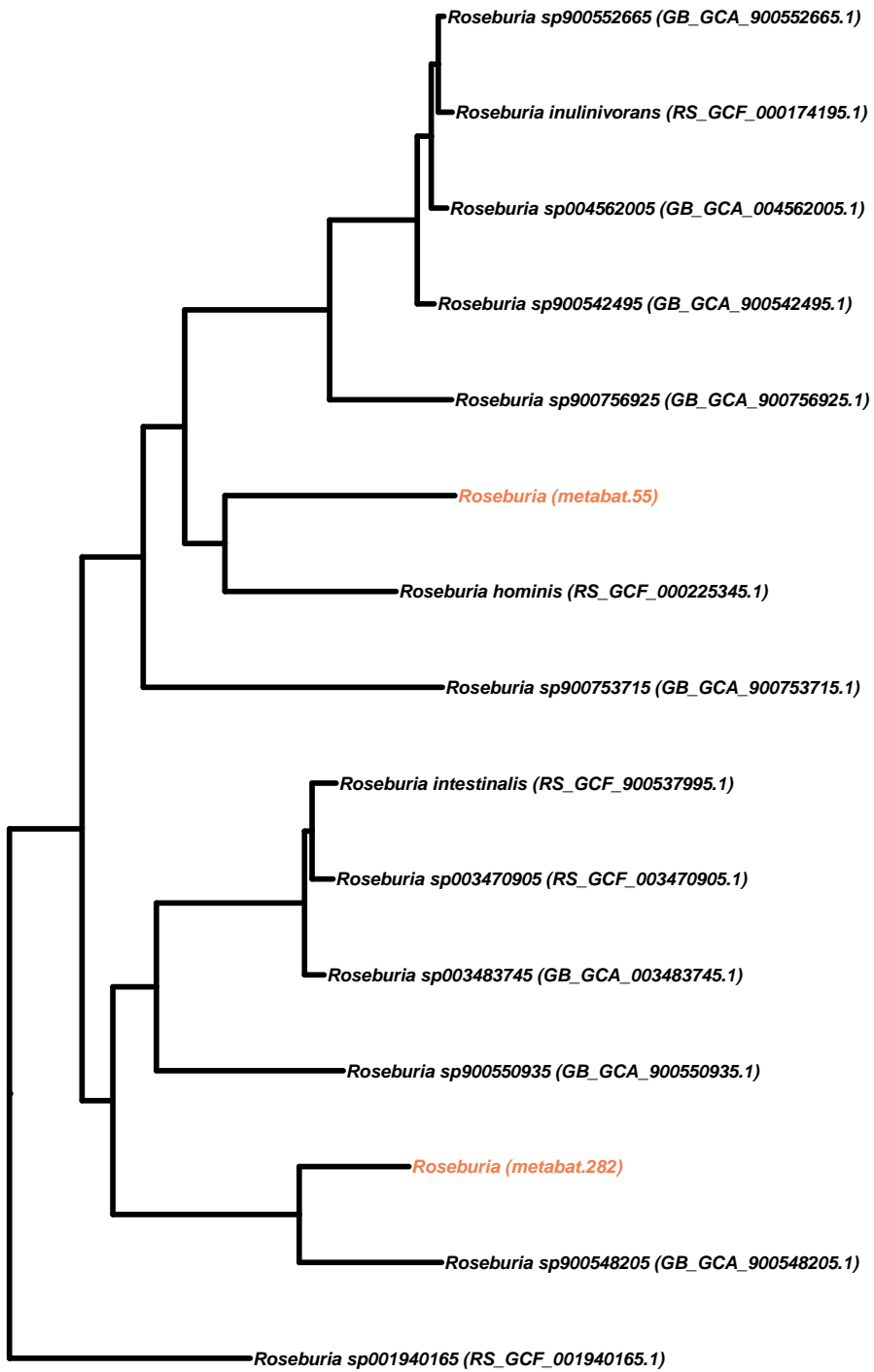

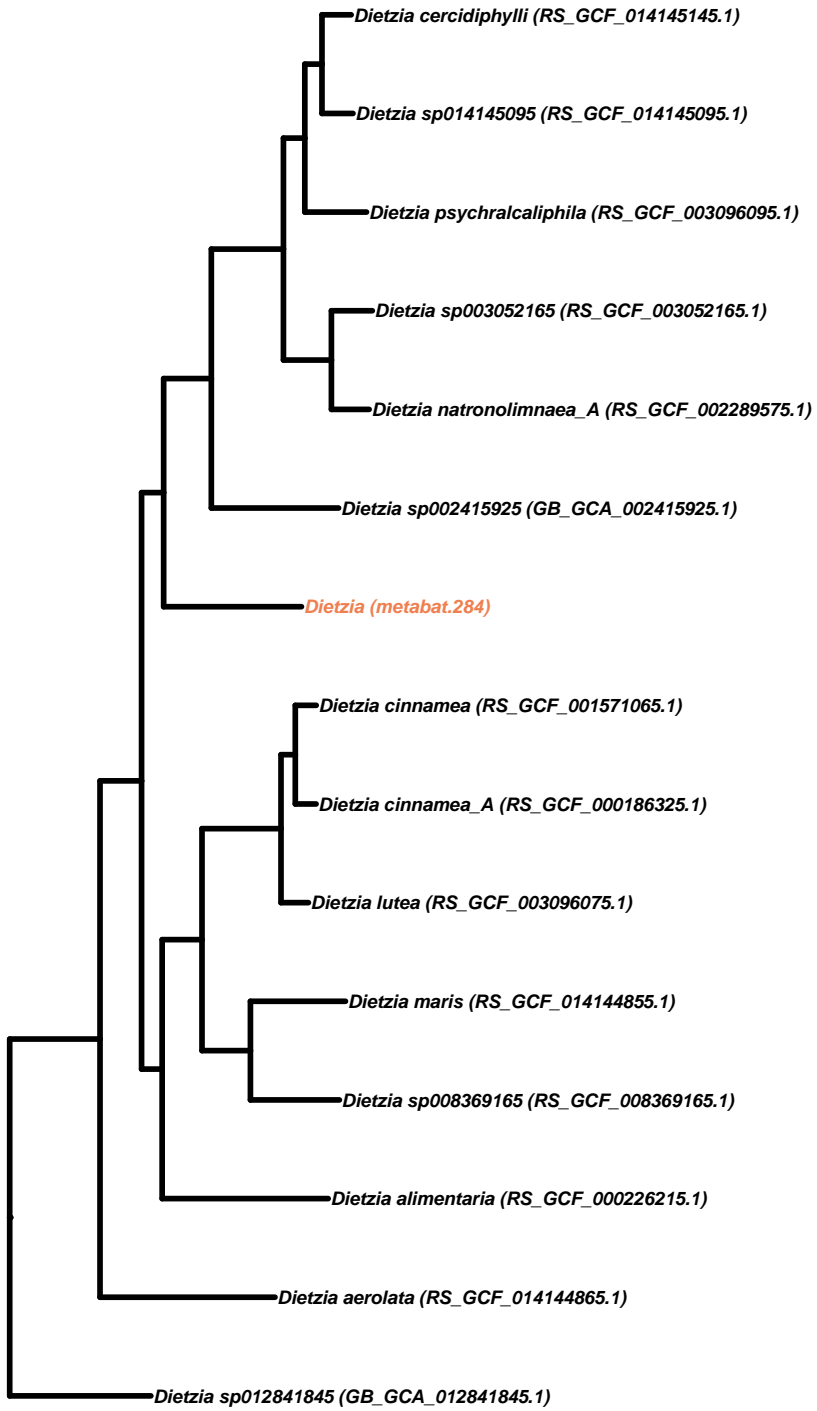

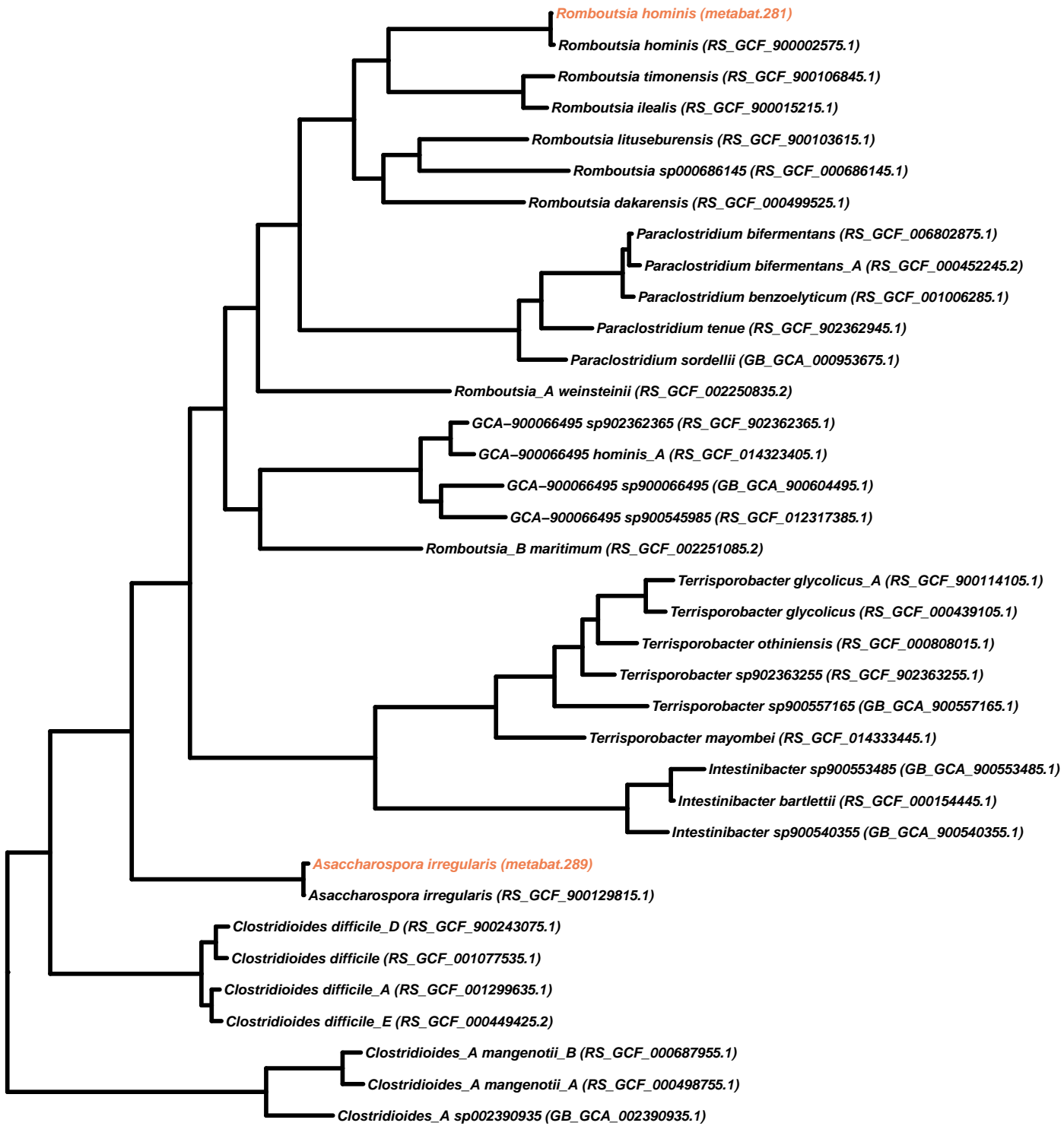

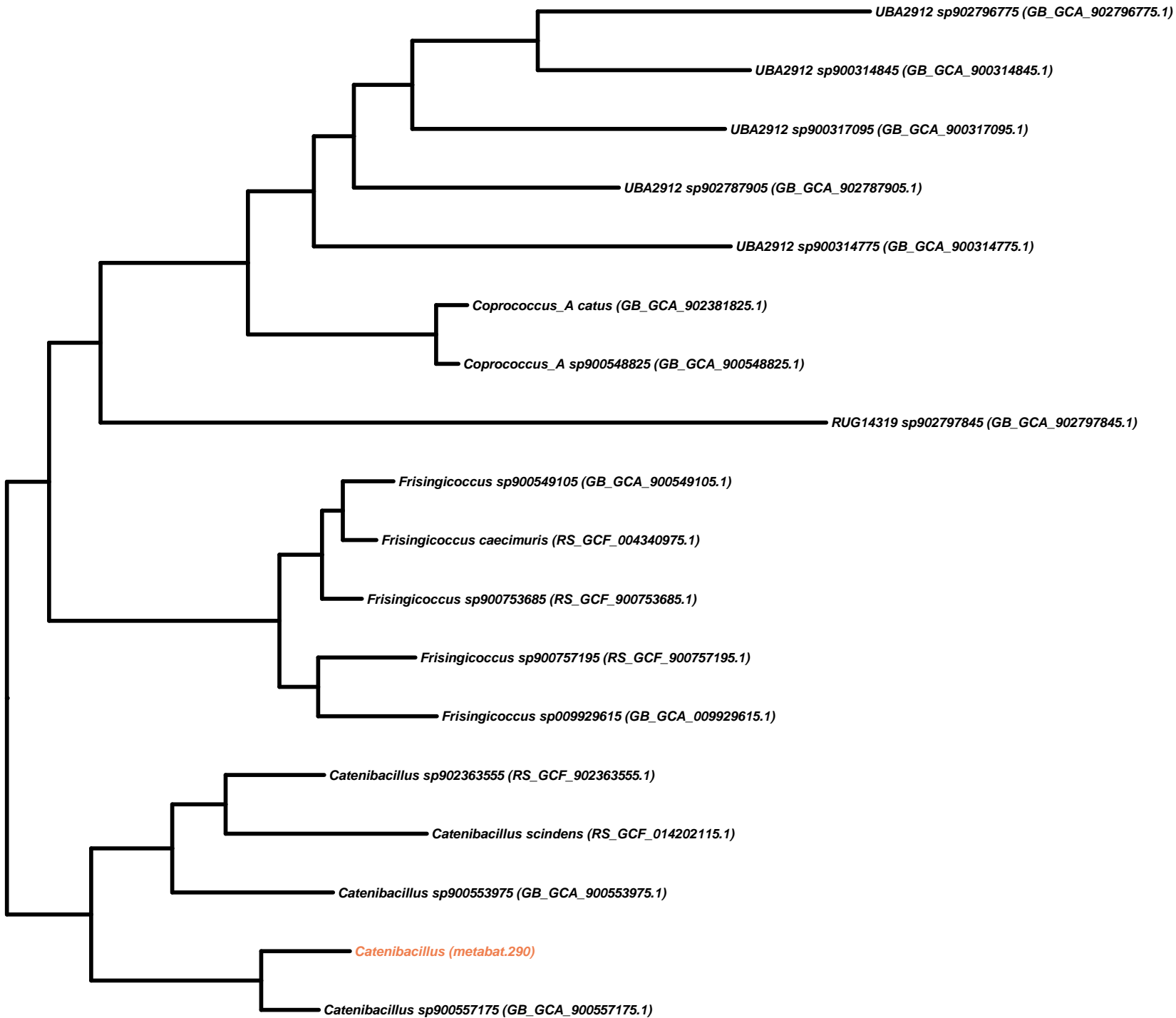

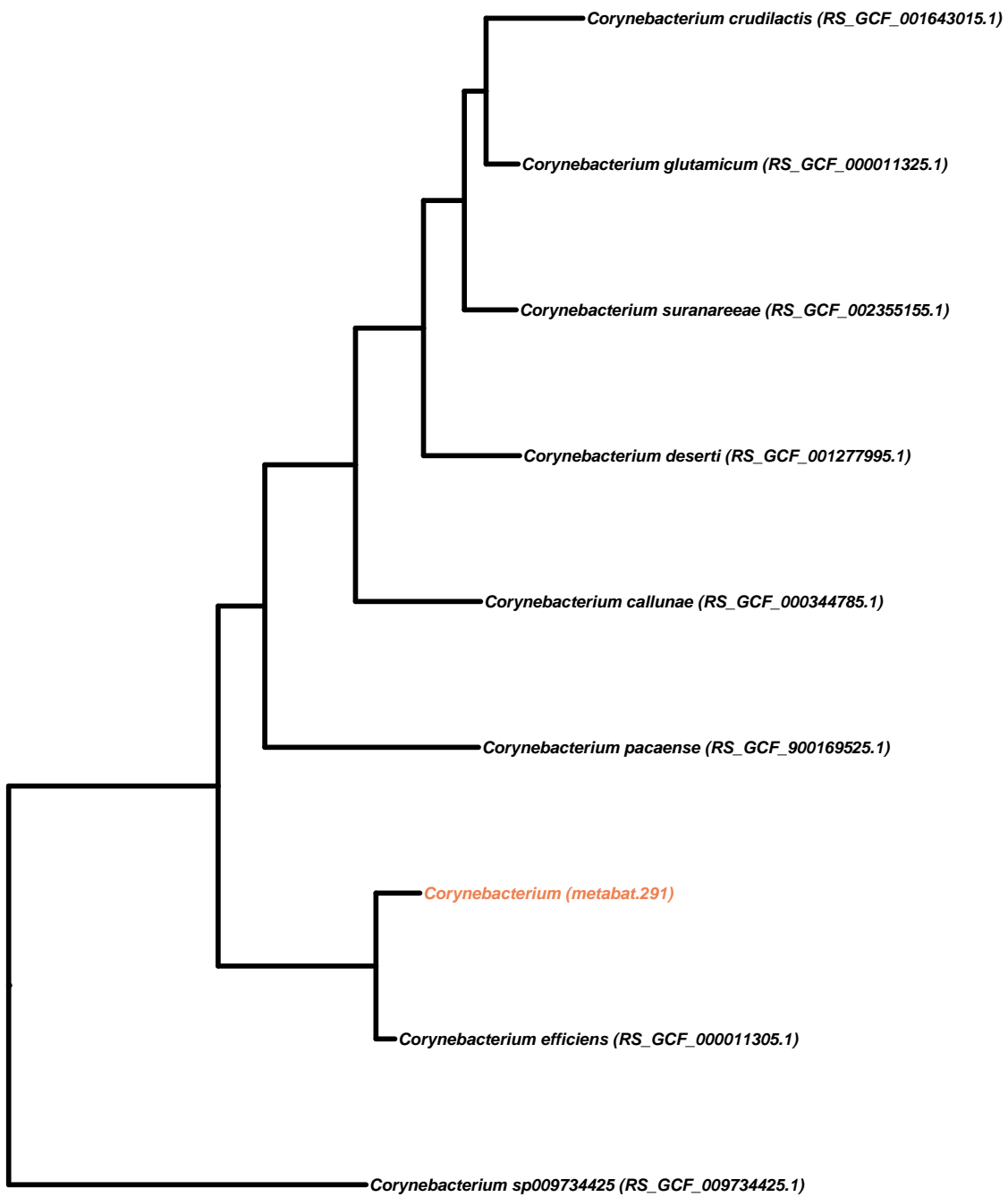

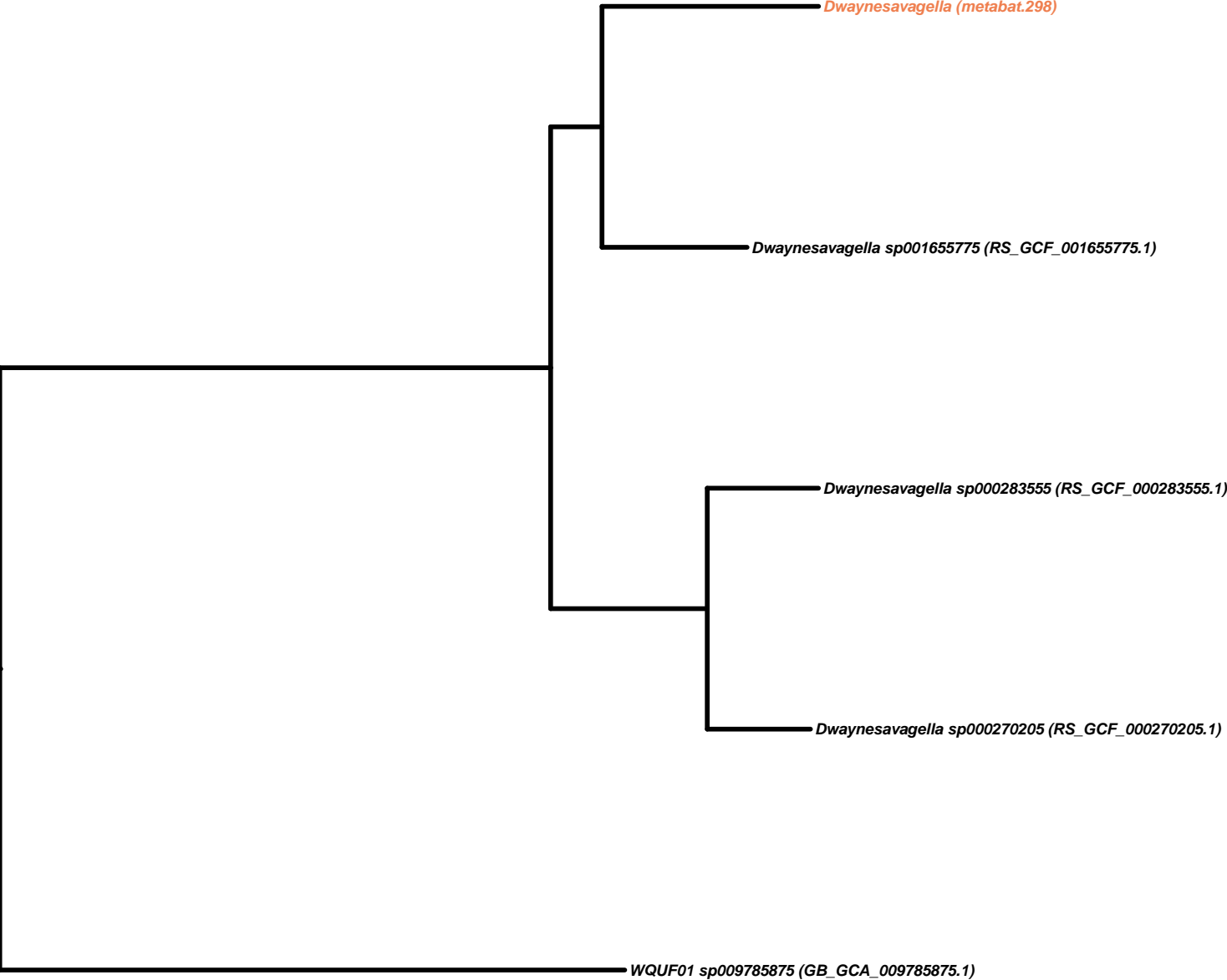

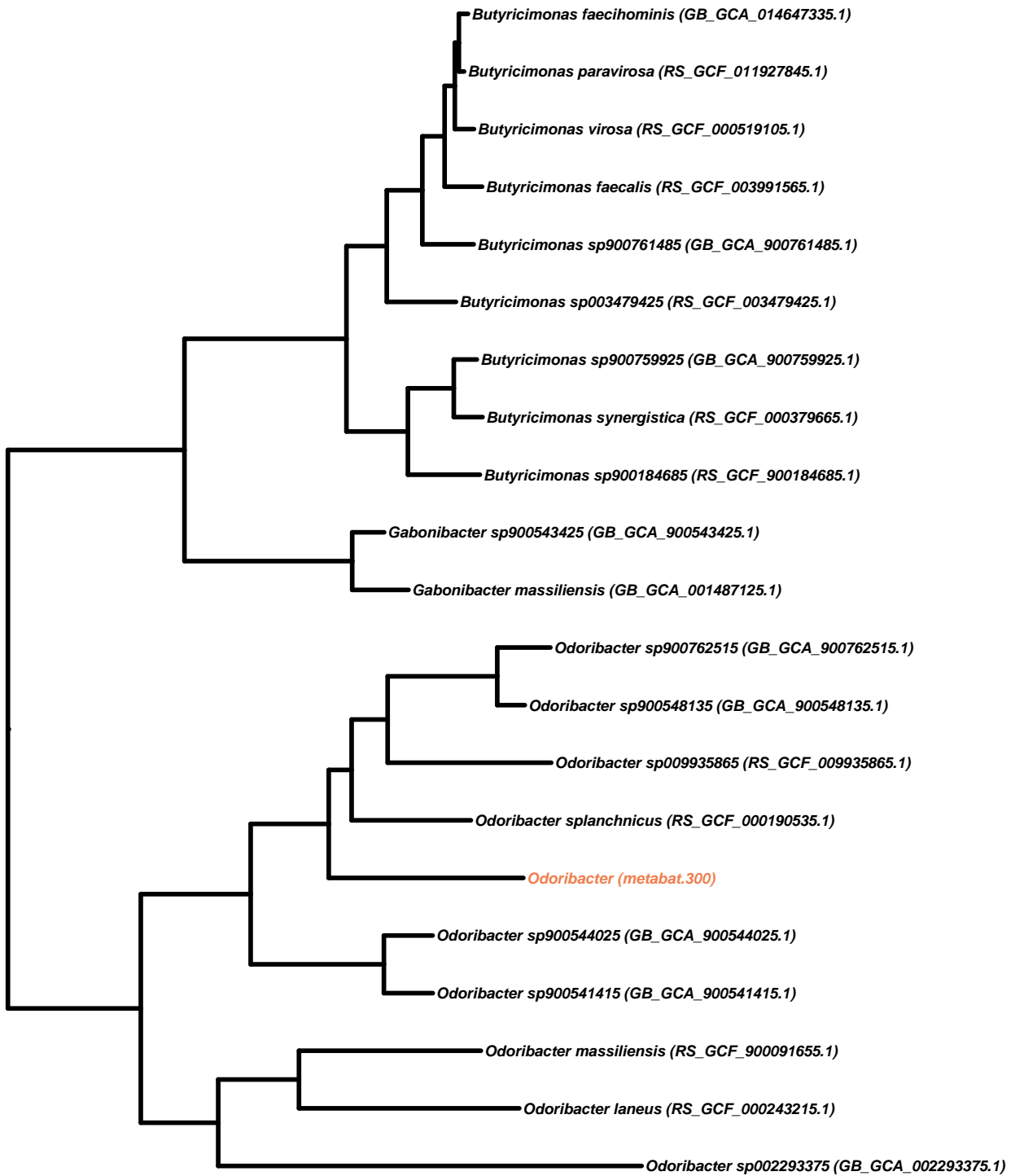

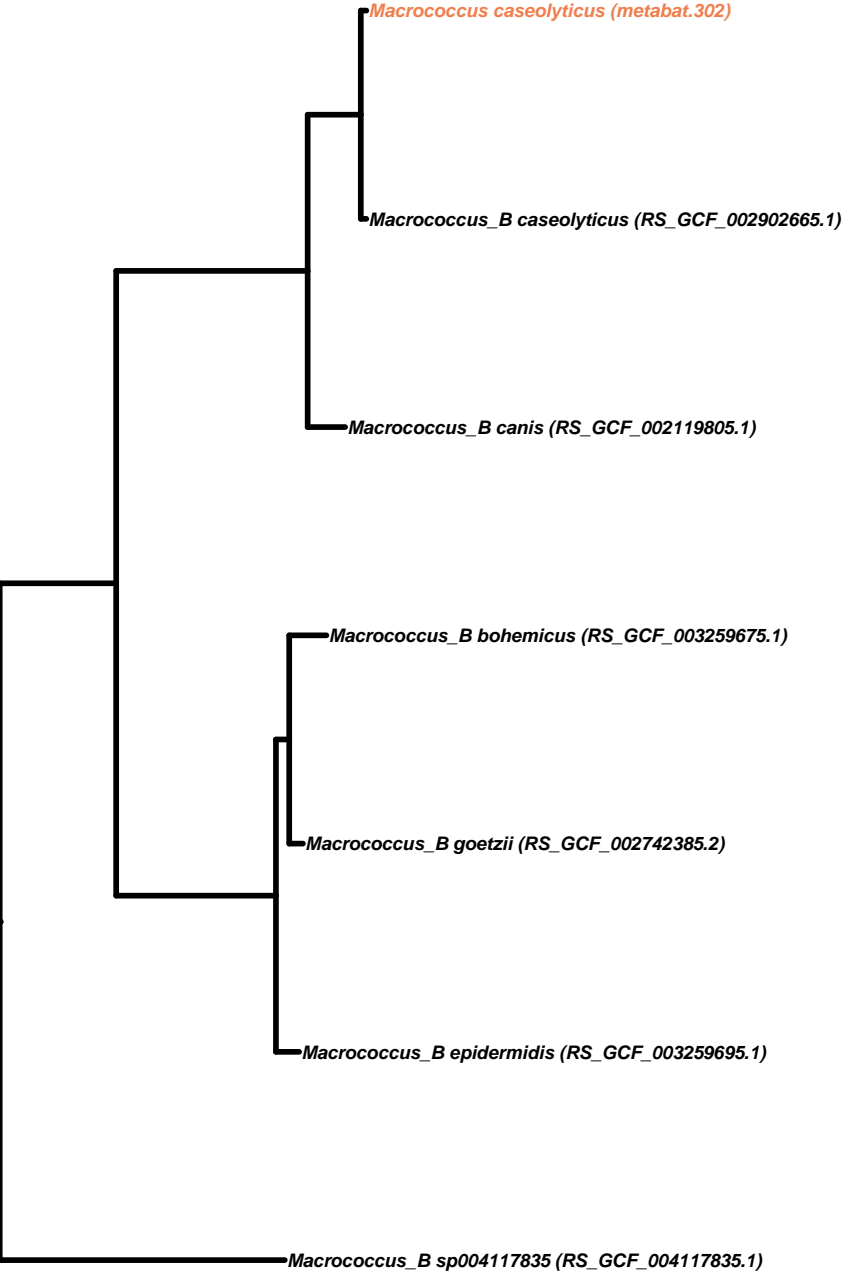

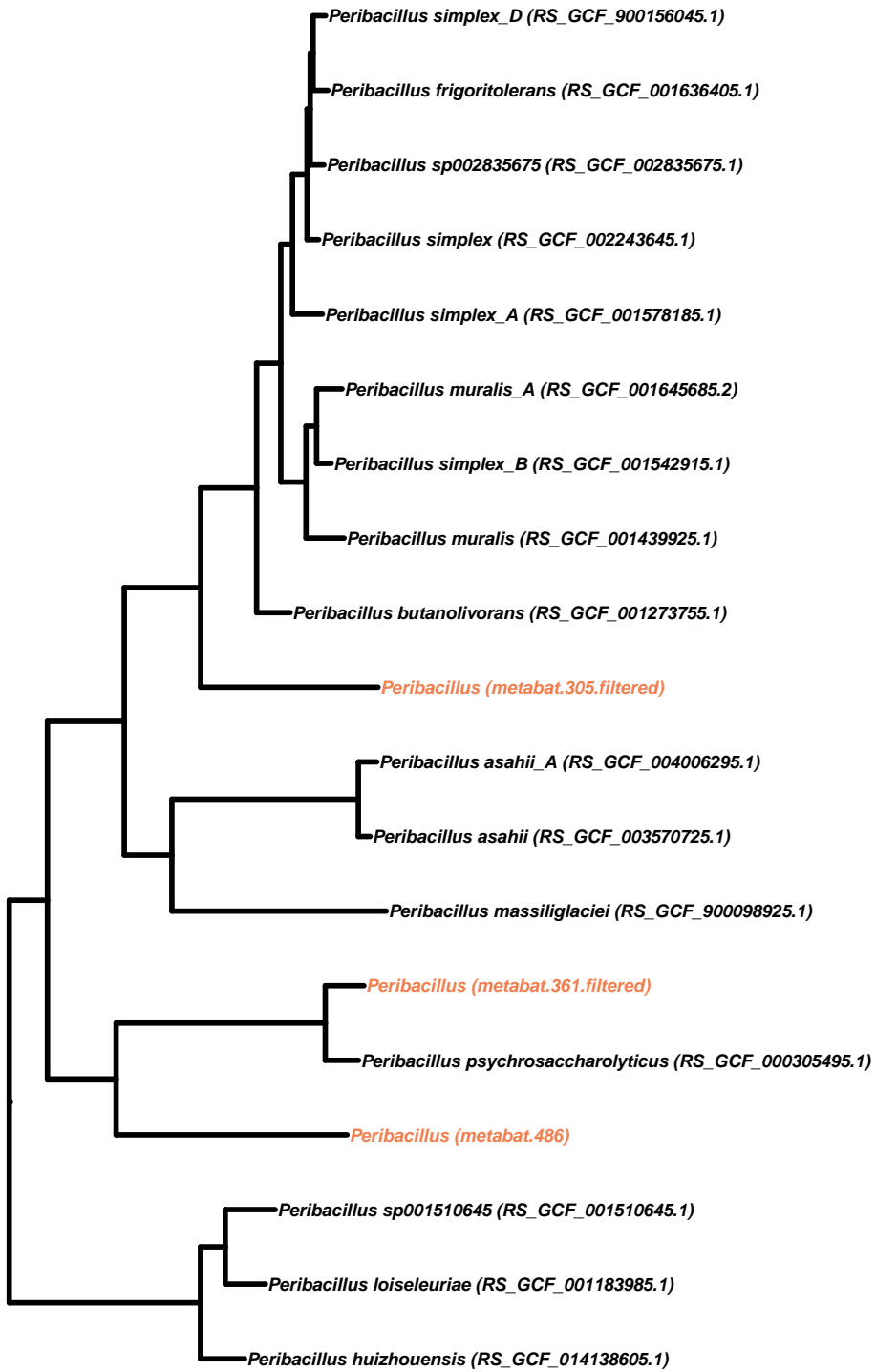

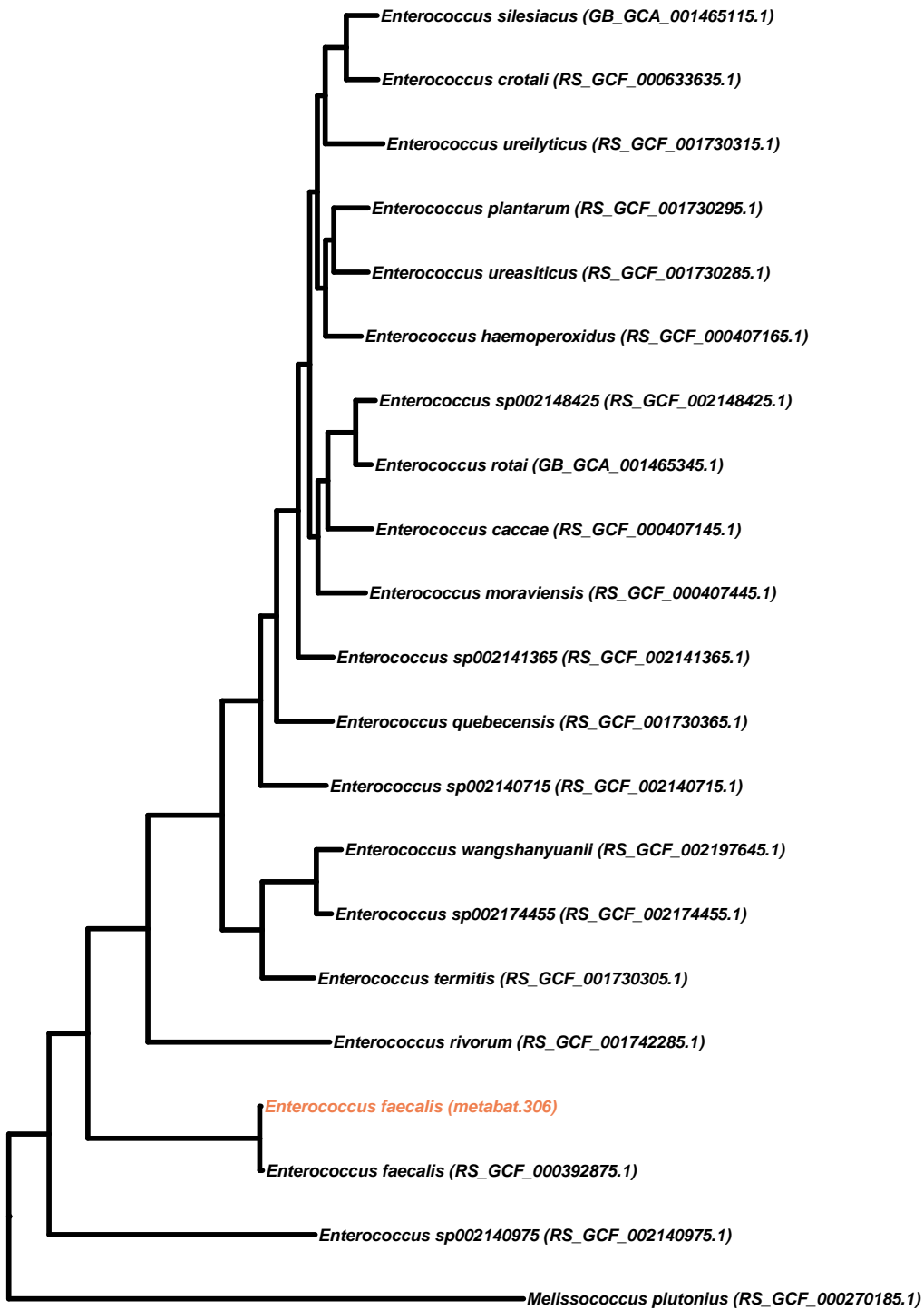

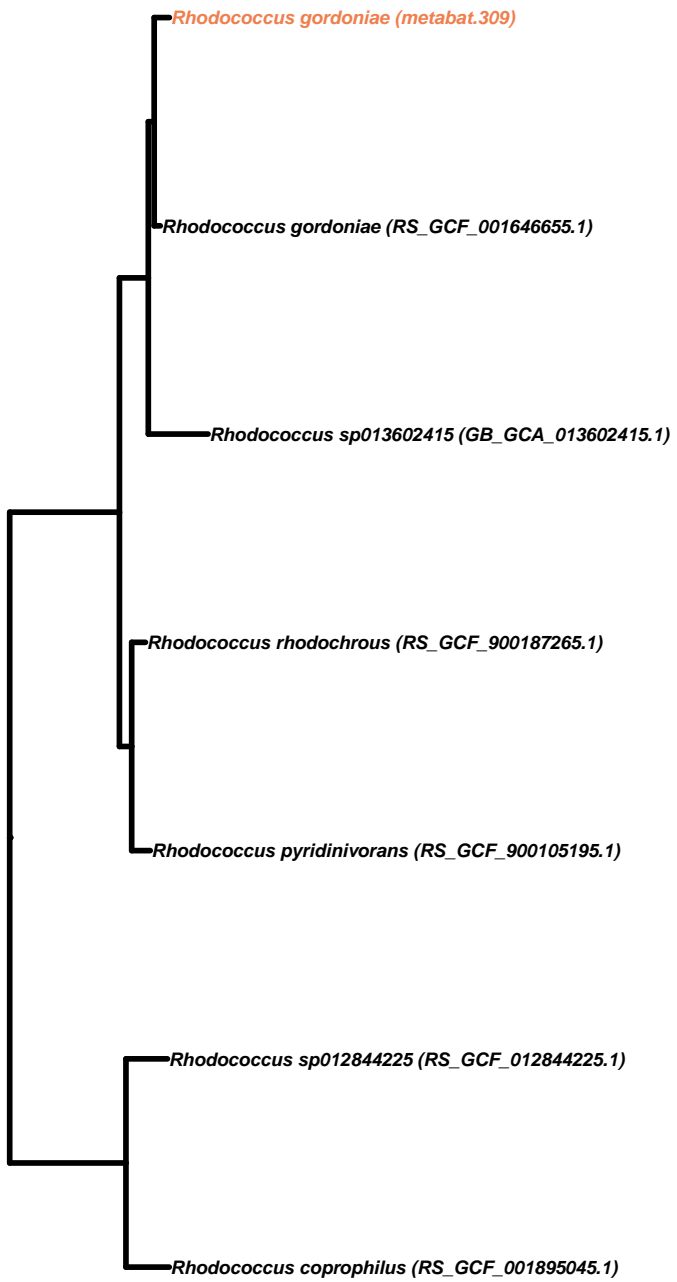

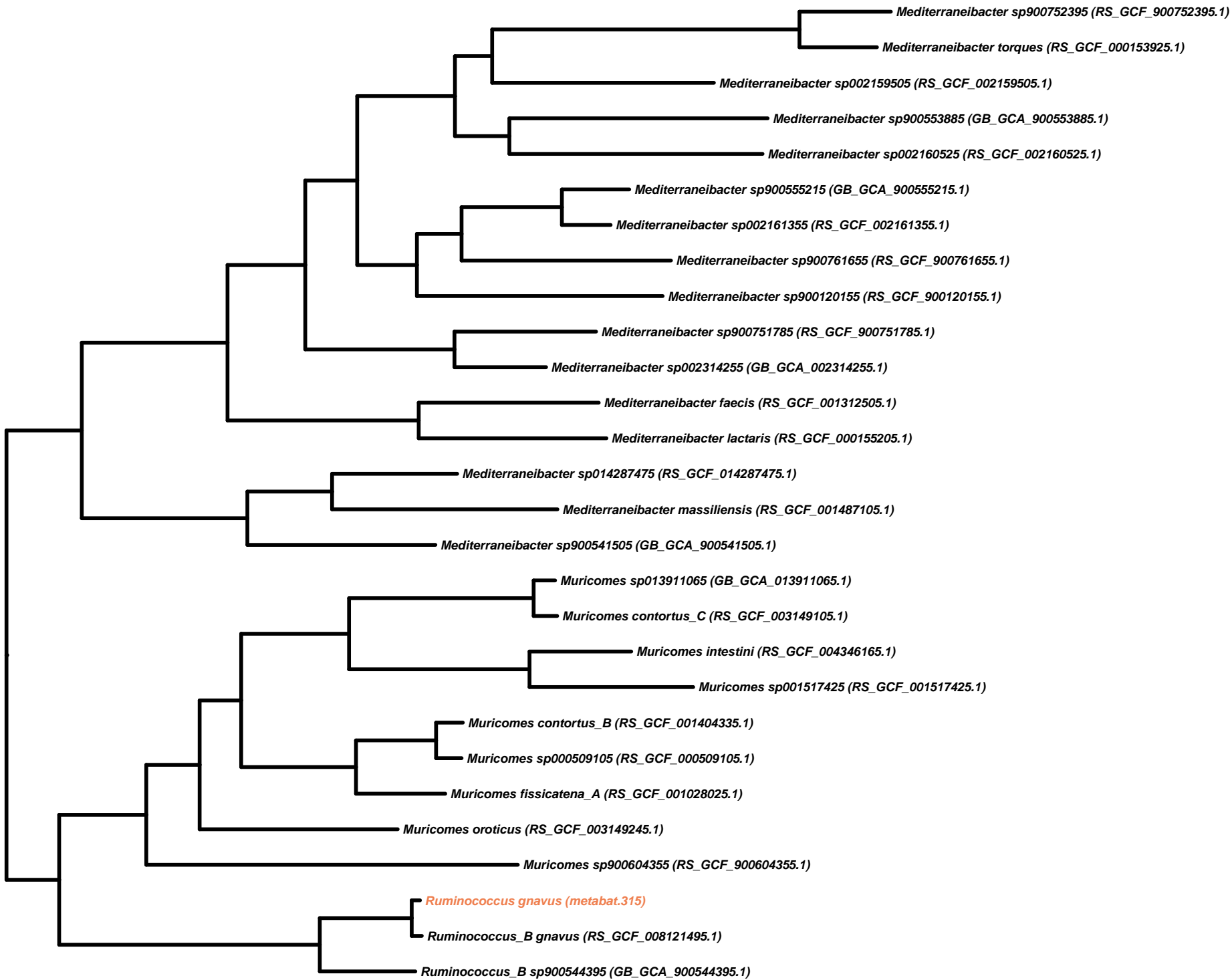

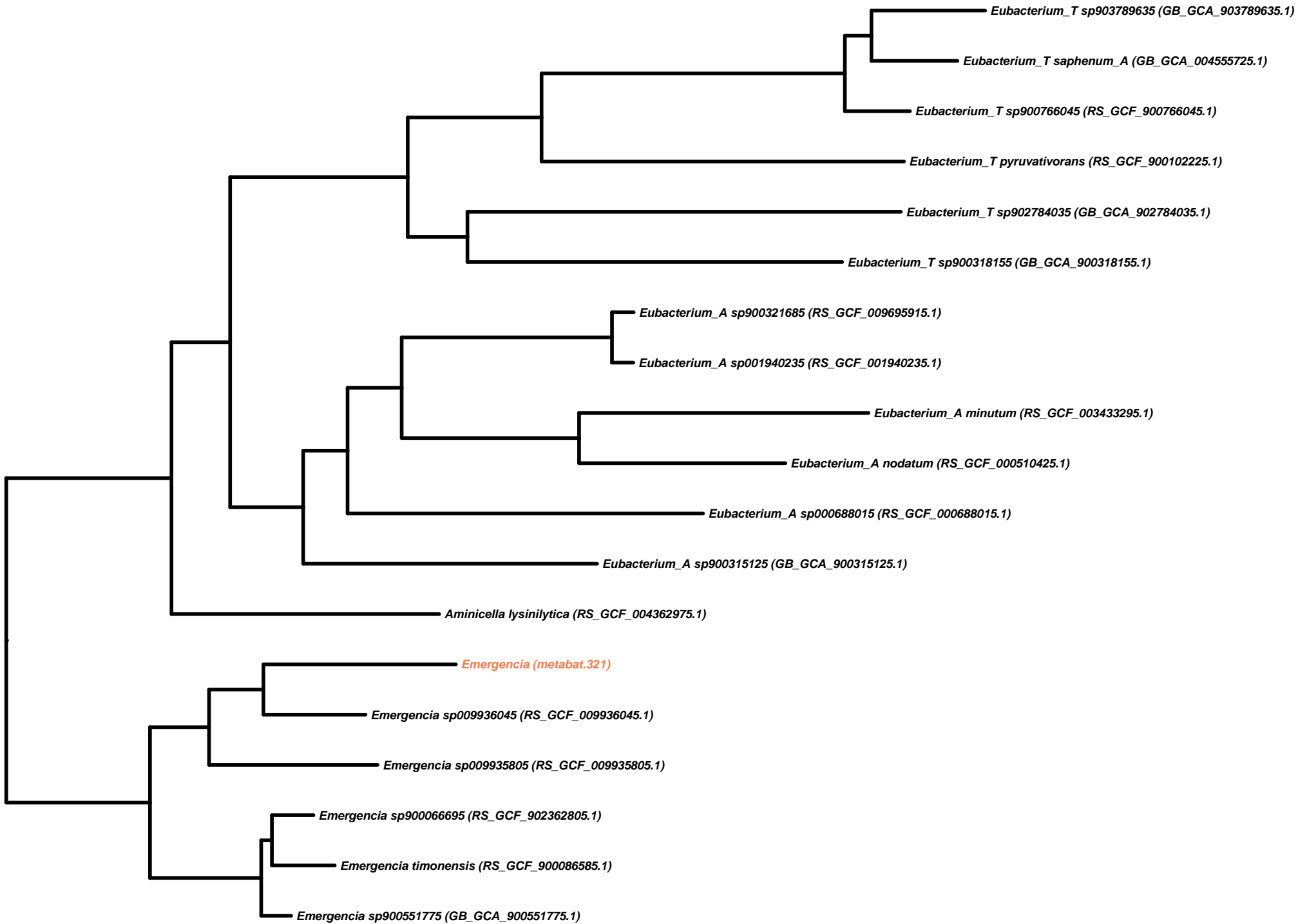

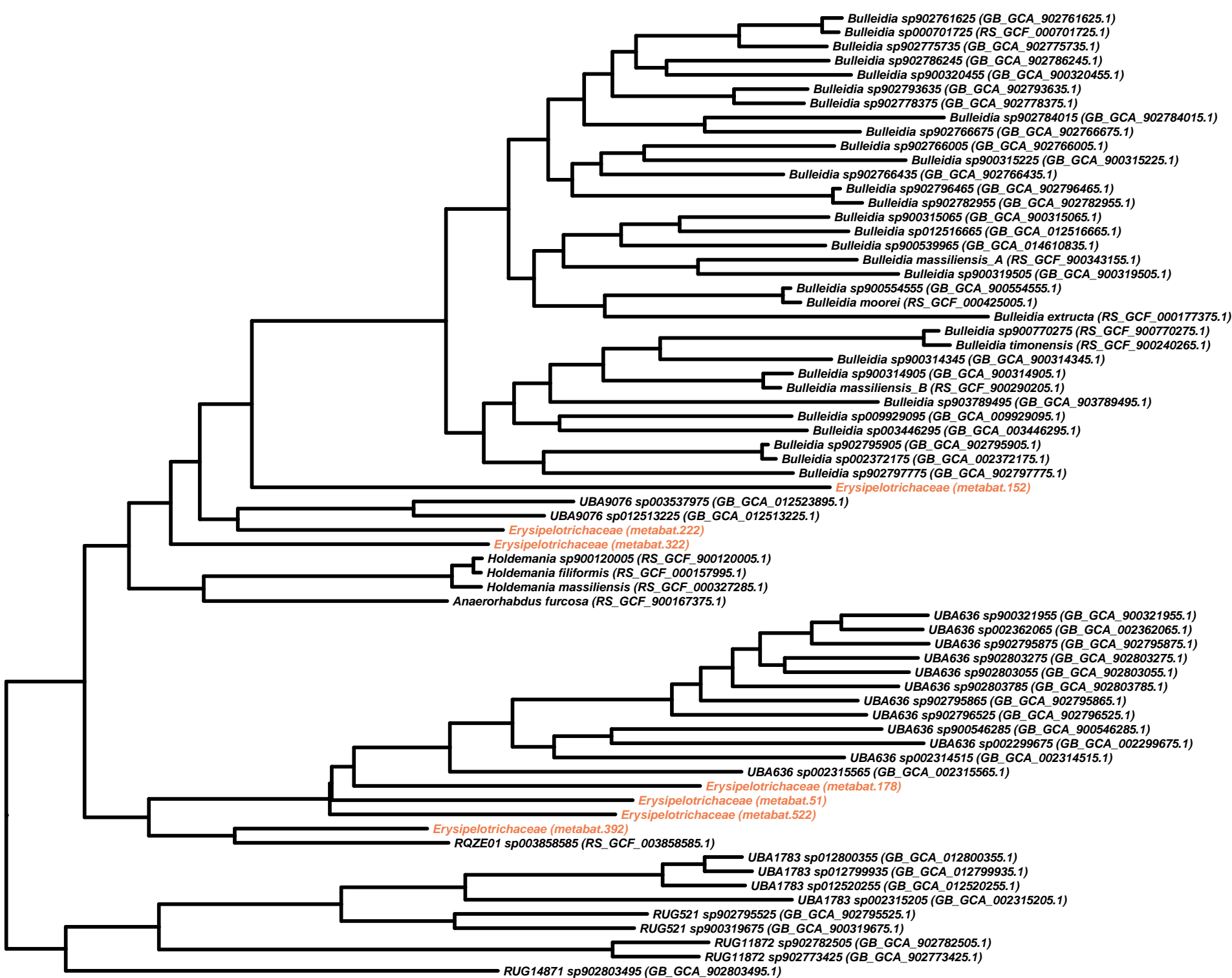

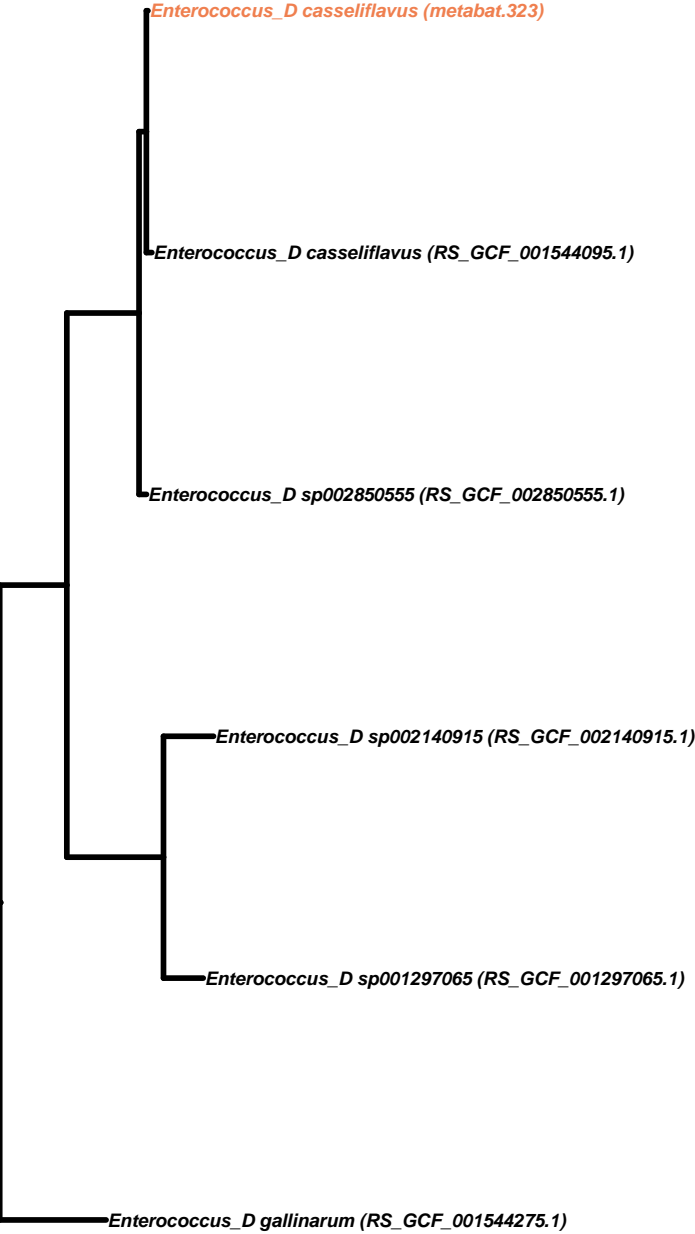

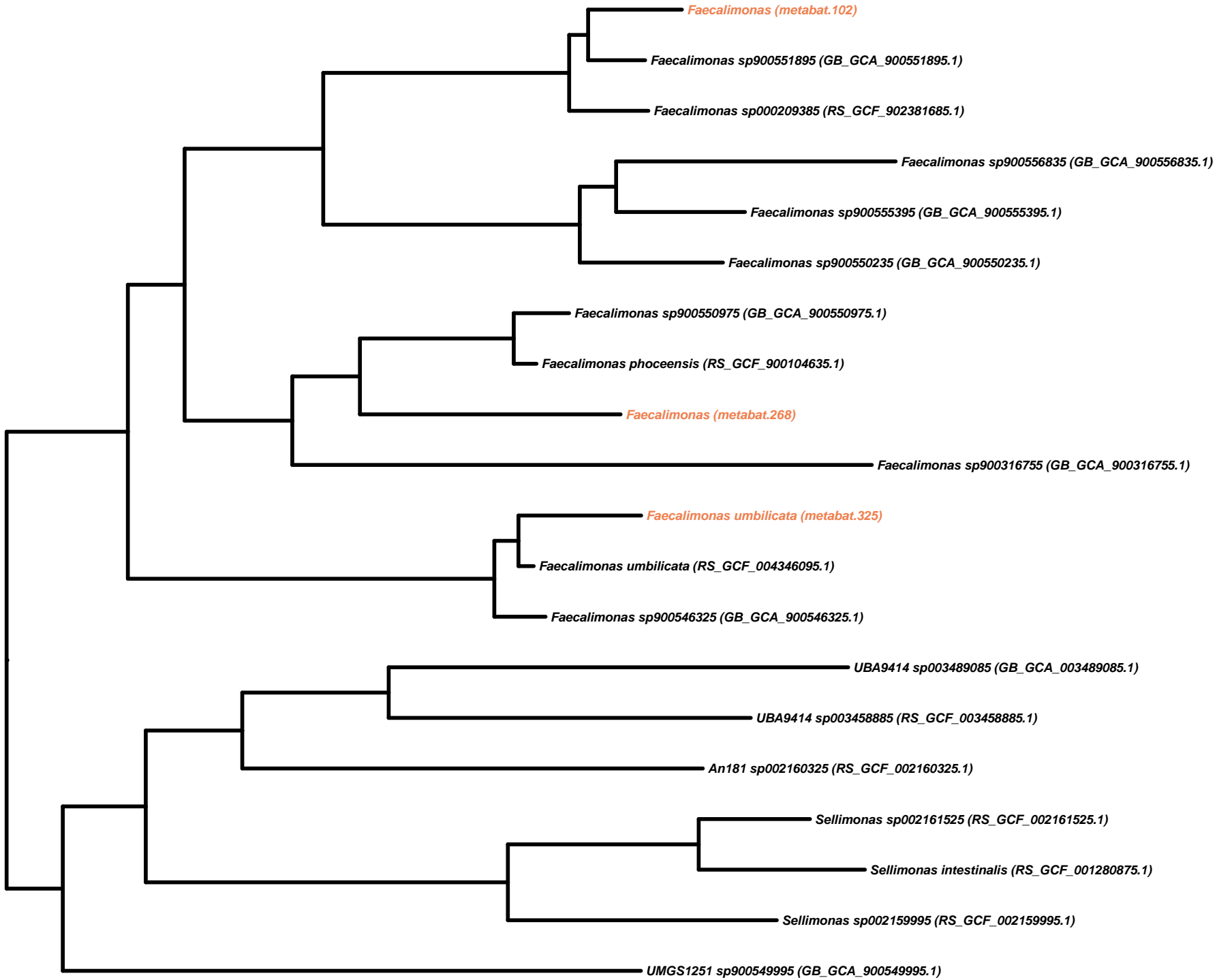

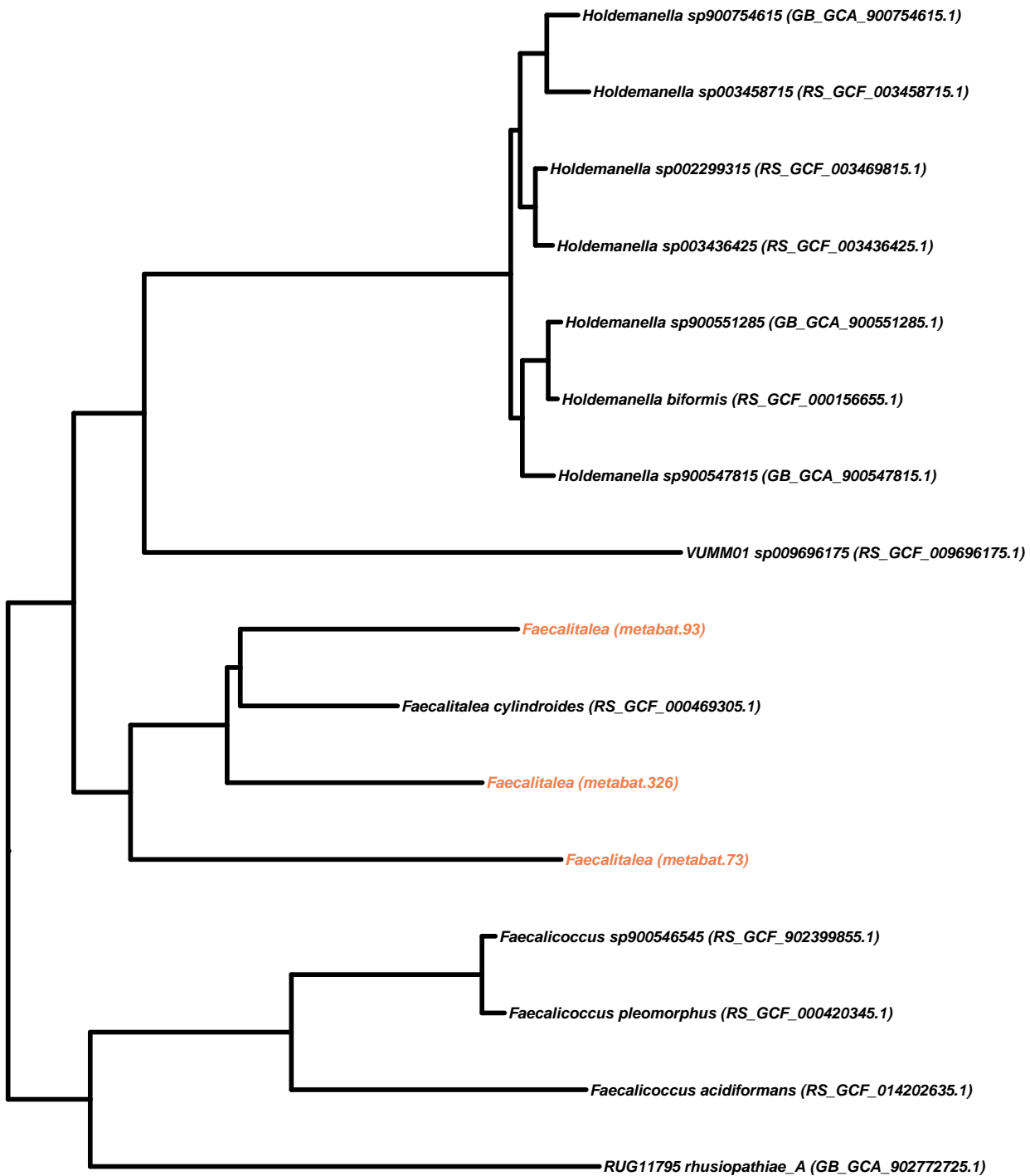

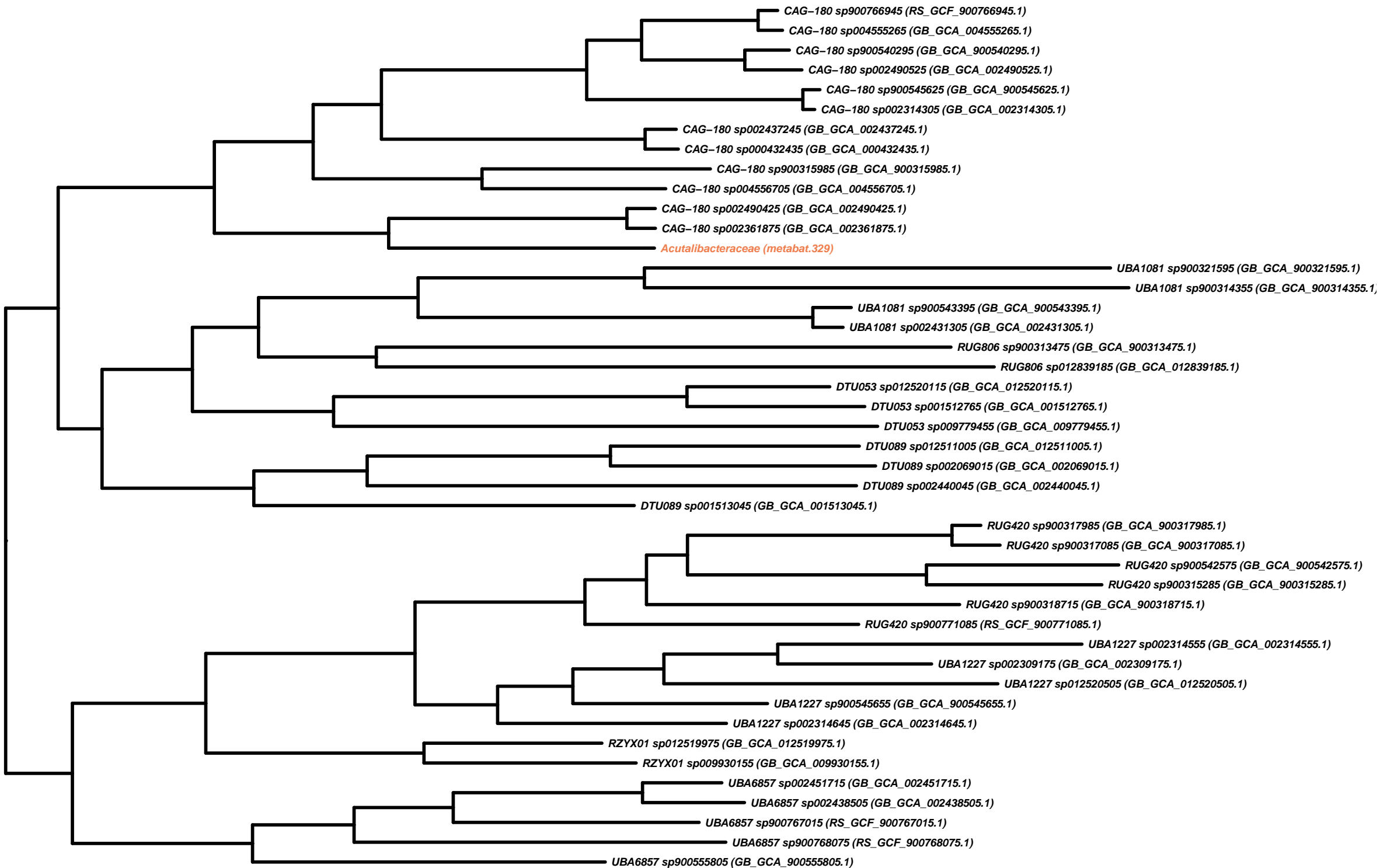

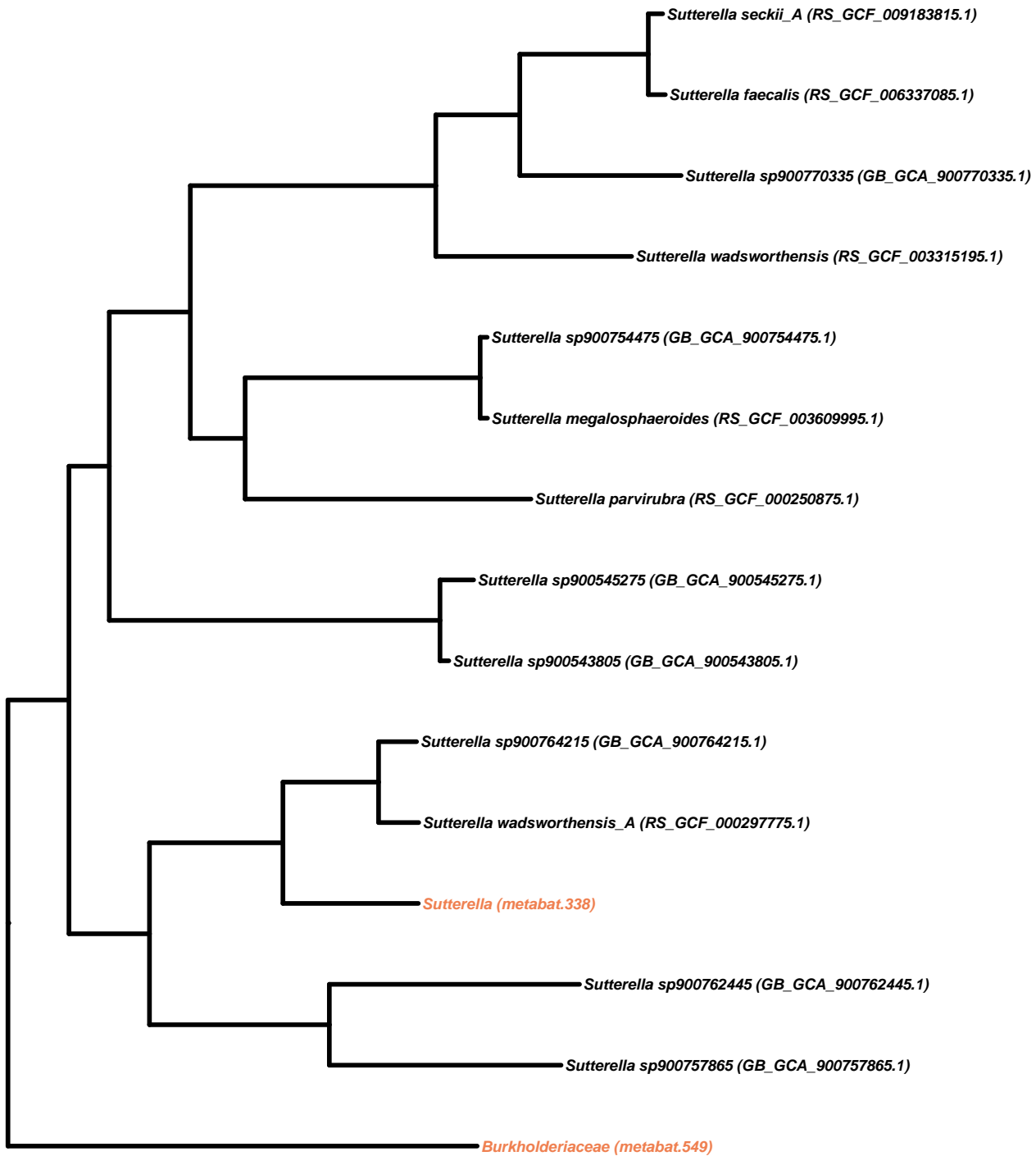

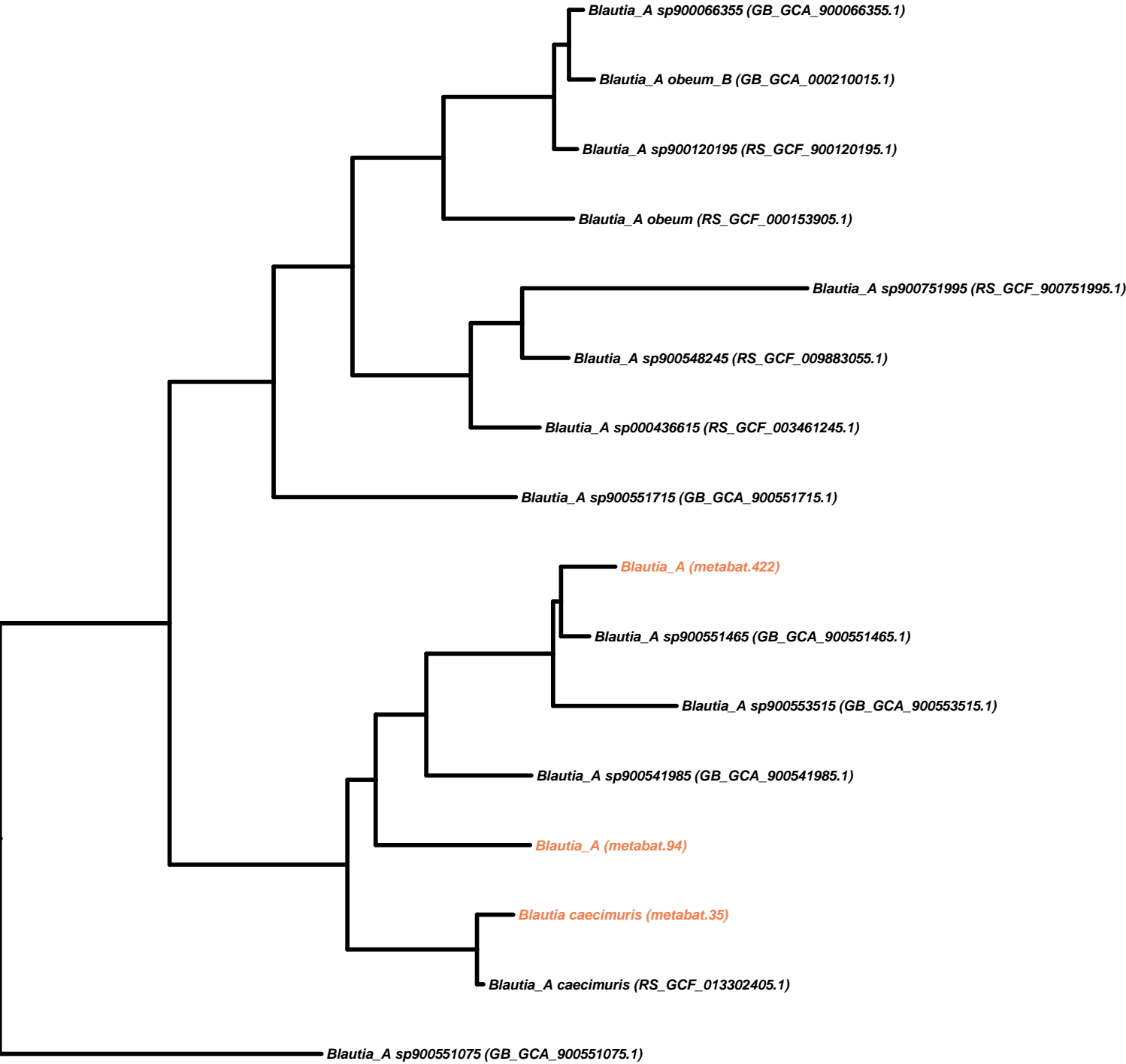

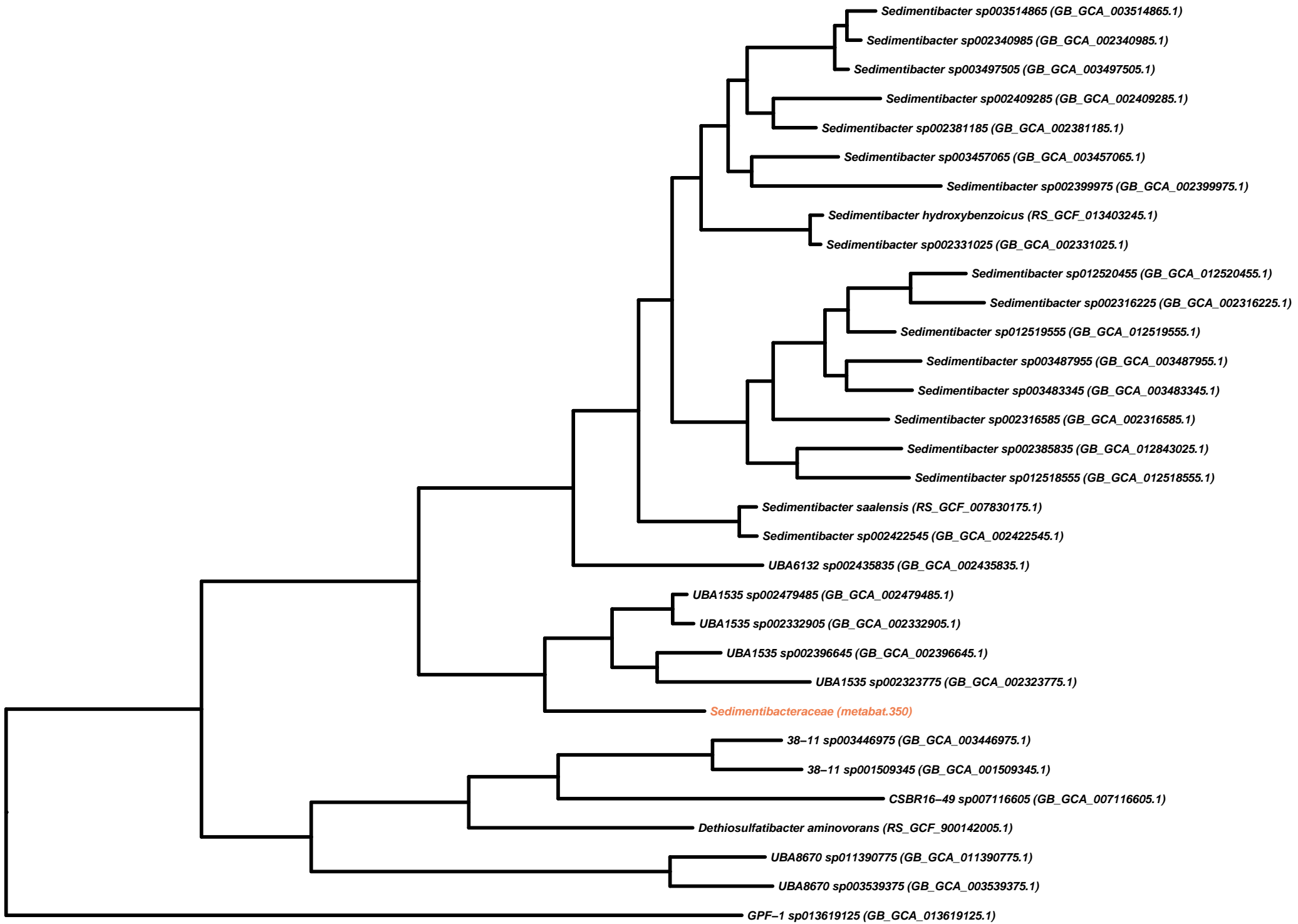

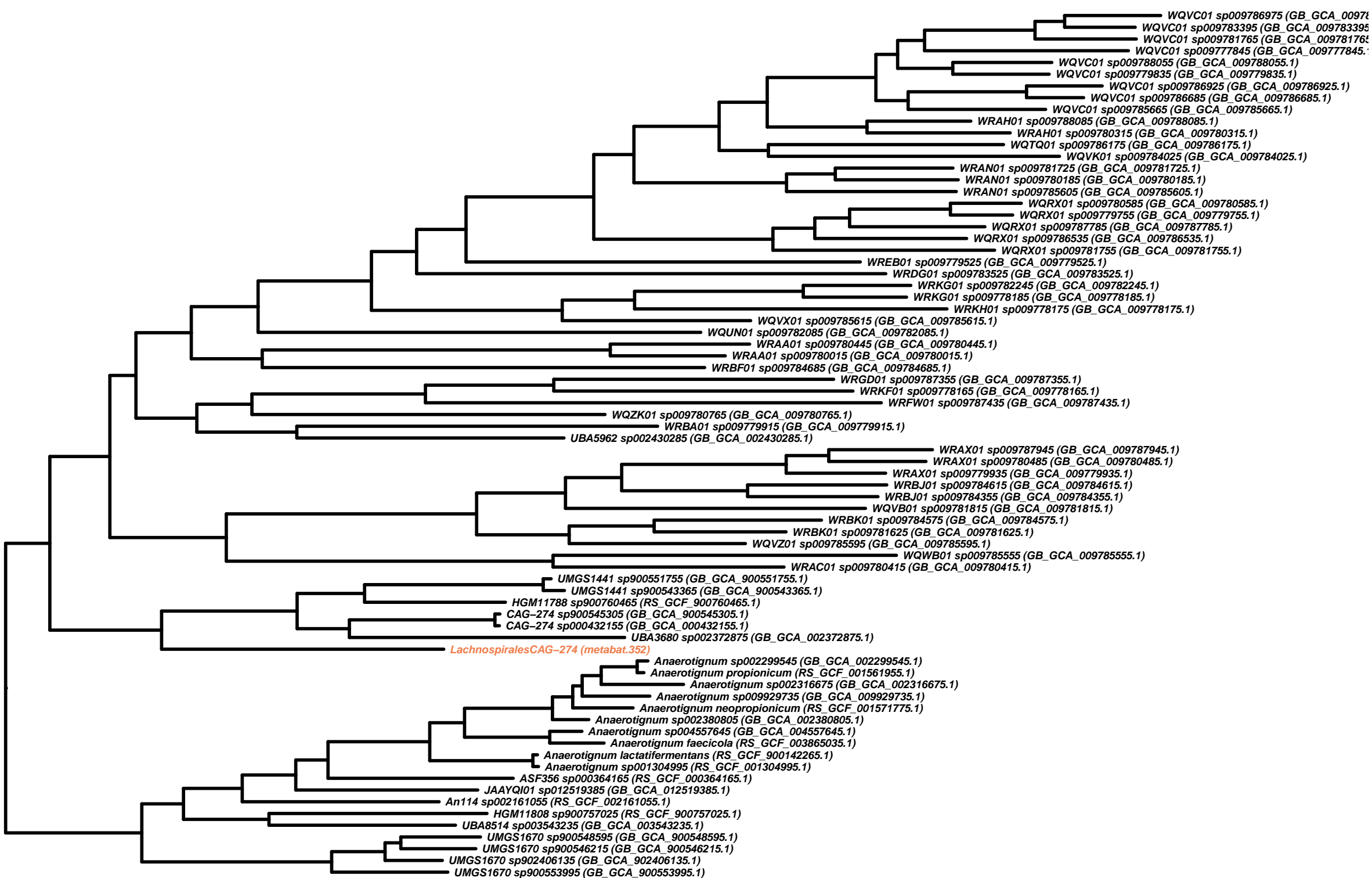

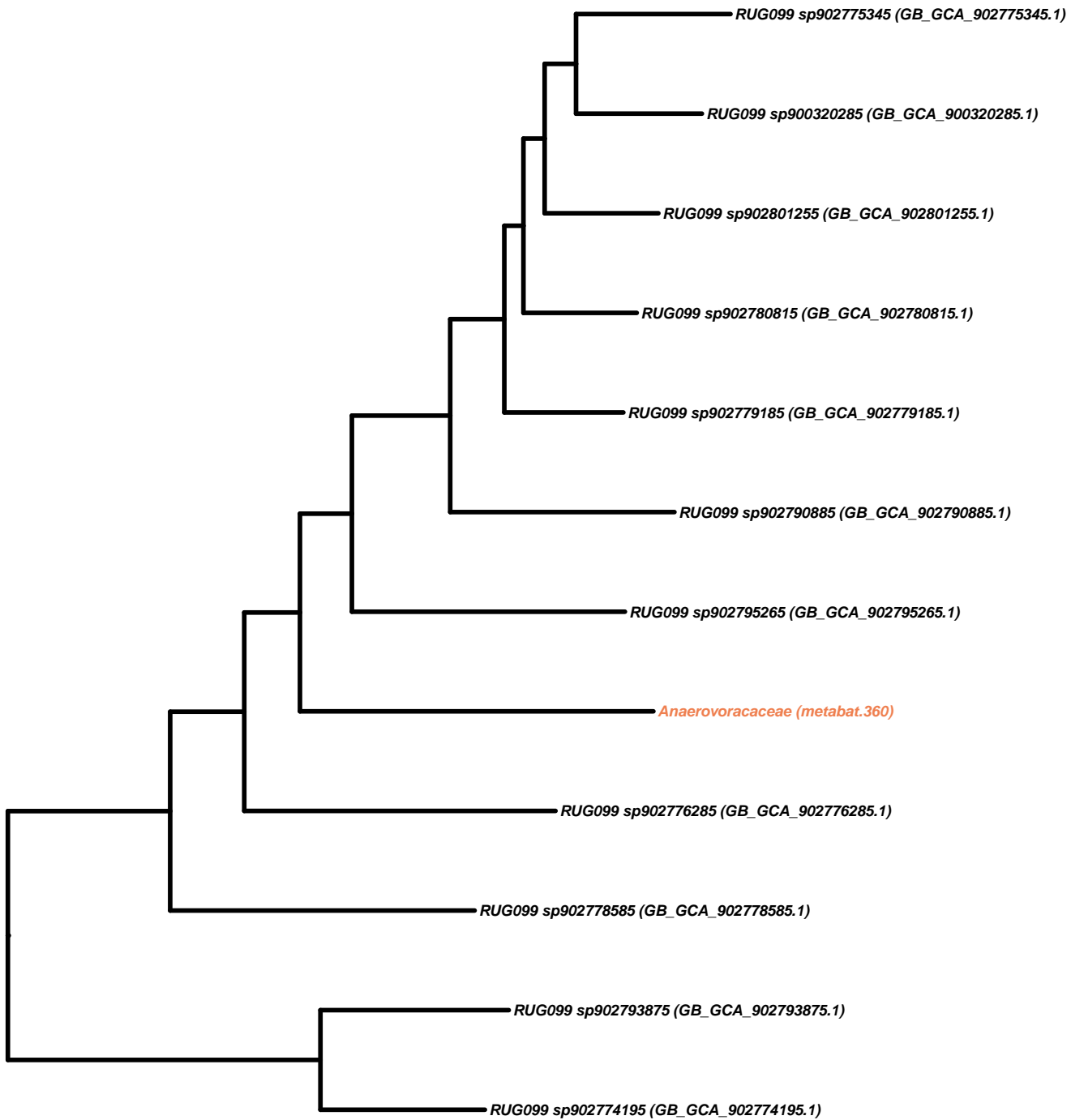

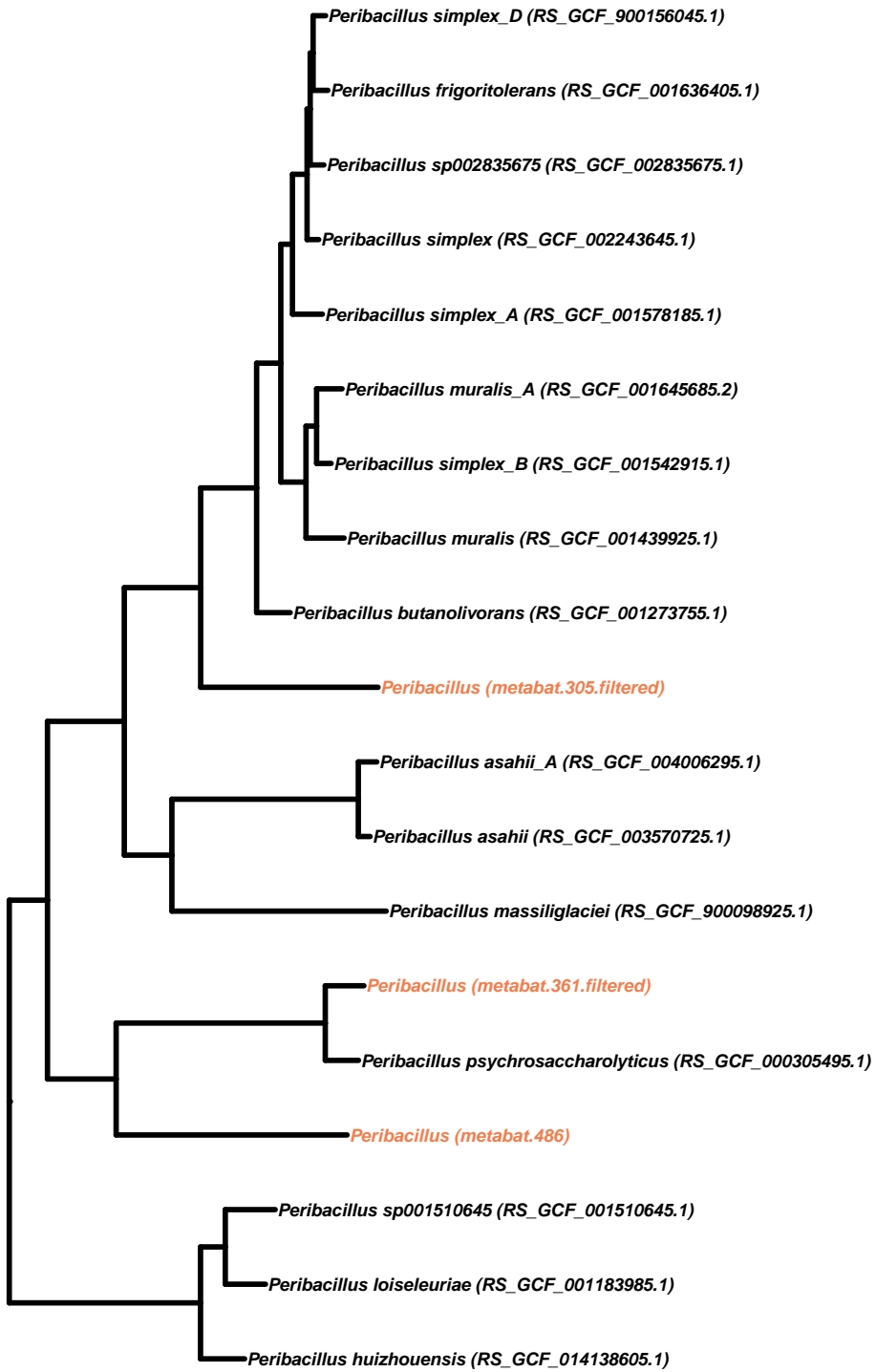

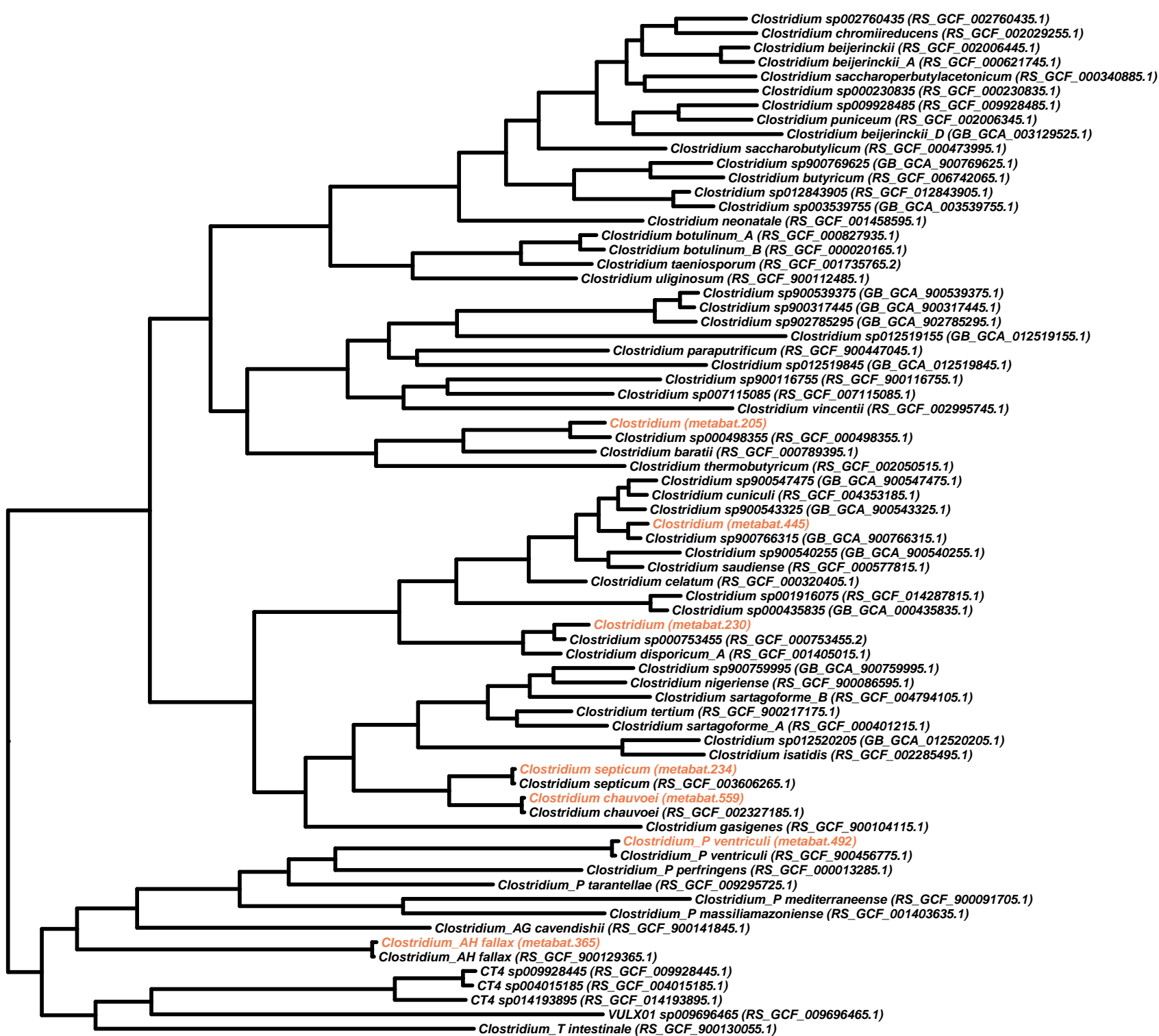

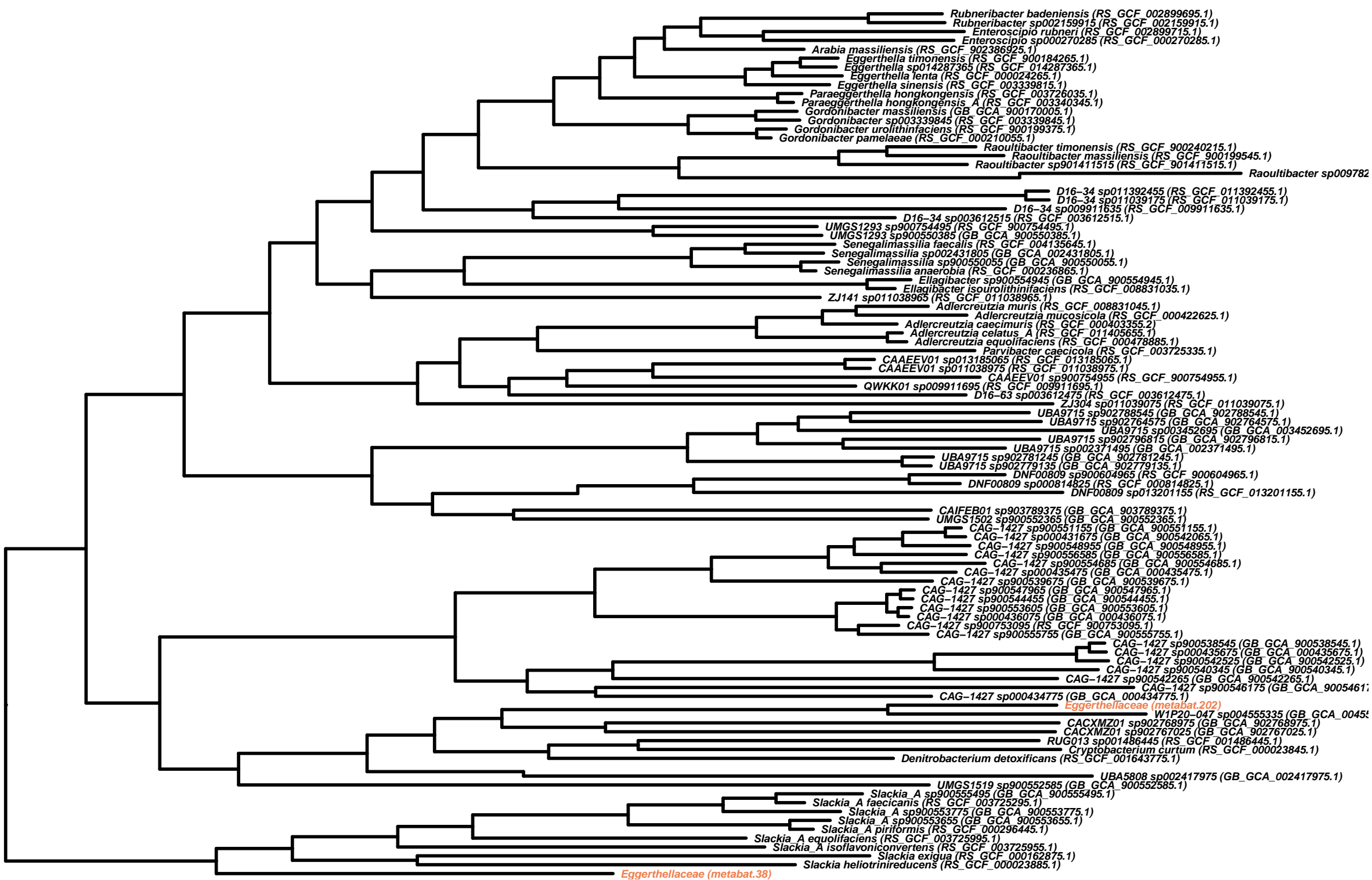

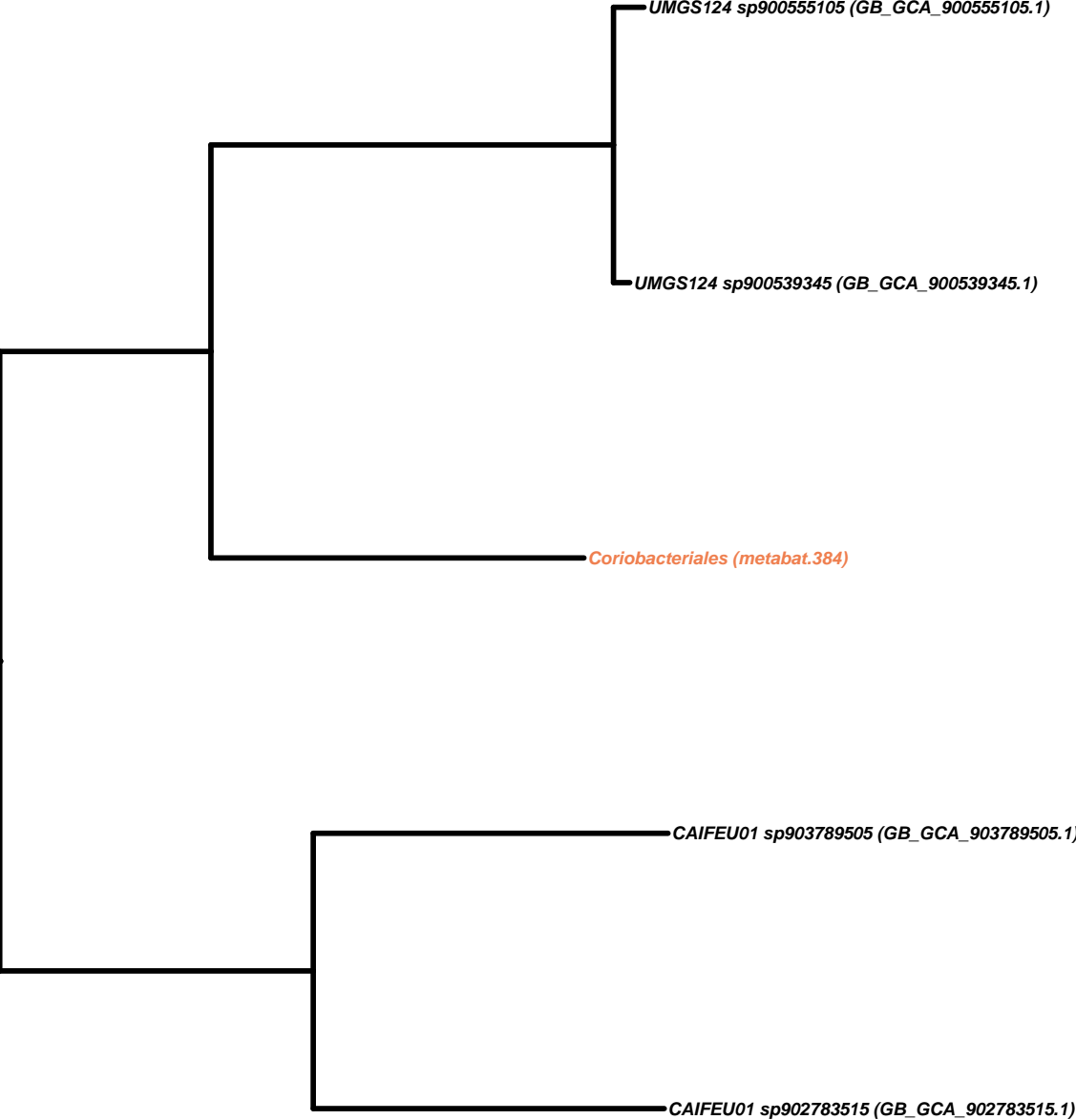

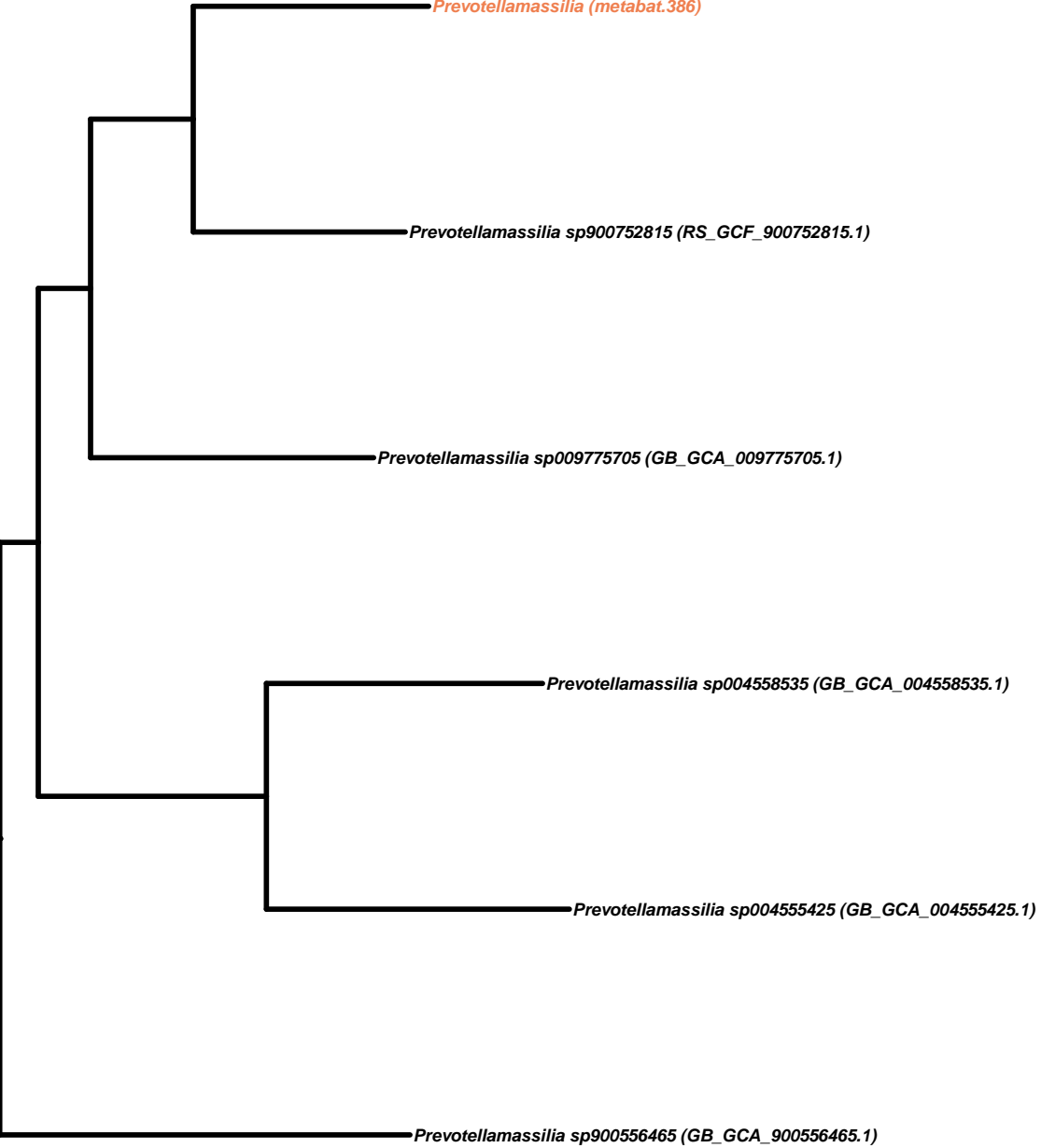

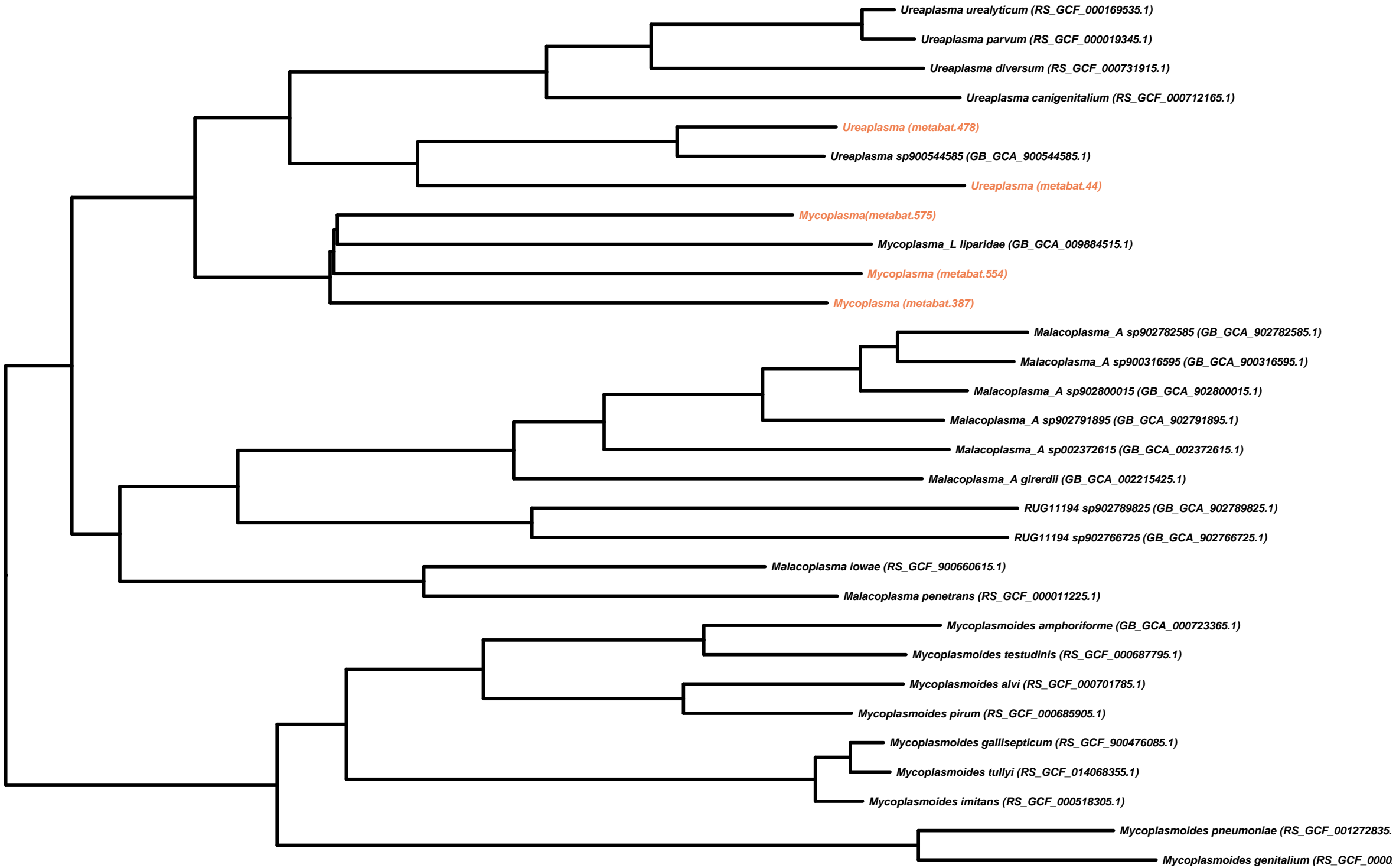

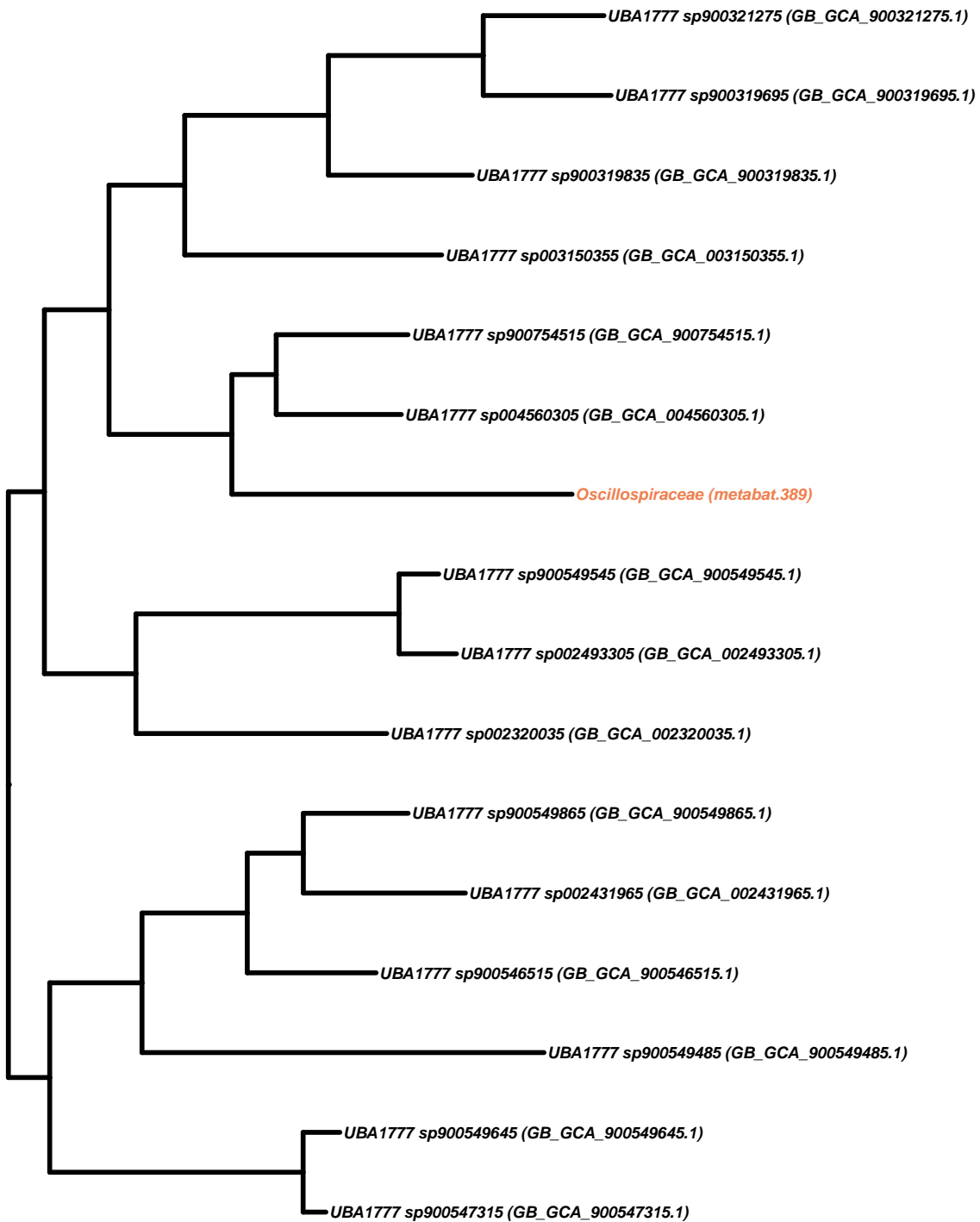

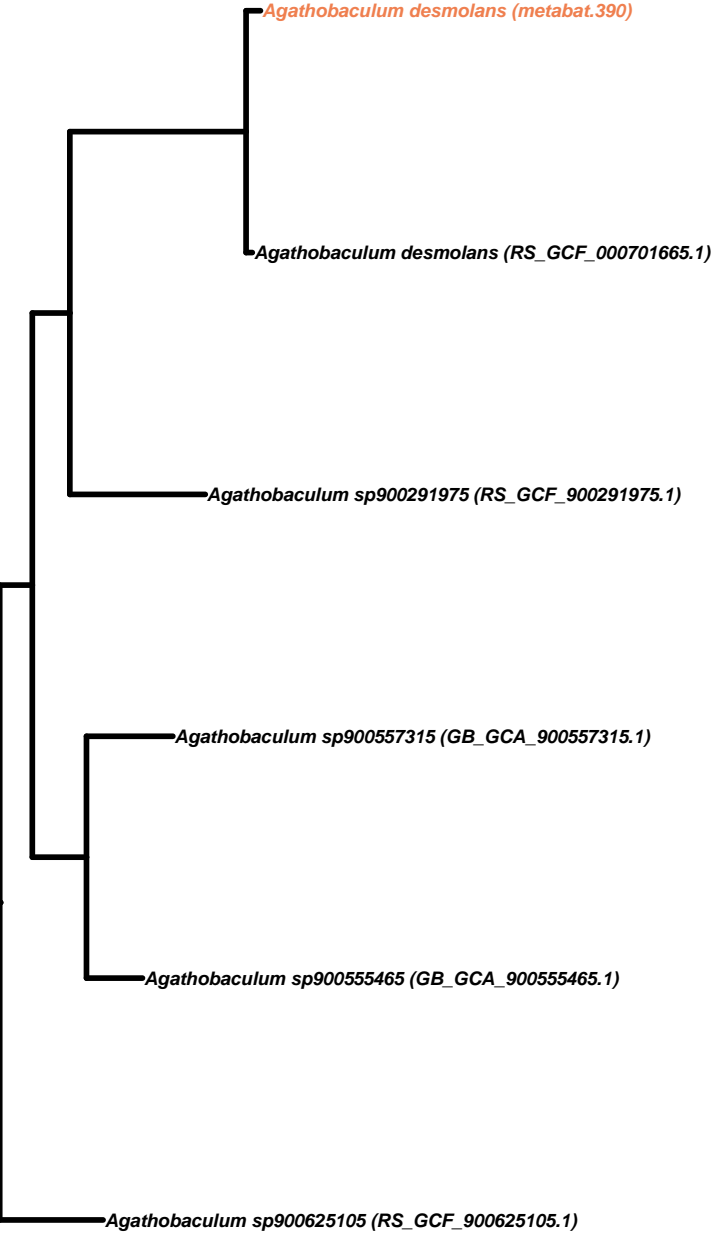

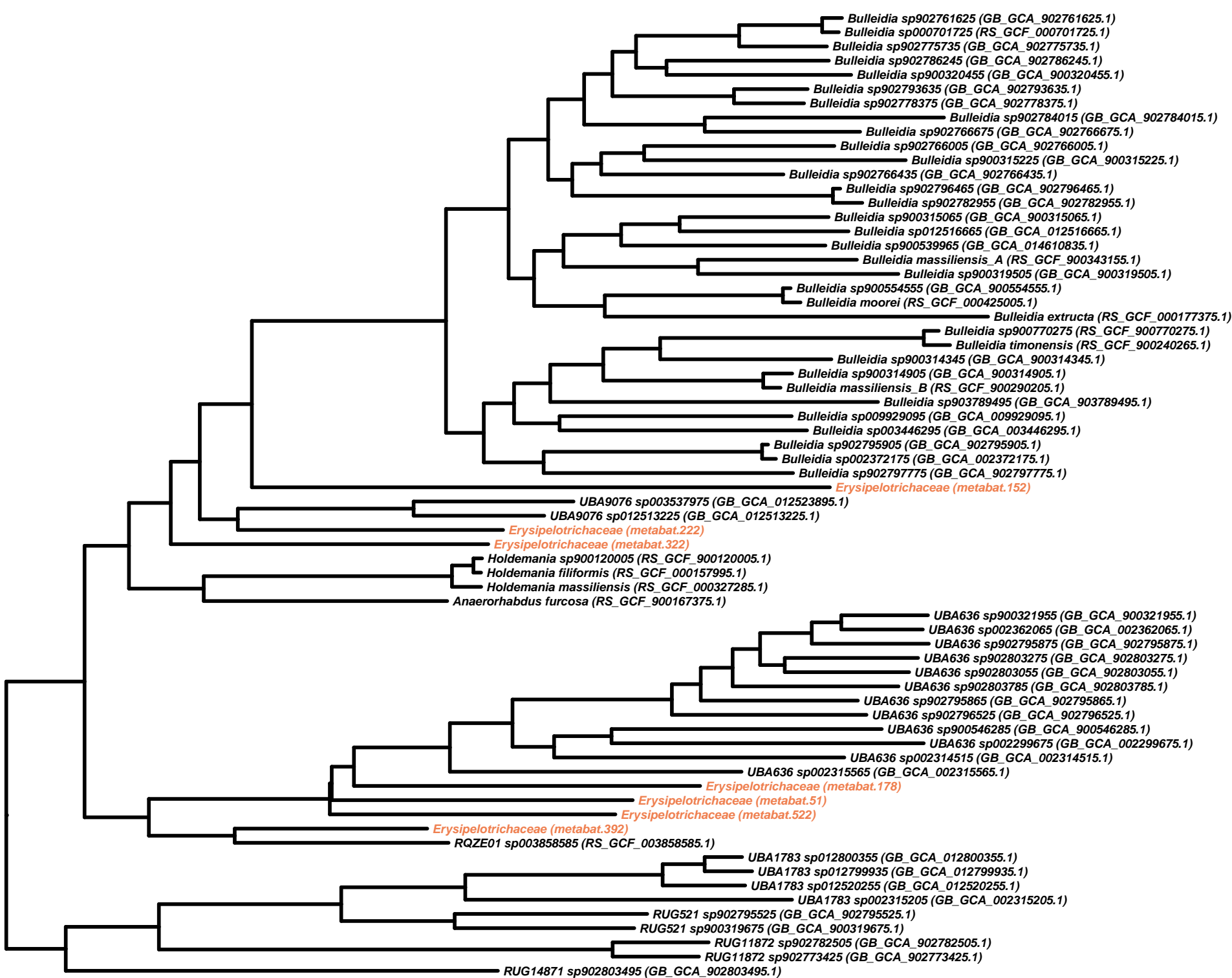

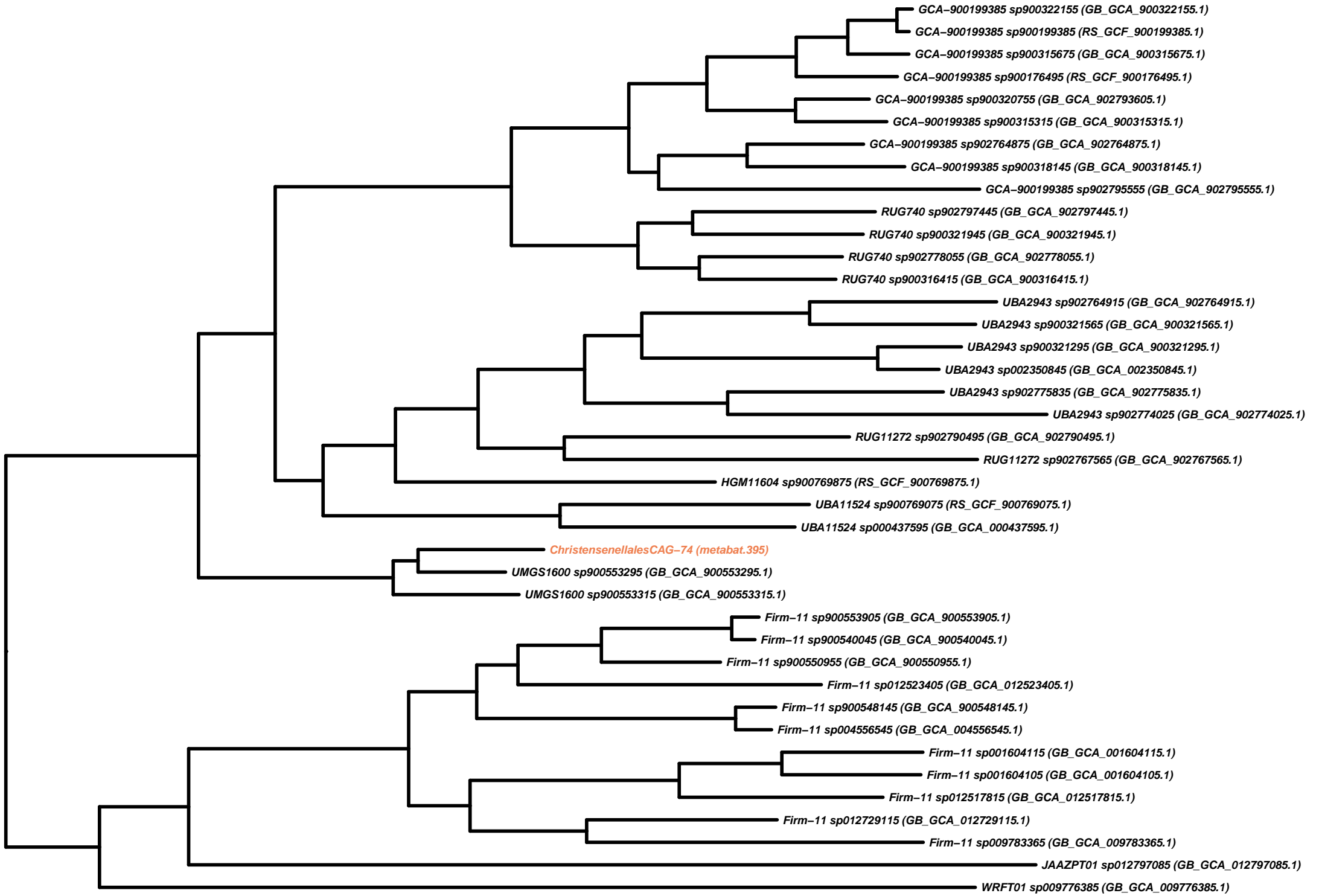

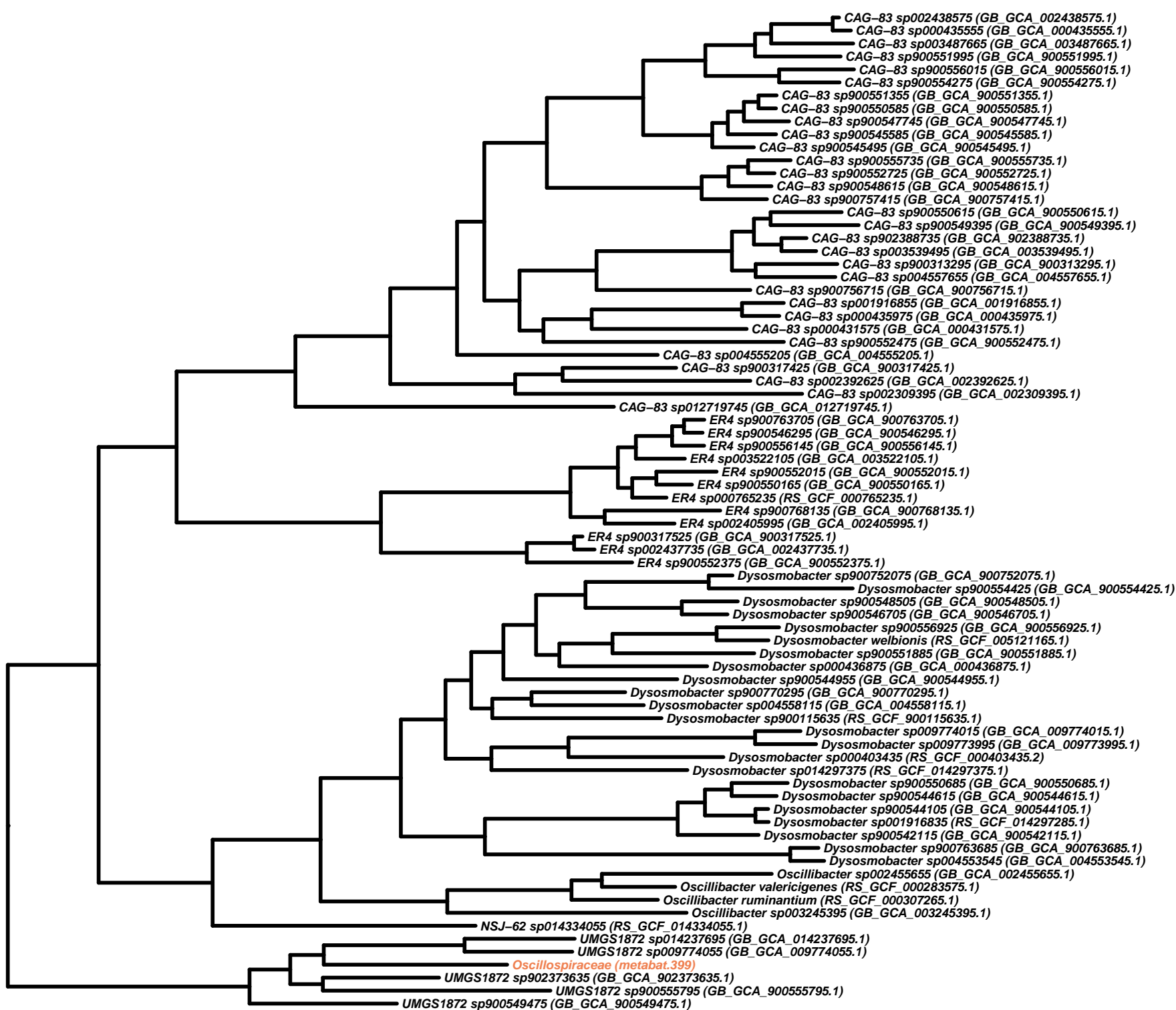

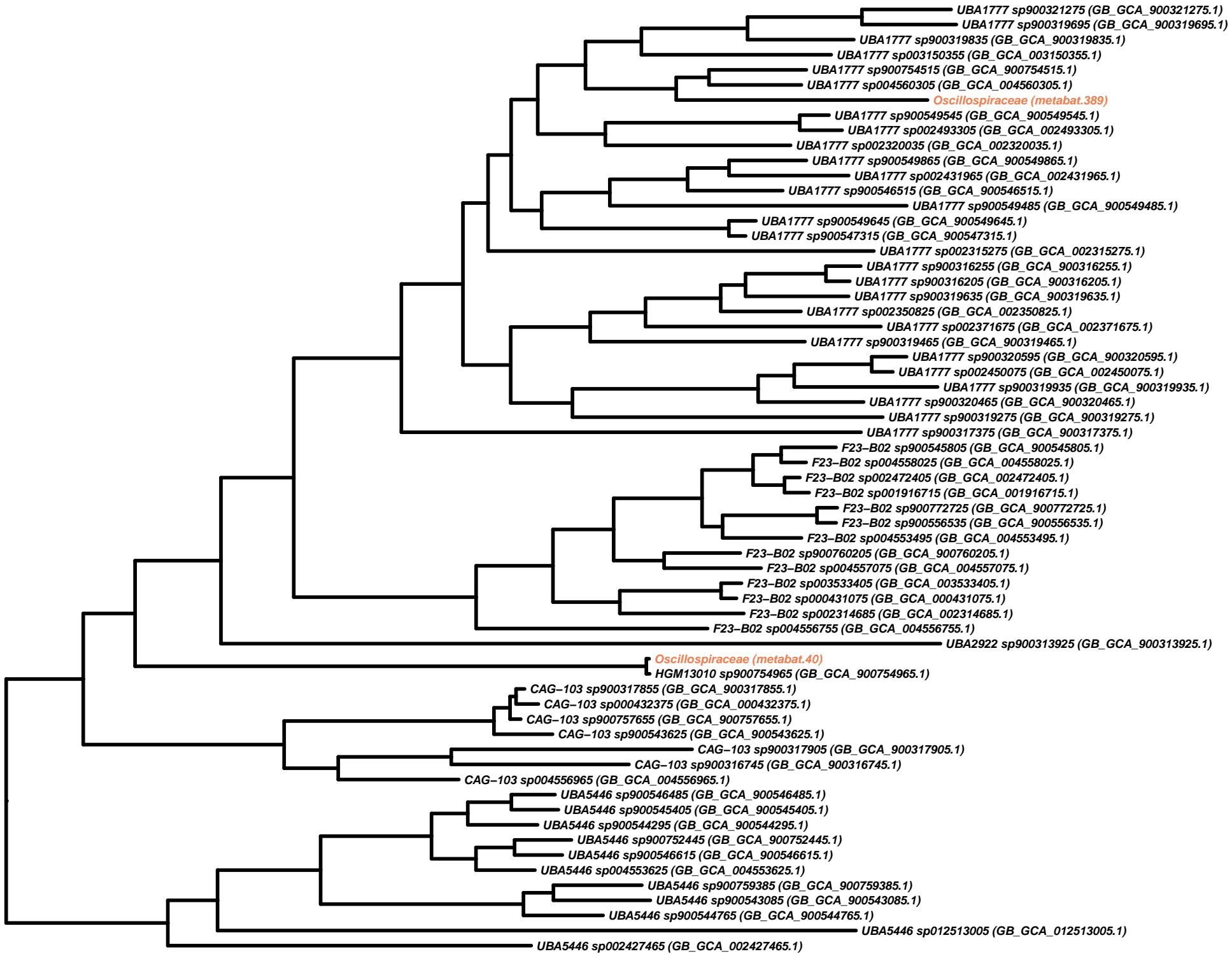

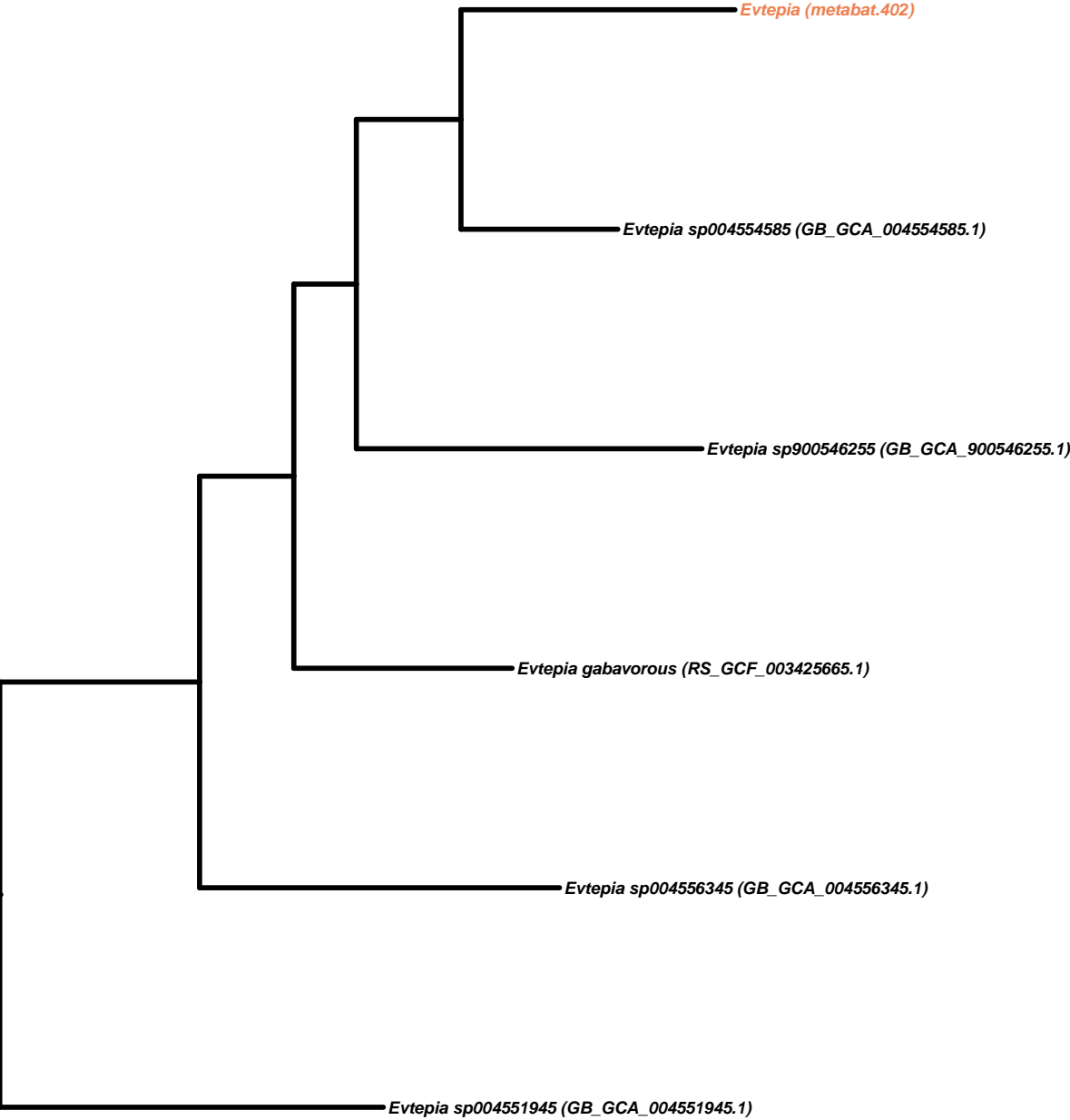

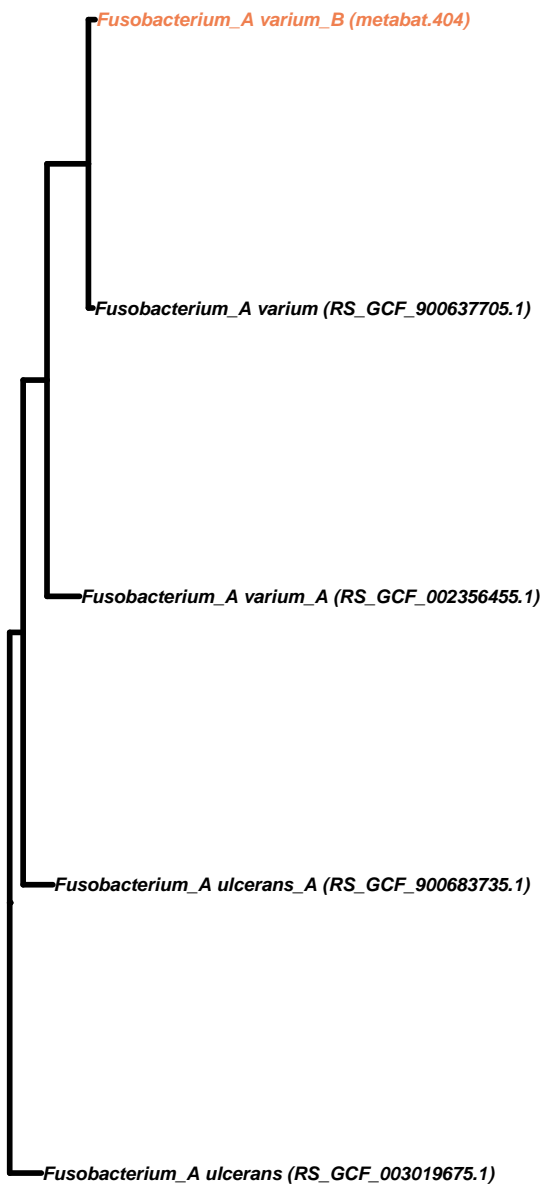

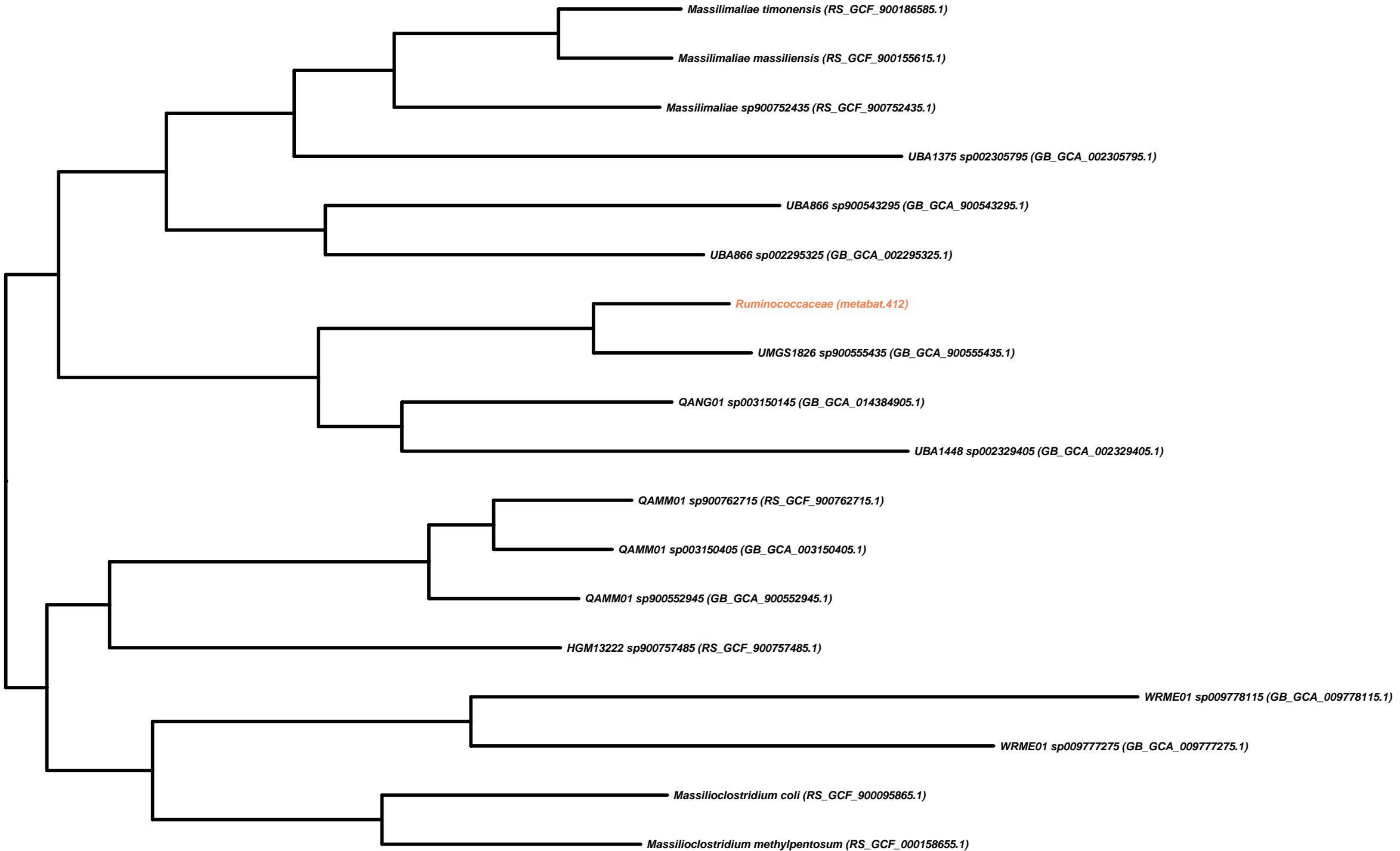

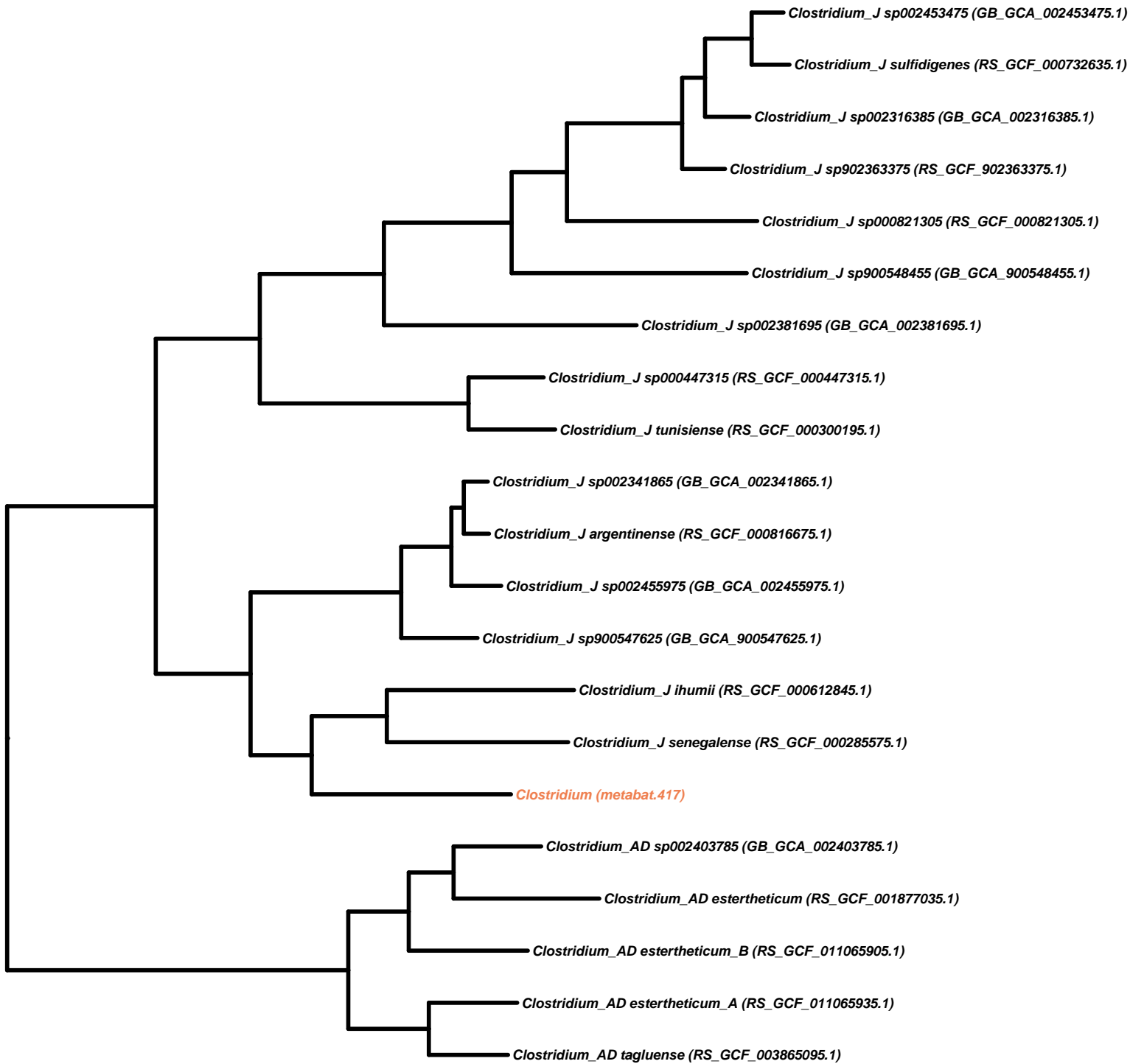

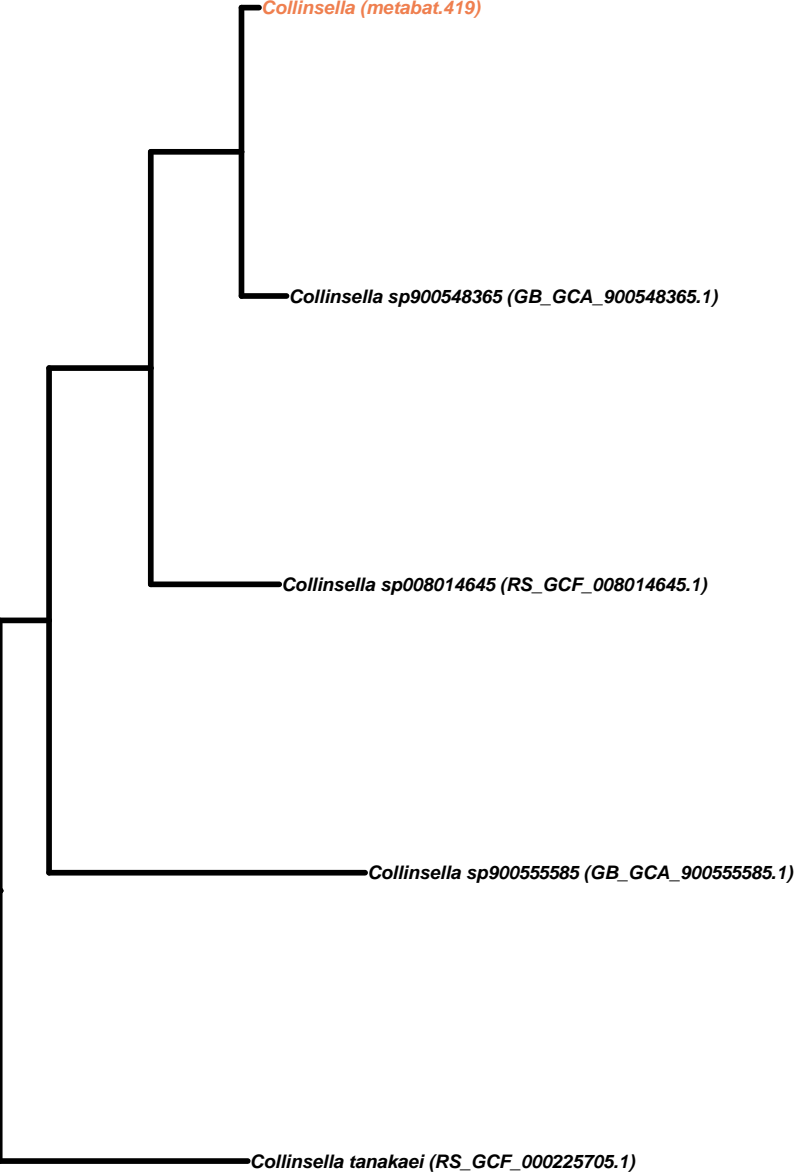

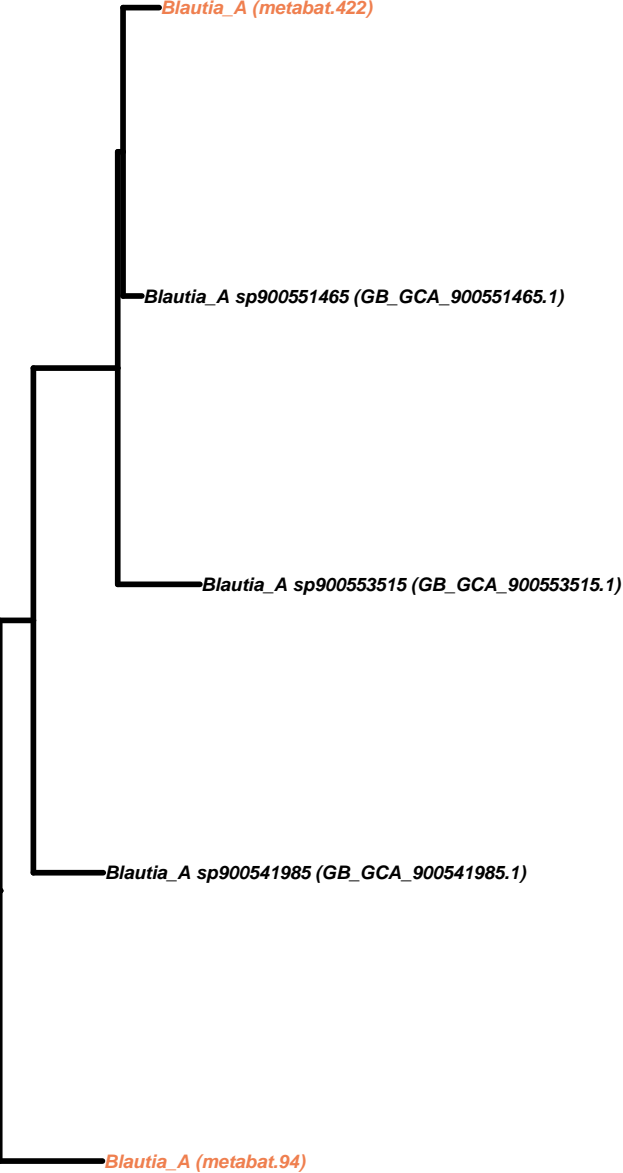

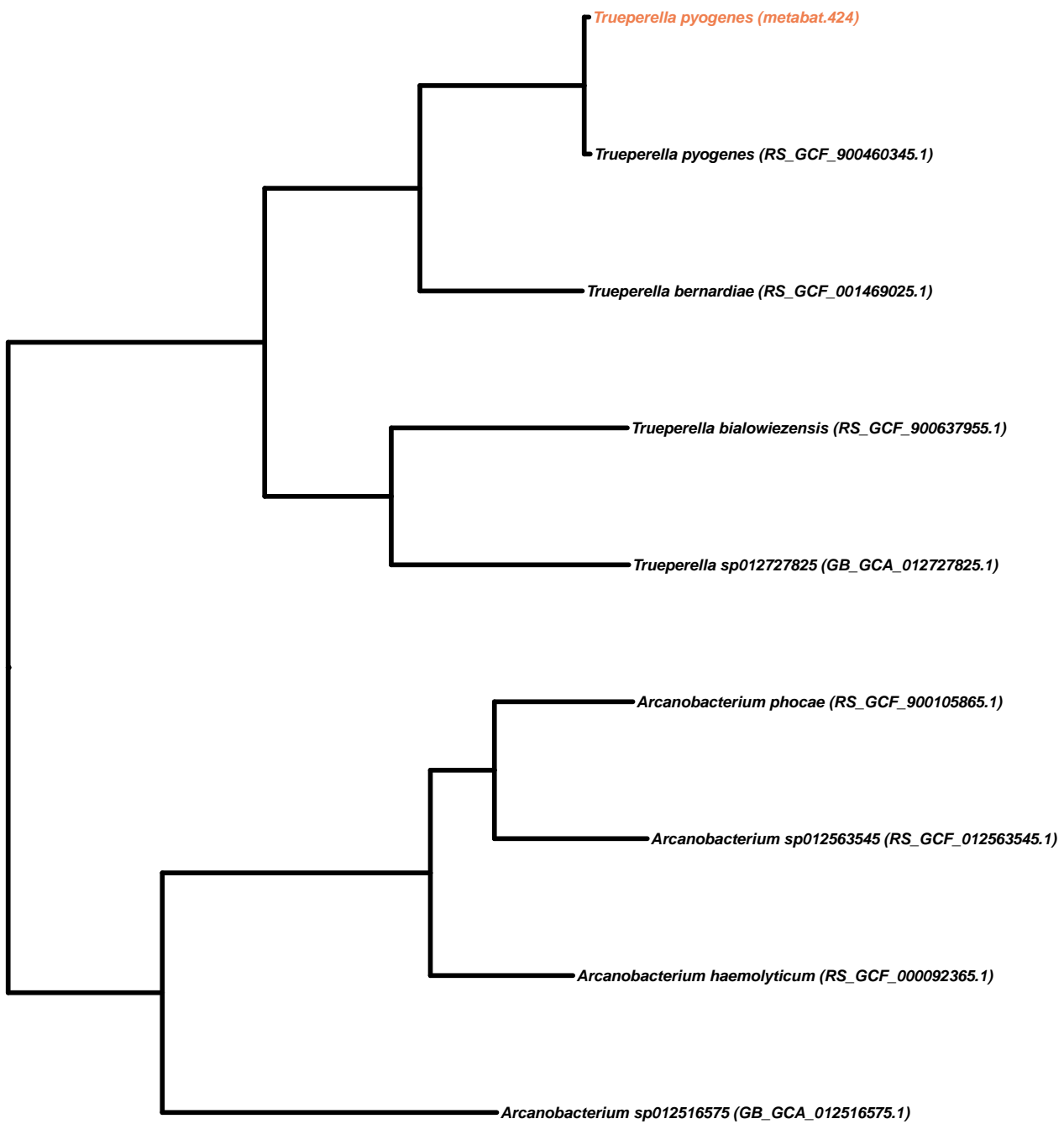

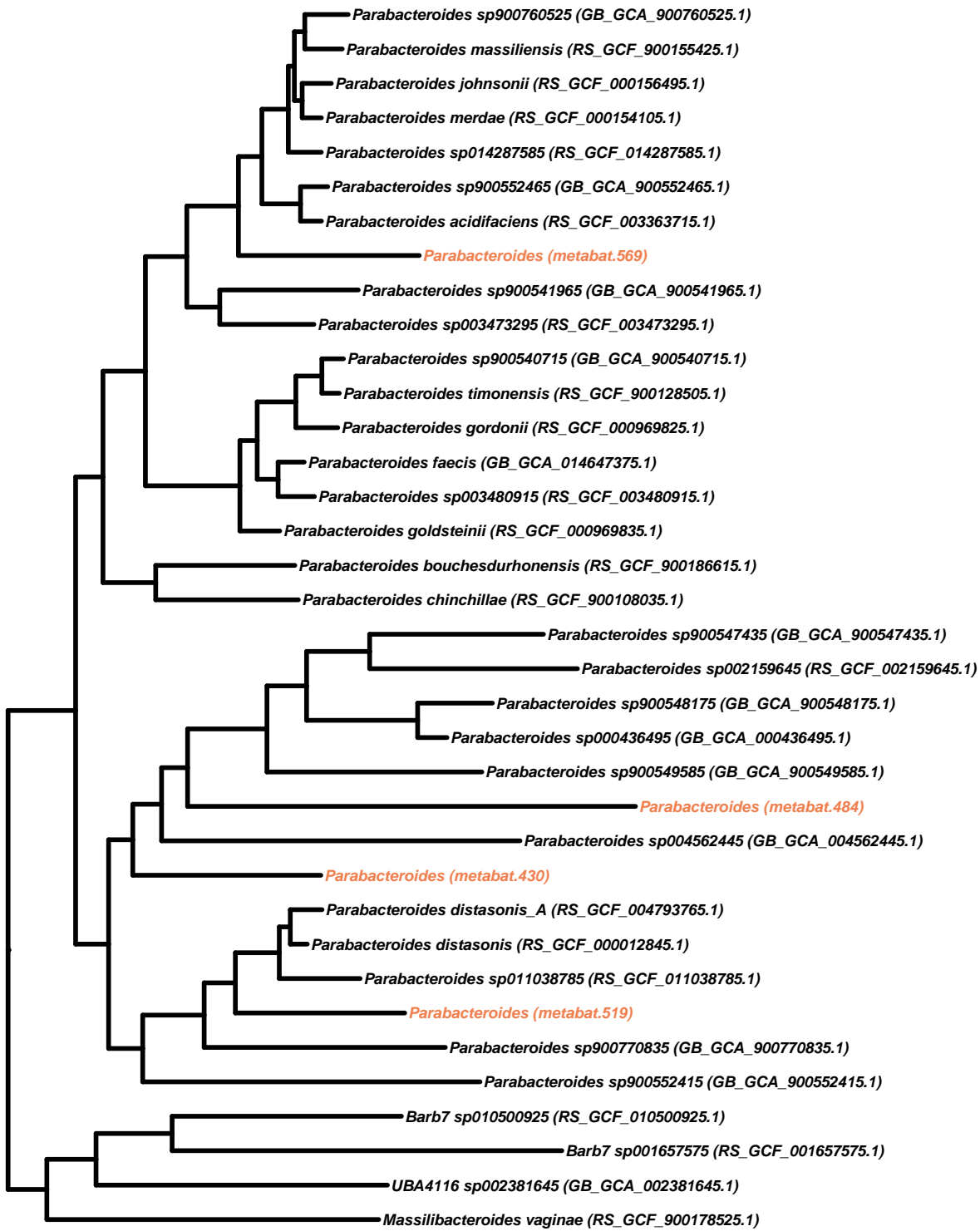

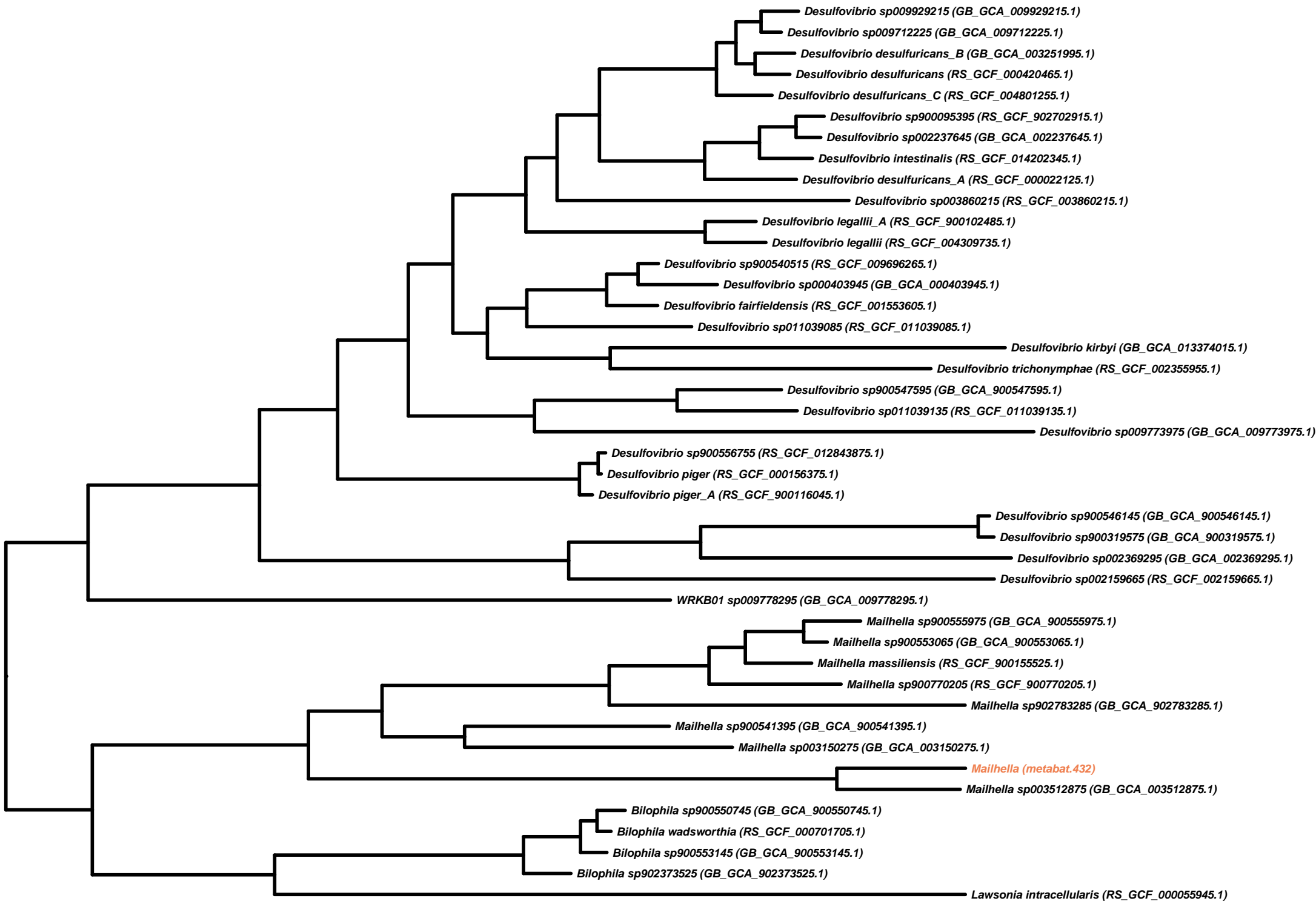

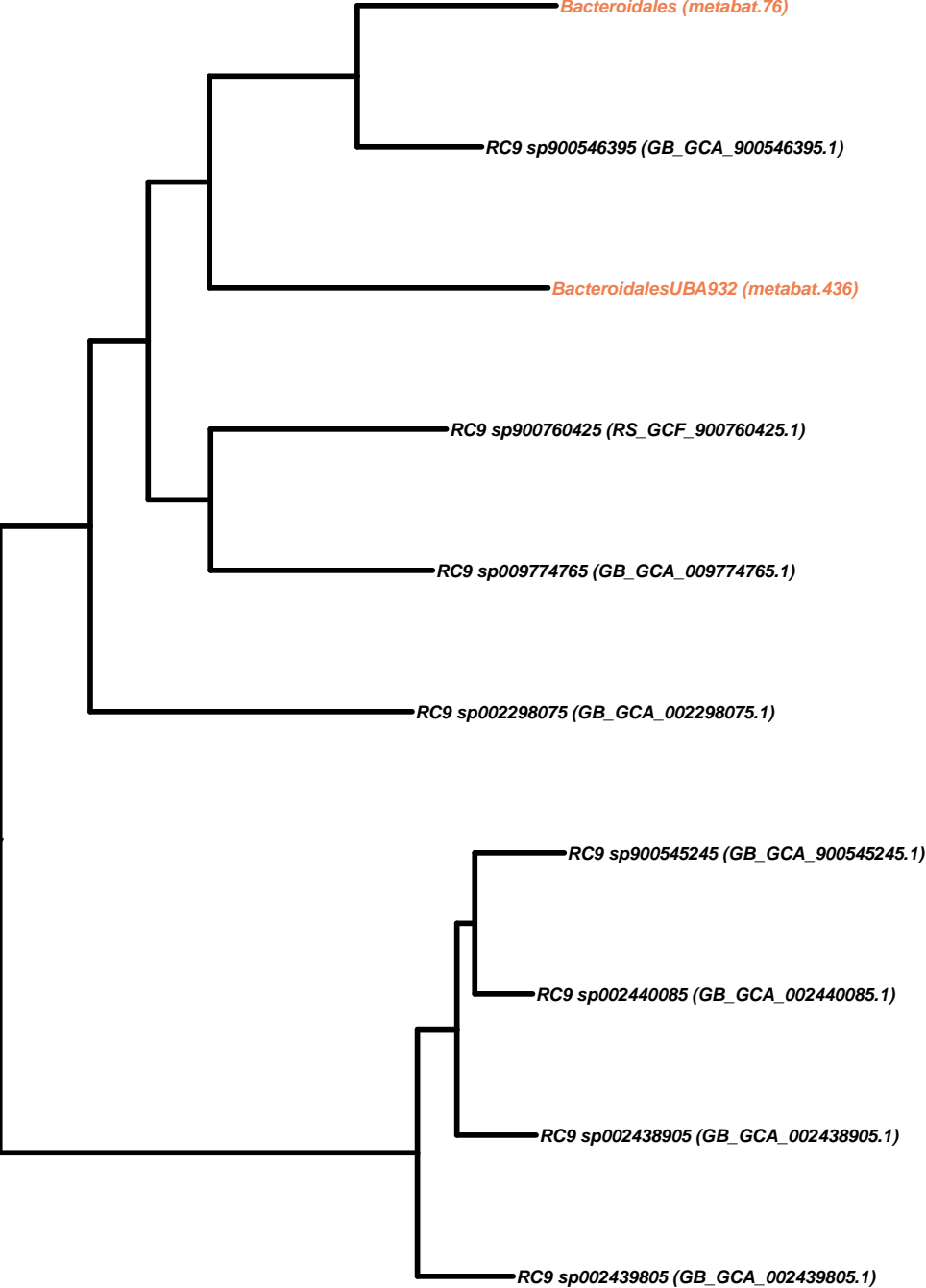

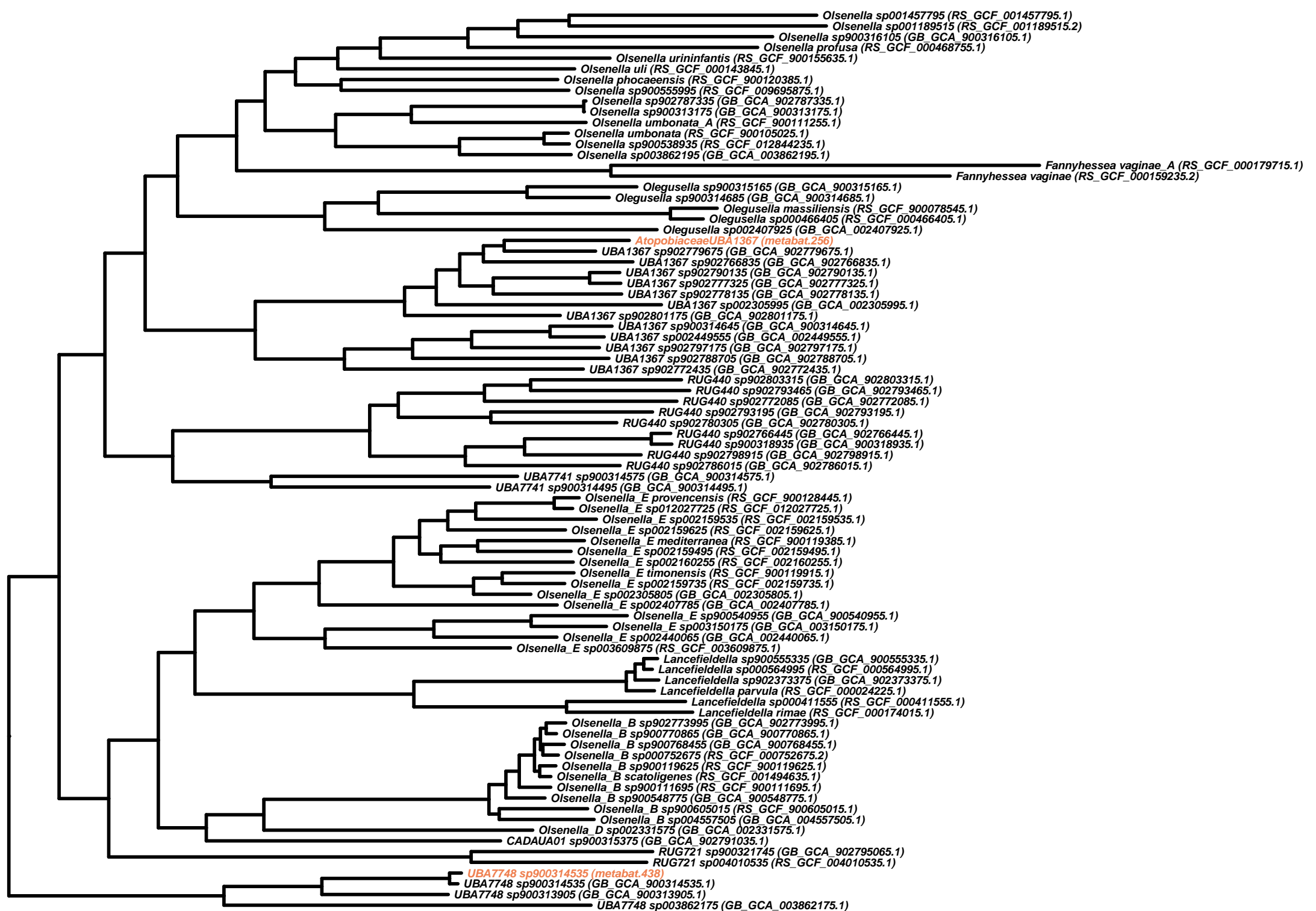

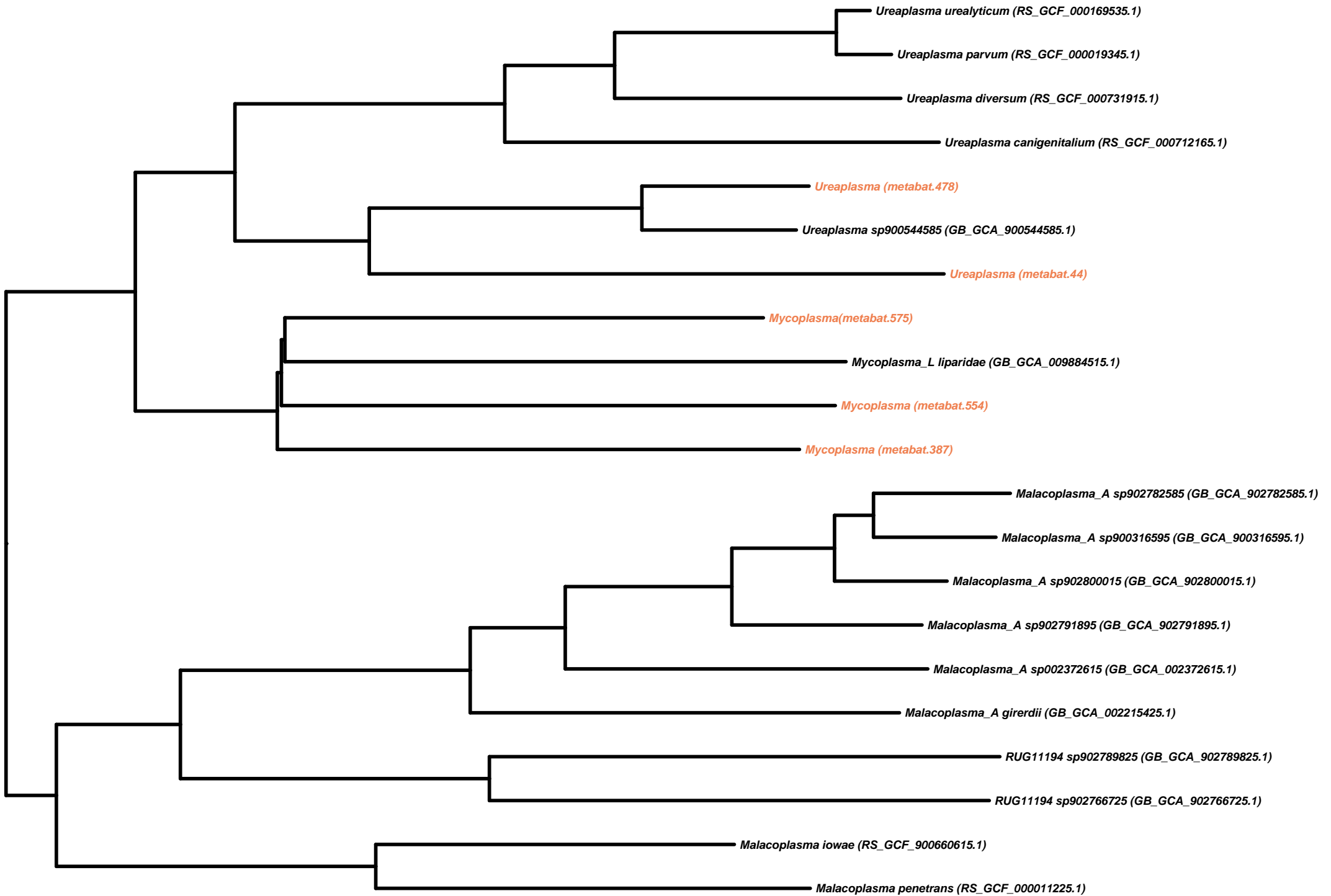

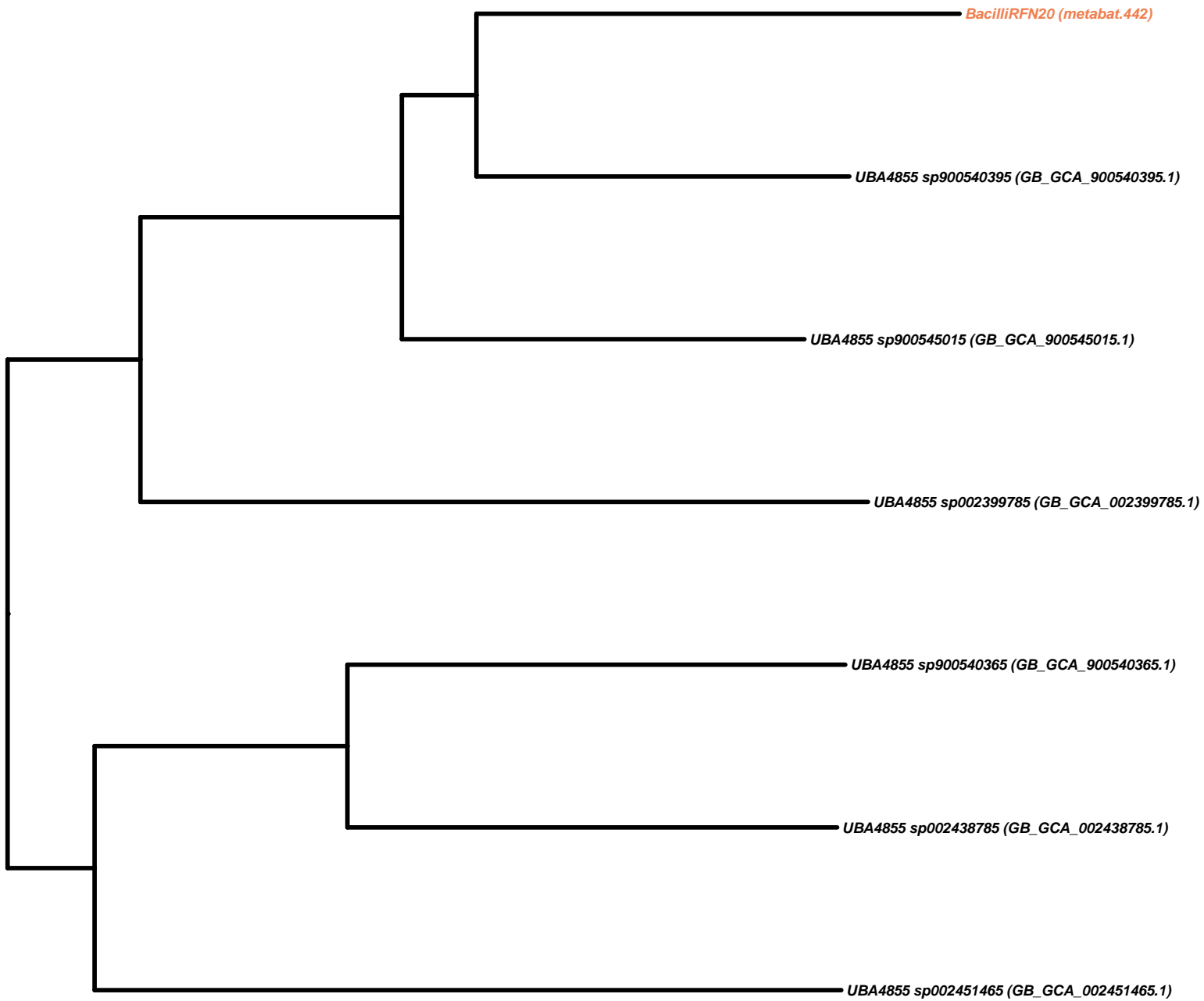

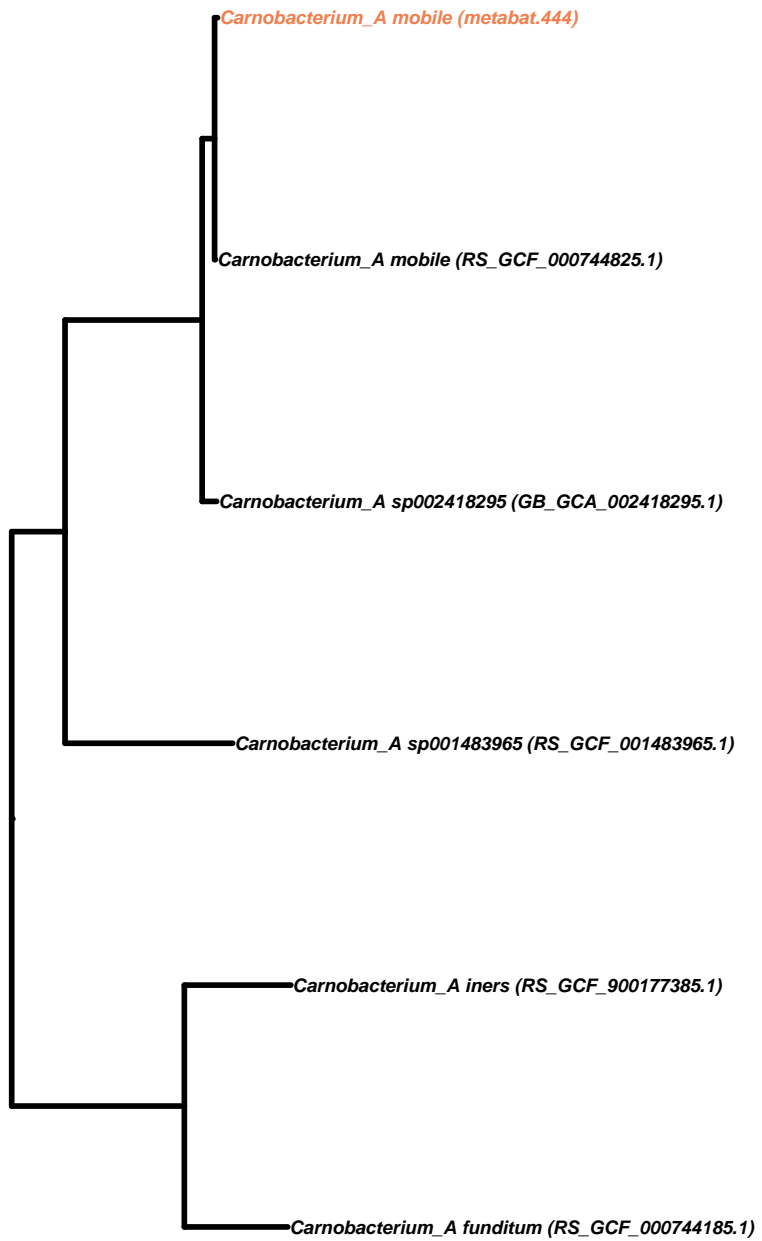

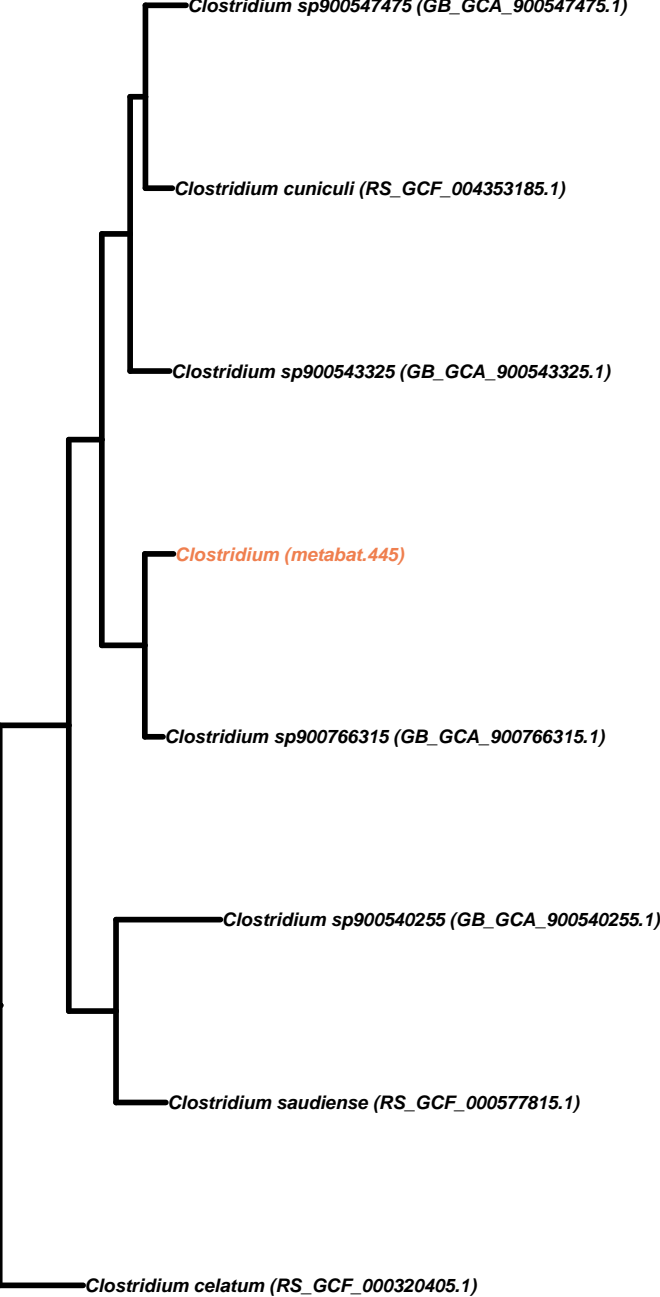

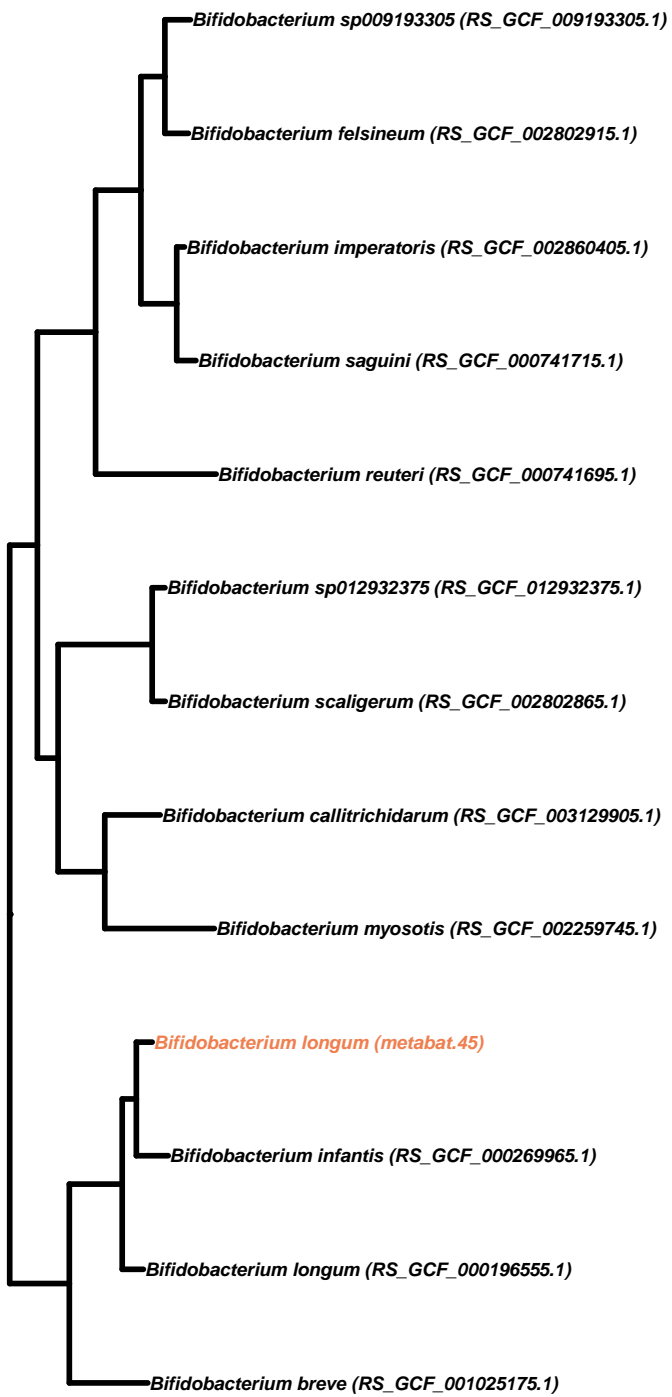

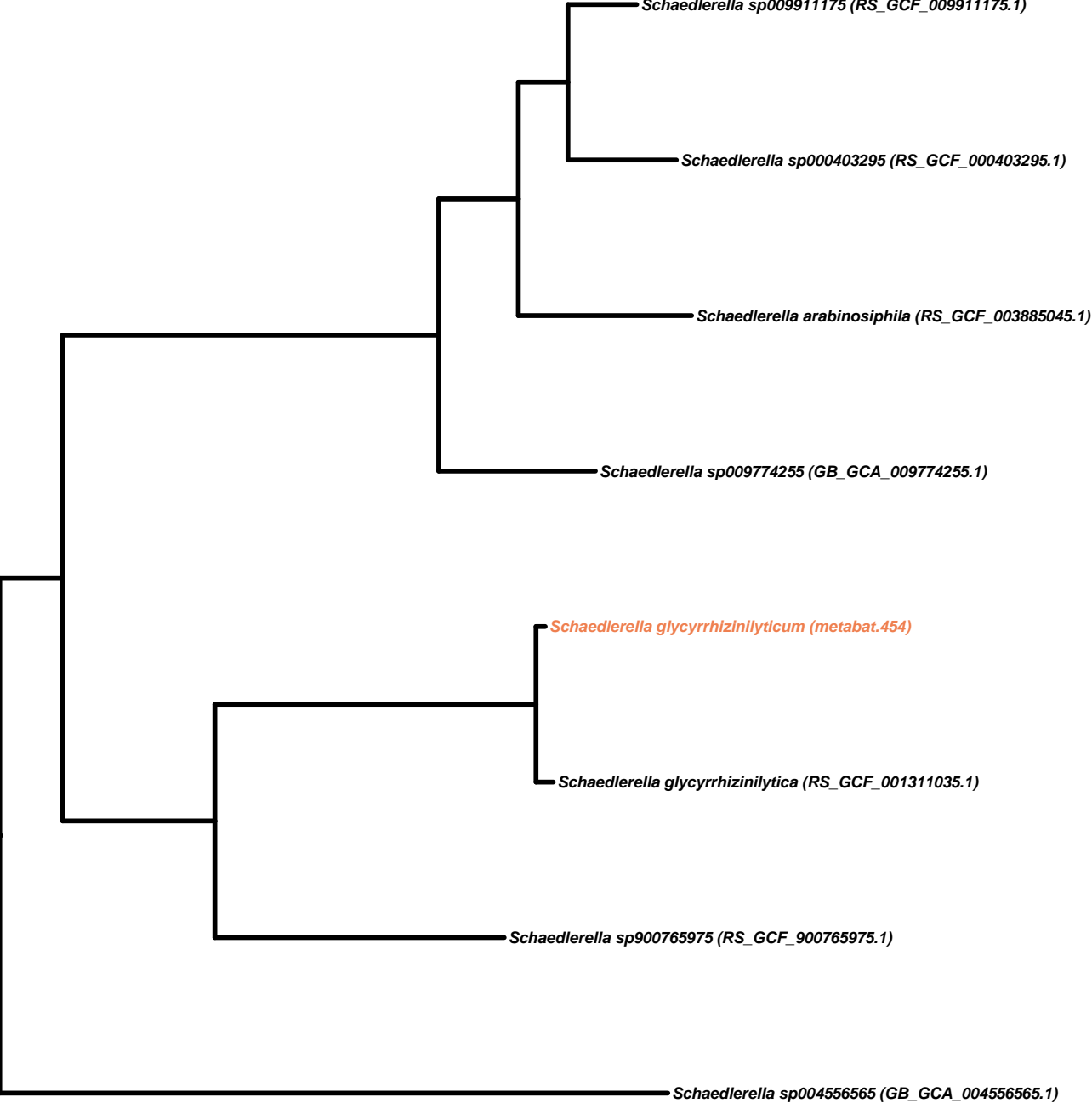

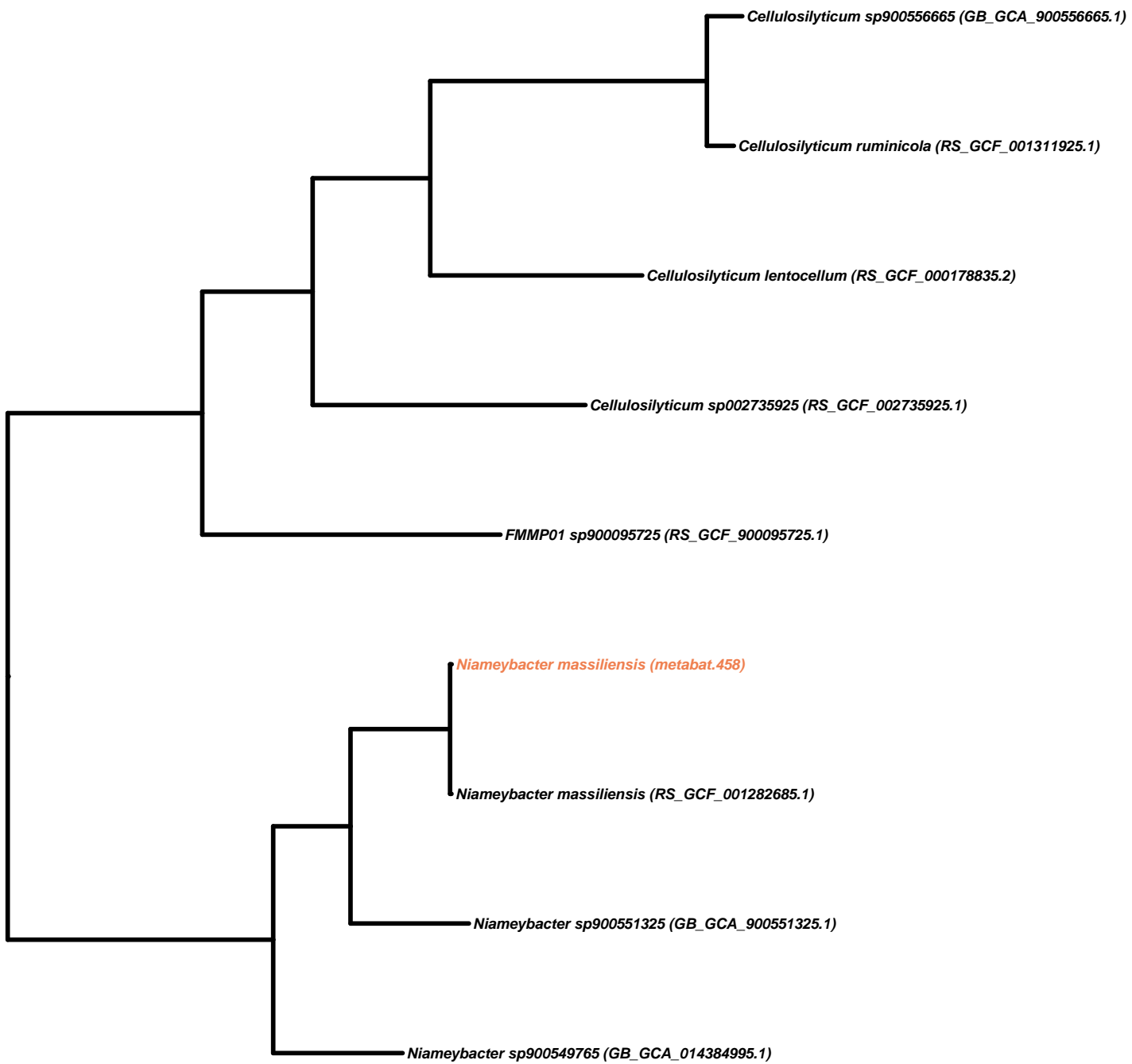

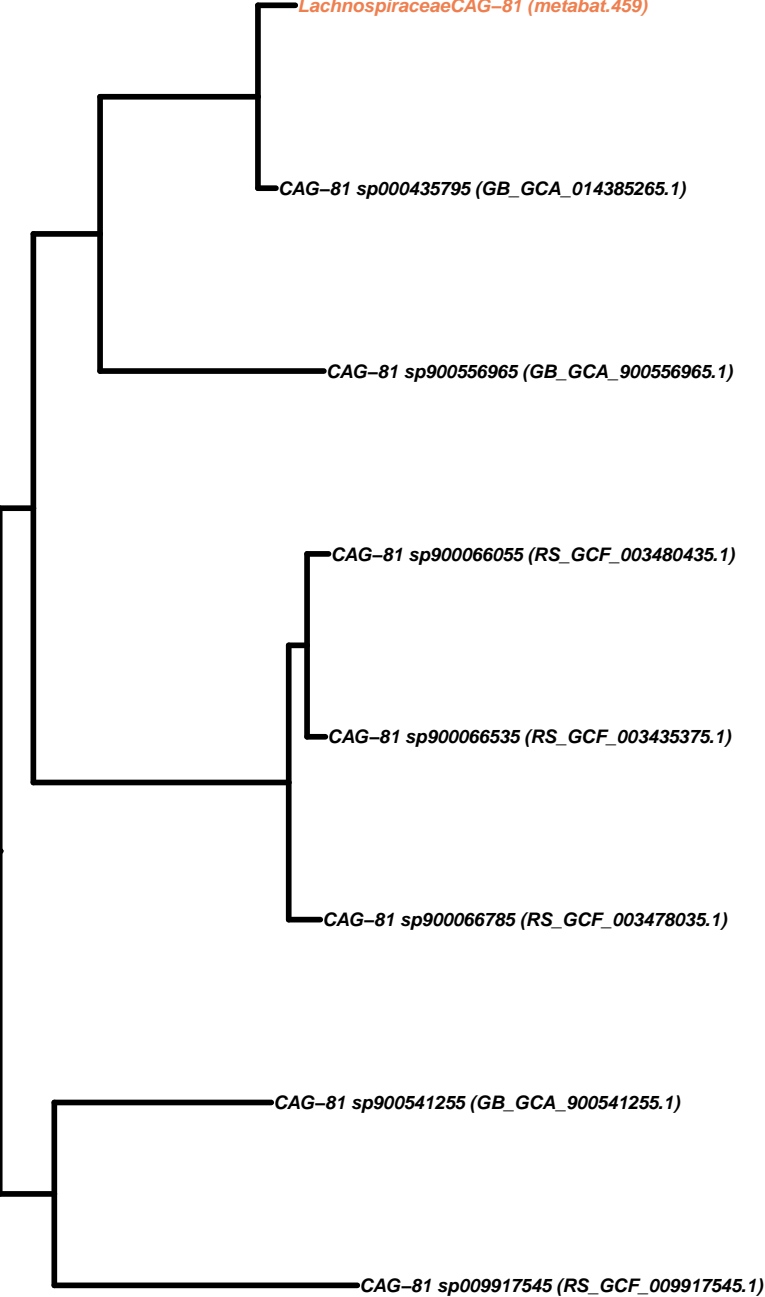

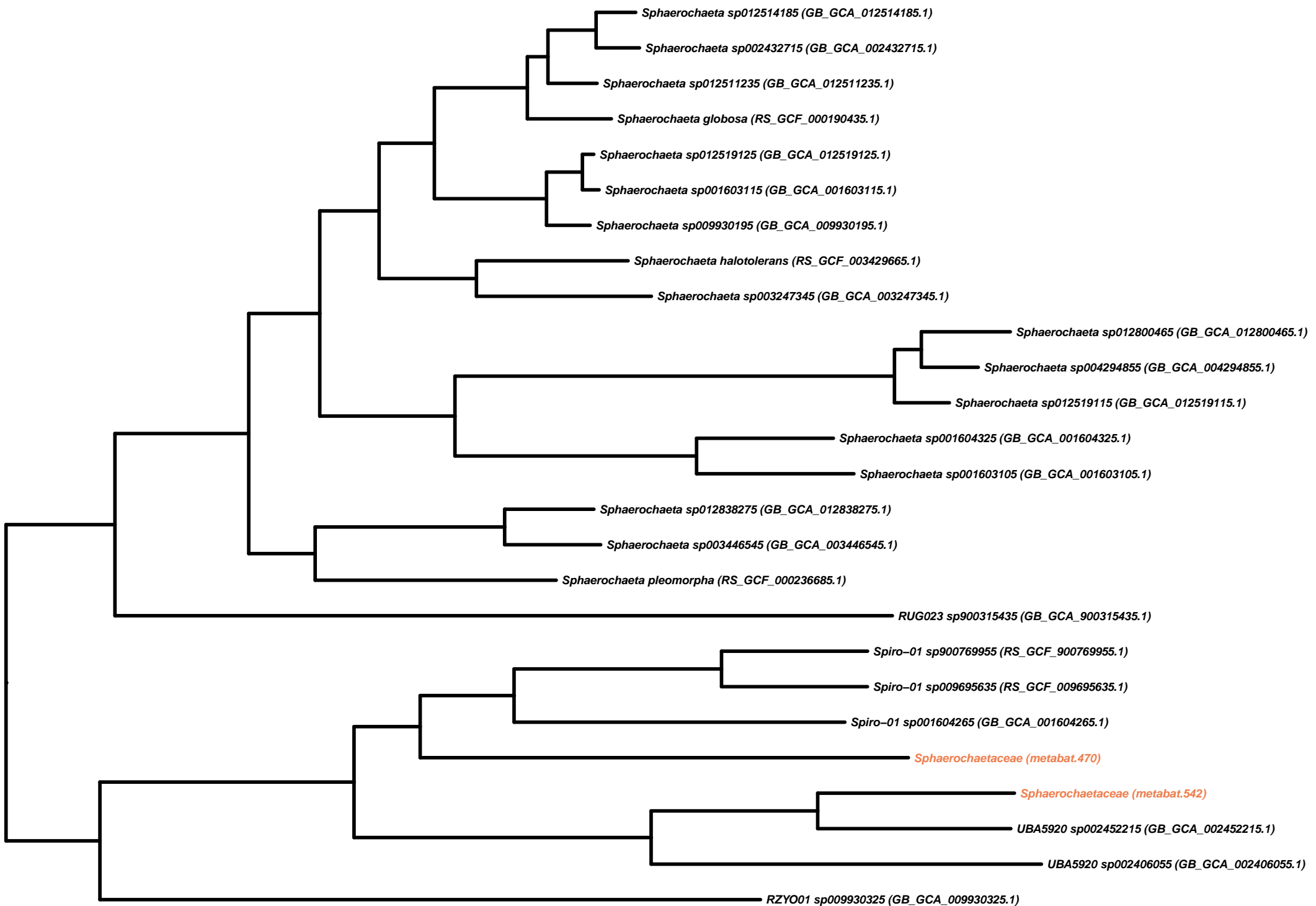

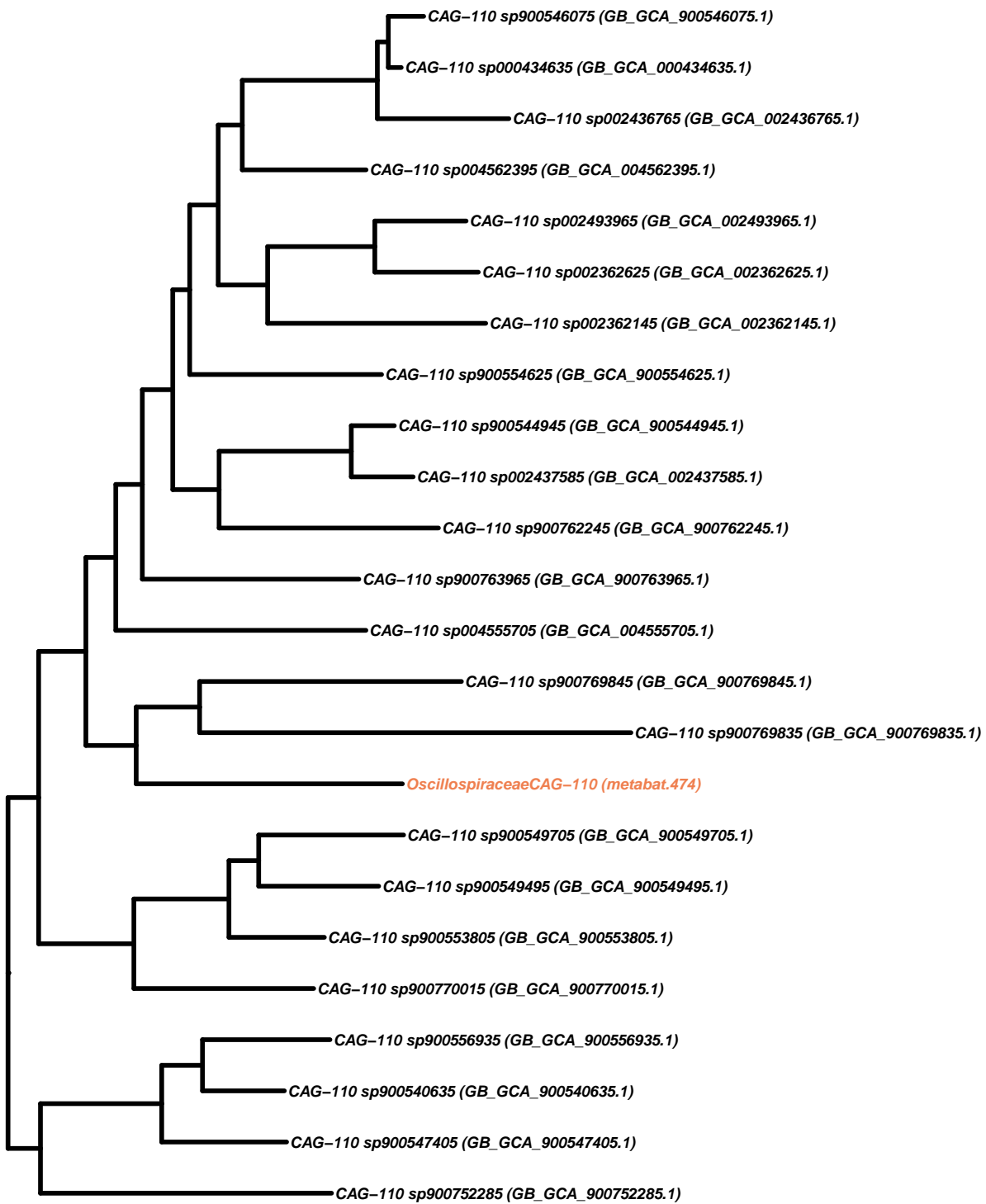

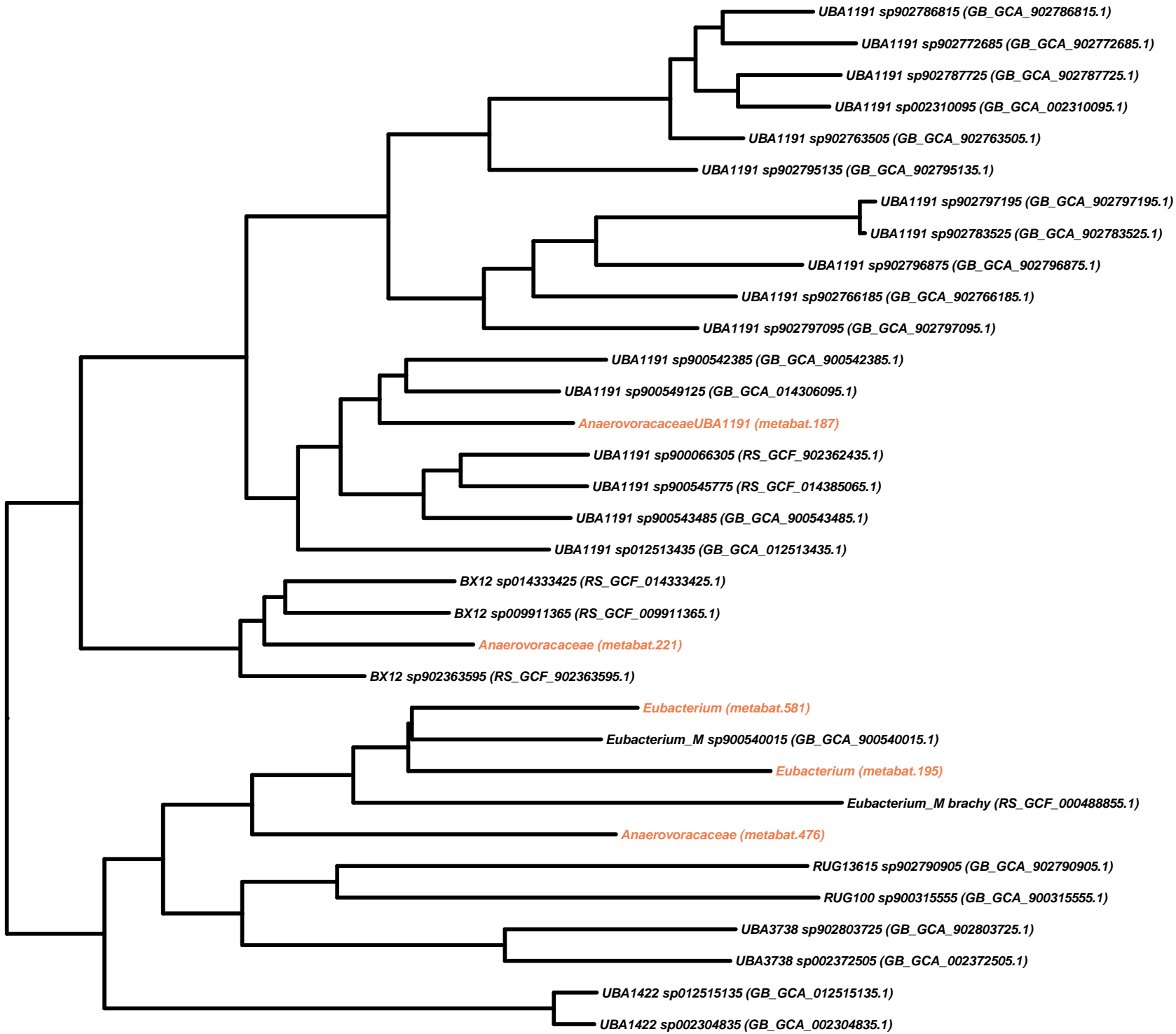

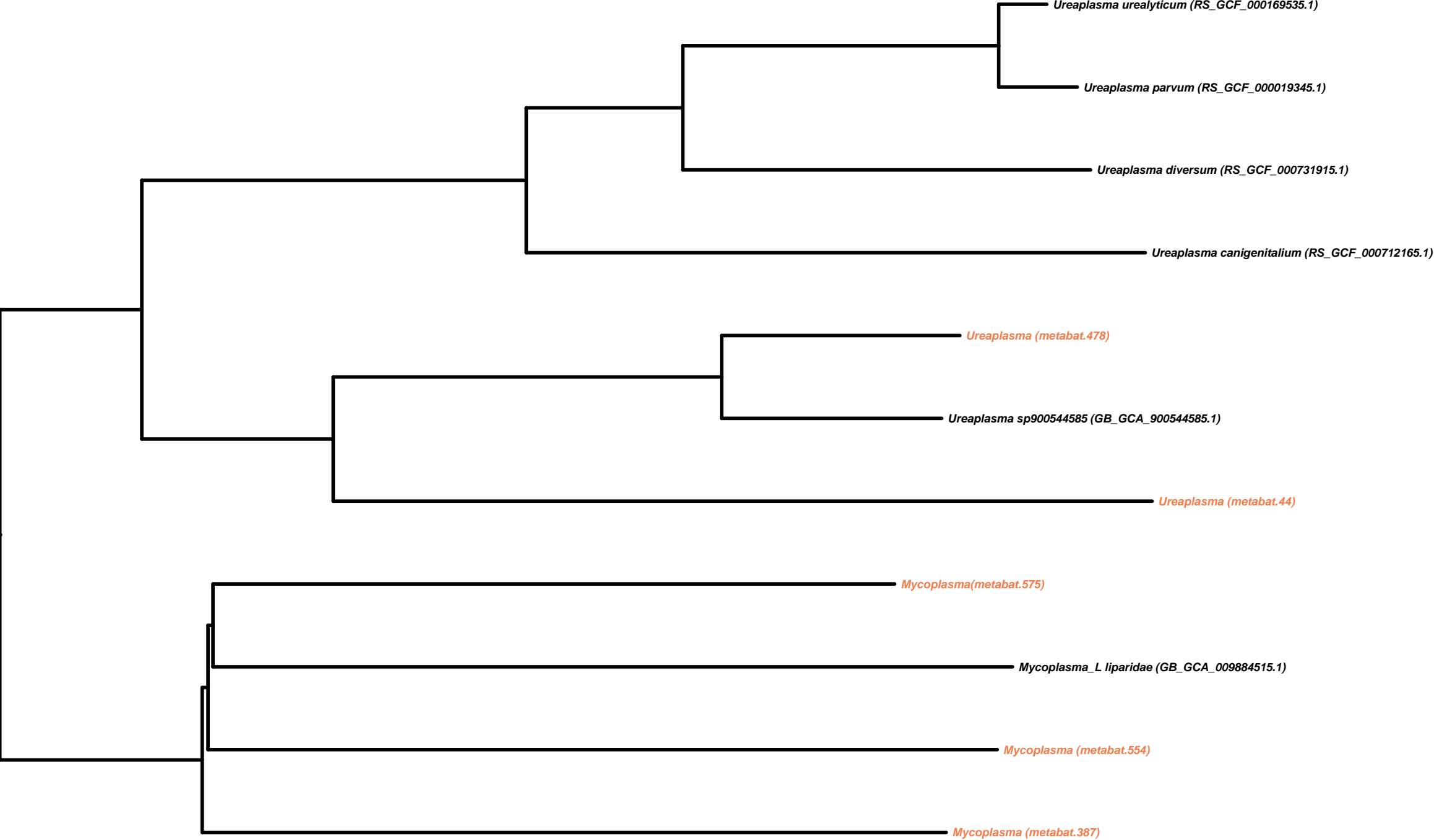

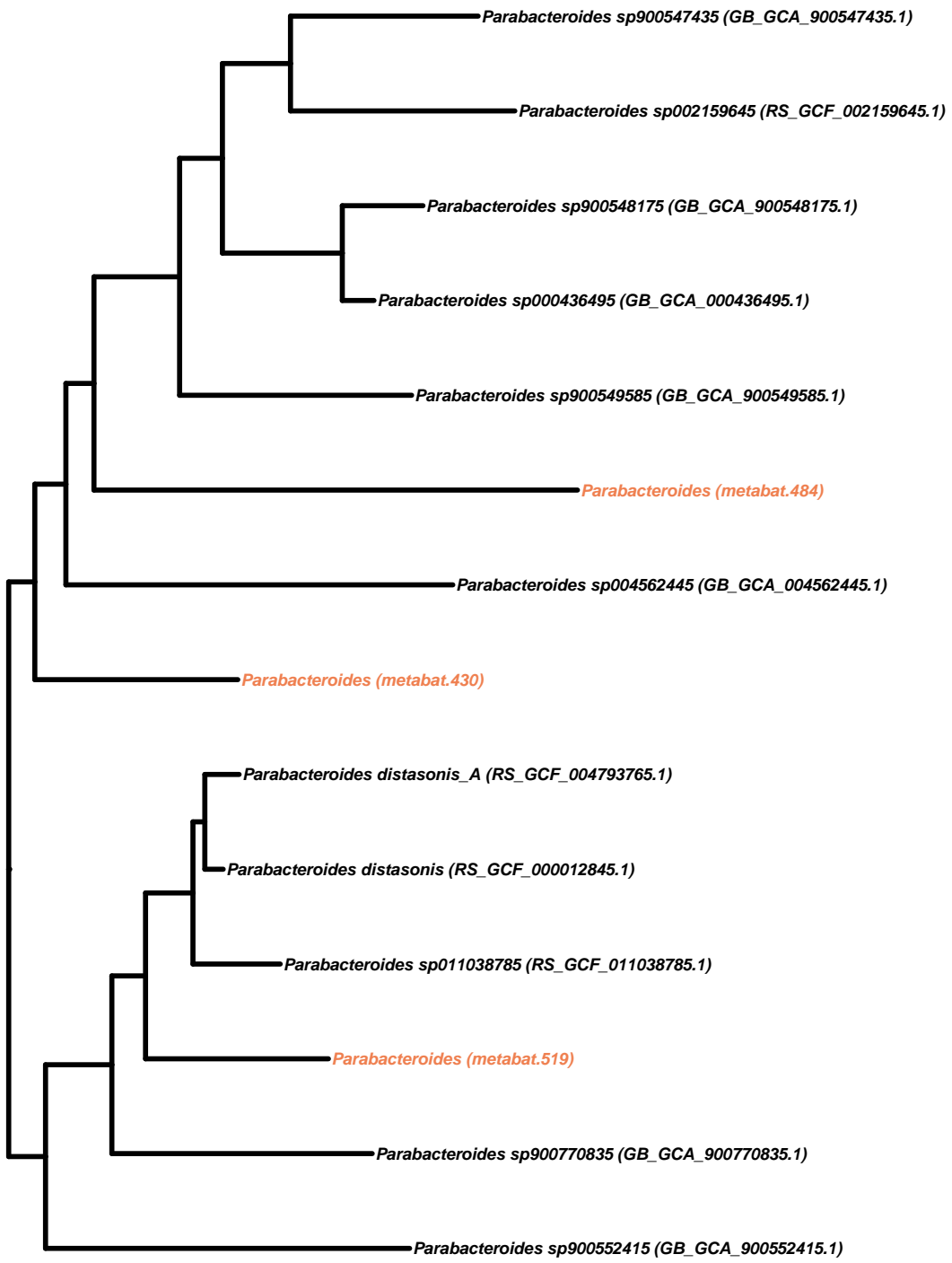

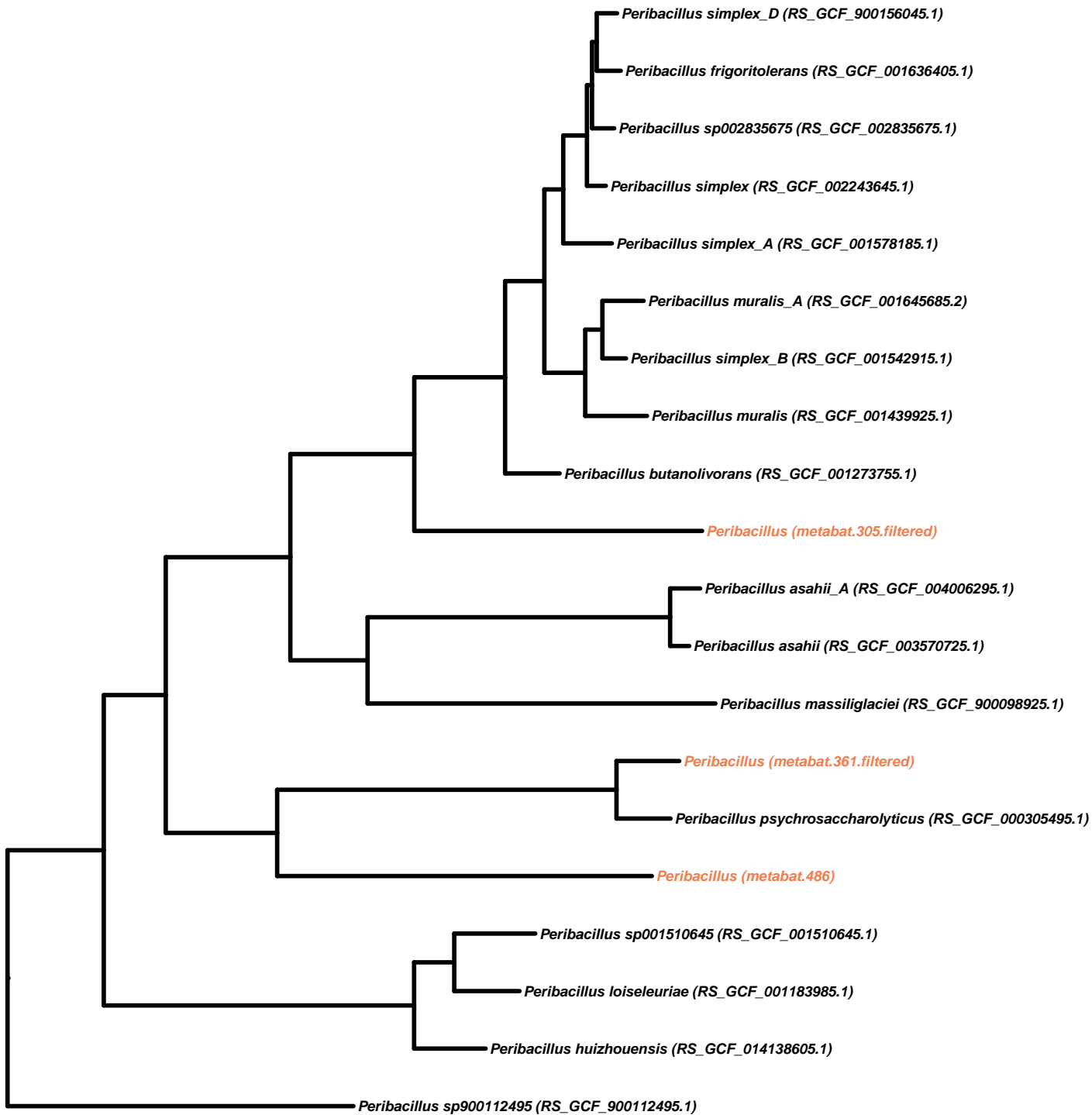

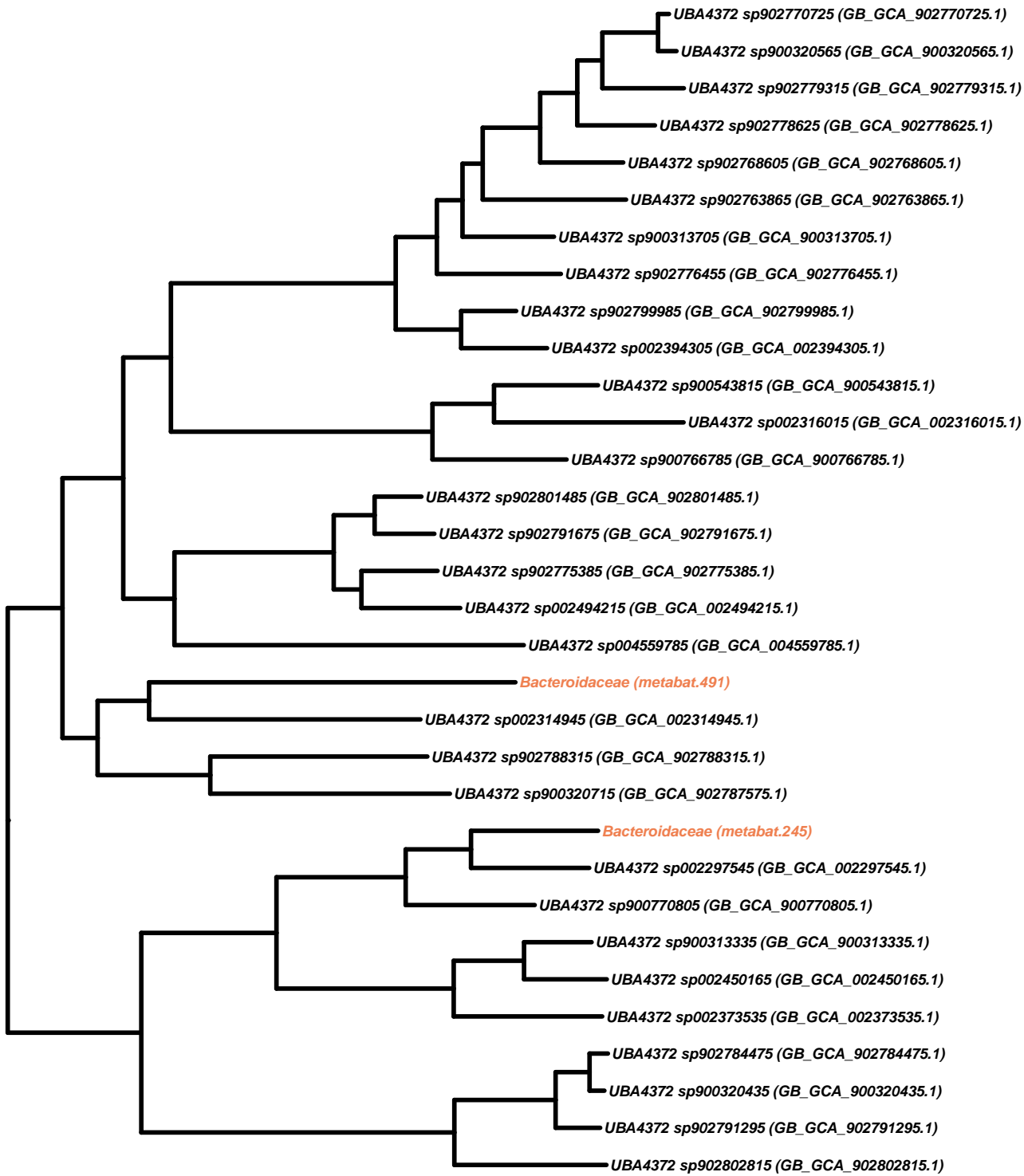

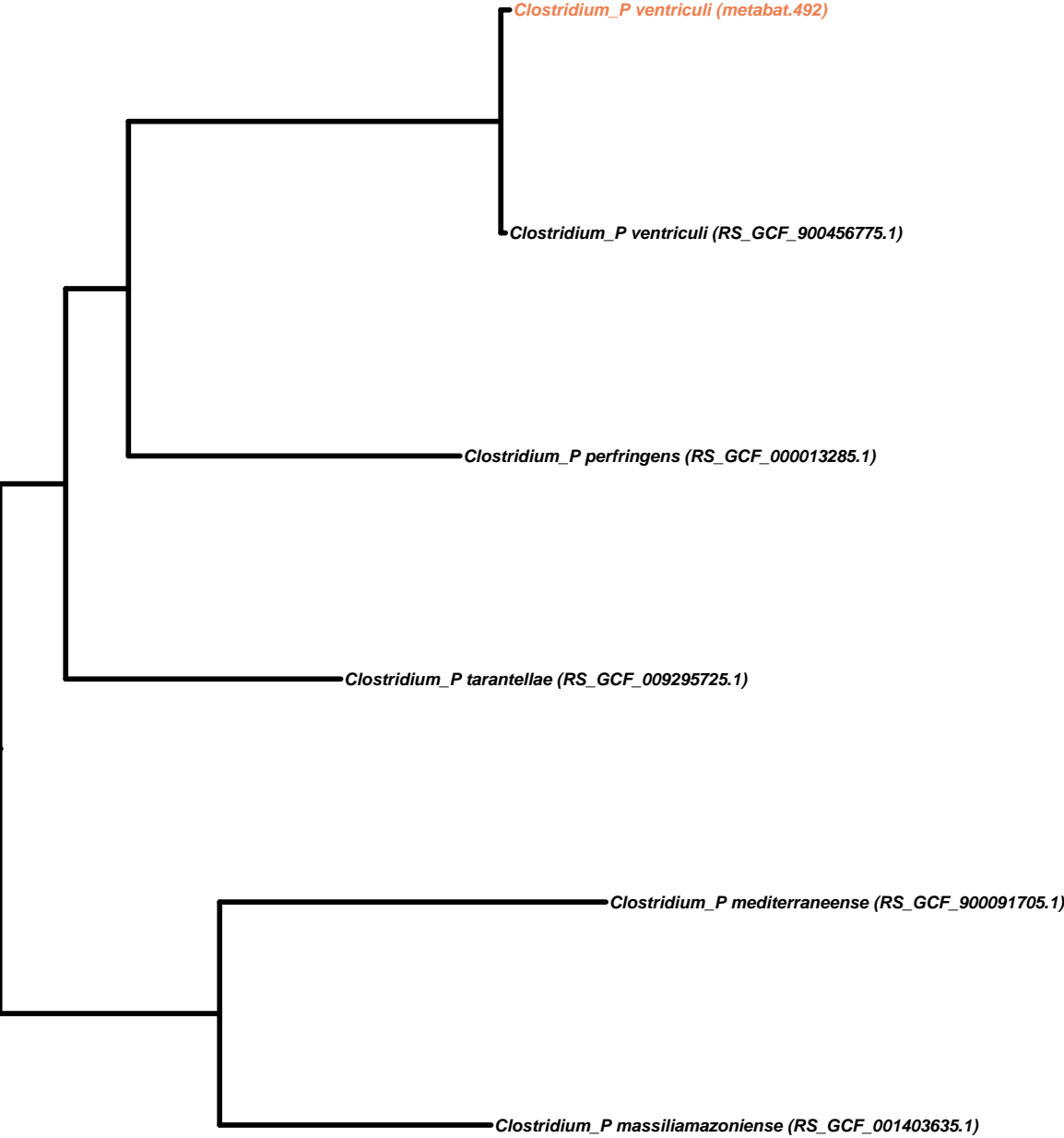

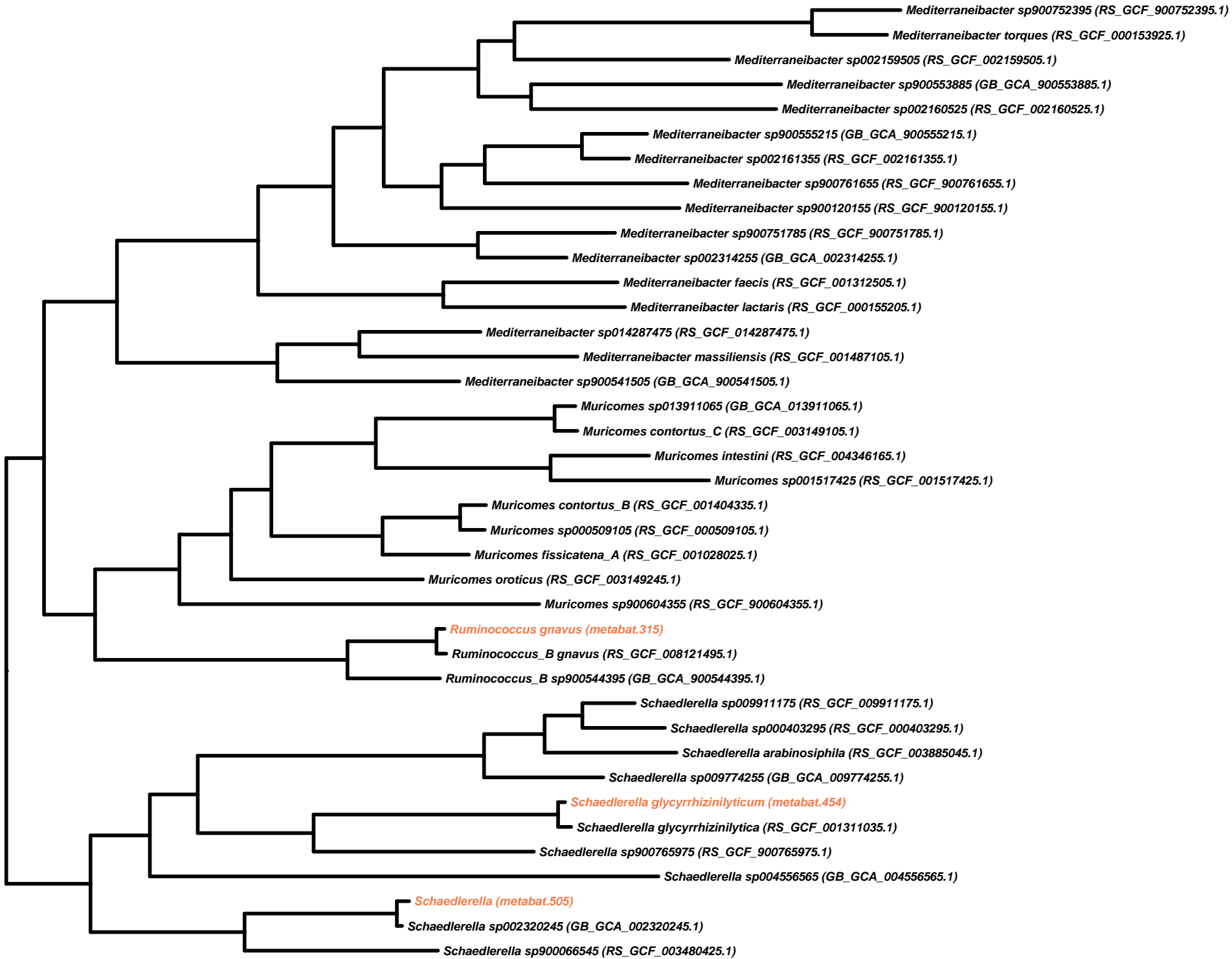

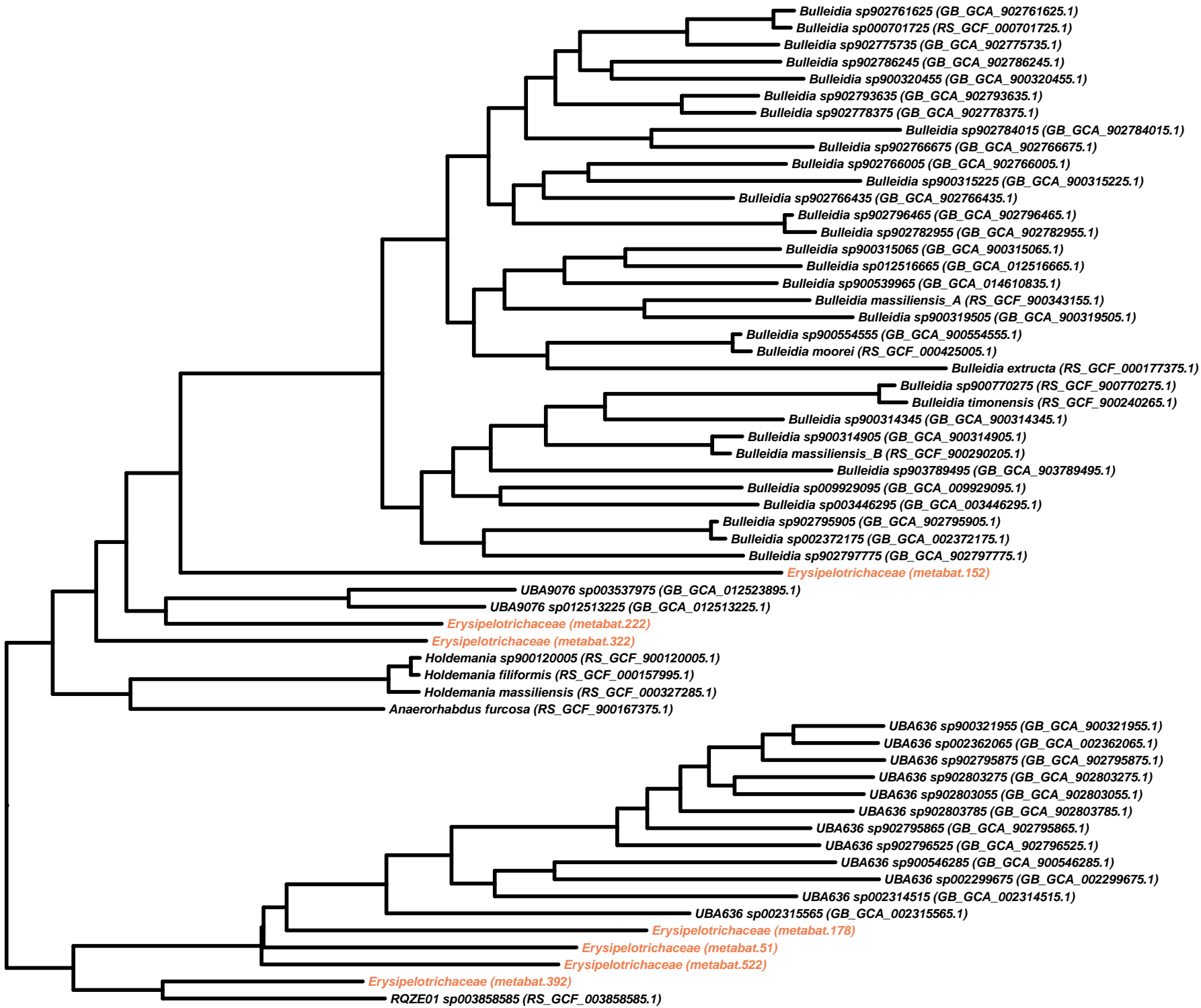

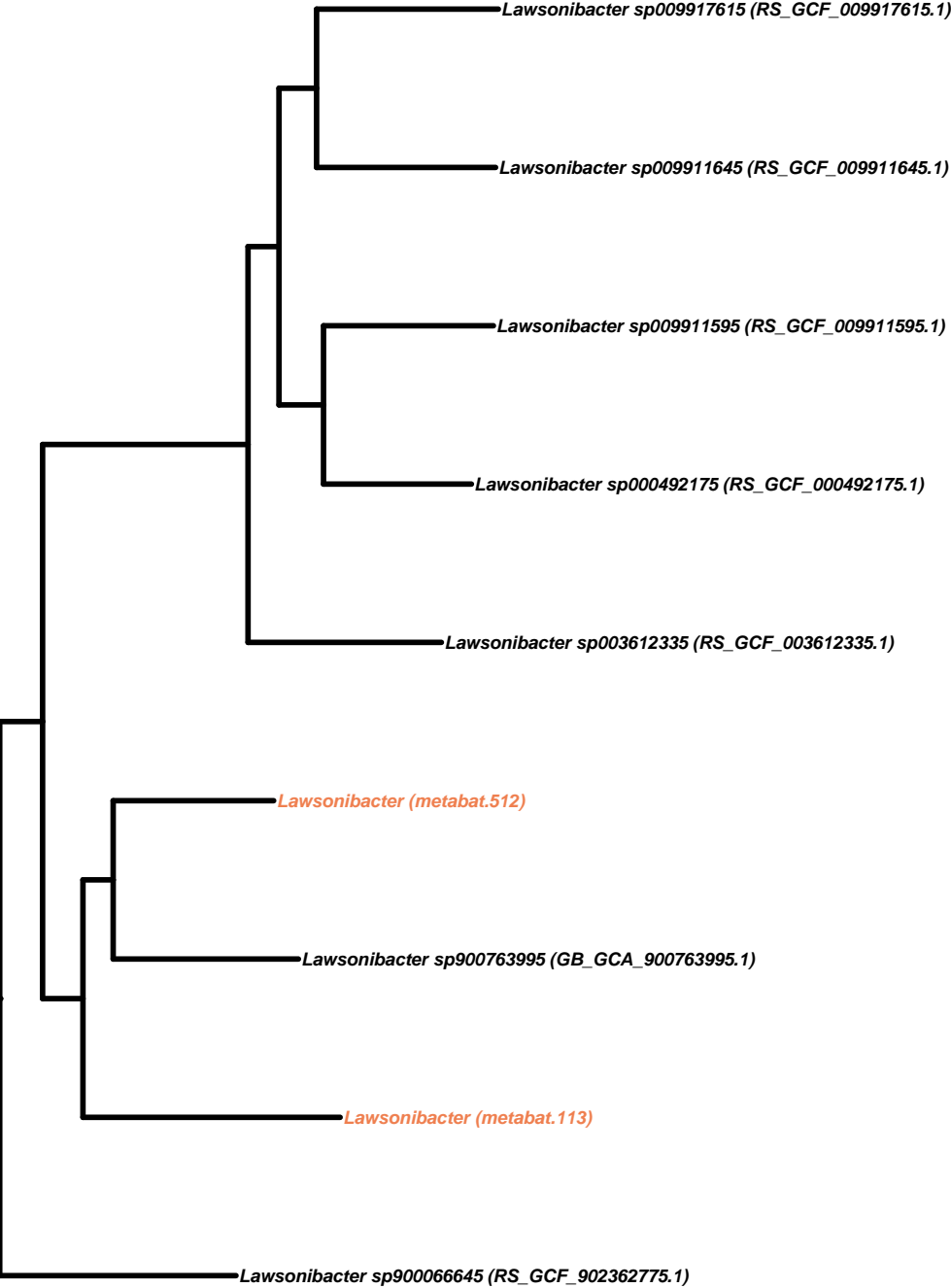

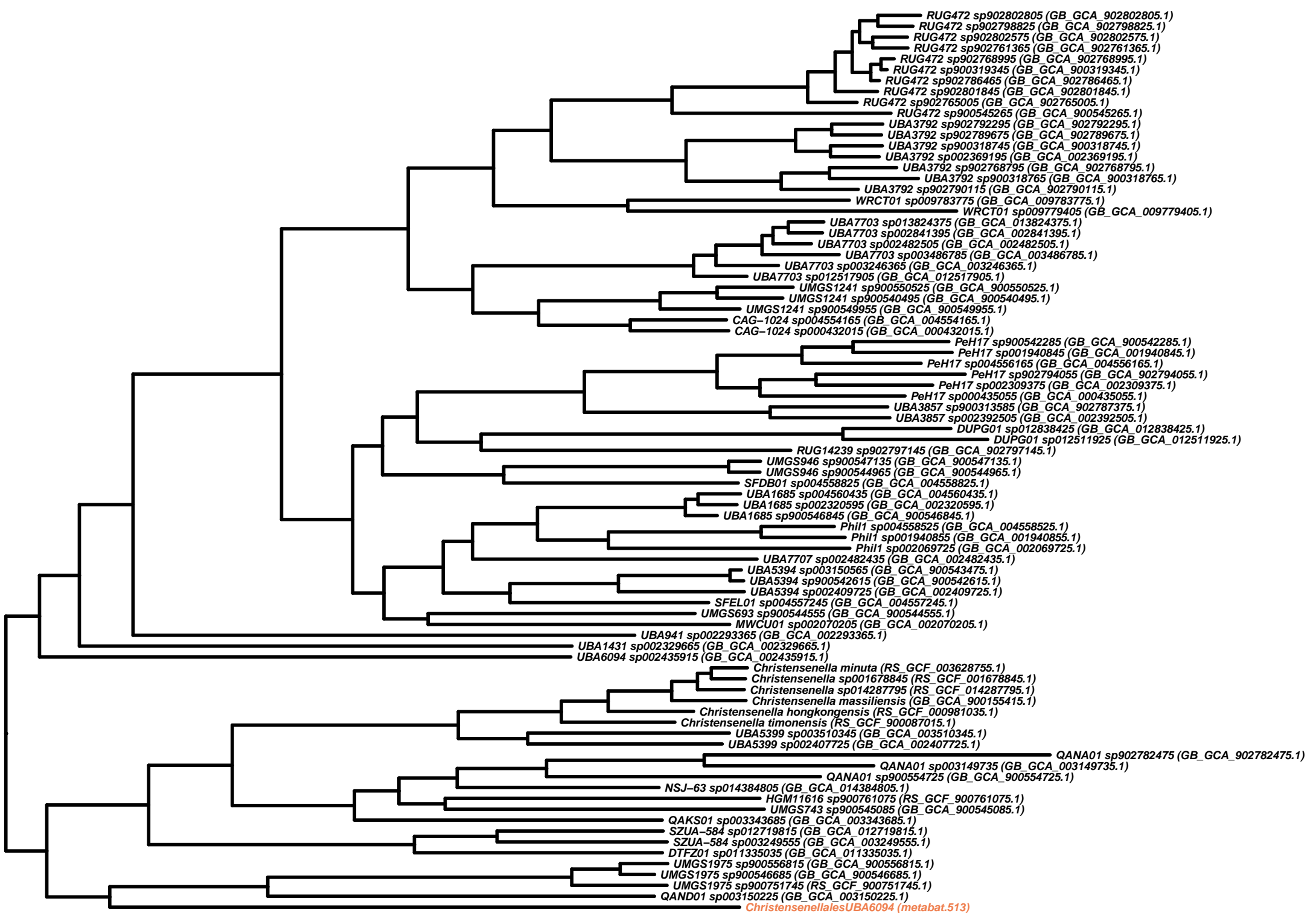

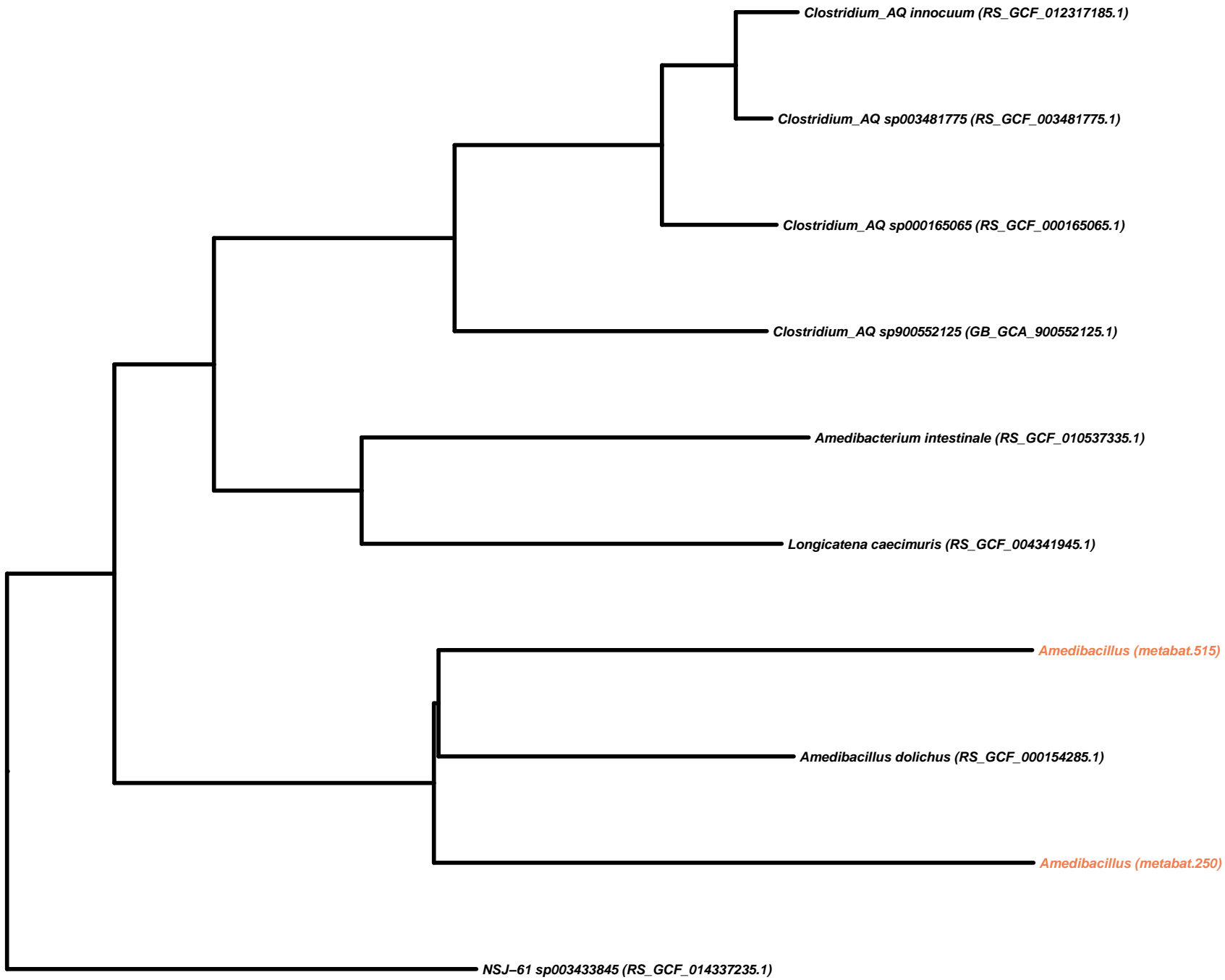

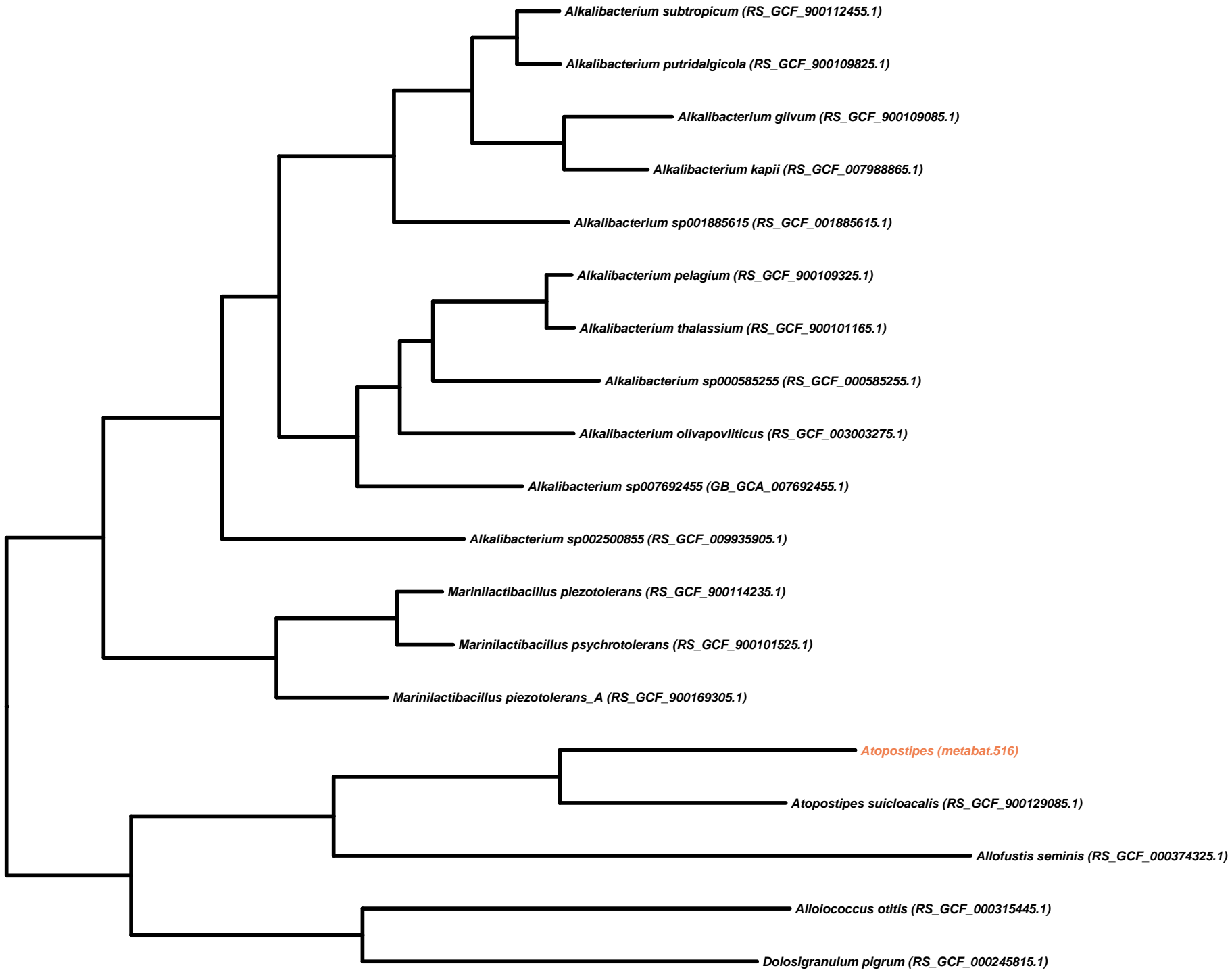

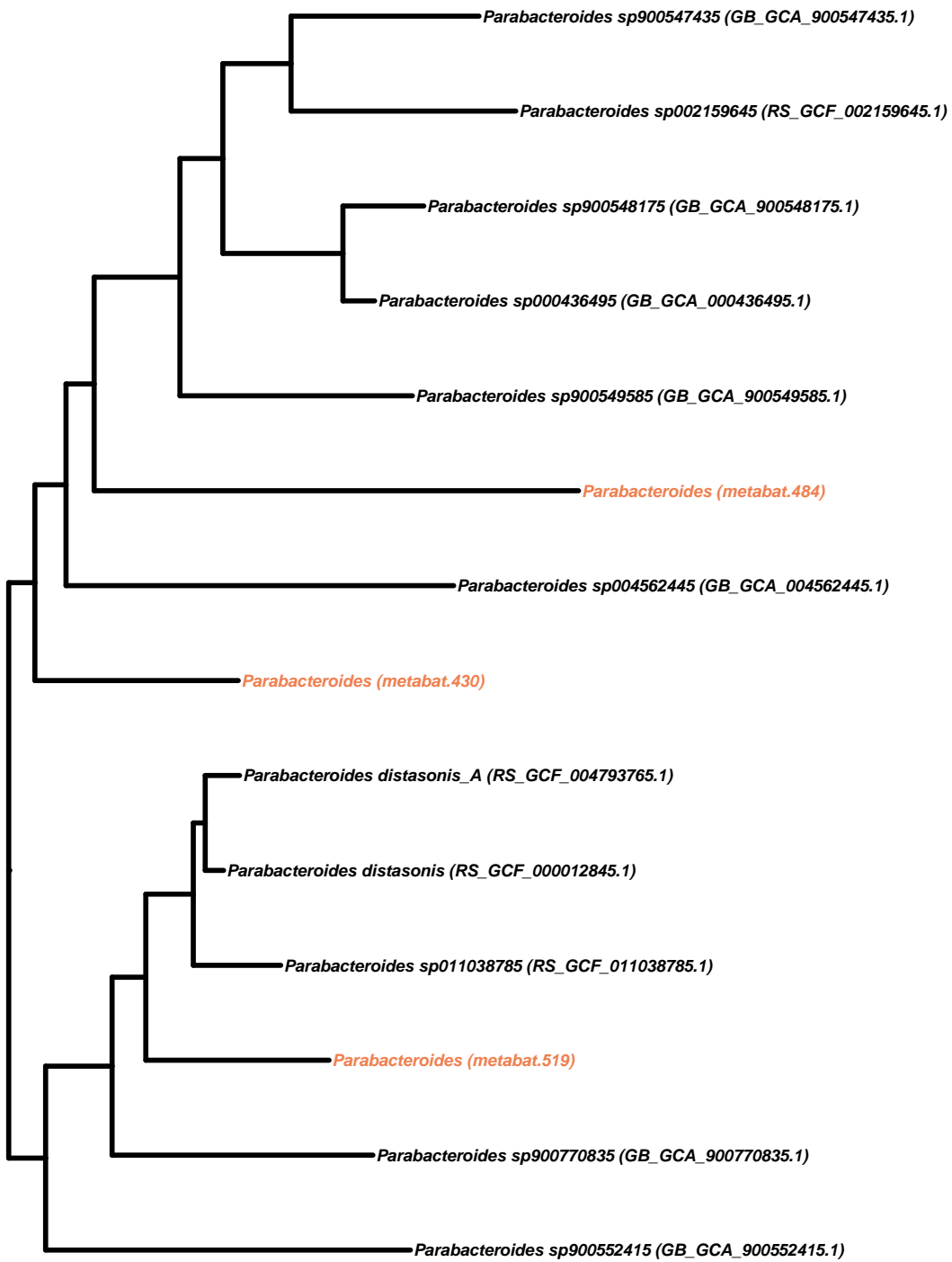

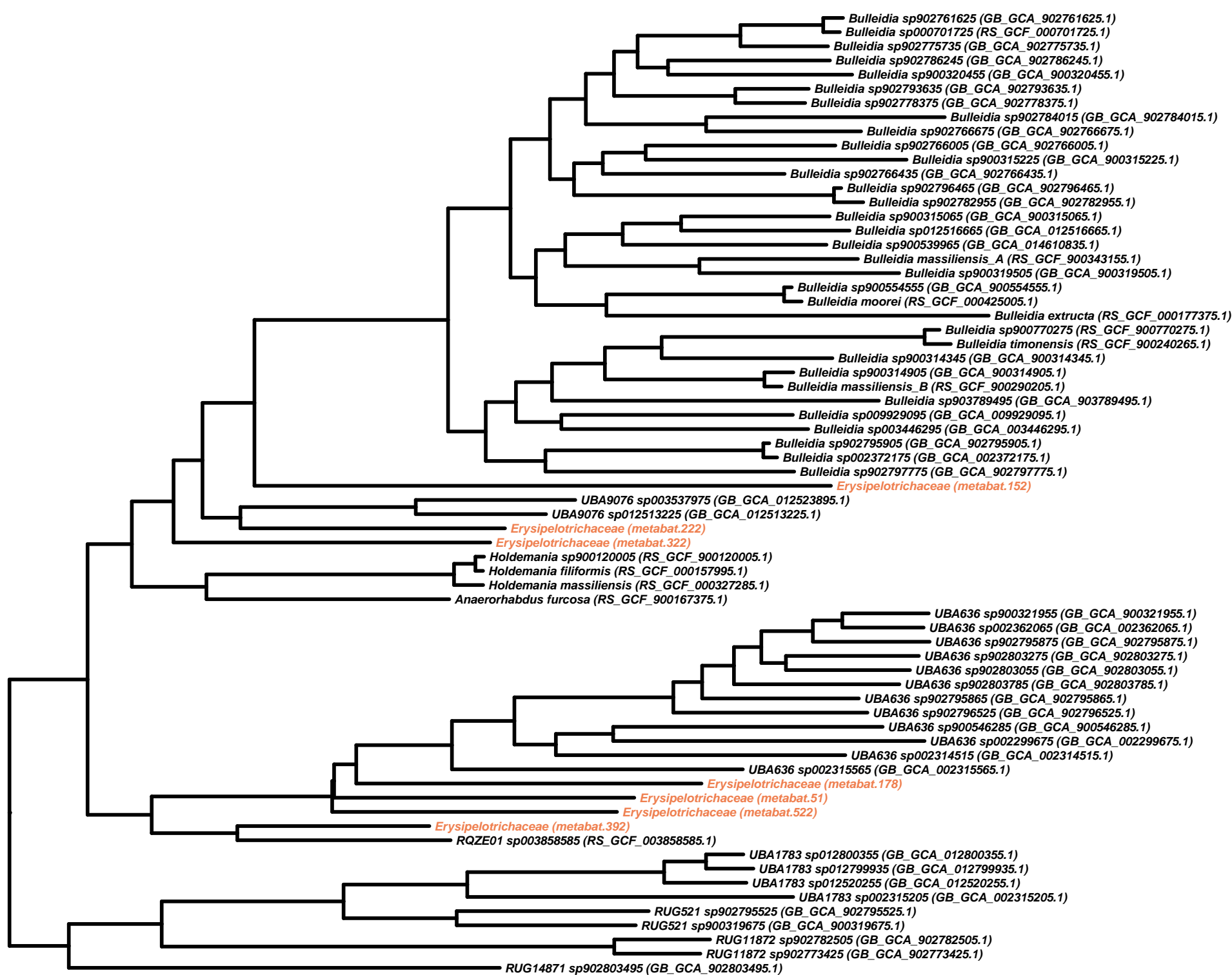

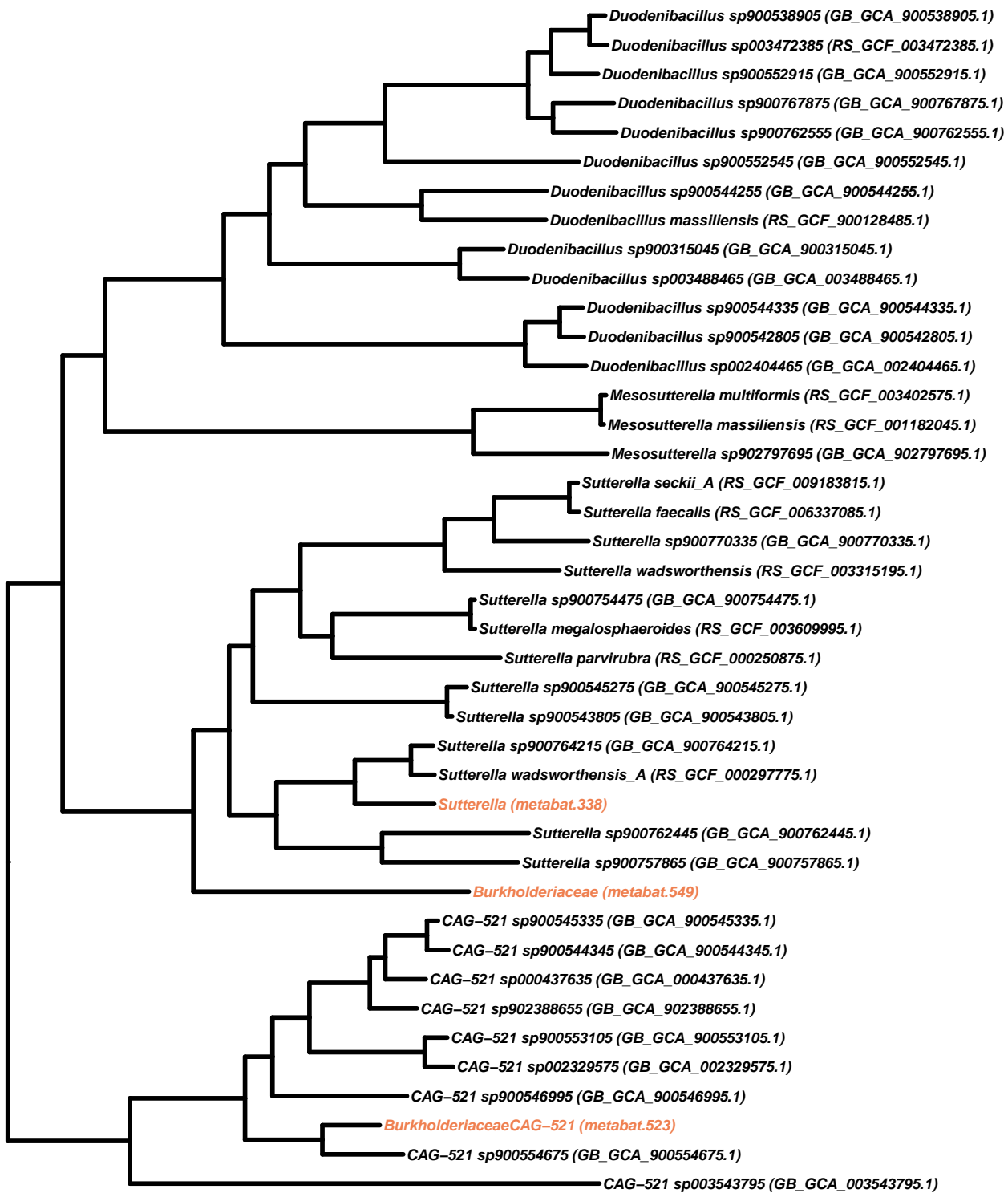

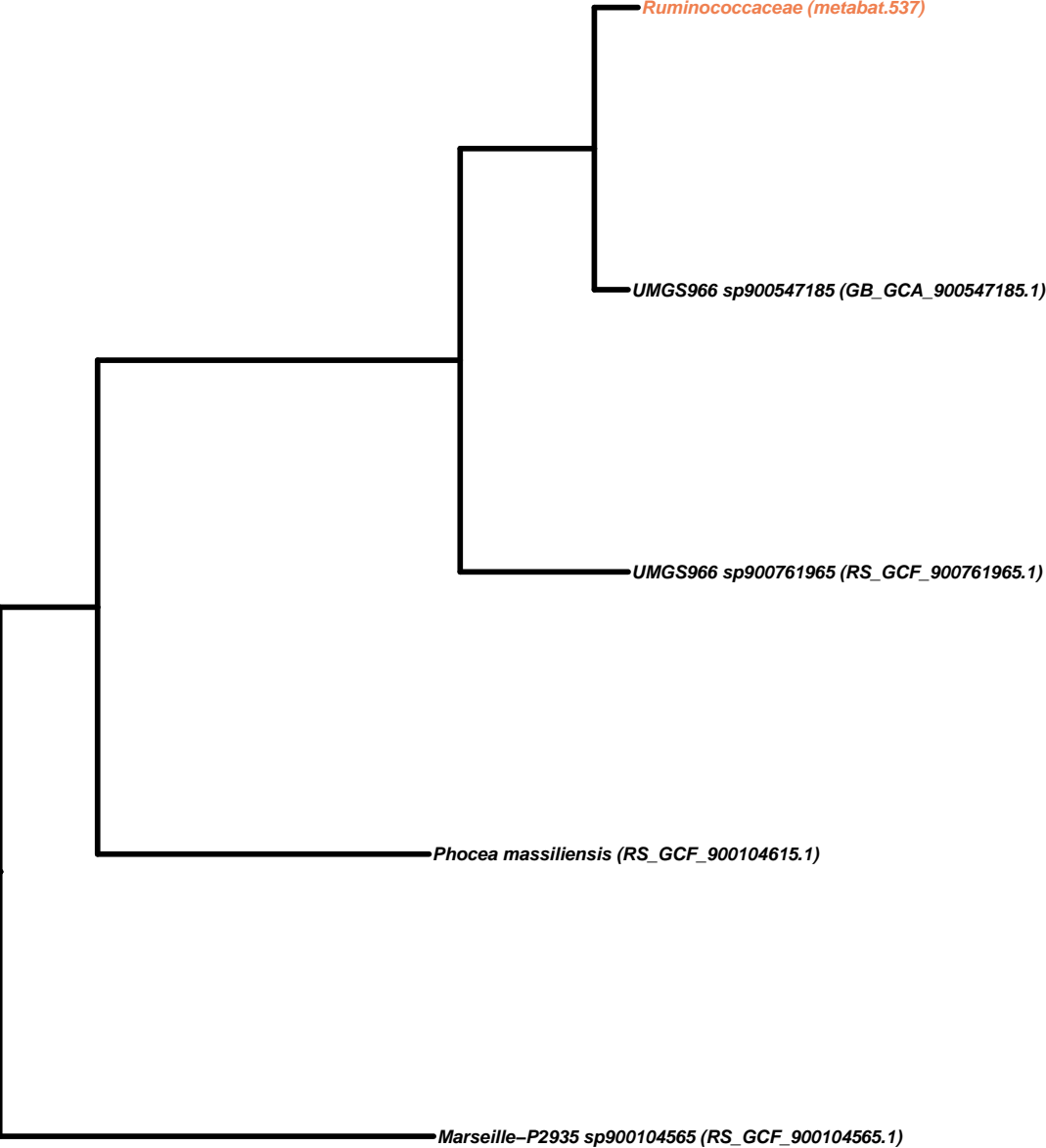

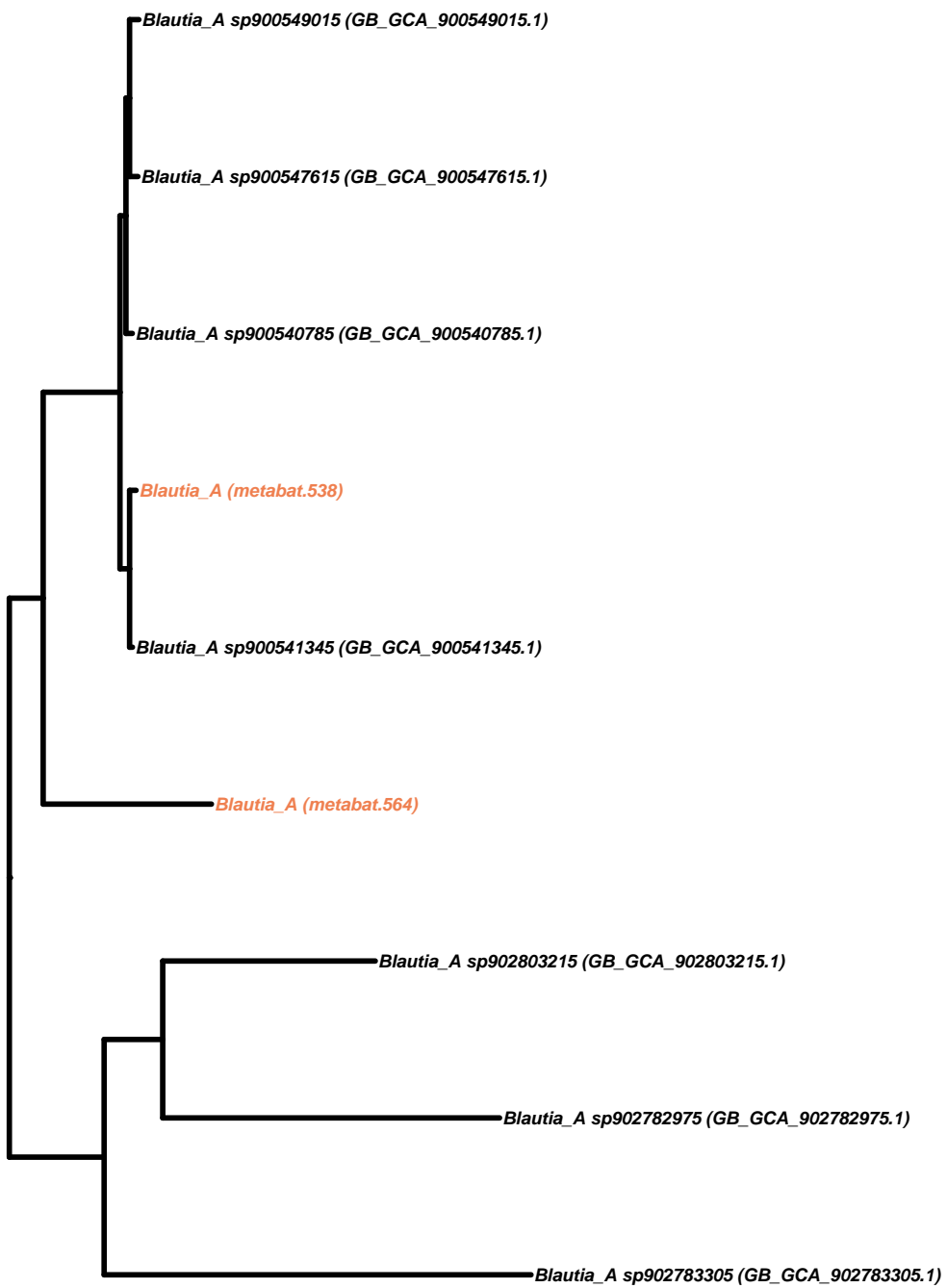

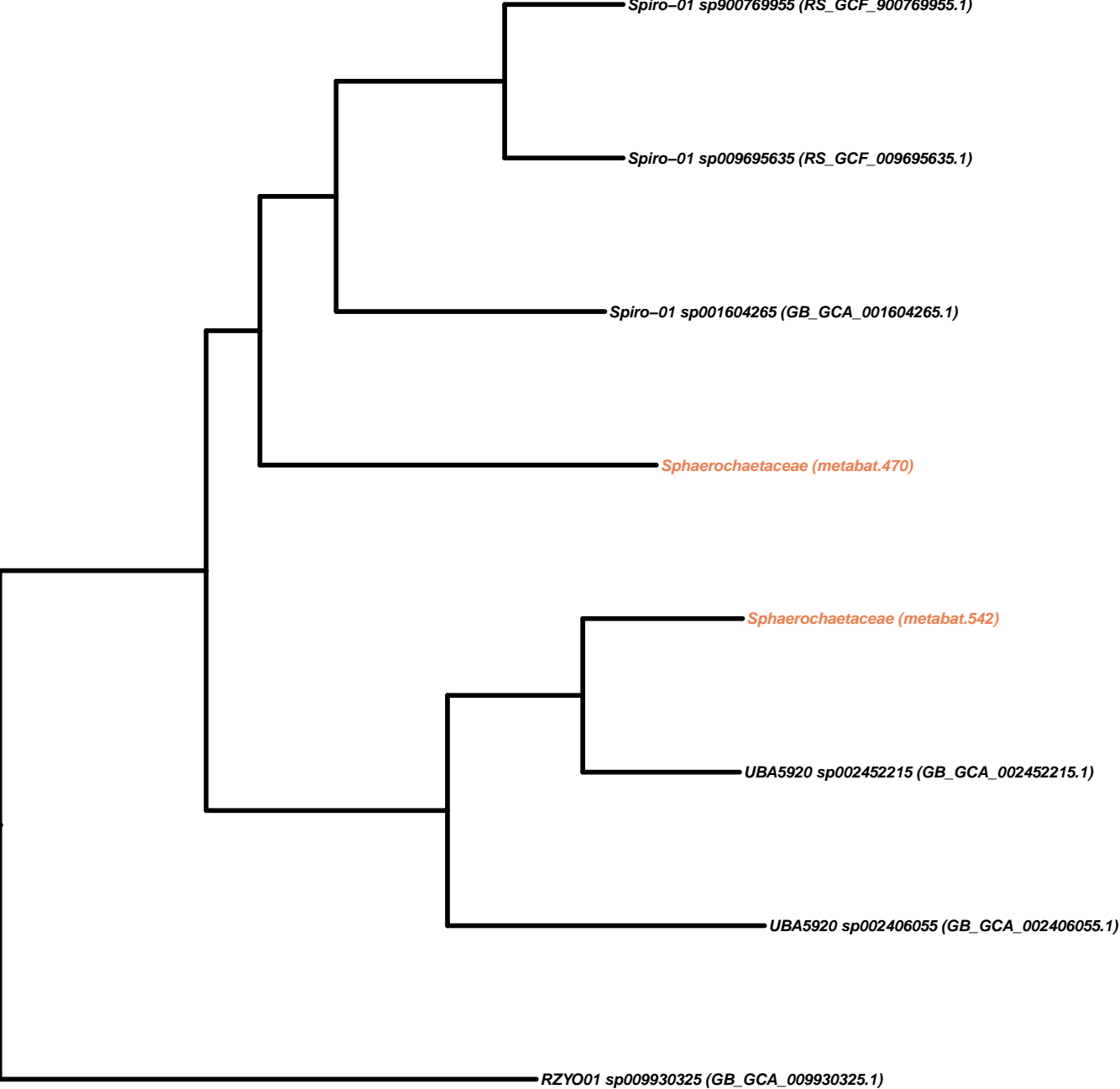

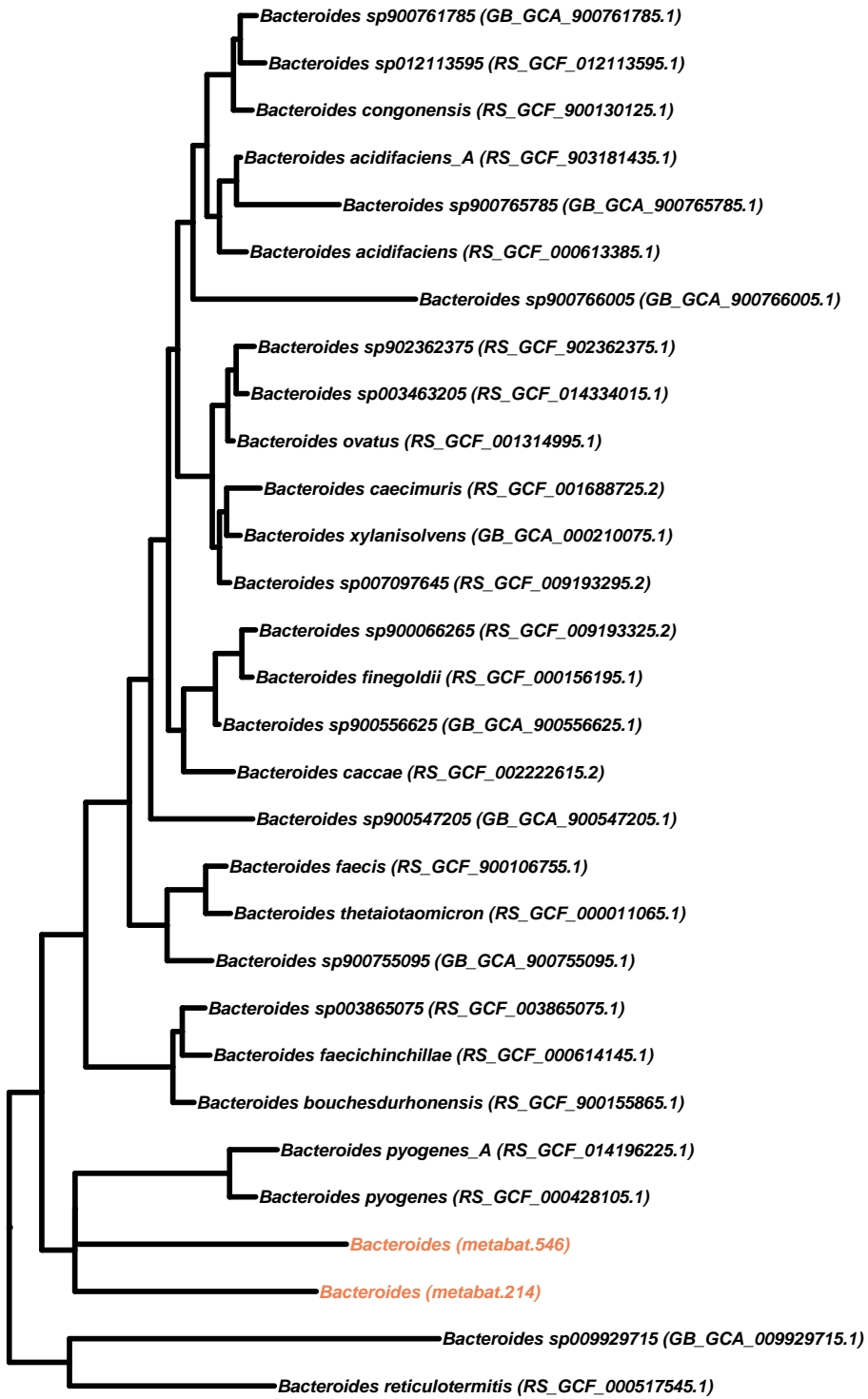

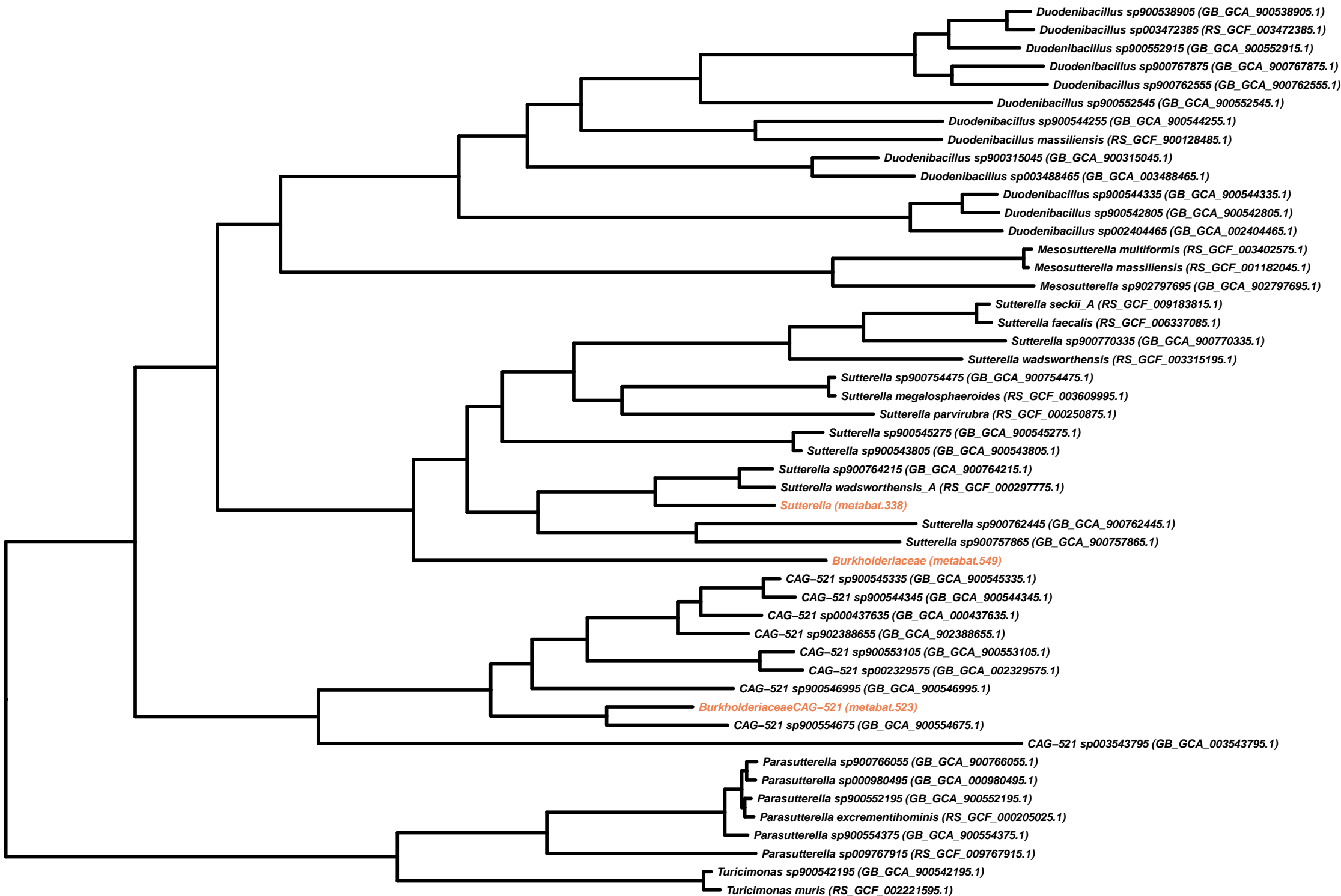

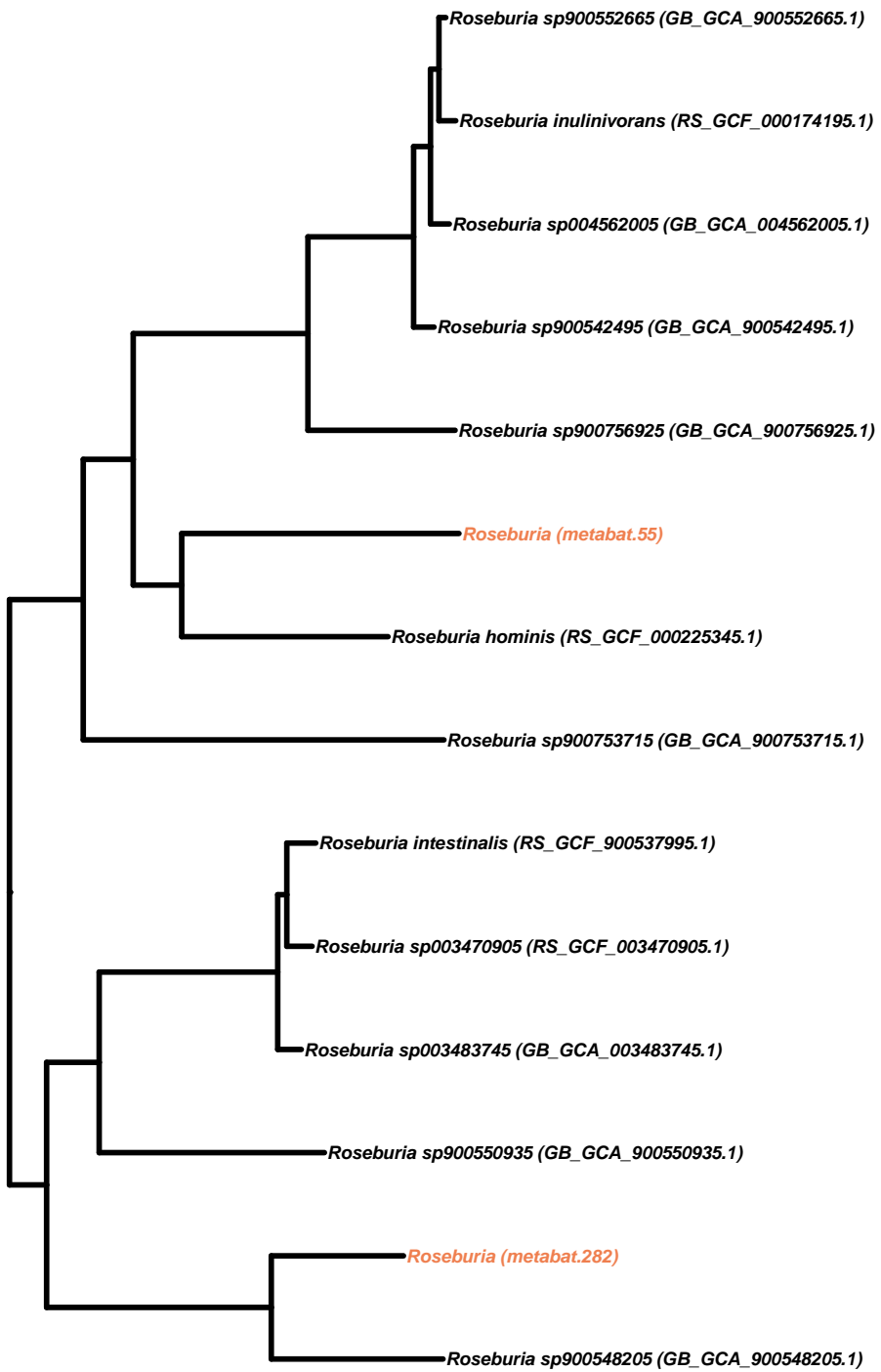

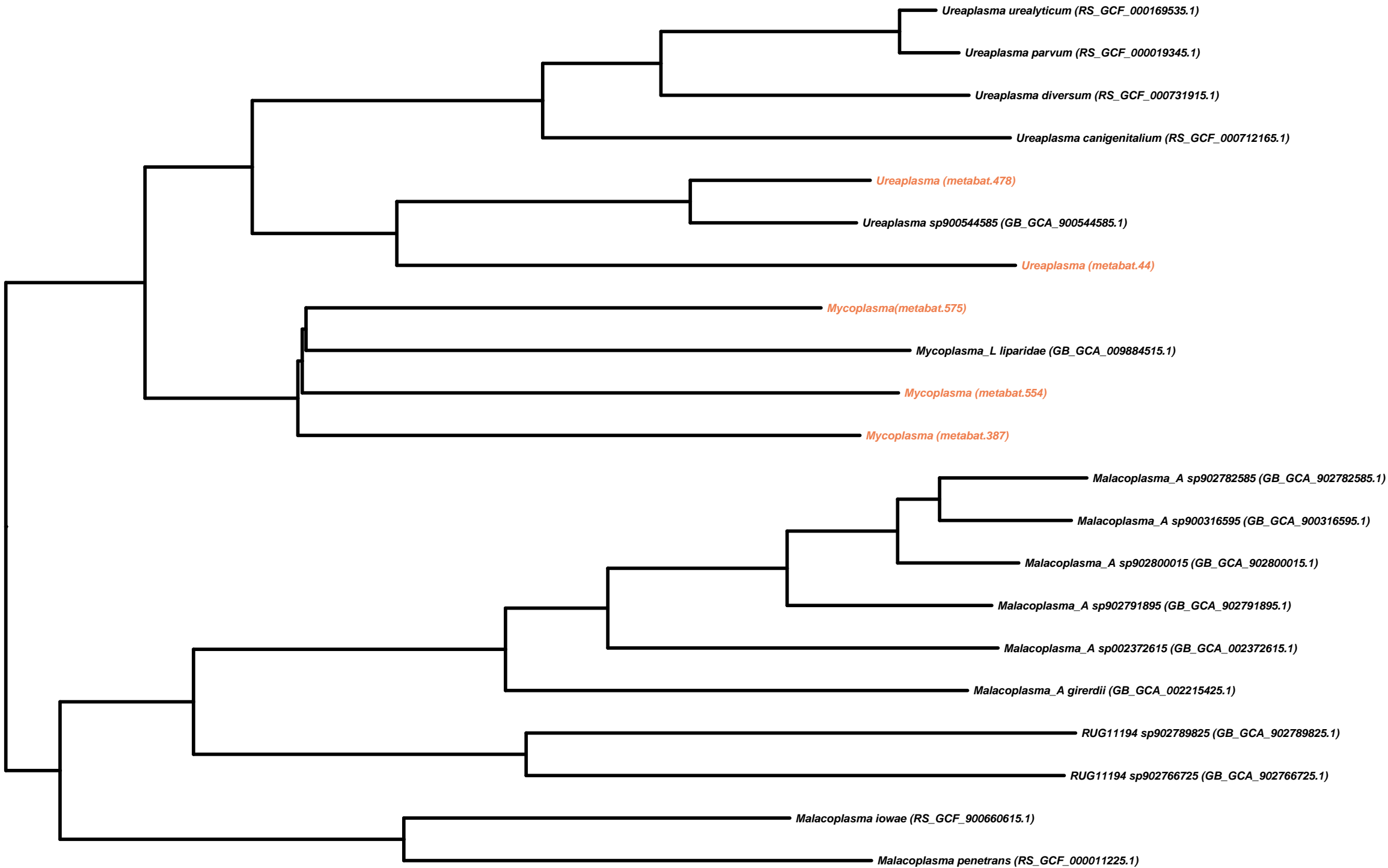

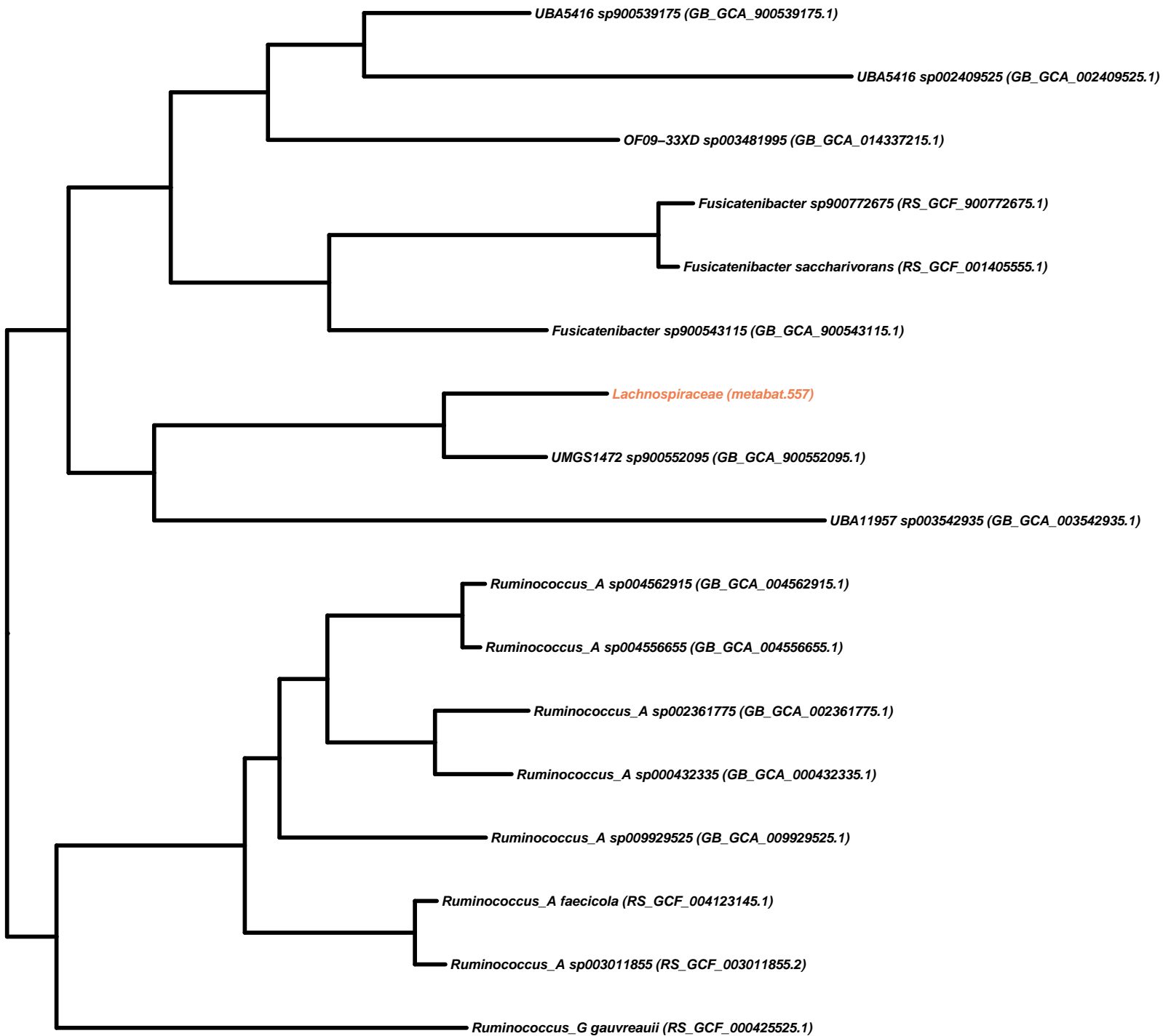

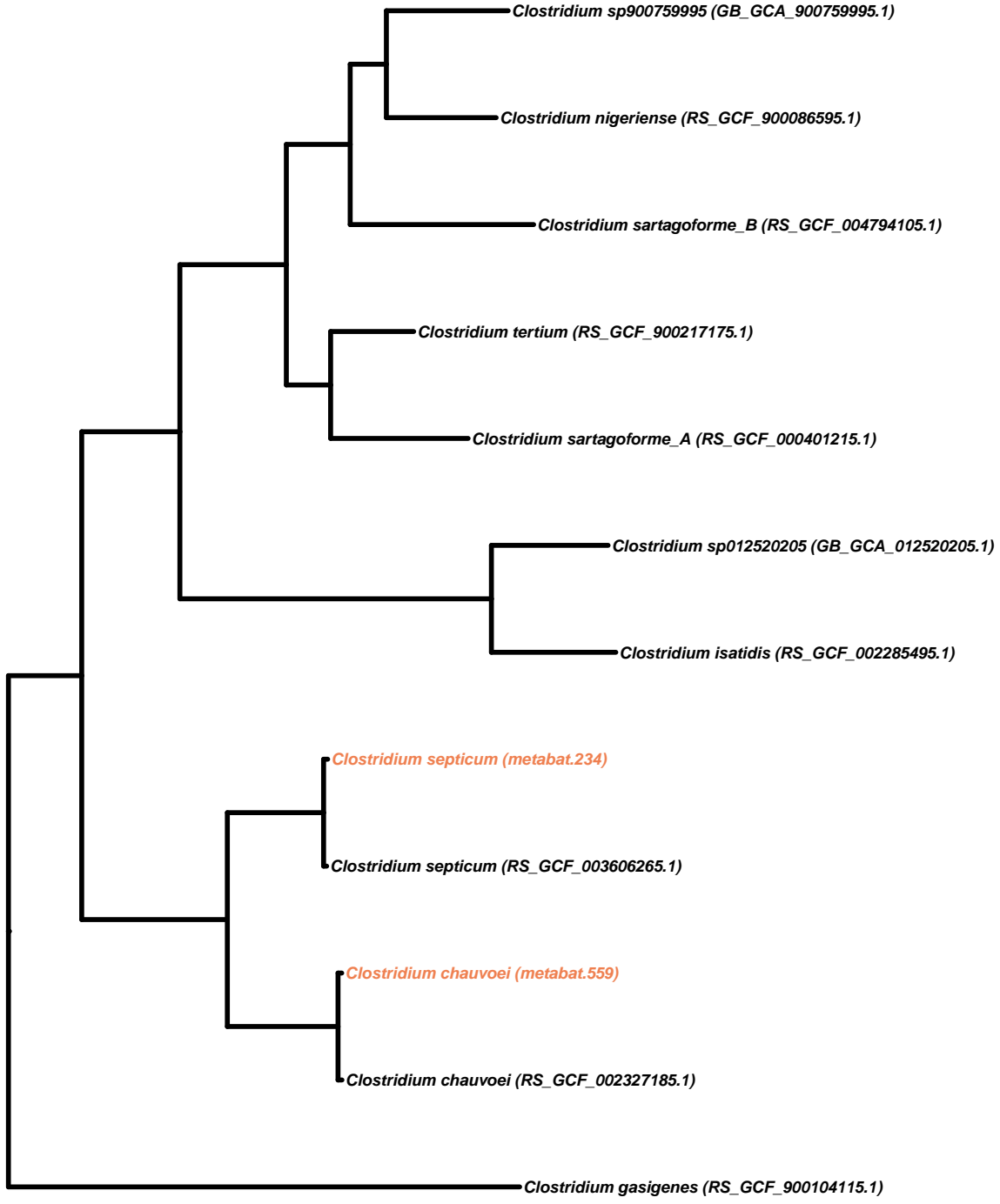

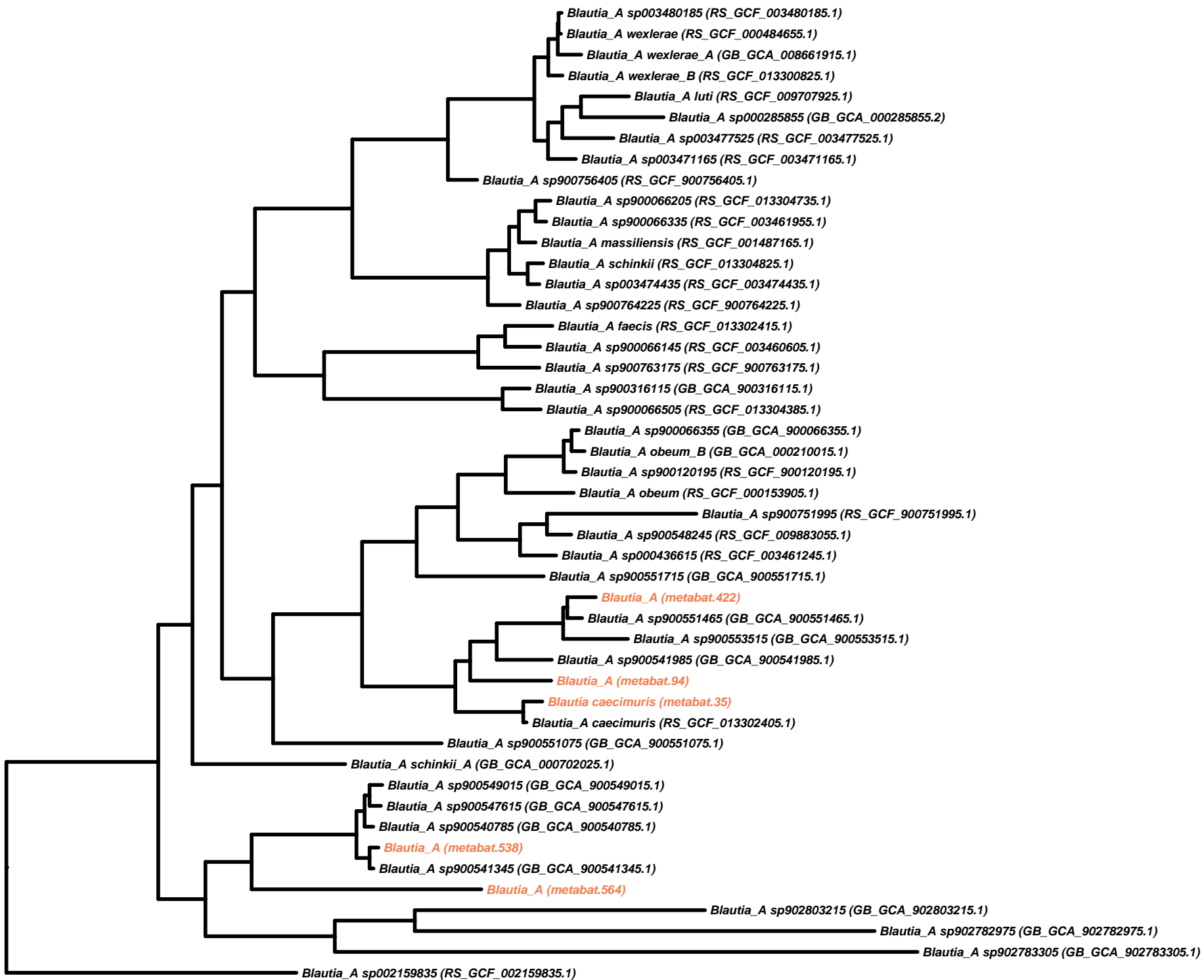

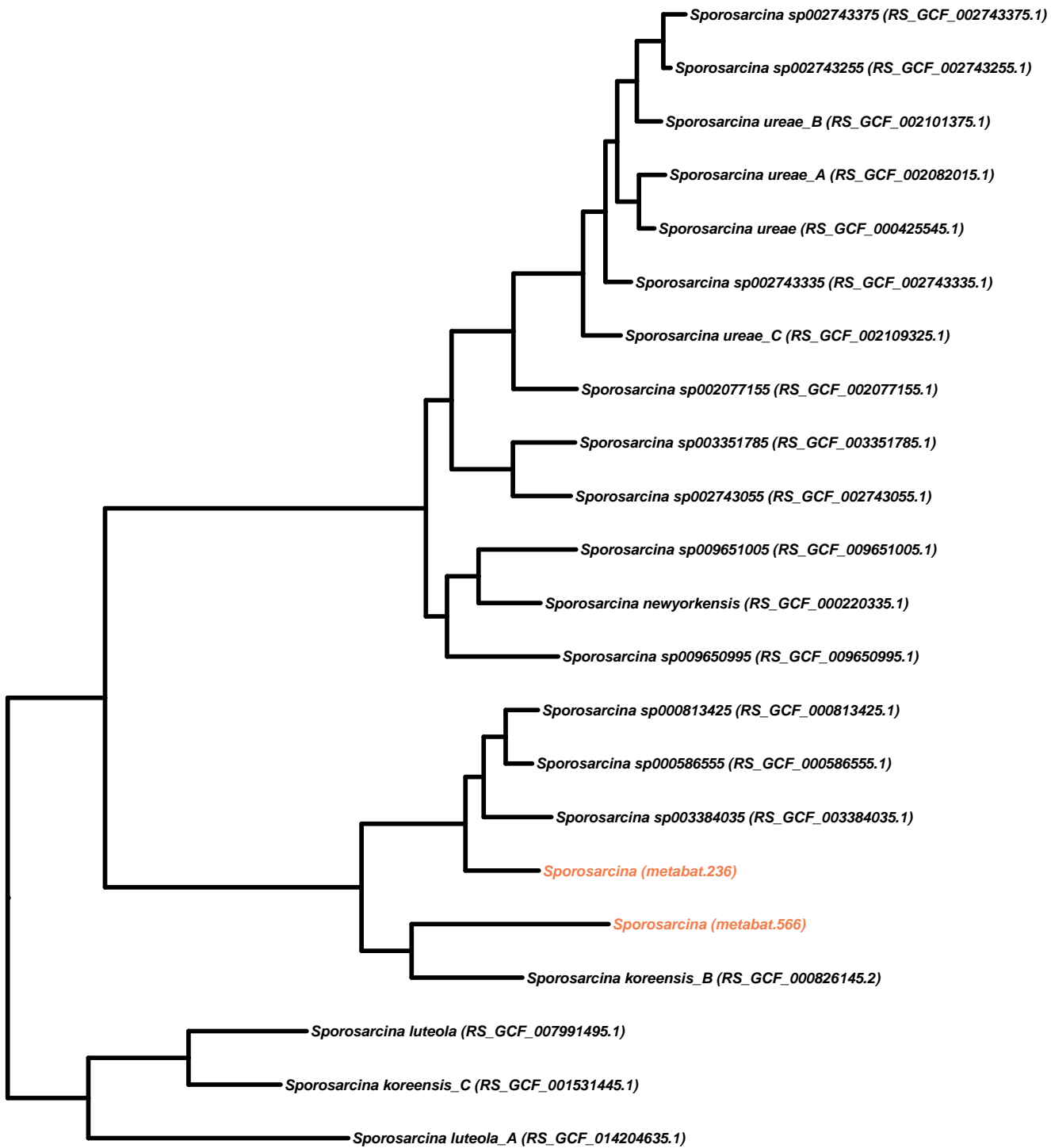

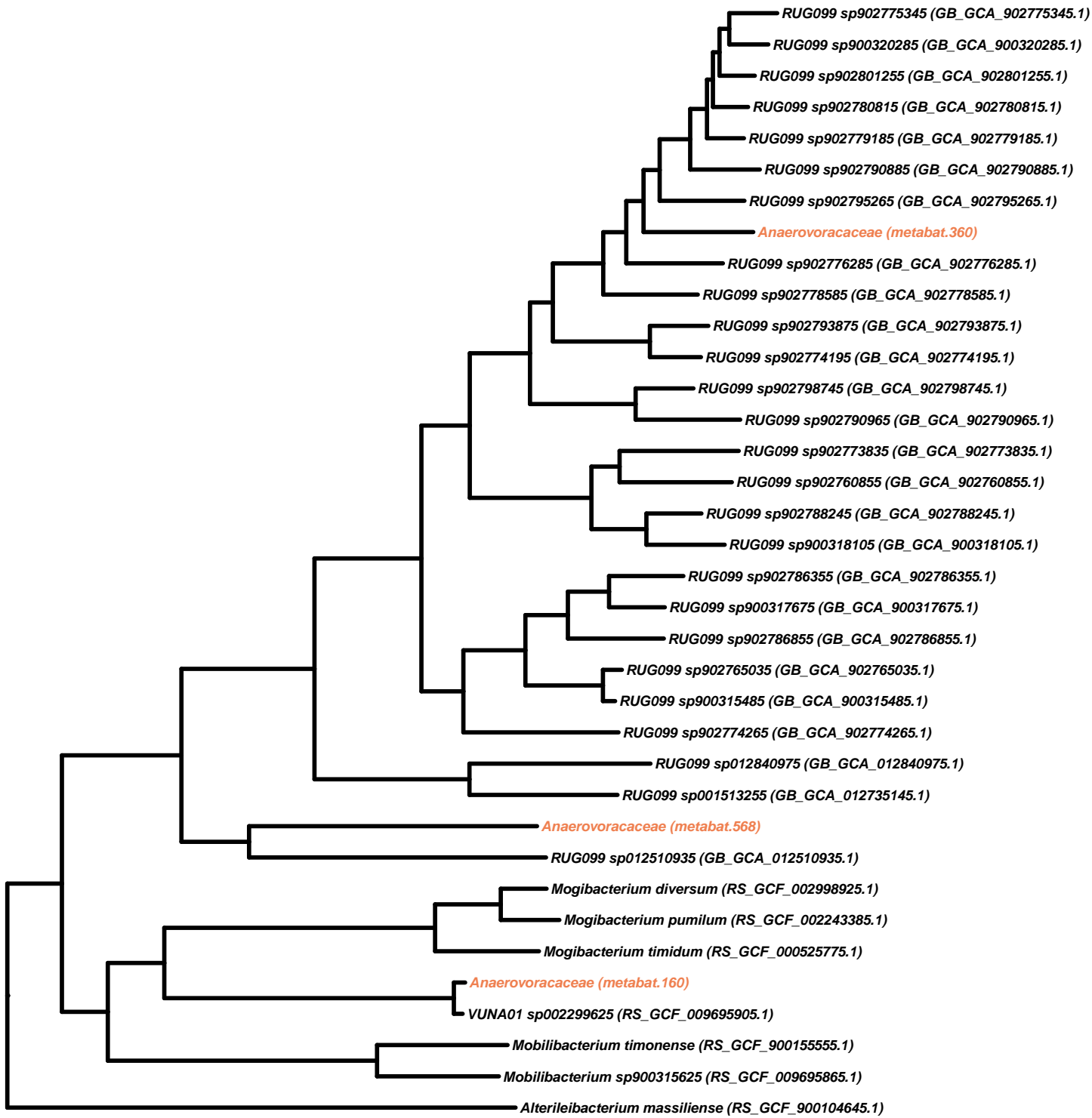

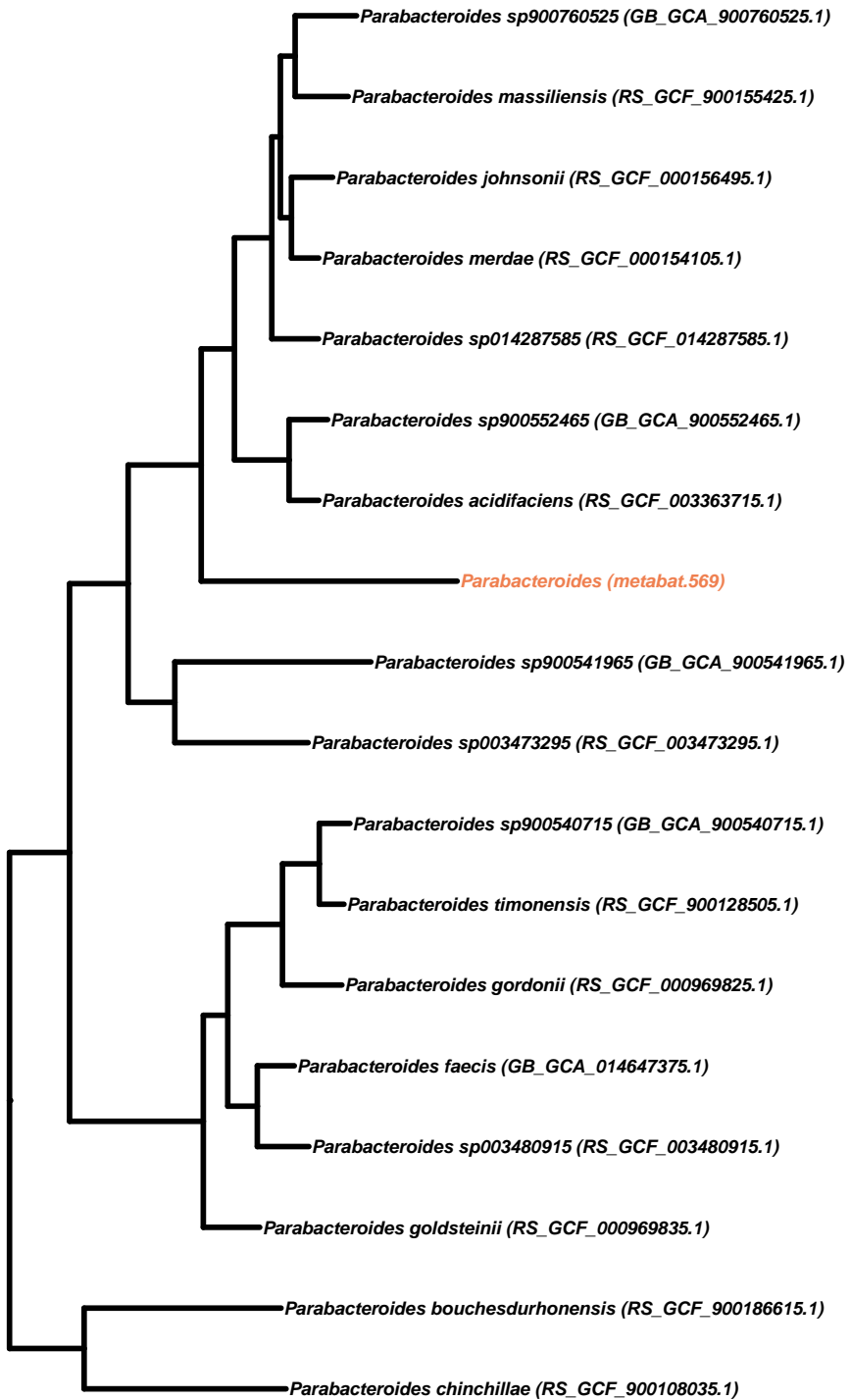

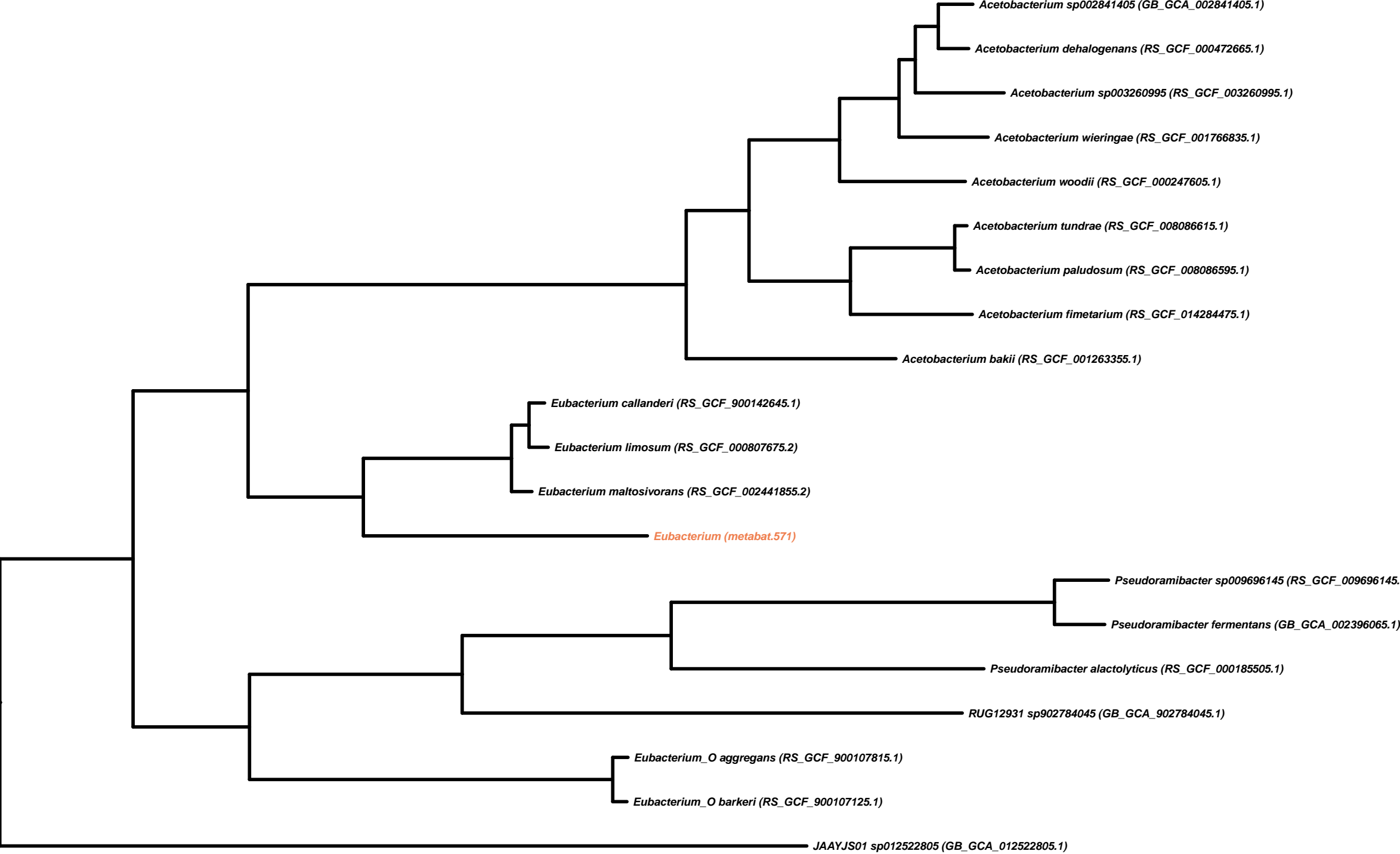

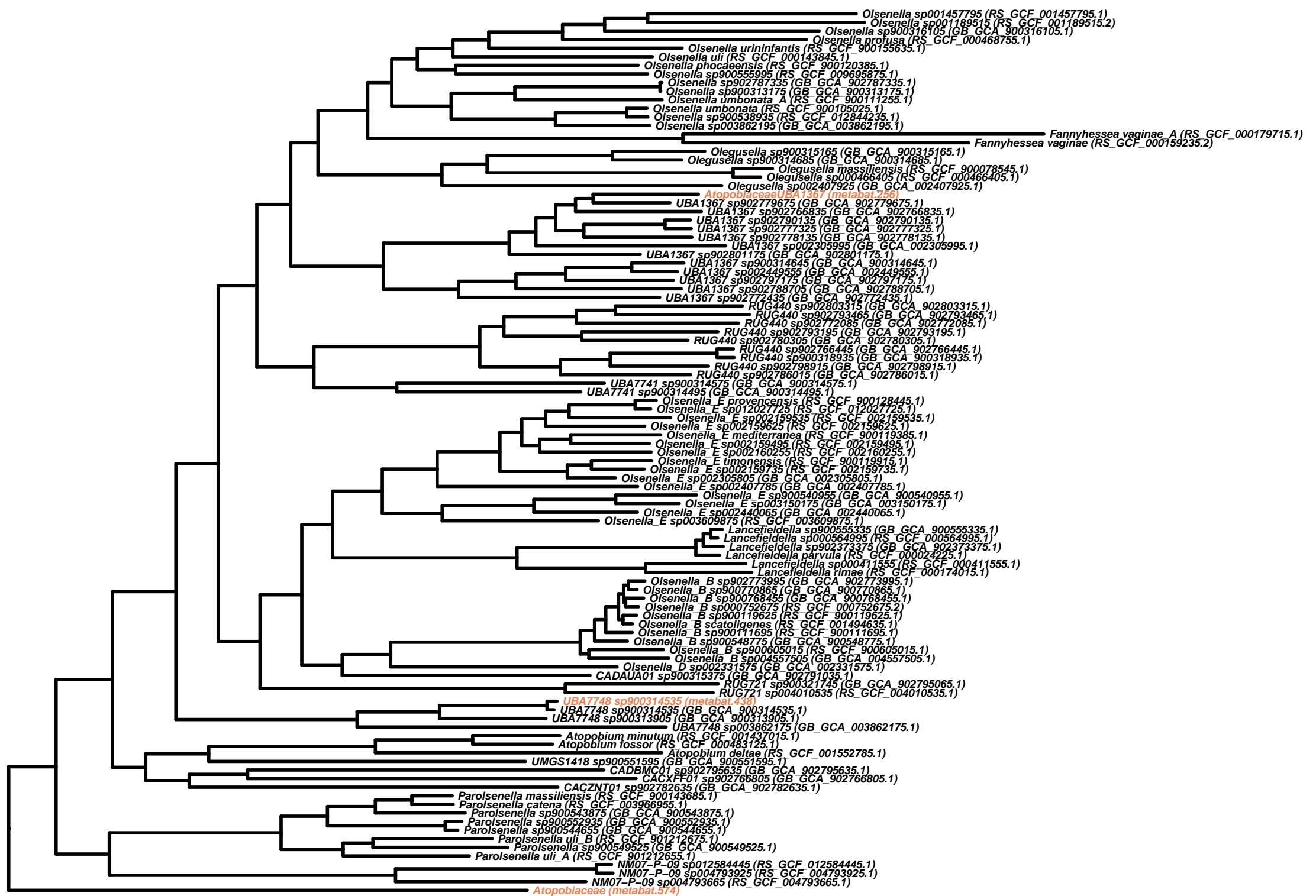

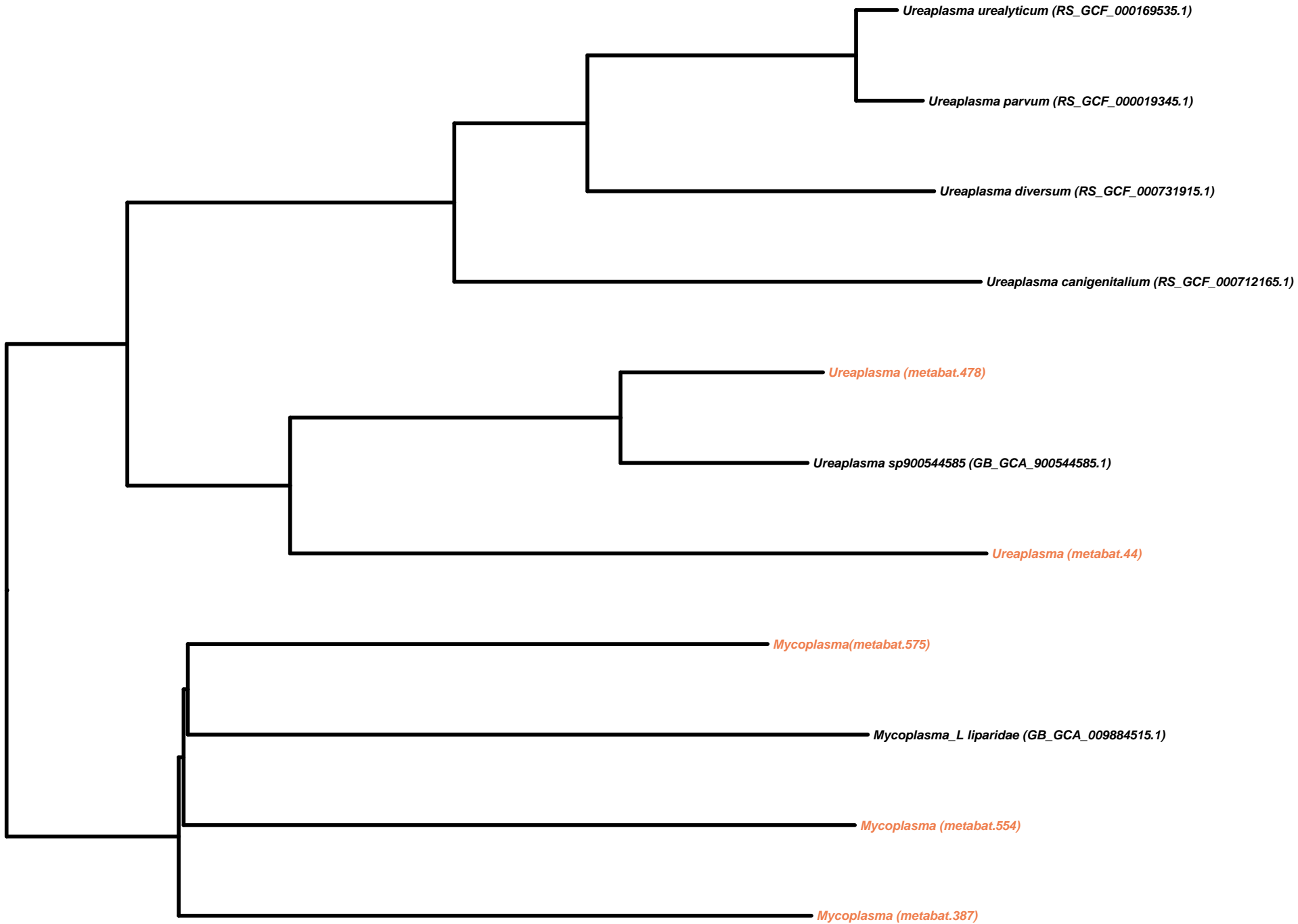

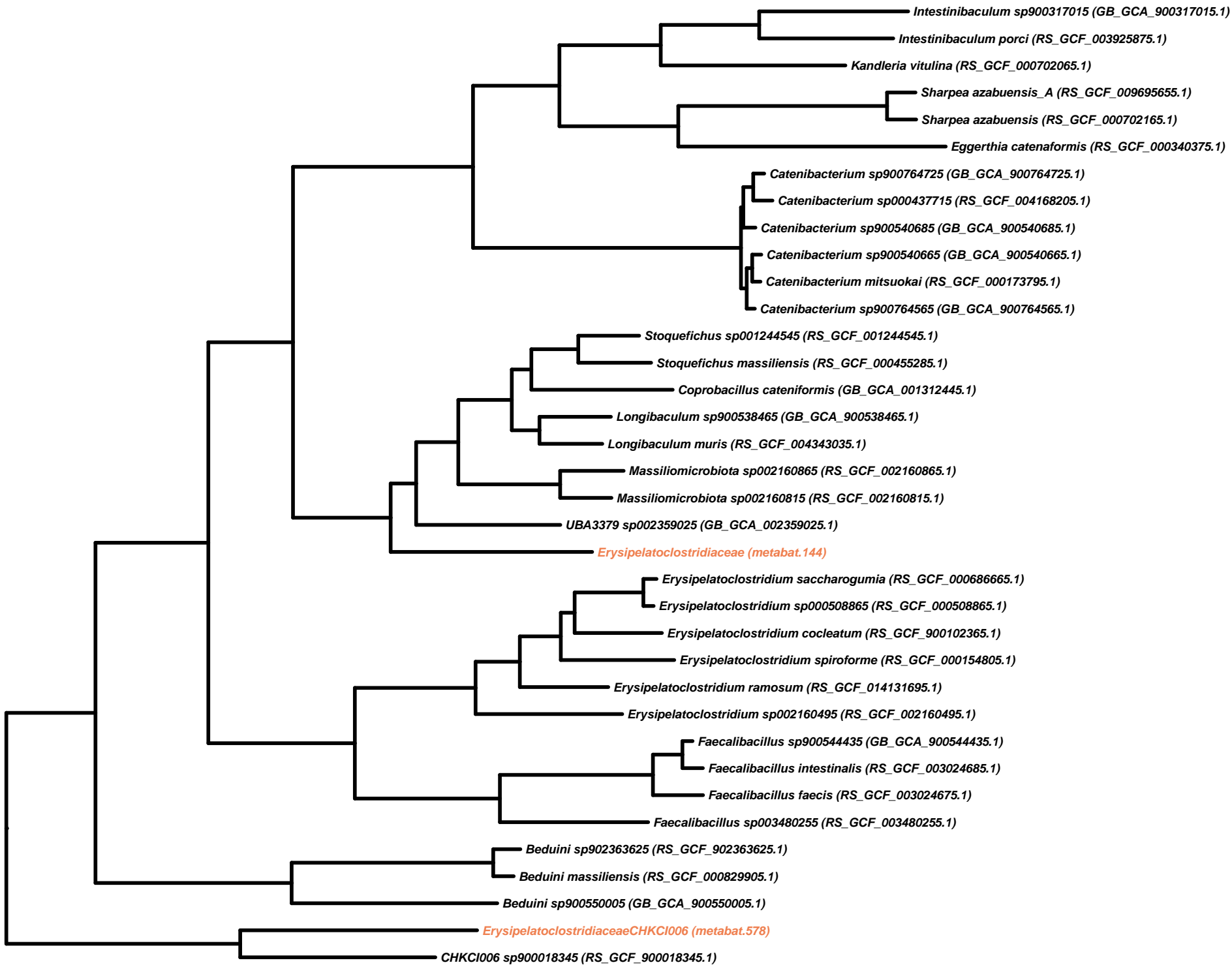

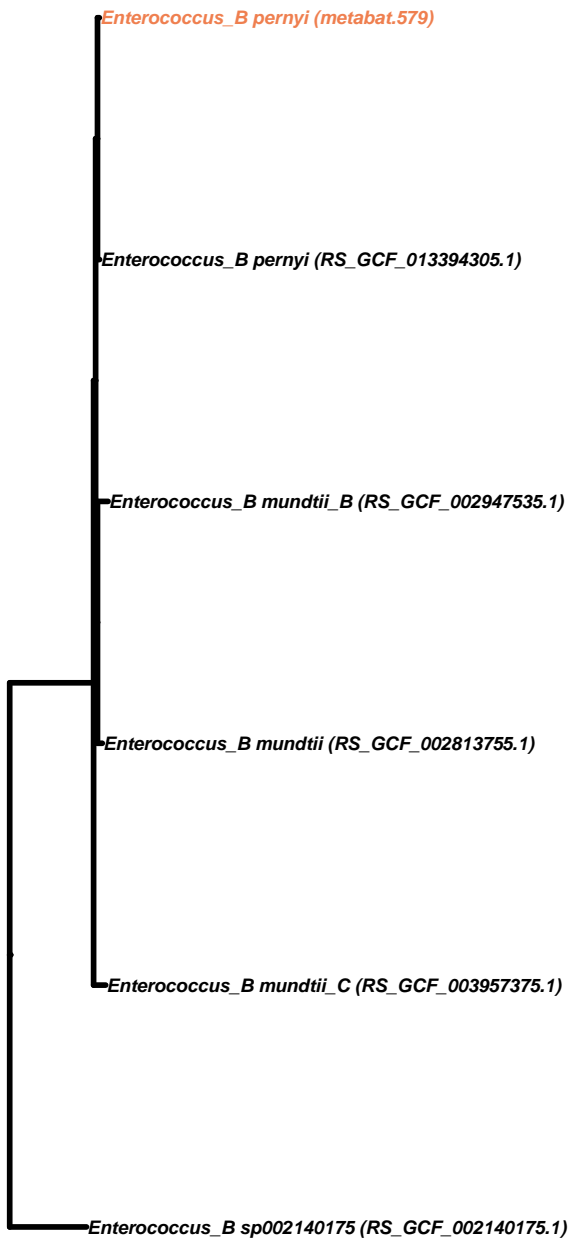

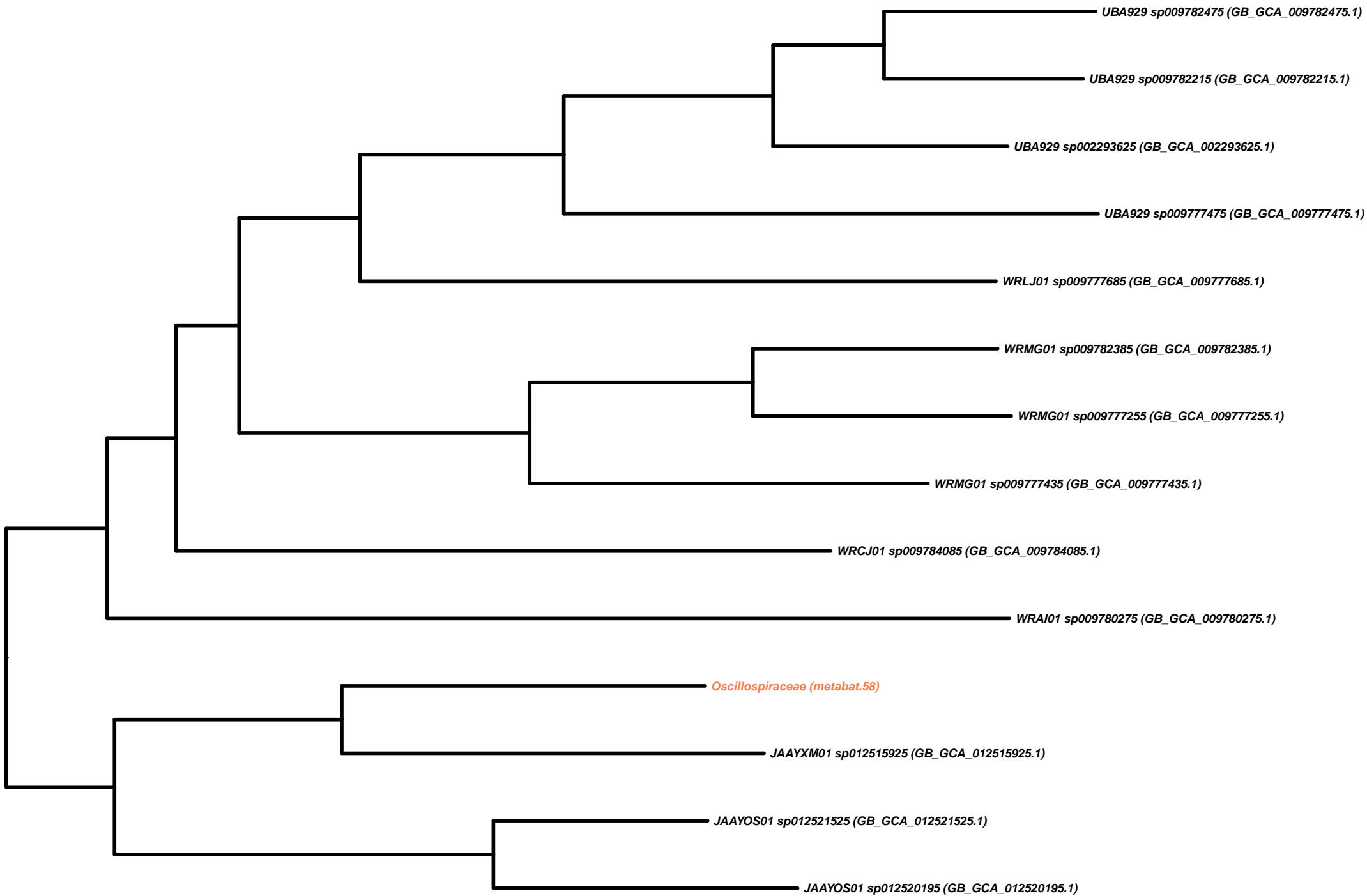

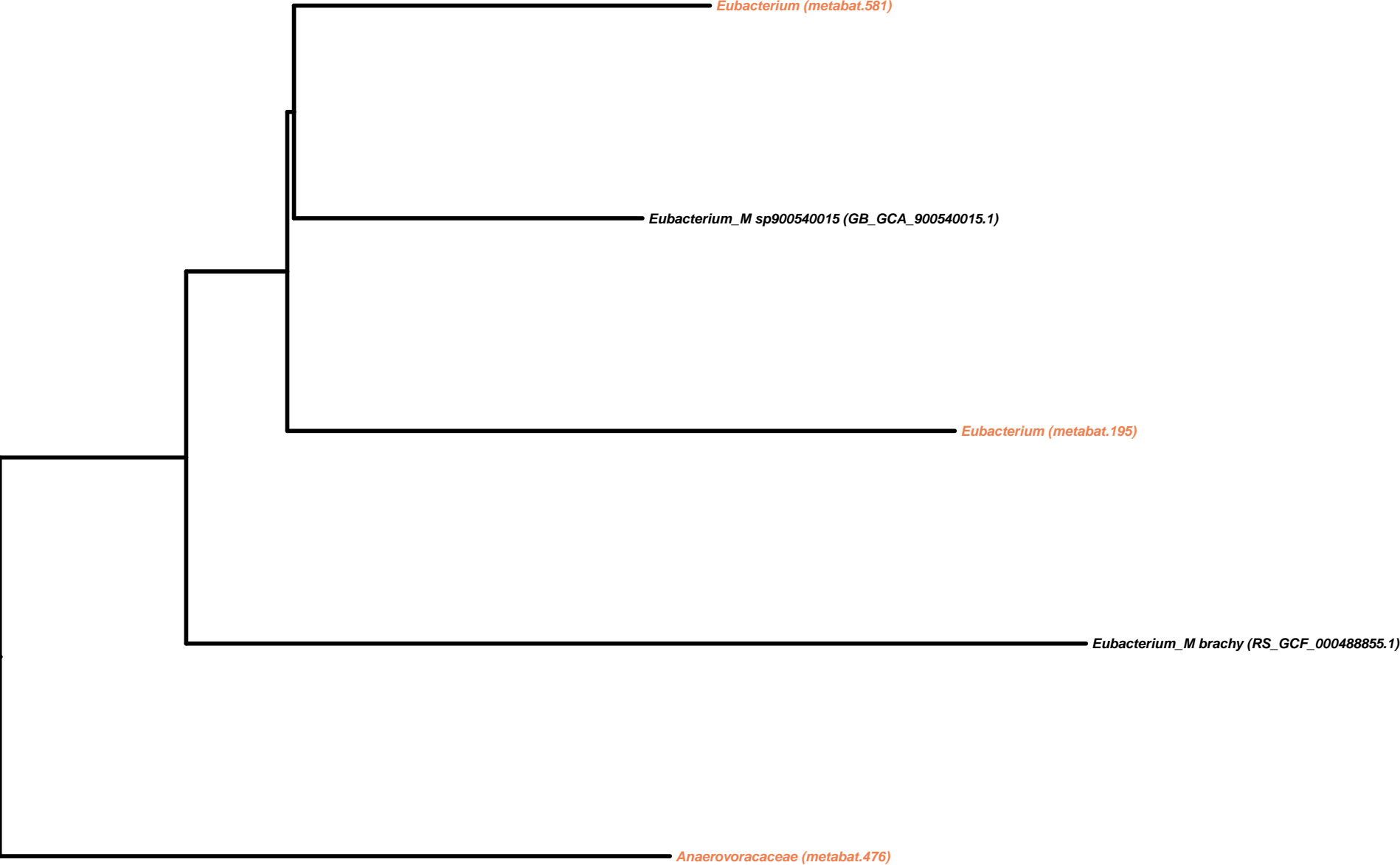

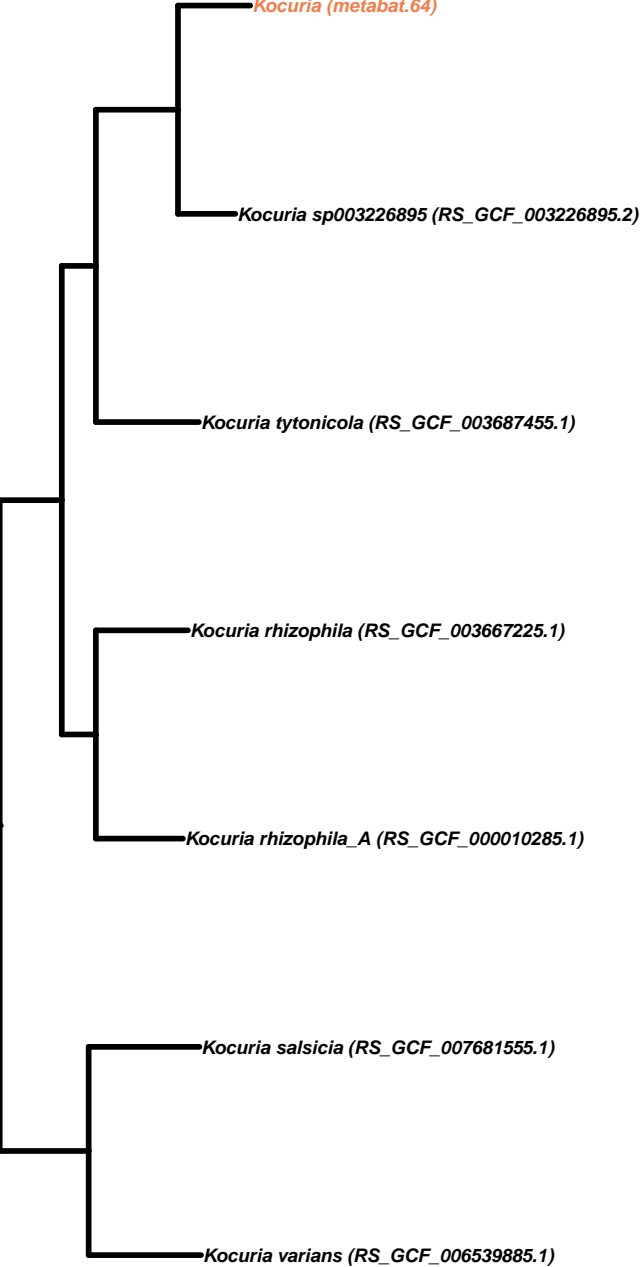

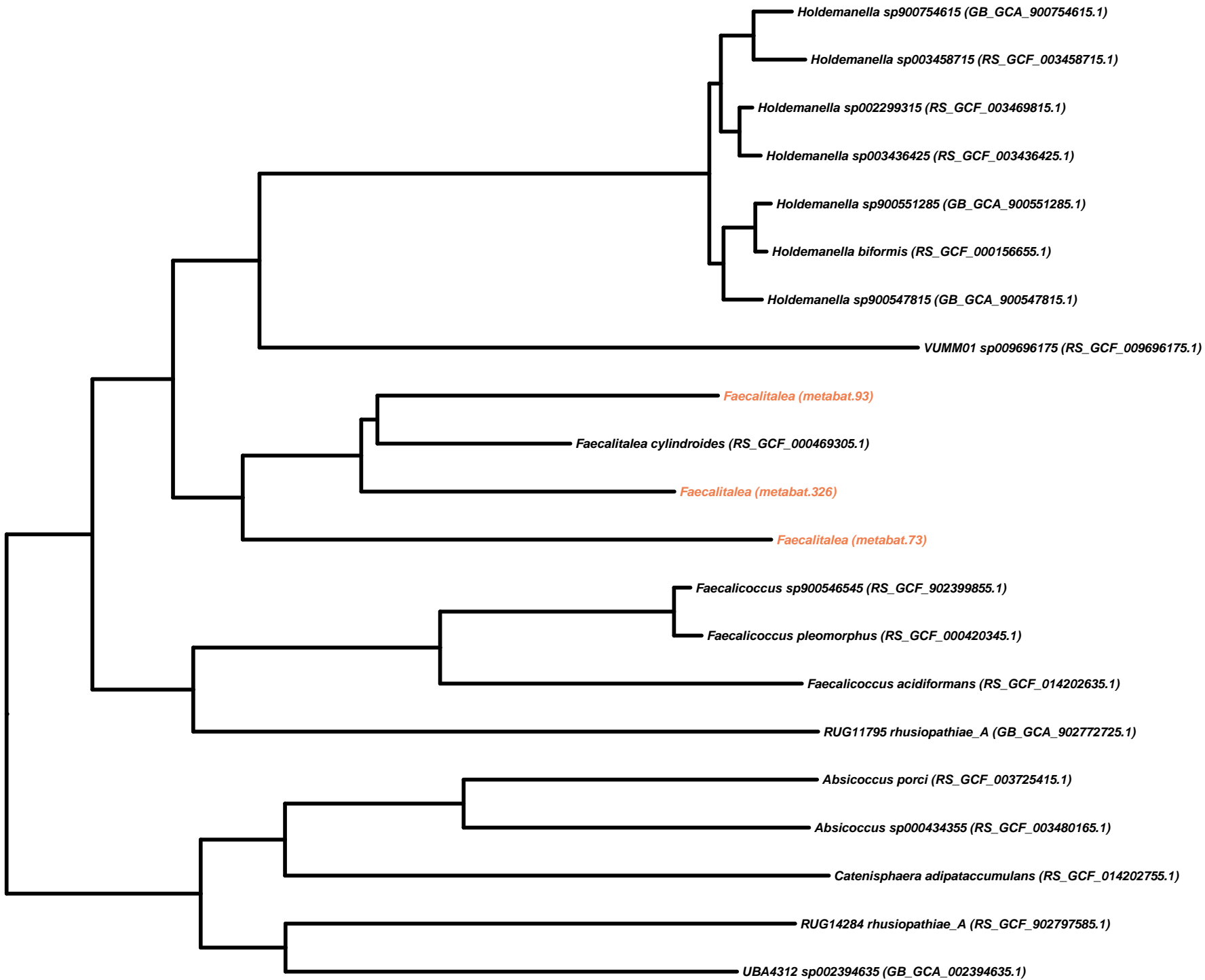

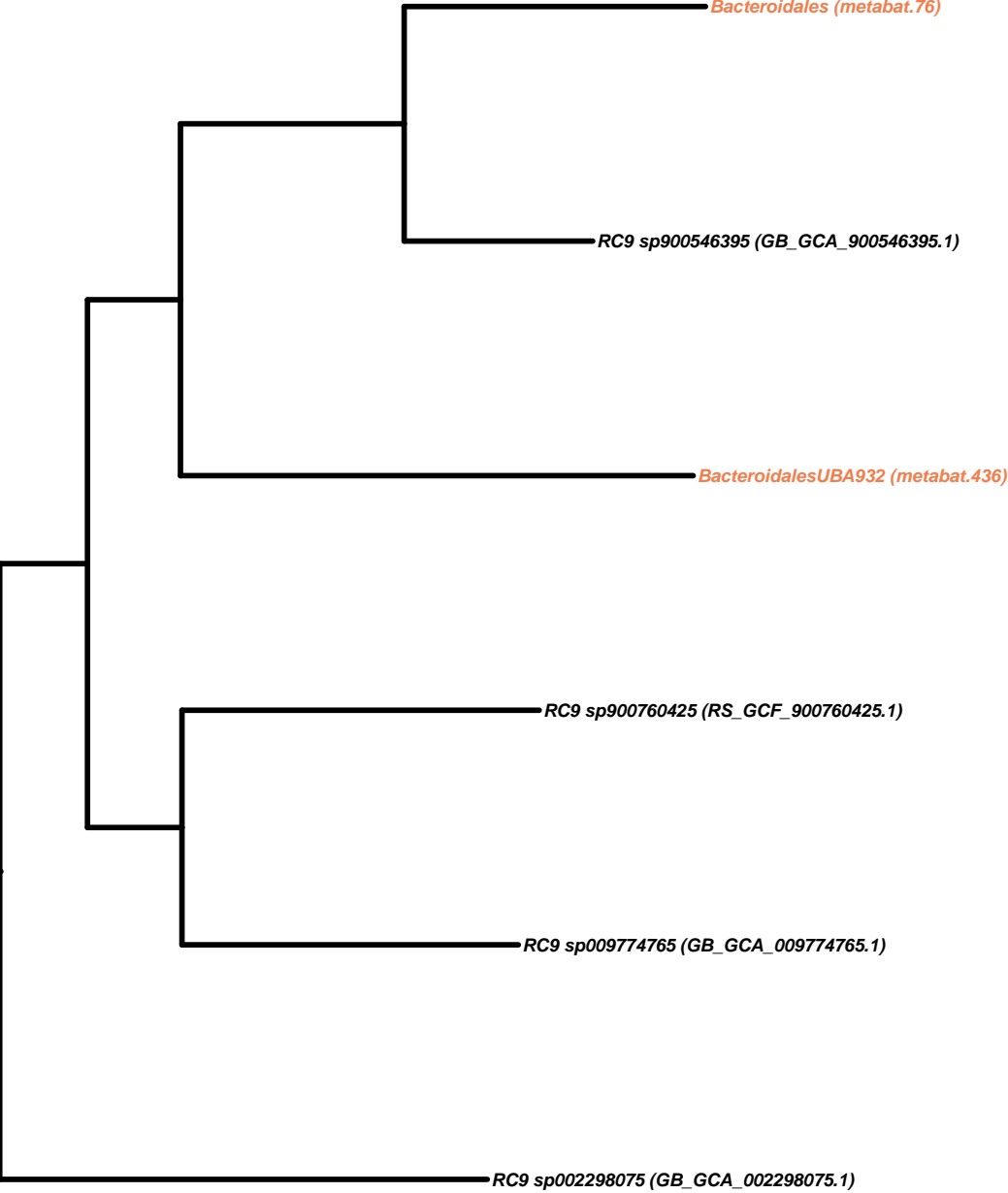

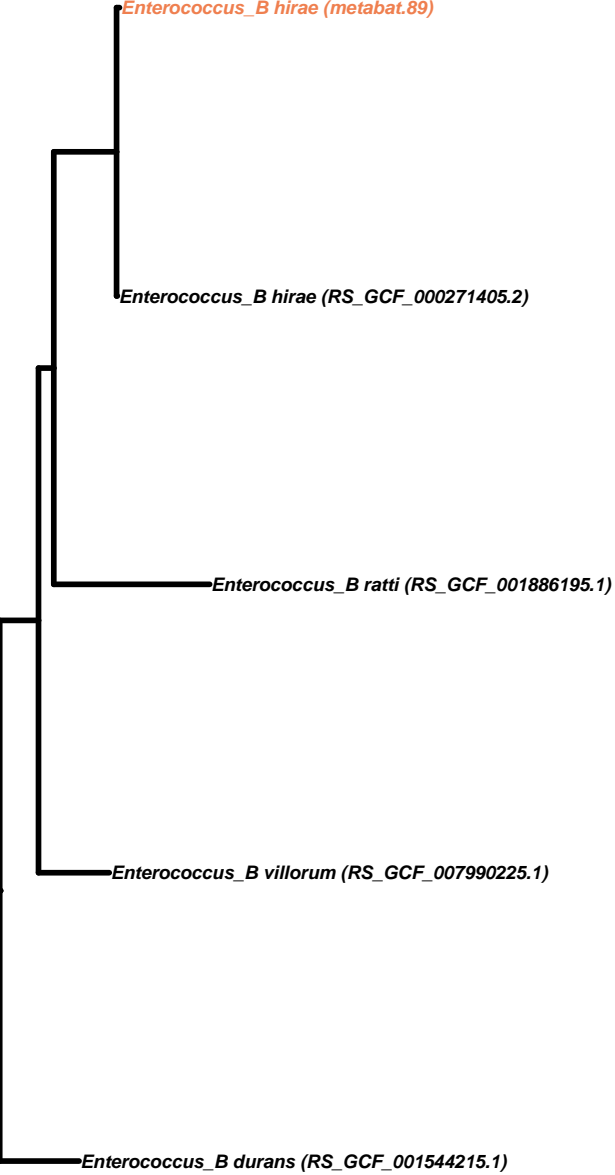

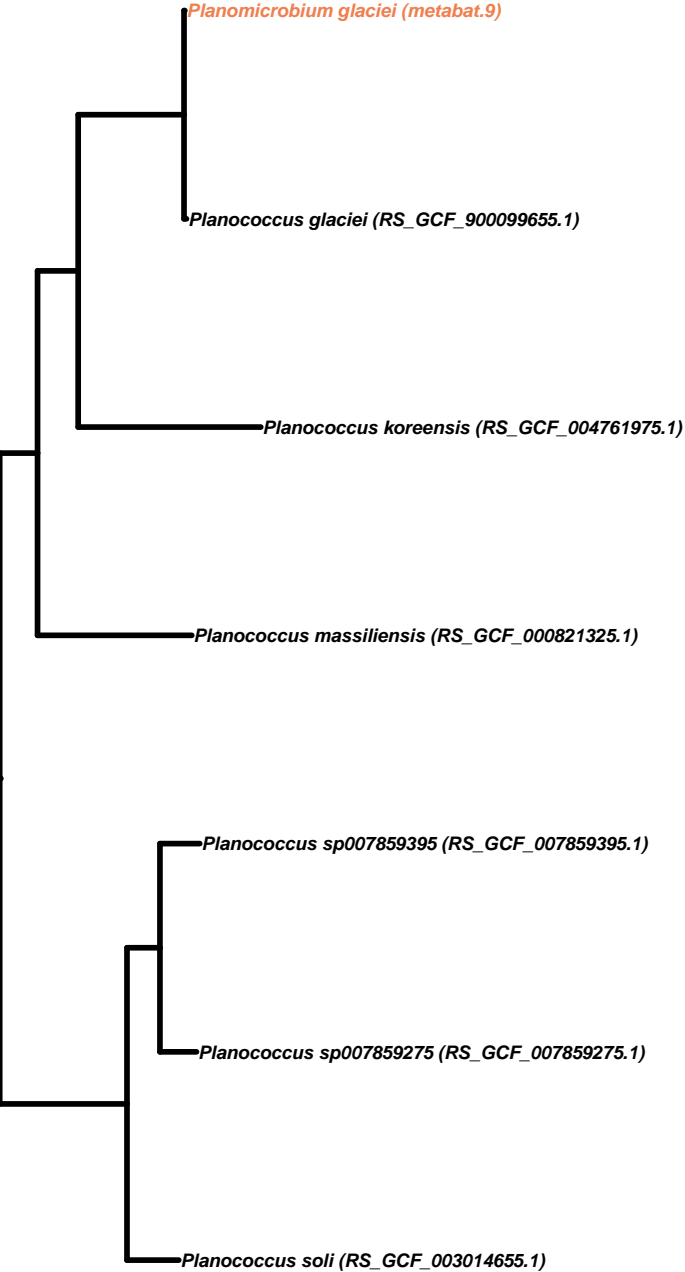

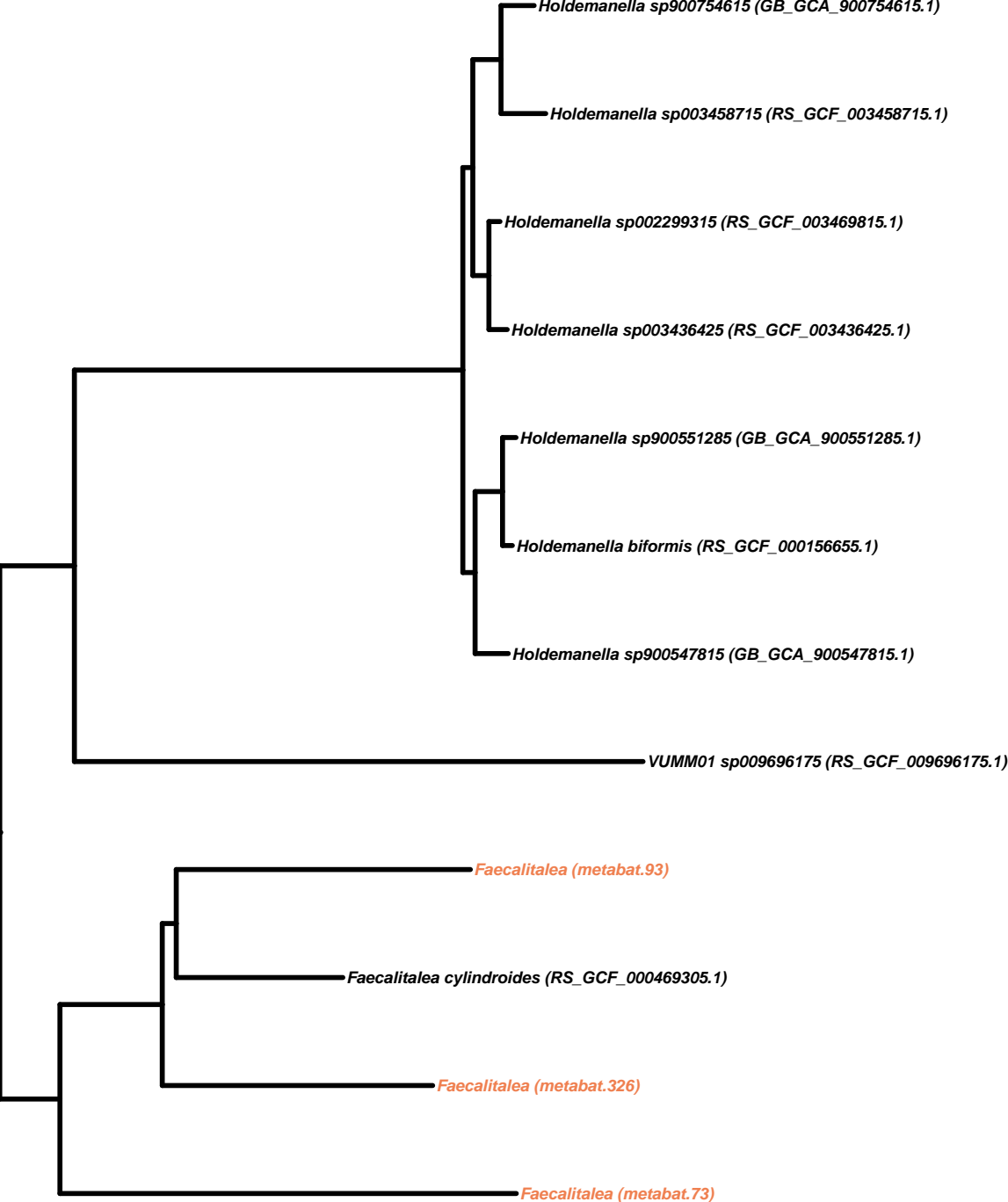

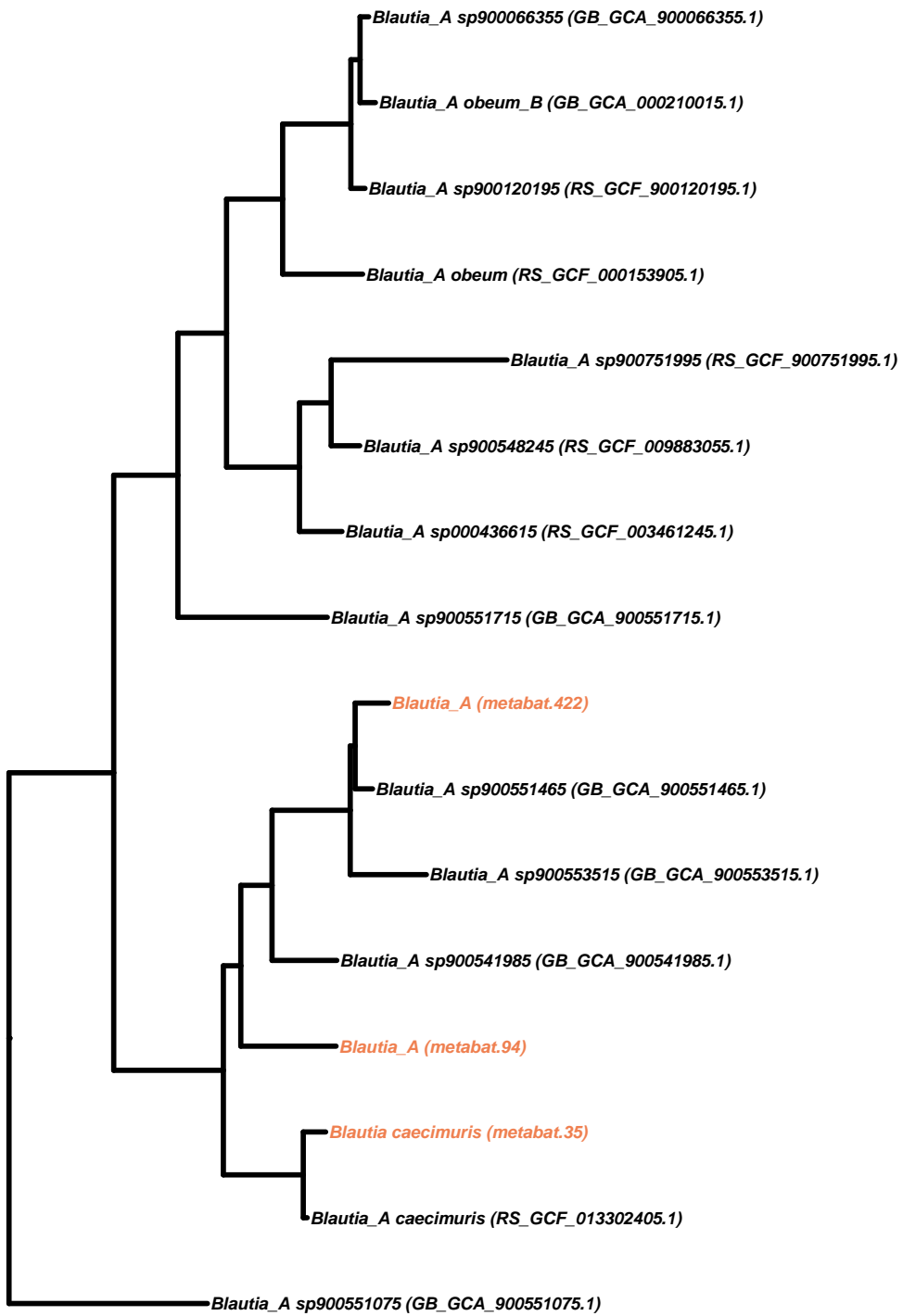

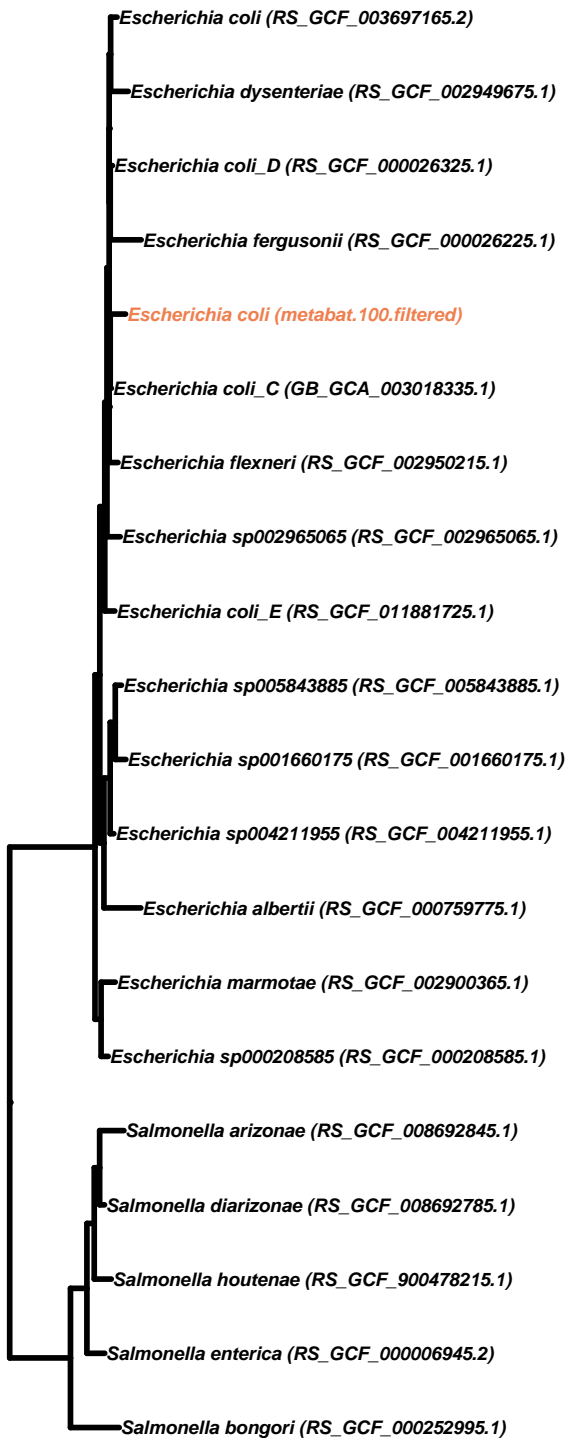

Supplement: FIG S2 [file msystems.00965-22-s0006.pdf]
